# Drug-target interactions prediction using marginalized denoising model on heterogeneous networks

**DOI:** 10.1186/s12859-020-03662-8

**Published:** 2020-07-23

**Authors:** Chunyan Tang, Cheng Zhong, Danyang Chen, Jianyi Wang

**Affiliations:** 1 School of Computer Science and Engineering, South China University of Technology, Guangzhou, China; 2 School of Computer, Electronics and Information, Guangxi University, Nanning, China; 3 Medical College, Guangxi University, Nanning, China

**Keywords:** Drug-target interaction, Marginalized denoising model, Drug discovery prediction, Drug repositioning prediction

## Abstract

**Background:**

Drugs achieve pharmacological functions by acting on target proteins. Identifying interactions between drugs and target proteins is an essential task in old drug repositioning and new drug discovery. To recommend new drug candidates and reposition existing drugs, computational approaches are commonly adopted. Compared with the wet-lab experiments, the computational approaches have lower cost for drug discovery and provides effective guidance in the subsequent experimental verification. How to integrate different types of biological data and handle the sparsity of drug-target interaction data are still great challenges.

**Results:**

In this paper, we propose a novel drug-target interactions (DTIs) prediction method incorporating marginalized denoising model on heterogeneous networks with association index kernel matrix and latent global association. The experimental results on benchmark datasets and new compiled datasets indicate that compared to other existing methods, our method achieves higher scores of *AUC* (area under curve of receiver operating characteristic) and larger values of *AUPR* (area under precision-recall curve).

**Conclusions:**

The performance improvement in our method depends on the association index kernel matrix and the latent global association. The association index kernel matrix calculates the sharing relationship between drugs and targets. The latent global associations address the false positive issue caused by network link sparsity. Our method can provide a useful approach to recommend new drug candidates and reposition existing drugs.

## Background

Identifying drug-target interactions (DTIs) is a critical work in drug discovery and drug repositioning. Although high-throughput screening and other biological assays are becoming available, the experimental methods for DTIs identification remain to be extremely costly. Different computational methods for predicting potential DTIs were proposed in the past decade [1–5].

In 2008, Yamanishi et al. [6] proposed a bipartite network model by integrating the chemical and genomic spaces to predict DTIs for four classes of target proteins, which are enzymes, ion channels (IC), G protein-coupled receptors (GPCR), and nuclear receptors (NR). Based on the four datasets, several methods to improve the accuracy of DTIs prediction were proposed. In early studies, the DTI prediction problem was treated as a binary classification problem. Some classical classifiers such as support vector machines (SVM) and regularized least squares (RLS) were used to predict drug-target interactions. A supervised bipartite local model (BLM) using SVM classifier was proposed to predict drug and target sets respectively [7]. To solve the problem of selecting negative samples, a semi-supervised learning method called Laplacian Regularized Least Squares (LapRLS) was proposed [8]. To analyze the relevance between the network topological information and DTIs prediction, a Gaussian interaction profile (GIP) kernel was defined to capture the topological information in DTIs network. And a Regularized Least Squares (RLS) classifier was employed with GIP kernel to predict DTIs [9]. The methods mentioned above focus on existing drug-target interaction pairs and mainly deal with the old drug reposition problem.

To predict new drugs or targets, a bipartite local model with neighbor-based interaction profile inferring (BLM-NII) was proposed [10]. A weighted nearest neighbor (WNN) profile and the GIP kernel were incorporated to handle new drug compounds [11]. A robust model against the overfitting problem of traditional statistical methods was proposed base on the Random Forest (RF) method [12]. Matrix factorization (MF) method is a feature extraction method widely used in recommendation system [13]. The MF method was used to identify latent features of drugs and targets to handle new drug discovery problem [14–19]. Zheng et al. [15] used collaborative matrix factorization (CMF) to predict potential DTIs. Liu et al. [17] used the logistic matrix factorization and neighborhood information of drugs and targets to predict DTIs. There are also some methods which form the final kernel matrix using the linear combination of two or more kernel matrices [7–9, 15, 17]. Hao et al. [18] combined different kernel matrix with nonlinear kernel diffusion, and employed the diffused kernel matrix with RLS classifier to predict DTIs. Hao et al. [19] integrated logistic matrix factorization and kernel diffusion to improve the accuracy of DTIs prediction. The model based on diffused kernel matrix outperforms the model based on the linearly weighted kernel matrix for DTIs prediction.

To predict more realistic drug-target interactions, some researchers used drug-target binding affinity. Binding affinity indicates the strength of interactions between drug-target pairs. Binding affinity is usually measured by the dissociation constant (*K*_*d*_), inhibition constant (*K*_*i*_), or the half maximal inhibitory concentration (IC50). Kronecker_rls [9, 20] is a method to predict drug-target binding affinity [21]. For more accurate prediction on continuous drug-target binding affinity data, a non-linear method called SimBoost was proposed by using the gradient boosting regression trees as the learning model [22].

With rapidly development of deep learning, some deep learning frameworks have been applied in the field of drug discovery [23–27]. Stacked auto-encoder was used to construct deep representation of drug-target pairs [24]. Hu et al. [25] used convolutional Neural Network (CNN) to predict DTIs. A new compound-protein interaction (CPIs) prediction approach was developed by combining graphical neural network (GNN) for compounds and convolutional neural network (CNN) for proteins [26]. Based on deep neural network (DNN), Tian et al. [27] proposed a method called DL_CPI to predict large-scale compound-protein interactions. Deep learning method has the advantages in dealing with growing compounds data. But analyzing deep learning models is difficult due to their black-box nature, more effective models are needed to improve the accuracy of DTIs prediction.

From the perspective of networks, the DTIs prediction problem can be treated as a network link prediction problem [28]. Chen et al. [29] developed a model of network-based random walk with restart on heterogeneous networks (NRWRH) to predict potential DTIs. Lan et al. [30] used the models of random walk with restart, *k* nearest neighbors (*k*NN), and heat kernel diffusion to label unknown DTIs to predict potential DTIs. Recently, Chen et al. used marginalized denoising model (MDM) to predict hidden or missing links in a given relational matrix by transforming a network link prediction problem to a matrix denoising problem [31]. The MDM-based method can predict new protein-protein interaction in the PPI network better than the MF-based methods. But the MDM-based predicting method has not been applied to the heterogeneous network such as drug-target interactions.

To further improve the accuracy of DTIs prediction, this paper proposes an integrated method using the marginalized denoising model on heterogeneous networks, association index and kernels fusion. We transform the DTIs prediction problem to a noise reduction problem on heterogeneous networks. The heterogeneous network is constructed by combining drug and target kernel matrices and the existing DTIs network. To construct the kernel matrix, we introduce the association index kernel matrix to measure the sharing interaction relationship between drugs and the sharing interaction relationship between targets. The sharing interaction relationship is derived from the common targets between drugs and the common drugs between targets. Furthermore, we not only use the information of associations of the nearest neighbors to perform DTIs prediction, but also incorporate the global association between drugs and targets to reduce the sparsity of DTIs network and improve prediction accuracy.

The rest of this paper is organized as follows. The experimental results are reported in section 2. The discussion of experimental results is given in section 3. The conclusion is given in section 4. The source of the benchmark dataset selected and new compiled dataset, construction of the matrices of similarity between drugs and similarity between targets, MDM model and our proposed prediction method are described in section 5.

## Results

Similar to the previous studies [11, 15, 17, 19], we conducted the experiments by five trials of 10-fold cross-validation (CV). We employed the area under curve of receiver operating characteristic (*AUC*) and area under precision-recall curve (*AUPR*) as the evaluation metrics. To valid our prediction method in drug reposition, in completely new drug discovery, and in completely new targets discovery respectively, we conducted the cross-validation under the following three settings:

1. CVP (cross-validation based on the drug-target interaction pairs): Validating for drug reposition. 90% of the drug-target interaction pairs in drug-target interaction network *Y* were randomly selected as training data, and the left 10% of the drug-target interaction pairs were selected as testing data. The CVP setting is used to verify the performance of the prediction method in drug reposition.
2. CVD (cross-validation based on the drugs): Validating for new drug in known targets. 90% of rows (drugs) in *Y* were randomly selected as training data, and the left 10% of rows (drugs) were selected as testing data. The CVD setting is used to verify the performance of the prediction method in new drug discovery.
3. CVT (cross-validation based on the targets): Validating for new target in known drugs. 90% of columns (targets) in *Y* were randomly selected as training data, and the left 10% of columns (targets) were selected as testing data. The CVT setting is used to verify the performance of the prediction method in new target discovery.

We evaluated our method DTIP_MDHN and three existing DTIs prediction methods BLM-NII [10], RLS-WNN [11], NRLMF [17], and DNILMF [19] on the benchmark datasets and the new dataset1. BLM-NII method integrated BLM model with neighbor-based interaction profile to handle the new drugs/targets problem. RLS-WNN is a GIP-based prediction method with a weighted nearest neighbor profile for predicting new drug compounds. NRLMF and DNILMF are two MF-based prediction methods. We implemented algorithm DTIP_MDHN in MATLAB. The experiment was conducted at the high-performance computing center of Guangxi University.^1^

We first evaluated our method DTIP_MDHN and other four methods BLM-NII, RLS-WNN, NRLMF and DNILMF in terms of *AUC* and *AUPR* on benchmark data. To verify the performance of the prediction methods in drug reposition, we conducted the experiment under CVP setting. The experimental results are shown in Table 1. In addition, to verify the performance of prediction methods in new drug/target discovery, we conducted the experiment under CVR and CVC settings. The experimental results are shown in Tables 2 and 3, respectively.

**Table 1:** *AUC* and *AUPR* scores of five methods under CVP setting

Dataset	Method	*AUPR*	*AUC*
Enzyme	BLM-NII	0.7560	0.9792
	RLS-WNN	0.7160	0.9640
	NRLMF	0.8920	0.9870
	DNILMF	0.9220	0.9890
	DTIP_MDHN	**0.9609**	**0.9970**
Ion Channel (IC)	BLM-NII	0.8256	0.9810
	RLS-WNN	0.7170	0.9590
	NRLMF	0.9060	0.9890
	DNILMF	0.9380	0.9900
	DTIP_MDHN	**0.9744**	**0.9976**
GPCR	BLM-NII	0.5420	0.9550
	RLS-WNN	0.5200	0.9440
	NRLMF	0.7490	0.9690
	DNILMF	0.8120	0.9750
	DTIP_MDHN	**0.9540**	**0.9957**
Nuclear Receptor (NR)	BLM-NII	0.6740	0.9153
	RLS-WNN	0.5890	0.9010
	NRLMF	0.7280	0.9500
	DNILMF	0.7510	0.9550
	DTIP_MDHN	**0.8626**	**0.9913**

The best results in each column are in **bold**

**Table 2:** *AUC* and *AUPR* scores of five methods under CVD setting

Dataset	Methods	*AUPR*	*AUC*
Enzyme	BLM-NII	0.2568	0.8230
	RLS-WNN	0.2780	0.8820
	NRLMF	0.3580	0.8710
	DNILMF	0.7960	0.9640
	DTIP_MDHN	**0.8378**	**0.9834**
Ion Channel (IC)	BLM-NII	0.3310	0.7973
	RLS-WNN	0.2580	0.7970
	NRLMF	0.3440	0.8130
	DNILMF	0.8220	0.9610
	DTIP_MDHN	**0.8587**	**0.9845**
GPCR	BLM-NII	0.3250	0.8315
	RLS-WNN	0.2950	0.8910
	NRLMF	0.3640	0.8950
	DNILMF	0.7810	0.9670
	DTIP_MDHN	**0.8487**	**0.9864**
Nuclear Receptor (NR)	BLM-NII	0.4389	0.8010
	RLS-WNN	0.5040	0.8900
	NRLMF	0.5450	0.9000
	DNILMF	0.7760	0.9560
	DTIP_MDHN	**0.8463**	**0.9917**

The best results in each column are in **bold**

**Table 3:** *AUC* and *AUPR* scores of five methods under CVT setting

Dataset	Methods	*AUPR*	*AUC*
Enzyme	BLM-NII	0.7376	0.9190
	RLS-WNN	0.5660	0.9470
	NRLMF	0.8120	0.9660
	DNILMF	**0.8890**	**0.9780**
	DTIP_MDHN	0.8848	0.9463
Ion Channel (IC)	BLM-NII	0.7658	0.9153
	RLS-WNN	0.6960	0.9500
	NRLMF	0.7850	0.9640
	DNILMF	0.8870	0.9700
	DTIP_MDHN	**0.9092**	**0.9705**
GPCR	BLM-NII	0.3532	0.7781
	RLS-WNN	0.5500	0.9260
	NRLMF	0.5560	0.9300
	DNILMF	0.6840	0.9330
	DTIP_MDHN	**0.8865**	**0.9593**
Nuclear Receptor (NR)	BLM-NII	0.4523	0.5430
	RLS-WNN	0.5310	0.9350
	NRLMF	0.4490	0.8510
	DNILMF	0.4830	0.8560
	DTIP_MDHN	**0.8113**	**0.9823**

The best results in each column are in **bold**

We can see from Tables 1, 2 and 3 that compared with other four methods, our method DTIP_MDHN achieves better results for *AUC* metric on the benchmark datasets under CVP and CVD settings, and obtains higher scores on IC, GPCR and NR datasets under CVT setting. For *AUPR* metric, DTIP_MDHN outperforms other four methods on all datasets under CVP and CVD settings, and achieves higher scores on IC, GPCR and NR datasets under CVT setting. The GPCR and NR datasets are sparser than Enzyme and IC datasets, so the prediction accuracy on GPCR and NR datasets is always lower in previous study. In our method DTIP_MDHN, the Jaccard index is introduced to measure the sharing interaction relationship between drugs and targets, the indirect interactions are introduced by global association to solve the data sparsity problem. The experimental result indicates that our method can improve the prediction accuracy, and it is more suitable for predicting DTIs on more sparse datasets such as GPCR and NR under CVT setting.

Constructing the final kernel matrices of drugs and targets is a key step to predict latent DTIs. To compare the effects of different final kernel matrices on the DTIs prediction results, we evaluated our constructed final kernel matrices *KFJD* and *KFJT* with other two final kernel matrices in GIP [9] and DNILMF [19], in terms of *AUC* and *AUPR* on benchmark data. In Table 4, we denote the final kernel matrices of drugs and targets constructed from GIP [9] as *KGD* and *KGT* respectively, the final kernel matrices of drugs and targets constructed from DNILMF [19] as *KFD* and *KFT* respectively*.* Table 4 shows the scores of *AUC* and *AUPR* of DTIP_MDHN using these kernel matrices under CVP setting.

**Table 4:** *AUC* and *AUPR* of DTIP_MDHN using 3 kernel matrices under CVP setting

Dataset	final kernel matrices	*AUPR*	*AUC*
Enzyme	*KGD/KGT*	0.8540	0.9831
	*KFD/KFT*	0.9480	0.9867
	*KFJD/KFJT*	**0.9738**	**0.9995**
Ion Channel (IC)	*KGD/KGT*	0.8735	0.9904
	*KFD/KFT*	0.9482	0.9917
	*KFJD/KFJT*	**0.9700**	**0.9994**
GPCR	*KGD/KGT*	0.8660	0.9812
	*KFD/KFT*	0.9480	0.9973
	*KFJD/KFJT*	**0.9651**	**0.9990**
Nuclear Receptor (NR)	*KGD/KGT*	0.7483	0.9867
	*KFD/KFT*	0.8086	0.9856
	*KFJD/KFJT*	**0.8315**	**0.9988**

The best results in each column are in **bold**

The experimental results in Table 4 indicate that our constructed final kernel matrices of drugs and targets *KFJD* and *KFJT* indeed leads to more accurate predictions in our method DTIP_MDHN than the final kernel matrices in GIP [9] and DNILMF [19].

Next, we evaluated our proposed prediction model with other machine learning models, such as supervised learning models SVM and RF, and Matrix Factorization (MF) model. We extracted our constructed final kernel matrices *KFJD/KFJT* as the features of drug-target pairs, drug-target interaction matrix *Y* as the classification labels for supervised learning prediction models. We used BLM [7] as the SVM-based method, DDR [12] as the RF-based method, and DNILMF [19] as the MF-based method. While the scores of *AUPR* and *AUC* were calculated under CVP setting. Table 5 shows the scores of *AUC* and *AUPR* for four prediction models.

**Table 5:** *AUC* and *AUPR* scores of four prediction models

Dataset	Method	*AUPR*	*AUC*
Enzyme	BLM	0.9552	0.9890
	DDR	0.9457	0.9849
	DNILMF	0.9367	0.9939
	DTIP_MDHN	**0.9609**	**0.9970**
Ion Channel (IC)	BLM	0.8814	0.9891
	DDR	0.9535	0.9914
	DNILMF	0.9499	0.9926
	DTIP_MDHN	**0.9744**	**0.9976**
GPCR	BLM	0.8344	0.9716
	DDR	0.8224	0.9841
	DNILMF	0.8353	0.9804
	DTIP_MDHN	**0.9543**	**0.9957**
Nuclear Receptor (NR)	BLM	0.5949	0.8489
	DDR	0.8302	0.9431
	DNILMF	0.7993	0.9727
	DTIP_MDHN	**0.8626**	**0.9913**

The best results in each column are in **bold**

The experimental results in Table 5 indicate that our proposed prediction model achieves higher scores of *AUC* and *AUPR* than SVM, RF, and MF models in DTIs prediction.

**Fig. 1:**
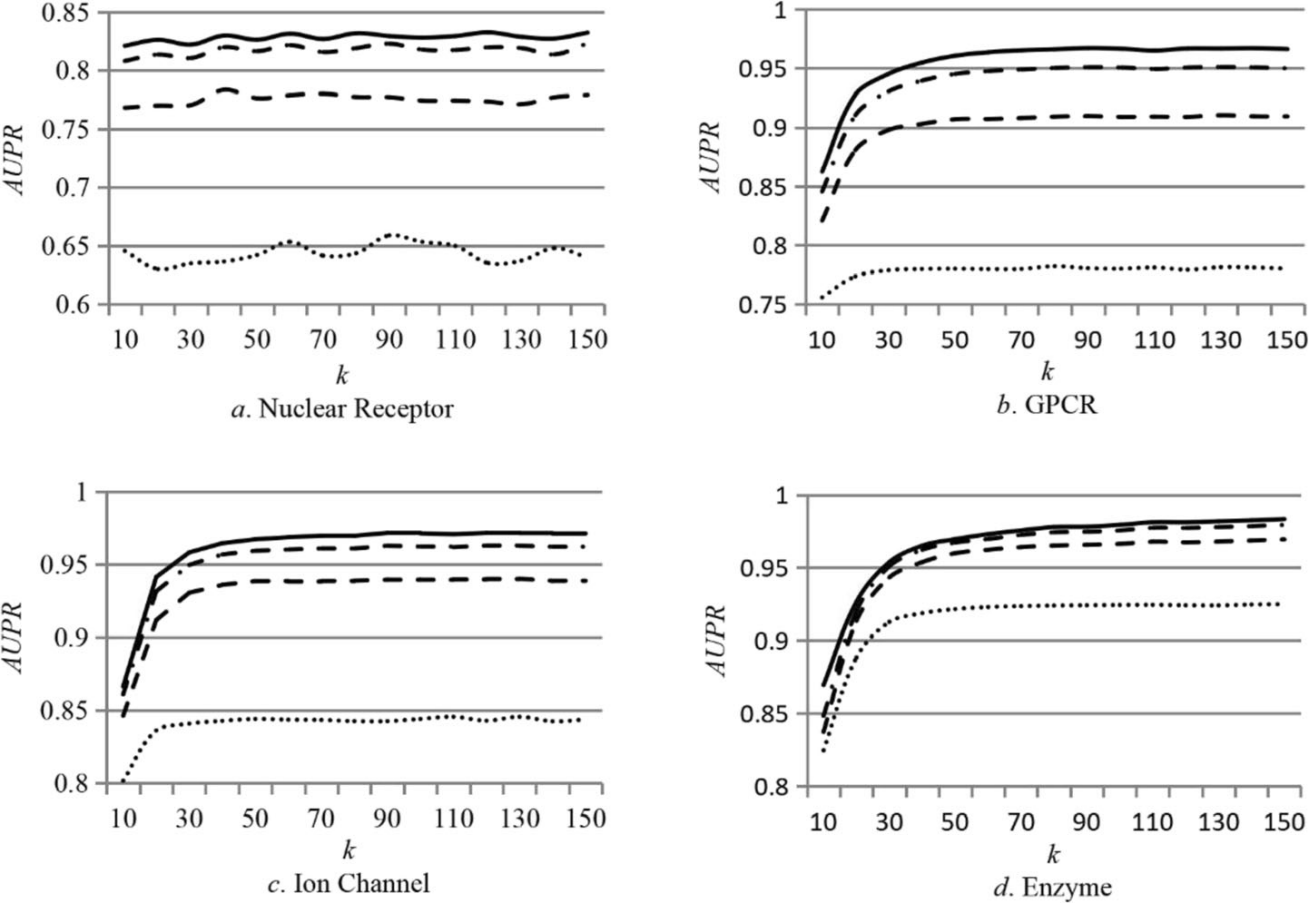
*AUPR* scores of DTIP_MDHN for different values of *noises* and *k* on the benchmark dataset*.* The *k* is set to the value in range [10, 150] as shown in the *x*-axis, and *noise* is set to 0.65, 0.75, 0.85 and 0.95. The solid line denotes the case that *noise* = 0.65, the dashed-dotted line represents the case that *noise* = 0.75, dashed line denotes the case that *noise* = 0.85, and dotted line represents the case that *noise* = 0.95

**Fig. 2:**
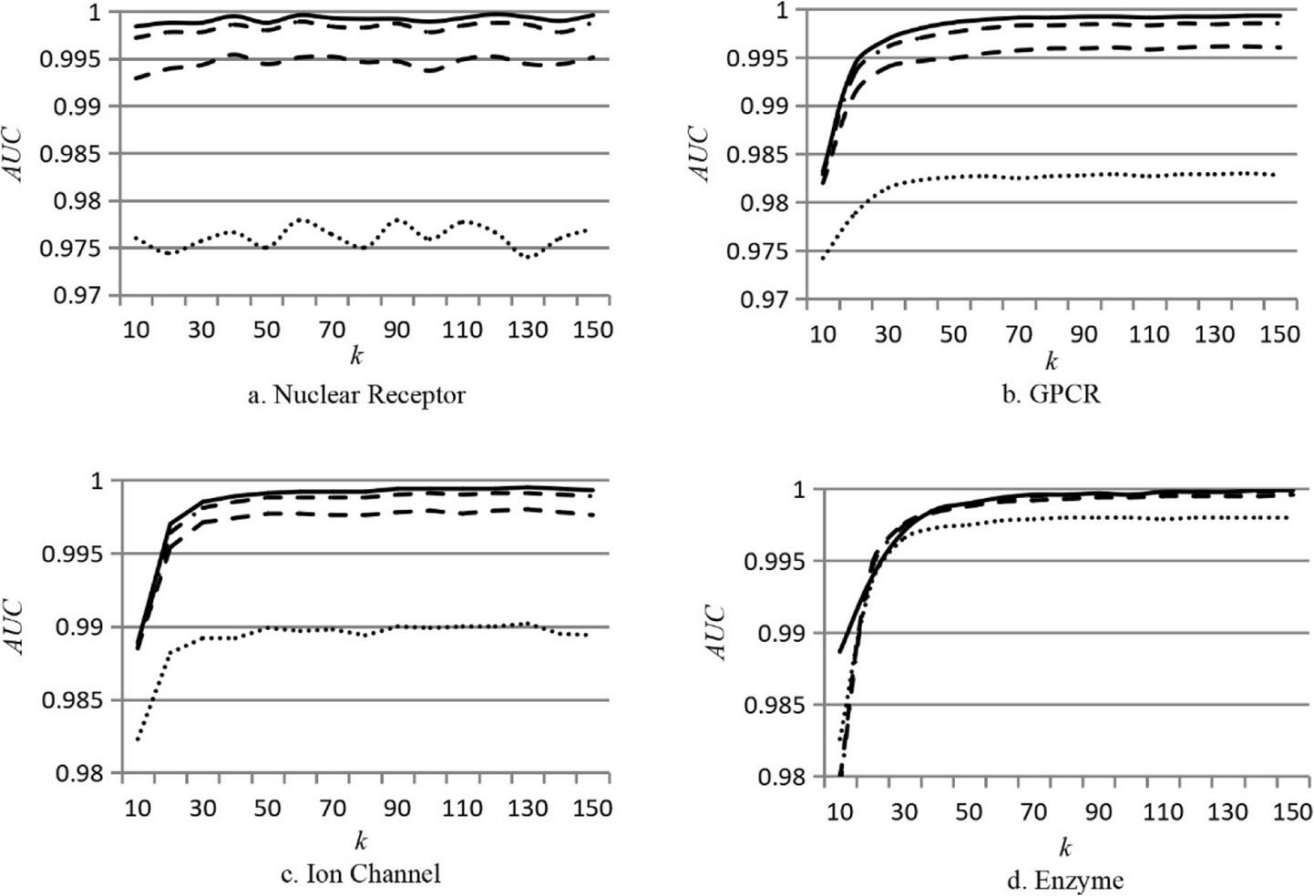
*AUC* scores of DTIP_MDHN for different values of *noises* and *k* on the benchmark dataset*.* The *k* is set to the value in range [10, 150] as shown in the *x*-axis, and *noise* is set to 0.65, 0.75, 0.85 and 0.95. The solid line denotes the case that *noise* = 0.65, the dashed-dotted line represents the case that *noise* = 0.75, dashed line denotes the case that *noise* = 0.85, and dotted line represents the case that *noise* = 0.95

In addition to the final kernel matrices of drugs and targets, there are two key parameters in DTIP_MDHN. One is the noise value (*noise*), and another one is the dimension of latent layer (*k*). We evaluated how the values of *noise* and *k* affect the scores of *AUC* and *AUPR* for DTIP_MDHN on the benchmark datasets respectively. The *noise* is set to 0.65, 0.75, 0.85 and 0.95, and *k* is set to the value in range [10, 150] according to the setting in MDM [31]. The experimental results on four datasets are shown in Figs. 1 and 2 respectively, where the solid line denotes the case that *noise* = 0.65, the dashed-dotted line represents the case that *noise* = 0.75, dashed line denotes the case that *noise* = 0.85, and dotted line represents the case that *noise* = 0.95.

From Figs. 1 and 2 we can see that DTIP_MDHN obtains the highest scores of *AUC* and *AUPR* on the four datasets when *noise* = 0.65. The dimension of latent layer (*k*) indicates the degree of dimensionality reduction in the Auto-Encode (AE). The key information will lose from original data if *k* is too small. The non-critical and redundant information still exists if the value of *k* is too large. In general, the choice of value of *k* depends on the dimension of different datasets. By analyzing the results in Figs. 1 and 2, we set the value of *k* according to the number of drugs for different datasets. Table 6 shows the values of *k* and *noise* on the benchmark datasets.

**Table 6:** Values of *k* and *noise* on the benchmark datasets

Dataset	Number of drugs	*k*	*noise*
Enzyme	445	100	0.65
Ion Channel	210	60	0.65
GPCR	223	60	0.65
Nuclear Receptor	54	20	0.65

To verify the validity of DTIP_MDHN method, we sort the new drug-target interaction pairs predicted by DTIP_MDHN in descending order of the prediction scores and obtain top 5 of the scores for Enzyme, IC, GPCR and NR respectively. If a new drug-target interaction is validated in the current version of KEGG [32], SuperTarget [33], DRUGBANK [34], and ChEMBL [35], the “Validated” item is labeled by “yes”; otherwise it is labeled by “No”. Table 7 shows the top 5 of new drug-target interactions predicted by DTIP_MDHN on the benchmark datasets.

**Table 7:** Top 5 Interactions predicted by DTIP_MDHN on the benchmark datasets

Dataset	KEGG Drug ID	Drug name	KEGG Has ID	Uniport ID	Gene name	Validated
Enzyme	D00542	Halothane	has:1571	P05181	CYP2E1	Yes
	D00139	Methoxsalen	has:1543	P04798	CYP1A1	Yes
	D00437	Nifedipine	has:1559	P11712	CYP2C9	Yes
	D00410	Metyrapone	has:1543	P04798	CYP1A1	Yes
	D00574	Aminoglutethimide	has:1589	P08686	CYP21A2	Yes
Ion Channel	D03365	Nicotine	has:1137	P43681	CHRNA4	Yes
	D00640	Propafenone hydrochloride	has:6336	Q9Y5Y9	SCN10A	Yes
	D02098	Proparacaine hydrochloride	has:8645	O95279	KCNK5	No
	D02356	Verapamil	has:2893	P48058	GRIA4	No
	D00552	Benzocaine	has:6331	Q14524	SCN5A	Yes
GPCR	D00683	Albuterol sulfate	has:153	P08588	ADRB1	Yes
	D02359	Ritodrine	has:153	P08588	ADRB1	No
	D02147	Albuterol	has:153	P08588	ADRB1	Yes
	D01386	Ephedrine hydrochloride	has:153	P08588	ADRB1	Yes
	D00604	Clonidine hydrochloride	has:148	P35348	ADRA1A	No
Nuclear Receptor	D00316	Etretinate	has:6096	Q58EY0	RORβ	No
	D01132	Tazarotene	has:6097	P51449	RORγ	No
	D00182	Norethindrone	has:2099	P03372	ESR1	Yes
	D00348	Isotretinoin	has: 5915	P10826	RARB	Yes
	D01115	Eplerenone	has:2908	P04150	NR3C1	No

As shown in Table 7, the top 5 of new drug-target interactions for Enzyme dataset are validated in current databases. 3 of the top 5 new drug-target interactions for IC and GPCR datasets are validated in current databases respectively. 2 of the top 5 new drug-target interactions for NR dataset are validated in current databases. The statistics for the “Validated” item in Table 10 shows that, the hit rate of prediction for all the four datasets is about 75%. In fact, the NR dataset is the most challenging dataset for DTIs prediction because it is the sparsest dataset among benchmark datasets [6, 8, 18].

We further analyze the no-validated DTI pairs in NR dataset. From Table 7 we can see that the top one of predicted items in NR dataset is a DTI pair between D00316 (Etretinate) and hsa6096 (RORβ). The study in [36] indicated that several retinoids bind to RORβ (hsa6096) to provide a novel pathway for retinoid action. As Etretinate is an aromatic retinoid, a second- generation retinoid, there is a high probability of interaction between Etretinate and RORβ. For the fifth item in NR dataset, D01115 (Eplerenone) is predicted to interact with a Glucocorticoid receptor (hsa2908). Although the interaction between D01115 and hsa2908 has not been found in the current version of KEGG, DRUGBANK, ChEMBL and SuperTarget, an antagonist activity assay confirms this interaction result in PubChem BioAssay ID: AID 761383 from ChEMBL [37].

The benchmark datasets were generated in 2008. Many new interactions are appended to the current version of the KEGG [32], SuperTarget [33], DrugBank [34], and BRENDA [38] nowadays. To enhance the diversity of experimental dataset and inspect the performance of our proposed method on the new database, we used the new dataset1 from KEGG to perform DTIs prediction. Following the category in KEGG, the target proteins can be divided into 8 datasets. In addition to the datasets of Enzyme, IC, GPCR and NR, the 4 new datasets are protein kinase (PK), transporter (TR), cell surface molecule and ligand (CSM), cytokine and cytokine receptor (CR). After deleting the redundant and invalid data, we compiled the new datasets with 11,912 known interactions linking 4495 unique drugs and 959 unique targets. We conducted the experiment to evaluate our method DTIP_MDHN and the newest MF-based method DNILMF. Some drugs may act on two or more different types of targets. For example, Cocaine (D00110) can act on SCN9A (hsa6335) which belongs to Ion channels, and can act on SLC6A2 (hsa6530) which is belongs to Transporters. So, we added a dataset containing all 8 classes of target proteins on KEGG as input in the experiment. This dataset is denoted as “ALL”.

Table 8 shows the *AUC* and *AUPR* scores for two prediction methods on the new datasets1 under CVP setting, in which DNILMF used the optimized parameters (*numLatent* = 90, *c* = 20, *thisAlpha* = 0.7, *λ*_*u*_ = 10, *λ*_*v*_ = 10, *K* = 2) for Enzyme and “ALL” datasets, used the parameters (*numLatent* = 90, *c* = 6, *thisAlpha* = 0.4, λ_u_ = 2, λ_v_ = 2, *K* = 2) for the other datasets, and DTIP_MDHN used the parameters *noise* = 0.65 and the value of *k* in Table 6.

**Table 8:** *AUC* and *AUPR* for DNILMF and DTIP_MDHN on new dataset1 under CVP setting

Dataset	Method	*AUPR*	*AUC*
Enzyme^a^	DNILMF	**0.9245**	**0.9950**
	DTIP_MDHN	0.9071	0.9911
Ion Channel (IC)	DNILMF	0.9921	0.9991
	DTIP_MDHN	**0.9968**	**0.9998**
GPCR	DNILMF	0.9239	0.9935
	DTIP_MDHN	**0.9615**	**0.9963**
Nuclear Receptor (NR)	DNILMF	0.9341	0.9897
	DTIP_MDHN	**0.9610**	**0.9910**
protein kinase	DNILMF	0.8713	0.9875
	DTIP_MDHN	**0.9408**	**0.9959**
transporter	DNILMF	0.8852	0.9907
	DTIP_MDHN	**0.9523**	**0.9978**
cytokine and cytokine receptor	DNILMF	0.8166	0.9827
	DTIP_MDHN	**0.8630**	**0.9842**
cell surface molecule and ligand	DNILMF	0.8557	0.9817
	DTIP_MDHN	**0.9076**	**0.9887**
ALL^a^	DNILMF	0.7578	0.9813
	DTIP_MDHN	**0.9743**	**0.9978**

^a^optimized parameters (*numLatent* = 90, *c* = 20, *thisAlpha* = 0.7, λ_u_ = 10, λ_v_ = 10, *K* = 2) were used in DNILMF

From Table 8, we can see that for the new dataset1 of Enzyme, IC, GPCR, and NR, the scores of *AUC* and *AUPR* computed by DNLMF and DTIP_MDHN are basically the same as that for the benchmark datasets. For the datasets of protein kinase, transporter, cell surface molecule and ligand, cytokine and cytokine receptor, the scores of *AUPR* and *AUC* are mostly about 0.9 and 0.99 respectively. For the “ALL” dataset, the score of *AUPR* is about 0.97 for DTIP_MDHN, and only about 0.63 for DNILMF. The “ALL” dataset is much sparser than any single dataset because the “ALL” dataset treats the interaction information on the above 8 datasets as a whole one. In DNILMF method, only the local neighborhood information is used to measure the similarity between drugs and targets. In DTIP_MDHN method, the global association is exploited to represent the indirect association relationship between drugs and targets, the influence of link sparsity is reduced, and the prediction accuracy is improved.

To verify the availability of our proposed method in binding affinity prediction, we evaluated our method DTIP_MDHN and two existing binding affinity prediction methods Kronecker_rls [9, 20] and SimBoost [22] on the David and Metz datasets, in terms of *AUPR*, *AUC* and concordance index (*CI*) [21, 39]. Kronecker_rls [9, 20] is a DTIs prediction method that first be used to predict binding affinity [21]. SimBoost is a supervised learning model and selects the gradient boosting regression trees to predict continuous binding affinity.

To measure with *AUPR* and *AUC*, the quantitative datasets were binarized by using relatively stringent cut-off thresholds (*K*_*d*_ < 30 *n*M and *K*_*i*_ < 28.18 *n*M) [21]. It means that if *K*_*d*_ *< 30 n*M or *K*_*i*_ < 28.18 *n*M, the affinity value is set to 1, otherwise, the affinity value is set to 0. To measure with the continuous values of *K*_*d*_ and *K*_*i*_*,* the concordance index (*CI*) was used as an evaluation metric [21, 39].

For the continuous values of *K*_*d*_ and *K*_*i*_, we use Pearson Correlation Coefficient (*PCC*) instead of the Jaccard index to calculate the association index kernel because Jaccard kernel matrix works well on binary interaction matrix, while *PCC* is originally developed to measure the relationship between two continuous variables [40] and can be calculated with the function *corrcoef*() in MATLAB. The values of CI calculated by DTIP_MDHN with PCC kernel are denoted as CI_PCC in the following Tables 9, 10, 11, 12, 13 and 14.

**Table 9:** *AUPR*, *AUC*, and *CI* for three binding affinity prediction methods under CVP setting

Dataset	Method	*AUPR*	*AUC*	*CI*	*CI_PCC*
David	Kronecker_rls	0.6586	0.9388	0.8740	–
	SimBoost	0.7580	0.9560	**0.8840**	–
	DTIP_MDHN	**0.7706**	**0.9671**	0.8623	0.8740
Metz	Kronecker_rls	0.5720	0.9340	**0.9340**	–
	SimBoost	0.6290	0.9580	0.8510	–
	DTIP_MDHN	**0.8303**	**0.9960**	0.8702	**0.8812**

The best results in each column are in **bold**. *CI_PCC* is the values of *CI* calculated by DTIP_MDHN with *PCC* kernel

**Table 10:** *AUPR*, *AUC*, and *CI* for two binding affinity prediction methods under CVD setting

Dataset	Method	*AUPR*	*AUC*	*CI*	*CI_PCC*
David	Kronecker_rls	0.2203	0.7055	**0.6981**	–
	DTIP_MDHN	**0.6896**	**0.8892**	0.5489	**0.8014**
Metz	Kronecker_rls	0.4301	0.8596	0.7244	–
	DTIP_MDHN	**0.7648**	**0.9947**	**0.8563**	**0.8043**

The best results in each column are in **bold**. *CI_PCC* is the values of *CI* calculated by DTIP_MDHN with *PCC* kernel

**Table 11:** *AUPR*, *AUC*, and *CI* for two binding affinity prediction methods under CVT setting

Dataset	Method	*AUPR*	*AUC*	*CI*	*CI_PCC*
David	Kronecker_rls	0.5012	0.8912	**0.8037**	–
	DTIP_MDHN	**0.7919**	**0.9701**	0.7875	**0.8293**
Metz	Kronecker_rls	0.2729	0.8355	0.6292	–
	DTIP_MDHN	**0.7473**	**0.9541**	**0.7803**	**0.8045**

The best results in each column are in **bold**. *CI_PCC* is the values of *CI* calculated by DTIP_MDHN with *PCC* kernel

**Table 12:** *AUPR*, *AUC*, and *CI* for different kernels under CVP setting

Kernel	Method	*AUPR*	*AUC*	*CI*	*CI_PCC*
2D	Kronecker_rls	0.6586	0.9388	**0.8740**	–
	DTIP_MDHN	**0.7706**	**0.9471**	0.8623	**0.8740**
3D	Kronecker_rls	0.6642	0.9419	0.8778	–
	DTIP_MDHN	**0.7712**	**0.9474**	**0.8821**	**0.8919**
ECFP4	Kronecker_rls	0.6654	0.9444	0.8793	–
	DTIP_MDHN	**0.7654**	**0.9457**	**0.8856**	**0.9020**

The best results in each column are in **bold**. *CI_PCC* is the values of *CI* calculated by DTIP_MDHN with *PCC* kernel

**Table 13:** *AUPR*, *AUC*, and *CI* for different kernels under CVD setting

Kernel	Method	*AUPR*	*AUC*	*CI*	*CI_PCC*
2D	Kronecker_rls	0.2203	0.7055	**0.6981**	–
	DTIP_MDHN	**0.6896**	**0.8892**	0.5489	**0.8014**
3D	Kronecker_rls	0.3308	0.7700	**0.7441**	–
	DTIP_MDHN	**0.7044**	**0.8970**	0.5547	**0.7470**
ECFP4	Kronecker_rls	0.3117	0.7487	**0.7504**	–
	DTIP_MDHN	**0.7138**	**0.9028**	0.6990	**0.7350**

The best results in each column are in **bold**. *CI_PCC* is the values of *CI* calculated by DTIP_MDHN with *PCC* kernel

**Table 14:** *AUPR*, *AUC*, and *CI* for different kernels under CVT setting

Kernel	Method	*AUPR*	*AUC*	*CI*	*CI_PCC*
2D	Kronecker_rls	0.5010	0.8912	**0.8037**	–
	DTIP_MDHN	**0.7919**	**0.9701**	0.7875	**0.8293**
3D	Kronecker_rls	0.5494	0.9045	**0.8192**	–
	DTIP_MDHN	**0.7903**	**0.9708**	0.7854	**0.8344**
ECFP4	Kronecker_rls	0.6024	0.9201	**0.8408**	–
	DTIP_MDHN	**0.7942**	**0.9700**	0.8313	**0.8354**

The best results in each column are in **bold**. *CI_PCC* is the values of *CI* calculated by DTIP_MDHN with *PCC* kernel

Table 9 shows the scores of *AUPR*, *AUC*, and *CI* for three prediction methods Kronecker_rls [21], SimBoost [22], and our method DTIP_MDHN. Tables 10 and 11 shows the scores of *AUPR*, *AUC*, and *CI* for Kronecker_rls and our method DTIP_MDHN under CVD and CVT setting, respectively. Because the supervised learning methods do not distinguish CVP, CVD and CVT setting, the comparison with SimBoost method was not included in Tables 10 and 11. These results in Tables 9, 10 and 11 were based on the protein target normalized SW sequence similarity and compound drug 2-dimensional structural similarity. We used the default parameters *noise* = 0.65 and *k* = 60 for *AUPR*, *AUC* and *CI,* and used parameters *noise* = 0.95 and *k* = 60 for *CI_PCC* in our method DTIP_MDHN.

From Tables 9, 10 and 11, we can see that our method DTIP_MDHN has higher scores of *AUPR* and *AUC* than Kronecker_rls and SimBoost. We can also see that SimBoost has higher score of C*I* than DTIP_MDHN and Kron_rls on David dataset, and Kron_rls has higher score of *CI* than DTIP_MDHN and SimBooston on Metz dataset under CVP setting in Table 9. Kron_rls has higher score of *CI* than DTIP_MDHN on David dataset, but DTIP_MDHN has higher score of *CI* than Kron_rls on Metz dataset under CVD and CVT setting in Tables 10 and 11 respectively. For DTIP_MDHN, the scores of *CI_PCC* are higher than that of *CI* on David and Metz datasets under CVP and CVT setting and on David dataset under CVD setting. This illustrates that *PCC* kernel matrix achieves higher accuracy than Jaccard kernel matrix in predicting drug-target binding affinity.

Next, we evaluate the prediction accuracy of DTIP_MDHN and Kronecker_rls with different chemical structure and sequence similarity kernels on David dataset in terms of *AUPR*, *AUC* and *CI* under different CV setting. We denote the two-dimensional Tanimoto coefficients similarity kernel matrix as 2D, denote three-dimensional Tanimoto coefficients similarity kernel matrix as 3D, and denote the extended-connectivity fingerprint ECFP4 similarity kernel matrix as ECFP4 in Tables 12, 13 and 14. The value of *CI* calculated by DTIP_MDHN with *PCC* kernel labeled as *CI_PCC*. Kronecker_rls uses the default parameters and DTIP_MDHN uses *noise* = 0.65 and *k* = 60 for *AUPR*, *AUC*, and *CI*, and DTIP_MDHN uses *noise* = 0.95 and *k* = 60 for *CI_PCC.*

From the experimental results shown in Tables 12, 13 and 14, we can see that in terms of *AUPR* and *AUC*, our method DTIP_MDHN outperforms over Kronecker_rls method for all three similarity kernels under all three CV setting. In terms of *CI*, DTIP_MDHN with *PCC* kernel achieves better prediction accuracy than DTIP_MDHN with Jaccard kernel in most cases. This illustrates that *PCC* is more suitable for continuous variable correlation comparison by different similarity kernels. DTIP_MDHN with *PCC* kernel gains better result than Kronecker_rls with 2-dimensional and 3-dimensional drug similarity kernels. Kronecker_rls with ECFP4 fingerprint drug similarity kernels gain better result than DTIP_MDHN with *PCC* kernel.

To inspect our method DTIP_MDHN for large-scale compound-protein interaction (CPIs) prediction, we evaluated DTIP_MDHN with BLM [7] and a deep learning model combining GNN and CNN [26] on new database 2. The new database 2 contains CPIs of *Homo sapiens* retrieved from STITCH database (Version 5.0) [41]. STITCH database contains a comprehensive resource for both known and predicted interactions of compounds and proteins. In order to ensure the accuracy of CPIs data, we extracted the CPIs interactions with combined scores greater than 900 from CPIs of *Homo sapiens* interactions. It means the CPIs interactions that we used in our experiment have the interaction probability greater than 90%. The experimental data contains 13,286 drugs, 5313 targets, and 116,199 interactions. The detailed compound protein interaction information can be referred to Additional file 1. Table 15 shows the values of *AUC* and *AUPR* for DTIP_MDHN, BLM, and GNN&CNN on STITCH dataset under CVP setting.

**Table 15:** *AUC* and *AUPR* on STITCH dataset under CVP setting

Method	*AUPR*	*AUC*
BLM	0.4856	0.9078
DTIP_MDHN	0.8125	**0.9850**
GNN&CNN	**0.8367**	0.9460

The best results in each column are in **bold**

From Table 15, we can see that DTIP_MDHN obtains higher score of *AUC* than BLM and GNN&CNN in large-scale CPIs prediction, which indicates that our method DTIP_MDHN can identify true negatives from the testing data more accurate than BLM and GNN&CNN methods. On the other hand, we can also see that GNN&CNN achieves higher score of *AUPR* than BLM and DTIP_MDHN. This is because GNN&CNN has high sensitivity with reliable negative samples.

## Discussion

In this paper, we propose a novel drug-target interactions (DTIs) prediction method incorporating marginalized denoising model on heterogeneous networks with association index kernel matrix and latent global association. We combine the chemical structure similarity matrix of drugs, the sequence similarity matrix of targets with the GIP kernel matrix and the association index kernel matrix to construct final kernel matrix. We use the association index kernel matrix to enhance the relevance between drugs and targets by calculating the sharing association between drugs and targets. In the building model step, we build a heterogeneous network with drug kernel matrix, target kernel matrix, and existing drug-target interaction network to construct global links for drugs, targets and known drug-target interactions, and further extract latent global associations from the heterogeneous network. The latent global associations between drugs and targets are important to reduce the data sparsity.

The experimental results on benchmark dataset show that our proposed prediction method outperforms the existing binary classification predicting methods and MF-based predicting methods in term of AUC and AUPR. Specifically, for the sparser datasets such as GPCR and NR, the prediction accuracy of our method is increased of 10% ~ 20% than other comparative methods. To compare the effects of different final kernel matrices on the DTIs prediction results, we evaluated our constructed final kernel matrices with other two final kernel matrices in GIP [9] and DNILMF [19]. The experimental results indicate that our constructed final kernel matrices of drugs and targets indeed leads to more accurate predictions than the final kernel matrices in GIP [9] and DNILMF [19]. To evaluate our proposed prediction model with supervised learning models SVM, RF, and Matrix Factorization (MF) model DNILMF, we extracted our constructed final kernel matrices *KFJD/KFJT* as the features of drug-target pairs, drug-target interaction matrix *Y* as the classification labels. The experimental results show that our proposed prediction model achieves higher predictions accuracy than SVM, RF, and DNILMF in DTIs prediction. We also evaluated the key parameters *noise* and *k* within a certain value range to optimize the prediction accuracy. The results show that DTIP_MDHN obtains higher predictions accuracy on the four datasets when *noise* = 0.65, and the optimized value of *k* vary with the number of drugs for different datasets.

To enhance the diversity of experiment data and inspect the performance of our proposed method on the new database, we evaluated our method DTIP_MDHN and the method DNLMF for the 8 classes of target proteins extracted from the current KEGG BRITE database. The experimental results also show that the scores of *AUC* and *AUPR* of DTIP_MDHN are higher than that of DNLMF on the compiled new DTIs database.

The experimental results on Davis and Metz datasets show that our method also can improve the accuracy for predicting drug-target binding affinity. For the continuous values of *K*_*d*_ and *K*_*i*_, we evaluated our method with two association index method, Pearson Correlation Coefficient (PCC) and Jaccard index, respectively. The experimental results show that PCC is more suitable to measure the relationship between two continuous variables, while Jaccard kernel matrix works well on binary interaction matrix.

To inspect our method DTIP_MDHN for large-scale compound-protein interaction (CPIs) prediction, we evaluated DTIP_MDHN with BLM [7] and a deep learning model combining GNN and CNN [26] on our new dataset 2. The experimental dataset contains 13,286 drugs, 5313 targets, and 116,199 interactions. This dataset is much sparser than the benchmark dataset and new dataset 1. The experimental results indicate that our method DTIP_MDHN can identify true negatives from the sparse dataset more accurately than other comparative methods.

## Conclusion

The performance improvement in our method depends on the association index kernel matrix and latent global association. The association index kernel matrix calculates the sharing relationship between drugs and targets. The latent global association addresses the false positive issue caused by network link sparsity. Our method can provide a useful approach to recommend new drug candidates and reposition existing drugs.

The features of a drug-target pair can be characterized more accurately by the biologic physicochemical properties. One future research direction is to use the key biologic physicochemical properties with feature selection method to improve similarity measurement in pharmacology, and extend our method to predict potential interaction relationship in other biologic interaction networks that play a part in pharmacology. Meanwhile, with application of deep learning in the field of drug discovery [23–27], it is also another future research direction for predicting drug-target interactions using deep learning framework on multiple information including biologic physicochemical properties.

## Methods

### Problem description

Given a set of drugs *D* = {*d*_1_,*d*_2_, *…*,*d*_*n*_} and a set of target proteins *T* = {*t*_1_,*t*_2_, …,*t*_*m*_}, a drug similarity matrix *SD* ∈ *ℝ*^*n* × *n*^, a target similarity matrix *ST* ∈ *ℝ*^*m* × *m*^, and a matrix of known interactions *Y* ∈ *ℝ*^*n* × *m*^ between drugs and targets are defined, where *n* is the number of drugs, *m* is the number of target proteins, and each item *Y*_*ij*_ ∈{0, 1}, *i* = 1,2, …,*n*, and *j* = 1,2, …,*m*. If drug *d*_*i*_ has a known interaction with target *t*_*j*_, the value of *Y*_*ij*_ is 1, otherwise is 0, *i* = 1,2, …,*n*, and *j* = 1,2, …,*m*. The goal of drug-target interactions (DTIs) prediction is to recommend new drug- target pairs using above three matrices and other source of information.

The prediction of old drug repositioning is to predict the interaction probability of drug and target when drug and target are known but drug has no known interaction with target. The prediction of new drug/target discovery is to predict the interaction probability of drug and target when drug is newly developed and target is a known protein or a protein target is newly identified and drug is a known compound.

**Fig. 3:**
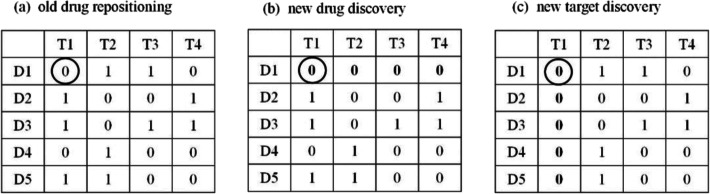
Illustration of the prediction scenarios on old drug repositioning and new drug/target discovery. There are 5 drugs (i.e., D1 - D5) and 4 targets (i.e., T1 - T4). For the D1-T1 interaction pair in a circle, D1 is a known drug, T1 is a known target, and the prediction result on D1-T1 pair is the old drug repositioning in (**a**); D1 is a new drug, T1 is a known target, and the prediction result on D1-T1 pair is the new drug discovery in (**b**); D1 is a known drug, T1 is a new target, and the prediction result on D1-T1 pair is the new target discovery in (**c**)

We illustrated the prediction scenarios on old drug repositioning, new drug/target discovery in Fig. 3. There are 5 drugs (i.e., D1 - D5) and 4 targets (i.e., T1 - T4) in Fig. 3. For the D1-T1 interaction pair in a circle, D1 is a known drug, T1 is a known target, and the prediction result on D1-T1 pair is the old drug repositioning in Fig. 3a; D1 is a new drug, T1 is a known target, and the prediction result on D1-T1 pair is the new drug discovery in Fig. 3b; D1 is a known drug, T1 is a new target, and the prediction result on D1-T1 pair is the new target discovery in Fig. 3c [19].

### Datasets

The benchmark datasets were originally provided by Yamanishi et al. [6]. The datasets are publicly available at http://web.kuicr.kyoto-u.ac.jp/supp/yoshi/drugtarget/. Protein sequences of targets were obtained from the KEGG GENES database [32]. The target similarity matrix is composed of the sequence similarity score between proteins, and it is computed by a normalized version of Smith-Waterman score [42]. Chemical compounds were obtained from the KEGG DRUG and COMPOUND databases [32]. The drug similarity matrix is composed of the chemical structure similarity score between drugs, and it is computed by the SIMCOMP tool [43]. The drug-target interaction matrix is composed of the known drug-target interaction pairs retrieved from databases of KEGG BRITE [32], SuperTarget [33], DrugBank [34], and BRENDA [38]. The benchmark datasets contain four datasets. The first one is enzymes containing 445 drugs and 664 targets. The second one is ion channels (IC) containing 210 drugs and 204 targets. The third one is G-protein coupled receptors (GPCR) containing 223 drugs and 95 targets. And the last one is nuclear receptors (NR) containing 54 drugs and 26 targets. Table 16 lists the statistics for the benchmark datasets [6].

**Table 16:** Statistics for the benchmark datasets [6]

Dataset	Number of drugs	Number of targets	Number of drug-target Interactions	Average degree of drugs	Average degree of targets
Enzymes	445	664	2926	6.57	4.40
IC	210	204	1476	7.02	7.23
GPCR	223	95	635	2.84	6.68
NR	54	26	90	1.66	3.46

In the past decade, an exponential growth of chemical biology data available in the public databases, such as KEGG [32], SuperTarget [33], Drugbank [34], ChEMBL [35], and STITCH [41]. To enhance the diversity of experimental datasets and inspect our proposed predicting method for the latest database, we extracted two new DTIs datasets from KEGG and STITCH respectively.

For new dataset 1, we obtained the classification information of drugs based on the “target-based classification of drugs” in the KEGG BRITE database,^2^ including 8 datasets which are enzymes, ion channels (IC), G protein-coupled receptors (GPCR), nuclear receptors (NR), Cytokines and receptors (CR), Cell surface molecules and ligands (CSM), Protein kinases (PK), and Transporters (TR). The chemical structure similarity matrix of drugs is computed by the SIMCOMP2 tool.^3^ Protein sequence similarity matrix of targets is composed of the scores derived from KEGG SSDB Paralog database. After deleting the redundant and invalid data of drugs, targets, and drug-target interaction pairs, we obtained a total of 8 new datasets containing 11,912 known interactions, 4495 unique drugs, and 959 unique targets. The statistics for new dataset 1 are listed in Table 17. The detailed drug target interaction information can be referred to Additional file 2.

**Table 17:** Statistics for the new dataset 1

Dataset	Number of drugs	Number of targets	Number of drug-target Interactions	Average degree of drugs	Average degree of targets
Enzymes	1178	370	2705	2.30	7.31
IC	462	127	3629	7.85	28.57
GPCR	1582	128	3472	2.19	27.13
NR	422	19	558	1.32	29.37
CR	199	101	283	1.42	2.80
CSM	102	78	234	2.29	3.00
PK	280	95	625	2.23	6.58
TR	270	41	406	1.50	9.9

As shown in Table 17, the amounts of drugs and targets in enzymes, ion channels (IC), G protein-coupled receptors (GPCR), and nuclear receptors (NR) are significantly different from that of the corresponding datasets in benchmark datasets. These datasets are important supplement to benchmark datasets in the experimental verification.

To inspect our proposed method for predicting large-scale compound-protein interactions (CPIs), we retrieved CPIs of *Homo sapiens* from STITCH database (Version 5.0) [41] as new dataset 2.^4^ The compound similarity matrix is derived from the scores of chemical_chemical links in STITCH database.^5^ Similarly, the protein sequence similarity matrix is obtained as new dataset 1. After deleting the redundant and invalid data of drugs, targets, and drug-target interaction pairs, we obtained 5,979,099 interactions between 15,324 unique proteins in *Homo sapiens* and 224,203 unique compounds.

To validate our proposed method for predicting drug-target binding affinity, we selected two kinase datasets from the studies by Davis et al. [44] and Metz et al. [45] respectively. These two datasets are available at http://staff.cs.utu.fi/~aatapa/data/DrugTarget/. In Davis dataset [44], the target similarity matrix is computed by a normalized version of Smith-Waterman score [42]. There are 3 drug similarity matrices in Davis dataset, two-dimensional and three-dimensional Tanimoto coefficients similarity matrices, and the extended-connectivity fingerprint ECFP4 [46] similarity matrix. The drug-target interaction affinity matrix used kinase disassociation constant (*K*_*d*_). There are 68 drugs, 442 targets, and 1527 interactions in Davis dataset.

In Metz dataset [45], the target similarity matrix is computed by a normalized version of Smith-Waterman score [42]. The drug similarity matrix is a two-dimensional Tanimoto coefficients similarity matrix. The drug-target interaction affinity matrix used kinase inhibition constant (*K*_*i*_). There are 1421 drugs, 156 targets, and 3200 interactions in Metz dataset.

The statistics for these two kinase datasets are listed in Table 18.

**Table 18:** Statistics for the kinase datasets

Dataset	Number of drugs	Number of targets	Number of drug-target Interactions	Average degree of drugs	Average degree of targets
David	68	442	1527	22.46	3.45
Ketz	1421	156	3200	2.25	20.51

### Method

We propose a new method to learn drug kernel matrix and target kernel matrix. We integrate drug kernel matrix, target kernel matrix, and drug-target interaction network to build a heterogeneous network. We apply the marginalized denoising model on heterogeneous network to improve the accuracy of drug-target interaction prediction. Our proposed prediction method consists of the following four steps:

- Step 1: Calculate drug kernel matrix *KFJD* by combining drug similarity matrix *SD*, Gaussian interaction profile kernel matrix for drugs *KGD*, and association index kernel matrix for drugs *KJD*, where *KGD* and *KJD* are constructed from drug-target interaction network *Y*.
- Step 2: Calculate target kernel matrix *KFJT* by combining target similarity matrix *ST*, Gaussian interaction profile kernel matrix for targets *KGT*, and association index kernel matrix for targets *KJT*, where *KGT* and *KJT* are constructed from *Y′* which is the transpose of drug-target interaction network *Y*.
- Step 3: Construct a heterogeneous network *M* by drug kernel matrix *KFJD*, target kernel matrix *KFJT*, and drug-target interaction network *Y*.
- Step 4: Create a marginalized denoising model (MDM) on the constructed heterogeneous network *M* with local and global associations between nodes (targets and drugs) to predict latent drug-target interaction pairs.

**Fig. 4:**
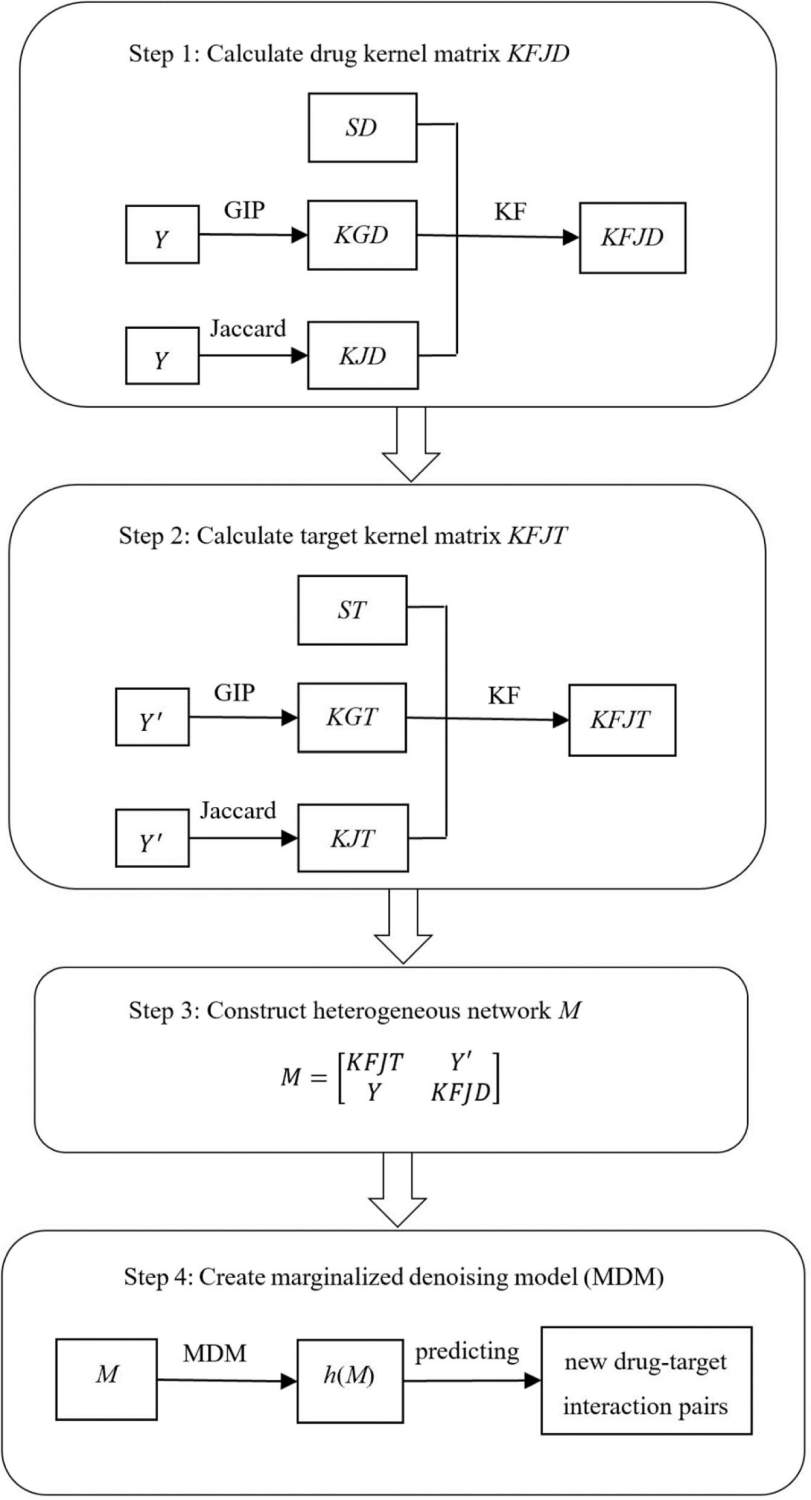
Procedure of our proposed predicting method. Drug kernel matrix *KFJD* was calculated by combining drug similarity matrix *SD*, GIP kernel matrix for drugs *KGD*, and association index kernel matrix for drugs *KJD*, where *KGD* and *KJD* are constructed from drug-target interaction network *Y* (seen in step 1). target kernel matrix *KFJT* was calculated by combining target similarity matrix *ST*, GIP kernel matrix for targets *KGT*, and association index kernel matrix for targets *KJT*, where *KGT* and *KJT* are constructed from *Y*′ which is the transpose of *Y* (seen in step 2). Next, a heterogeneous network *M* was constructed by drug kernel matrix *KFJD*, target kernel matrix *KFJT*, and drug-target interaction network *Y* (seen in step 3). Finally, a marginalized denoising model (MDM) was created on the heterogeneous network *M* with local and global associations between nodes (targets and drugs) to predict latent drug-target interaction pairs (seen in step 4)

The procedure of our proposed prediction method is shown in Fig. 4.

#### Constructing final kernel matrix

The final kernel matrix combines different kernels with drug similarity matrix and target similarity matrix for potential DTIs prediction. Based on kernel fusion [18, 19], we calculate drug kernel matrix by combining drug similarity matrix with GIP kernel matrix and Jaccard kernel matrix, and calculate target kernel matrix by combining target protein similarity matrix with GIP kernel matrix and Jaccard kernel matrix.

The final drug kernel matrix *KFJD* and final target kernel matrix *KFJT* are calculated according to the following steps.

Firstly, GIP kernel matrix for drugs *KGD* and GIP kernel matrix for targets *KGT* are calculated respectively [9]:

1 $$ {\displaystyle \begin{array}{l}{KGD}_{d_i,\kern0.75em {d}_j}=\mathit{\exp}\left(-{\gamma}_d{\left\Vert {y}_{d_i}-{y}_{d_j}\right\Vert}^2\right),1\le i,j\le n\\ {}\ {KGT}_{t_i,\kern0.75em {t}_j}=\mathit{\exp}\left(-{\gamma}_t{\left\Vert {y}_{t_i}-{y}_{t_j}\right\Vert}^2\right),1\le i,j\le m\end{array}} $$

where ${y}_{d_i}$ and ${y}_{d_j}$ are interaction profiles of drugs *d*_*i*_ and *d*_*j*_ respectively, which are represented by binary vectors encoding presence or absence of interaction with every target in interaction matrix *Y*. Similarly, ${y}_{t_i}$ and ${y}_{t_j}$ are interaction profiles of targets *t*_*i*_ and *t*_*j*_ respectively, which are represented by binary vectors encoding presence or absence of interaction with every drug in interaction matrix *Y*. Parameters *γ*_*d*_ and *γ*_*t*_ are used to control kernel bandwidth and are defined as follows [9]:

2 $$ {\displaystyle \begin{array}{l}{\gamma}_d=1/\left(\frac{1}{n_d}{\sum}_{i=1}^{n_d}{\left|{y}_{d_i}\right|}^2\right)\\ {}{\gamma}_t=1/\left(\frac{1}{n_t}{\sum}_{j=1}^{n_t}{\left|{y}_{t_j}\right|}^2\right)\end{array}} $$

Secondly, Jaccard profile kernel matrix for drugs and Jaccard profile kernel matrix for targets are calculated respectively.

Jaccard index [47] is commonly used in association index. Compared with cosine, Pearson correlation coefficient, and other association index, Jaccard index is more suitable for binary data with high sparsity, and Jaccard index is used to measure the degree of sharing association between two nodes in biological interaction network [40]. Hence, we use Jaccard index to construct an association index kernel matrix between drugs and an association index kernel matrix between targets in DTIs network respectively. Next, we discuss how to calculate Jaccard kernel matrix for drug *KJD* and Jaccard kernel matrix for target *KJT*.

The value of Jaccard index kernel for drugs *d*_*i*_ and *d*_*j*_ in DTIs network, ${KJD}_{d_i,{d}_j}$, is calculated as follows [40]:

3 $$ {KJD}_{d_i,{d}_j}=\frac{D_{11}}{D_{01}+{D}_{10}+{D}_{11}},1\le i,j\le n $$

where *D*_01_, *D*_10_, and *D*_11_ are three parameters to measure the sharing relationship between *d*_*i*_ and *d*_*j*._*D*_01_ is total number of targets when the value of *Y*(*d*_*i*_, *t*_*k*_) is 0 and the value of *Y* (*d*_*j*_, *t*_*k*_) is 1, *D*_10_ denotes total number of targets when the value of *Y*(*d*_*i*_, *t*_*k*_) is 1 and the value of *Y*(*d*_*j*_, *t*_*k*_) is 0, *D*_11_ represents total number of targets when the value of *Y*(*d*_*i*_, *t*_*k*_) is 1 and the value of *Y*(*d*_*j*_, *t*_*k*_) also is 1, where *Y* is the target-drug interaction matrix, and *t*_*k*_ is a target contained in *Y*, *i, j* = 1,2,…,*n*, and *k* = 1,2,…,*m*.

Similarly, the value of Jaccard index kernel for targets *t*_*i*_ and *t*_*j*_ in DTIs network, ${KJT}_{t_i,{t}_j}$, is computed as follows [40]:

4 $$ {KJT}_{t_i,{t}_j}=\frac{T_{11}}{T_{01}+{T}_{10}+{T}_{11}},1\le i,j\le m $$

where *T*_01_, *T*_10_, and *T*_11_ are three parameters to measure the sharing relationship between *t*_*i*_ and *t*_*j*._*T*_01_ is total number of drugs when the value of *Y*(*d*_*k*_, *t*_*i*_) is 0 and the value of *Y*(*d*_*k*_, *t*_*j*_) is 1, *T*_10_ denotes total number of drugs when the value of *Y*(*d*_*k*_, *t*_*i*_) is 1 and the value of *Y*(*d*_*k*_, *t*_*j*_) is 0, *T*_11_ represents total number of drugs when the value of *Y*(*d*_*k*_, *t*_*i*_) is 1 and the value of *Y*(*d*_*k*_, *t*_*j*_) also is 1, where *Y* is the target-drug interaction matrix, and *d*_*k*_ is a drug contained in *Y*, *i, j* = 1,2,…,*m*, and *k* = 1,2,…,*n*.

Thirdly, based on the nonlinear kernel fusion technique [17, 18], the final drug kernel matrix *KFJD* is calculated according to three matrices *SD*, *KGD* and *KJD*, and the final target kernel matrix *KFJT* is calculated according to three matrices *ST, KGT* and *KJT*.

The calculation for *KFJD* is described as follows.

The three kernel matrices *SD*, *KGD*, and *KJD* are first normalized according to Hao’s method [18]. The normalized matrices are denoted by *PD*1, *PD*2, and *PD*3 respectively [18]:

5 $$ {\displaystyle \begin{array}{l} PD1\left({d}_i,{d}_j\right)=\left\{\begin{array}{c}\frac{SD\left({d}_i,{d}_j\right)}{2{\sum}_{k\ne i} SD\left({d}_i,{d}_k\right)},j\ne i\\ {}\kern2.25em 1/2\kern1.75em ,j=i\end{array}\right.,1\le i,j\le n\\ {} PD2\left({d}_i,{d}_j\right)=\left\{\begin{array}{c}\frac{KGD\left({d}_i,{d}_j\right)}{2{\sum}_{k\ne i} KGD\left({d}_i,{d}_k\right)},j\ne i\\ {}\kern2.25em 1/2\kern1.75em ,j=i\end{array}\right.,1\le i,j\le n\\ {} PD3\left({d}_i,{d}_j\right)=\left\{\begin{array}{c}\frac{KJD\left({d}_i,{d}_j\right)}{2{\sum}_{k\ne i} KJD\left({d}_i,{d}_k\right)},j\ne i\\ {}\kern2.25em 1/2\kern1.75em ,j=i\end{array}\right.,1\le i,j\le n\end{array}} $$

Then, we apply the *k* nearest neighbors (*k*NN) algorithm to compute local similarity matrices *LD*1, *LD*2, and *LD*3 for *PD*1, *PD*2, and *PD*3 respectively [18]:

6 $$ {\displaystyle \begin{array}{l} LD1\left({d}_i,{d}_j\right)=\left\{\begin{array}{c}\frac{PD1\left({d}_i,{d}_j\right)}{\sum_{d_k\in {N}_i} PD1\left({d}_i,{d}_k\right)},{d}_j\in {N}_i\\ {}\kern3.5em 0\kern2.5em ,{d}_j\notin {N}_i\end{array}\right.,1\le i,j\le n\\ {} LD2\left({d}_i,{d}_j\right)=\left\{\begin{array}{c}\frac{PD2\left({d}_i,{d}_j\right)}{\sum_{d_k\in {N}_i} PD2\left({d}_i,{d}_k\right)},{d}_j\in {N}_i\\ {}\kern3.5em 0\kern2.25em ,\kern0.5em {d}_j\notin {N}_i\end{array}\right.,1\le i,j\le n\\ {} LD3\left({d}_i,{d}_j\right)=\left\{\begin{array}{c}\frac{PD3\left({d}_i,{d}_j\right)}{\sum_{d_k\in {N}_i} PD3\left({d}_i,{d}_k\right)},{d}_j\in {N}_i\\ {}\kern3.5em 0\kern2.5em ,\kern0.5em {d}_j\notin {N}_i\end{array}\right.,1\le i,j\le n\end{array}} $$

where *N*_*i*_ denotes the *k* nearest neighbors of drug *d*_*i*_, *i* = 1,2,…,*n*. In formula (6), the similarity between any two non-nearest neighbors is set to zero to reduce the influence on prediction results from the non-nearest drug-target interaction pairs.

The key step of fusion operation is an iterative calculation [18]:

7 $$ {\displaystyle \begin{array}{l}\ {PD}_{t+1}^1= LD1\times \frac{\left(\ {PD}_t^2+{PD}_t^3\right)}{2}\times LD{1}^{\prime}\\ {}{PD}_{t+1}^2= LD2\times \frac{\left(\ {PD}_t^1+{PD}_t^3\right)}{2}\times LD{2}^{\prime}\\ {}{PD}_{t+1}^3= LD3\times \frac{\left(\ {PD}_t^1+{PD}_t^2\right)}{2}\times LD{3}^{\prime}\end{array}} $$

where ${PD}_{t+1}^1$, ${PD}_{t+1}^2$, and ${PD}_{t+1}^3$ are the results of *PD*1, *PD*2, and *PD*3 after *t* iterations respectively, and *LD*1^′^, *LD*2^′^, and *LD*3^′^ are the transposes of *LD*1, *LD*2, and *LD*3 respectively.

During each iteration, the values of ${PD}_{t+1}^1$, ${PD}_{t+1}^2$, and ${PD}_{t+1}^3$ are further updated by ${PD}_{t+1}^1=\left({PD}_{t+1}^1+{PD_{t+1}^1}^{\prime}\right)/2+I$, ${PD}_{t+1}^2=\left({PD}_{t+1}^2+{PD_{t+1}^2}^{\prime}\right)/2+I$, and ${PD}_{t+1}^3=\left({PD}_{t+1}^3+{PD_{t+1}^3}^{\prime}\right)/2+I$ respectively, where *I* is an identity matrix.

After *t* iterations, the final drug kernel matrix *KFJD* can be obtained by [17]:

8 $$ KFJD=\left({PD}_t^1+{PD}_t^2+{PD}_t^3\right)/3 $$

Similarly, the final target kernel matrix *KFJT* can be obtained as follows:

9 $$ KFJT=\left({PT}_t^1+{PT}_t^2+{PT}_t^3\right)/3 $$

More detailed description about the kernel fusion can be seen in [18, 19, 48].

#### Marginalized denoising model

Our method treats DTIs prediction problem as network link prediction problem. We use Marginalized denoising model (MDM) [31] on heterogeneous network composed of the final drug and target kernel matrices and the known drug-target interaction matrix to predict potential DTIs. Marginalized denoising model [31] is inspired by the idea of marginalized denoising auto- encoders [49].

Auto-Encoder (AE) is a type of artificial neural networks, which is used to learn efficient data coding in an unsupervised manner [50, 51]. The AE encodes original input dataset *x* with weight *w* into latent representation *h* and decodes *h* into output *y*, where *h* = *f*(*x*) and *y* = *g*(*h*). The AE is trained to minimize reconstruction error $\mathcal{L}\left(x,g\Big(f(x)\right)\Big)$ to guarantee that output *y* closely matches original data *x*. The AE is widely used to extract features and reduce dimensionality. The AE can also be used to learn new features.

Denoising Auto-Encoder (DAE) [52] transforms original input dataset *x* into partially corrupted input $\overset{\sim }{x}$ and trains $\overset{\sim }{x}$ to recover undistorted original input *x*. To train an auto-encoder to denoised data, a preliminary stochastic mapping $x\to \overset{\sim }{x}$ is performed to corrupt the data, and $\overset{\sim }{x}$ with weight *w* is used as an input for normal auto-encoder. The loss function of DAE is represented by $\mathcal{L}\left(x,g\Big(f\left(\overset{\sim }{x}\right)\right)\Big)$ instead of $\mathcal{L}\left(x,g\Big(f(x)\right)\Big)$. The corrupted input $\overset{\sim }{x}$ can be constructed by randomly setting original input *x* to zero with given probability *p*, where 0 < *p* < 1. The original noises in original input dataset *x* are removed during the corrupting process. To a certain extent, the training data are close to the testing data after the training data are denoised, and the robustness of weight *w* is enforced after training.

Marginalized denoising auto-encoder (mDA) [49] is a variant of DAE. The mDA is used to solve the problem with high computational cost of the DAE. “Marginalized” means that the loss function $\mathcal{L}\left(x,g\Big(f\left(\overset{\sim }{x}\right)\right)\Big)$ is approximated by the expected value $\mathbbm{E}{\left\Vert \mathcal{L}\left(x,g\Big(f\left(\overset{\sim }{x}\right)\right)\Big)\right\Vert}_{p\left(\overset{\sim }{x}|x\right)}$ of loss function with conditional distribution $p\left(\overset{\sim }{x}|x\right)$ based on the weak law of large number [53].

#### Our prediction method

The latent drug-target interactions are impacted by the existing drug-target interaction pairs in the drug- target interaction network. The probability of predicting drug-target interactions may also be influenced by the matrix of similarities between drugs and the matrix of similarities between targets [19].

We treat the drug-target interactions (DTIs) prediction problem as network link prediction problem. To improve the prediction accuracy, we propose a DTIs prediction method using marginalized denoising model on heterogeneous network. The heterogeneous network can be represented by matrix $$ M=\left[\begin{array}{cc} KFJT& Y^{\prime}\\ {}Y& KFJD\end{array}\right] $$ of size (*m + n*) × (*m + n*), where *KFJT* ∈ *ℝ*^*m* × *m*^ is the target kernel matrix, *KFJD* ∈ *ℝ*^*n* × *n*^ is the drug kernel matrix, *Y* ∈*ℝ*^*n* × *m*^ is the drug-target interaction network, *Y*′ is the transpose of *Y*, *m* is the number of targets, and *n* is the number of drugs.

To generate the training data, we inject random noise to original input matrix *M* to construct the corrupted matrix $\overset{\sim }{M}$. The set of corrupted matrices $\overset{\sim }{\mathcal{M}}=\left\{{\overset{\sim }{M}}_1,{\overset{\sim }{M}}_2,\dots, {\overset{\sim }{M}}_c\right\}$ is the training data. Then, we train the mapping function $h\left(\overset{\sim }{M}\right)$ such that the final output M* closely matches the original matrix *M.* That is to minimize the loss function $\mathcal{L}\left(h\left(\overset{\sim }{M}\right)\right)$:

10 $$ \mathcal{L}\left(h\left(\overset{\sim }{M}\right)\right)={\sum}_{\overset{\sim }{M}\in \overset{\sim }{\mathcal{M}}}{\left\Vert M-h\left(\overset{\sim }{M}\right)\right\Vert}_{\mathrm{F}}^2 $$

11 $$ {M}^{\ast }=h\left(\overset{\sim }{M}\right)={\sum}_{l=1}^{m+n}{L}_{il}{\overset{\sim }{M}}_{lj}+{\sum}_{l=1}^{m+n}{\sum}_{k=1}^{m+n}{\overset{\sim }{M}}_{il}{G}_{lk}{\overset{\sim }{M}}_{jk}+{b}_i,1\le i,j\le m+n $$

where the mapping function $h\left(\overset{\sim }{M}\right)$ consists of the latent local and global associations between any two drug or target nodes in *M*, ${\left\Vert .\right\Vert}_{\mathrm{F}}^2$ denotes the Frobenius norm of matrix, $\overset{\sim }{M}$ s in corrupted matrices set $\overset{\sim }{\mathcal{M}}$ are constructed by randomly setting the value of elements in *M* to zero with given probability *p*, where 0 < *p* < 1, *b*_*i*_ is a bias value, *L* is local association weighted matrix, ${\sum}_{l=1}^{m+n}{L}_{il}{\overset{\sim }{M}}_{lj}$ is latent local interaction between nodes *i* and *j* via node *l*, *G* is global association weighted matrix and ${\sum}_{l=1}^{m+n}{\sum}_{k=1}^{m+n}{\overset{\sim }{M}}_{il}{G}_{lk}{\overset{\sim }{M}}_{jk}$ is latent global association between nodes *i* and *j* via nodes *l* and *k*, 1 ≤ *i*, *j* ≤ *m* + *n*.

**Fig. 5:**
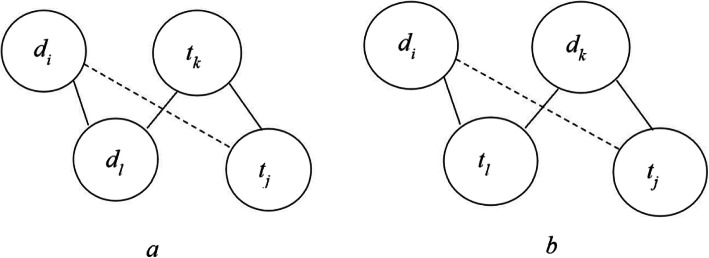
Illustration of latent global association. The solid line shows the existing association, and the dashed line shows latent global association. As shown in (**a**), if drug *d*_*i*_ is highly similar to drug *d*_*l*_, *d*_*l*_ has an interaction with target *t*_*k*_, and *t*_*k*_ is highly similar to target *t*_*j*_, then *d*_*i*_ has an interaction with *t*_*j*_ with high probability. As shown in (**b**), if both drugs *d*_*i*_ and *d*_*k*_ have an interaction with target *t*_*l*_, and *d*_*k*_ has an interaction with target *t*_*j*_, then *d*_*i*_ has an interaction with *t*_*j*_ with high probability

We illustrate an example of latent global association in Fig. 5. The solid line shows the existing association, and the dashed line shows latent global association.

As shown in Fig. 5a, if drug *d*_*i*_ is highly similar to drug *d*_*l*_, *d*_*l*_ has an interaction with target *t*_*k*_, and *t*_*k*_ is highly similar to target *t*_*j*_, then *d*_*i*_ has an interaction with *t*_*j*_ with high probability. We can also see from Fig. 5b that, if both drugs *d*_*i*_ and *d*_*k*_ have an interaction with target *t*_*l*_, and *d*_*k*_ has an interaction with target *t*_*j*_, then *d*_*i*_ has an interaction with *t*_*j*_ with high probability. The latent global association represents the weighted value of indirect drug-target interaction. The iterative training with latent local and global associations will obtain a more precise drug-target interaction prediction result M*.

To prevent loss function $\mathcal{L}(h)$ from overfitting and enhance the learning performance, we construct a new objective function $\mathcal{L}\left(L,G,b\right)$ by Tikhonov regularization terms:

12 $$ \mathcal{L}\left(L,G,b\right)={\sum}_{\overset{\sim }{M}\in \overset{\sim }{\mathcal{M}}}{\left\Vert M-L\overset{\sim }{M}-\overset{\sim }{M}G{\overset{\sim }{M}}^T-{\left(b\ast {1}_n\right)}^T\right\Vert}_{\mathrm{F}}^2+\frac{\lambda_1}{2}\left({\left\Vert L\right\Vert}_F^2\right)+\frac{\lambda_2}{2}\left({\left\Vert G\right\Vert}_F^2\right) $$

where *L* and *G* represent latent local and global association weighted matrices respectively, *b* is a bias vector, 1_*n*_ denotes an all-one column vector of size *n*, and *λ*_1_ and *λ*_2_ are the regularization coefficients. Tikhonov regularization is used to ensure the smoothness of fitting curves of *L* and *G* [54]_._

In the denoising auto-encoder, the more the training data used, the more accurate the prediction results are. Ideally, we use infinite training data to compute weight matrices *L* and *G*. However, when the size of set $\overset{\sim }{\mathcal{M}}$ is increased, the computation cost becomes more expensive. According to the weak law of large number [53], when the size of set $\overset{\sim }{\mathcal{M}}$ becomes very large, we can rewrite the sum part of formula (12) into the expectation form as follows:

13 $$ \mathcal{L}\left(L,G,b\right)={\mathbbm{E}}_{p\left(\overset{\sim }{M}|M\right)}\left[{\left\Vert M-L\overset{\sim }{M}-\overset{\sim }{M}G{\overset{\sim }{M}}^T-b{1}_n^T\right\Vert}_{\mathrm{F}}^2\right]+\frac{\lambda_1}{2}\left({\left\Vert L\right\Vert}_{\mathrm{F}}^2\right)+\frac{\lambda_2}{2}\left({\left\Vert G\right\Vert}_{\mathrm{F}}^2\right) $$

where $p\left(\overset{\sim }{M}|M\right)$ is a conditional distribution, and the expectation is with respect to the random variable $\overset{\sim }{M}$.

To apply formula (13) in large data matrix, low rank approximation is used [31]. Formula (13) is rewritten with respect to *L = UU*^*T*^ and *G = VV*^*T*^ as follows:

14 $$ \mathcal{L}\left(U,V,b\right)=0.5\ast tr\left({M}^{\mathrm{T}}M\right)- tr\left({U}^T\ast \overset{\sim }{M}{M}^T\ast U+{V}^T\ast {\overset{\sim }{M}}^T{M}^T\overset{\sim }{M}\ast V+{M}^Tb{1}_{\mathrm{n}}^{\mathrm{T}}\right)+0.5\ast tr\left({U}^T\ast U{U}^T\overset{\sim }{M}{\overset{\sim }{M}}^T\ast U+{V}^T\ast {\overset{\sim }{M}}^T\overset{\sim }{M}V{V}^T{\overset{\sim }{M}}^T\overset{\sim }{M}\ast V+{b}^T\ast b{1}_{\mathrm{n}}^{\mathrm{T}}\right)+ tr\left({U}^T\ast \overset{\sim }{M}V{V}^T{\overset{\sim }{M}}^T{\overset{\sim }{M}}^T\ast U+{U}^T\ast b{1}_{\begin{array}{c}\mathrm{n}\end{array}}^{\mathrm{T}}{\overset{\sim }{M}}^T\ast U+{V}^T\ast {\overset{\sim }{M}}^Tb{1}_{\begin{array}{c}n\end{array}}^T\overset{\sim }{M}\ast V\right)\kern3.5em +0.5\ast \mathrm{tr}\left({U}^T\ast {\lambda}_1\mathrm{I}\ast U\right)+0.5\ast tr\left({V}^T\ast {\lambda}_2I\ast V\ \right) $$

where *U*, *V* ∈*ℝ*^(*m* + *n*) × *k*^, *k* is the dimension of latent variables *U* and *V*, *tr*(*) represents the trace of matrices, and *I* is the identity matrix.

To minimize the norm function $\mathcal{L}\left(U,V,b\right)$, the partial gradient of formula (14) is calculated with respect to *U*, *V* and *b* as follows:

15 $$ \frac{\partial \mathcal{L}}{\kern0.5em \partial U}=\mathbbm{E}\left[\left(U{U}^T\overset{\sim }{M}{\overset{\sim }{M}}^T+\overset{\sim }{M}{\overset{\sim }{M}}^TU{U}^T+\overset{\sim }{M}V{V}^T{\overset{\sim }{M}}^T{\overset{\sim }{M}}^T+\overset{\sim }{M}\overset{\sim }{M}V{V}^T{\overset{\sim }{M}}^T+b{1}_{\mathrm{n}}^{\mathrm{T}}{\overset{\sim }{M}}^T+\overset{\sim }{M}{b}^T-M{\overset{\sim }{M}}^T-\overset{\sim }{M}{M}^T\right)\right]U+{\lambda}_1U $$

16 $$ \frac{\partial \mathcal{L}}{\partial\ V}=\mathbbm{E}\left[{\overset{\sim }{M}}^T\left(U{U}^T\overset{\sim }{M}+{\overset{\sim }{M}}^TU{U}^T+\overset{\sim }{M}V{V}^T{\overset{\sim }{M}}^T+\overset{\sim }{M}V{V}^T{\overset{\sim }{M}}^T+b{1}_n^T+{b}^T-M-{M}^T\right)\overset{\sim }{M}\right]V+{\lambda}_2V $$

17 $$ \frac{\partial \mathcal{L}}{\partial b}=\mathbbm{E}\left[\left(U{U}^T\overset{\sim }{M}+\overset{\sim }{M}V{V}^T{\overset{\sim }{M}}^T+b{1}_n^T-M\right){1}_n\right] $$

Given *q* as the residual probability for $\overset{\sim }{M}$, *q* = 1-*p*, we label a constant matrix containing no $\overset{\sim }{M}$ as *C*, and calculate the gradients for different terms of $\overset{\sim }{M}$. For a term containing only one $\overset{\sim }{M}$, $\mathbbm{E}\left[C\overset{\sim }{M}\right]=C\mathbbm{E}\left[\overset{\sim }{M}\right]= qCM$. For a term containing two $\overset{\sim }{M}\mathrm{s}$, we need to analyze the cases that the two $\overset{\sim }{M}$ s are the same or not, e.g., if the two $\overset{\sim }{M}$ s are the same, $\mathbbm{E}\left[{\overset{\sim }{M}}^TC\overset{\sim }{M}\right]={q}^2{M}^TC\ M$, otherwise $\mathbbm{E}\left[{\overset{\sim }{M}}^TC\overset{\sim }{M}\right]=q\left(1-q\right)\operatorname{diag}\left({M}^T\ast \operatorname{diag}(C)\right)$. The term containing two $\overset{\sim }{M}$ s, $\mathbbm{E}\left[{\overset{\sim }{M}}^TC\overset{\sim }{M}\right]$, is given in formula (18) [31]:

18 $$ \mathbbm{E}\left[{\overset{\sim }{M}}^TC\overset{\sim }{M}\right]={q}^2{M}^TC\ M+q\left(1-q\right)\mathit{\operatorname{diag}}\left({M}^T\ast \mathit{\operatorname{diag}}(C)\right) $$

For the term containing three or more $\overset{\sim }{M}$ s, we need to analyze the cases that all the $\overset{\sim }{M}$ s are the same or any two $\overset{\sim }{M}$ s are the same or all the $\overset{\sim }{M}$ s are different. The term containing three $\overset{\sim }{M}$ s, $\mathbbm{E}\left[\overset{\sim }{M}C{\overset{\sim }{M}}^T{\overset{\sim }{M}}^T\right]$, is given as follows [31]:

19 $$ \mathbbm{E}\left[\overset{\sim }{M}C{\overset{\sim }{M}}^T{\overset{\sim }{M}}^T\right]={q}^3 MC\ {M}^T{M}^T+{q}^2\left(1-q\right)\left(\mathit{\operatorname{diag}}\left(M\ast \mathit{\operatorname{diag}}(C)\right){M}^T+M\ast C\ast \mathit{\operatorname{diag}}\left.\left(\mathit{\operatorname{diag}}(M)\right)+\mathit{\operatorname{diag}}\left(\mathrm{M}\right)\ast sum\Big(C{}^{\circ}M,2\right)\right)+q\left(1-2q\right)\left(1-q\right)\mathit{\operatorname{diag}}\Big(\mathit{\operatorname{diag}}(M){}^{\circ}\left(\mathit{\operatorname{diag}}(C)\right) $$

where the function *diag*(*) outputs the diagonal elements of a matrix, the operator ° denotes the Hadamard product (element-wise product), and the function *sum*(∗, 2) outputs the sum by rows of a matrix.

We use the L-BFGS (Limited-memory BFGS) [55] to optimize the objective functions with respect to latent variables *U*, *V,* and *b*. The L-BFGS [55] is an optimization algorithm in the family of quasi-Newton methods. The Newton’s method is an iterative optimization using Taylor’s second-order expansion. The Newton’s method finds extrema for loss function by computing Hessian matrix. It is too expensive to compute Hessian matrix for every iteration. The L-BFGS algorithm optimizes the calculation of Newton’s method and simplifies the calculation of Hessian matrix. L-BFGS has the feature of fast convergence and no storage of Hessian matrix.

Finally, we calculate the final matrix *M*^∗^ = *UU*^′^*M* + *MVV*^′^*M*^′^ + *b*, and compute the evaluation metrics *AUC* (area under curve of receiver operating characteristic) and *AUPR* (area under precision-recall curve) by comparing *M* with M^*^.

Based on the above steps, we propose a drug-target interaction prediction algorithm using marginalized denoising model on heterogeneous network called DTIP_MDHN, in which its input files *SDFile*, *STFile* and *YFile* are derived from http://web.kuicr.kyoto-u.ac.jp/supp/yoshi/ drugtarget/, *noise* is the noise value, and *k* is the dimension of latent layer. Algorithm DTIP_MDHN is described in algorithm 1.

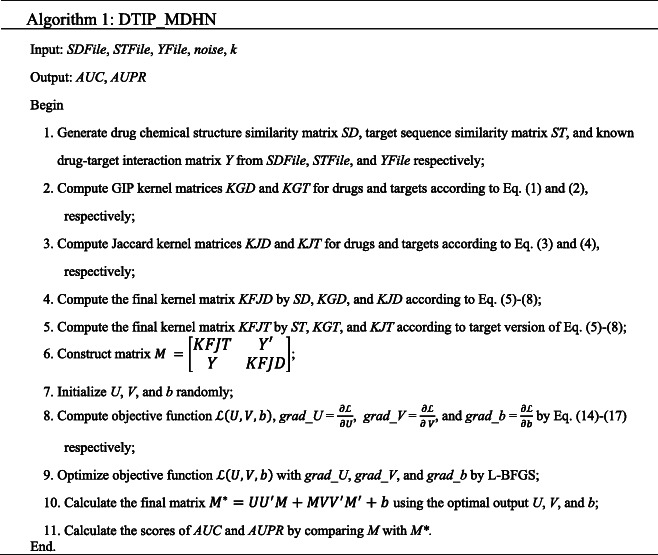



Our method DTIP_MDHN can obtain more accurate prediction than other existing methods because it introduces Jaccard index kernel matrix to measure the sharing interaction relationship between drugs and targets, and uses both local and global associations to reduce the sparsity of DTIs network.

## Supplementary information

**Additional file 1. **Compound-Protein Interaction pairs selected for large-scale CPIs prediction. This file records the detailed compound-protein interaction pairs selected for large-scale CPIs prediction. These data were extracted from STITCH database (Version 5.0) with combined scores greater than 900 in *Homo sapiens* and contains 13,286 drugs, 5313 targets, and 116,199 interactions. In this file, compounds are derived from PubChem with the prefix “CID”, proteins are derived from Ensembl with the prefix “ENSP”, scores are the combined scores in STITCH database. The scores indicate the interaction probability of corresponding compound protein interaction pair. All detailed information about these interactions can be found in STITCH database.

**Additional file 2.** Drug-Target Interaction pairs in the new Dataset 1. This file records the detailed drug-target interaction pairs on enzymes, ion channels, GPCRs, nuclear receptors, Cytokines and receptors, Cell surface molecules and ligands, Protein kinases, and Transporters of the new Dataset 1. The new database 1 was extracted from KEGG database and contains 4495 drugs, 959 targets, and 11,912 known interactions.

## Data Availability

The benchmark datasets were publicly available at http://web.kuicr.kyoto-u.ac.jp/supp/yoshi/drugtarget/. Algorithm DTI_MDHN is implemented in MATLAB. The software suite of our method is available at 10.6084/m9.figshare.11980161.

## Abbreviations

DTI: Drug-target interaction
AUC: Area under curve of receiver operating characteristic
AUPR: Area under precision-recall curve
DTIP_MDHN: A drug-target interaction prediction method using marginalized denoising model on heterogeneous network
GIP: Gaussian interaction profile
MF: Matrix factorization
MDM: Marginalized denoising model
CPI: Compound-protein interaction
PPI: Protein-protein interaction
ECFP: The extended-connectivity fingerprint
DAE: Denoising Auto-Encoder
CI: The concordance index

## Footnotes

1 http://hpc.gxu.edu.cn

2 https://www.kegg.jp/kegg-bin/get_htext?br08310.keg

3 https://www.genome.jp/tools/simcomp2/

4 http://stitch.embl.de/download/protein_chemical.links.v5.0/9606.protein_chemical.links.v5.0.tsv.gz

5 http://stitch.embl.de/download/chemical_chemical.links.v5.0.tsv.gz
