# Supplementary material for: Drug-target interactions prediction using marginalized denoising model on heterogeneous networks
Source: BMC Bioinformatics. 2020 Jul 23;21:330. doi: 10.1186/s12859-020-03662-8 (PMC7653902; doi:10.1186/s12859-020-03662-8)
Supplement: Supplementary file 2 — Additional file 2. Drug-Target Interaction pairs in the new Dataset 1. This file records the detailed drug-target interaction pairs on enzymes, ion channels, GPCRs, nuclear receptors, Cytokines and receptors, Cell surface molecules and ligands, Protein kinases, and Transporters of the new Dataset 1. The new database 1 was extracted from KEGG database and contains 4495 drugs, 959 targets, and 11,912 known interactions. [file 12859_2020_3662_MOESM2_ESM.docx]

Additional file 2: Drug-target interaction pairs in the new dataset 1

The detailed drug-target interaction on enzymes, ion channels, GPCRs, nuclear receptors, Cytokines and receptors, Cell surface molecules and ligands, Protein kinases, and Transporters in the new Dataset 1. The data were shown in Table 1-8 respectively. It contains 11912 drug-target interaction pairs in total, of which 2705 for enzymes, 3629 for ion channels, 3472 for GPCRs, 558 for nuclear receptors,283 for Cytokines and receptors, 234 for Cell surface molecules and ligands, 625 for Protein kinases, and 406 for Transporters.

In each table, the column named drug is the KEGG drug id, the column named target is the KEGG hsa id. All detailed information about these interactions can be found in KEGG database.

Table1. The overall of drug-target interactions for enzymes class on new dataset 1

| **drug** | **target** | **drug** | **target** | **drug** | **target** | **drug** | **target** |
| --- | --- | --- | --- | --- | --- | --- | --- |
| hsa43 | D00043 | hsa5143 | D02218 | hsa200895 | D06239 | hsa5159 | D09635 |
| hsa590 | D00043 | hsa5144 | D02218 | hsa2322 | D06272 | hsa1432 | D09639 |
| hsa7299 | D00073 | hsa5150 | D02218 | hsa2324 | D06272 | hsa5600 | D09639 |
| hsa3643 | D00085 | hsa5151 | D02218 | hsa3791 | D06272 | hsa5603 | D09639 |
| hsa290 | D00087 | hsa5153 | D02218 | hsa3815 | D06272 | hsa6300 | D09639 |
| hsa5530 | D00107 | hsa8622 | D02218 | hsa5159 | D06272 | hsa5689 | D09640 |
| hsa5532 | D00107 | hsa8654 | D02229 | hsa5894 | D06272 | hsa5690 | D09640 |
| hsa5533 | D00107 | hsa3156 | D02258 | hsa5979 | D06272 | hsa5693 | D09640 |
| hsa5742 | D00109 | hsa3290 | D02264 | hsa673 | D06272 | hsa3065 | D09641 |
| hsa5743 | D00109 | hsa5742 | D02290 | hsa5243 | D06277 | hsa3066 | D09641 |
| hsa476 | D00112 | hsa5743 | D02290 | hsa26279 | D06283 | hsa8841 | D09641 |
| hsa477 | D00112 | hsa43 | D02292 | hsa30814 | D06283 | hsa7153 | D09654 |
| hsa478 | D00112 | hsa7153 | D02321 | hsa391013 | D06283 | hsa7155 | D09654 |
| hsa480 | D00112 | hsa7155 | D02321 | hsa50487 | D06283 | hsa1956 | D09660 |
| hsa5742 | D00118 | hsa231 | D02323 | hsa5319 | D06283 | hsa25 | D09664 |
| hsa5743 | D00118 | hsa231 | D02328 | hsa5320 | D06283 | hsa6714 | D09664 |
| hsa5742 | D00120 | hsa5742 | D02341 | hsa5322 | D06283 | hsa25 | D09665 |
| hsa5743 | D00120 | hsa5743 | D02341 | hsa64600 | D06283 | hsa6714 | D09665 |
| hsa217 | D00123 | hsa5742 | D02350 | hsa81579 | D06283 | hsa5604 | D09666 |
| hsa219 | D00123 | hsa5743 | D02350 | hsa8399 | D06283 | hsa5605 | D09666 |
| hsa224 | D00123 | hsa5742 | D02355 | hsa84647 | D06283 | hsa240 | D09667 |
| hsa7153 | D00125 | hsa5743 | D02355 | hsa2321 | D06285 | hsa92 | D09670 |
| hsa7155 | D00125 | hsa7498 | D02365 | hsa2324 | D06285 | hsa5580 | D09671 |
| hsa5742 | D00126 | hsa6240 | D02368 | hsa3791 | D06285 | hsa5581 | D09671 |
| hsa5743 | D00126 | hsa6716 | D02398 | hsa3815 | D06285 | hsa5583 | D09671 |
| hsa5742 | D00127 | hsa43 | D02418 | hsa5159 | D06285 | hsa5588 | D09671 |
| hsa5743 | D00127 | hsa11238 | D02441 | hsa43 | D06288 | hsa79001 | D09676 |
| hsa5743 | D00130 | hsa23632 | D02441 | hsa43 | D06296 | hsa79001 | D09677 |
| hsa217 | D00131 | hsa377677 | D02441 | hsa10013 | D06320 | hsa10135 | D09678 |
| hsa219 | D00131 | hsa759 | D02441 | hsa3065 | D06320 | hsa7150 | D09679 |
| hsa224 | D00131 | hsa760 | D02441 | hsa3066 | D06320 | hsa3480 | D09680 |
| hsa5742 | D00132 | hsa761 | D02441 | hsa8841 | D06320 | hsa2321 | D09683 |
| hsa5743 | D00132 | hsa762 | D02441 | hsa43 | D06365 | hsa2324 | D09683 |
| hsa5742 | D00141 | hsa763 | D02441 | hsa2224 | D06378 | hsa3791 | D09683 |
| hsa5743 | D00141 | hsa765 | D02441 | hsa2224 | D06379 | hsa1956 | D09689 |
| hsa1719 | D00142 | hsa766 | D02441 | hsa7153 | D06386 | hsa2064 | D09689 |
| hsa200895 | D00142 | hsa767 | D02441 | hsa7155 | D06386 | hsa2066 | D09689 |
| hsa5742 | D00151 | hsa768 | D02441 | hsa5243 | D06387 | hsa1956 | D09690 |
| hsa5743 | D00151 | hsa771 | D02441 | hsa6716 | D06397 | hsa2064 | D09690 |
| hsa1312 | D00152 | hsa1588 | D02451 | hsa2321 | D06402 | hsa2066 | D09690 |
| hsa1588 | D00153 | hsa43 | D02558 | hsa2322 | D06402 | hsa10038 | D09692 |
| hsa100 | D00155 | hsa590 | D02558 | hsa2324 | D06402 | hsa10039 | D09692 |
| hsa3290 | D00156 | hsa4128 | D02559 | hsa3791 | D06402 | hsa142 | D09692 |
| hsa5742 | D00158 | hsa4128 | D02560 | hsa3815 | D06402 | hsa143 | D09692 |
| hsa5743 | D00158 | hsa4128 | D02561 | hsa5156 | D06402 | hsa1586 | D09701 |
| hsa5340 | D00160 | hsa4129 | D02562 | hsa5159 | D06402 | hsa2147 | D09707 |
| hsa3251 | D00161 | hsa4128 | D02563 | hsa5979 | D06402 | hsa2159 | D09710 |
| hsa5742 | D00169 | hsa4128 | D02564 | hsa231 | D06403 | hsa5580 | D09718 |
| hsa5743 | D00169 | hsa4129 | D02564 | hsa1956 | D06407 | hsa5581 | D09718 |
| hsa2147 | D00181 | hsa43 | D02565 | hsa3791 | D06407 | hsa5583 | D09718 |
| hsa5530 | D00184 | hsa4128 | D02579 | hsa5979 | D06407 | hsa5588 | D09718 |
| hsa5532 | D00184 | hsa4129 | D02579 | hsa2147 | D06410 | hsa1956 | D09724 |
| hsa5533 | D00184 | hsa4128 | D02580 | hsa5972 | D06412 | hsa2064 | D09724 |
| hsa43 | D00196 | hsa4129 | D02580 | hsa25 | D06413 | hsa2066 | D09724 |
| hsa2548 | D00216 | hsa4128 | D02581 | hsa3815 | D06413 | hsa3643 | D09727 |
| hsa2595 | D00216 | hsa476 | D02587 | hsa5156 | D06413 | hsa25 | D09728 |
| hsa279 | D00216 | hsa477 | D02587 | hsa5159 | D06413 | hsa6714 | D09728 |
| hsa280 | D00216 | hsa478 | D02587 | hsa1969 | D06414 | hsa10038 | D09730 |
| hsa8972 | D00216 | hsa480 | D02587 | hsa25 | D06414 | hsa10039 | D09730 |
| hsa5742 | D00217 | hsa495 | D02593 | hsa2534 | D06414 | hsa142 | D09730 |
| hsa5743 | D00217 | hsa496 | D02593 | hsa3815 | D06414 | hsa238 | D09731 |
| hsa11238 | D00218 | hsa5141 | D02655 | hsa3932 | D06414 | hsa6098 | D09731 |
| hsa23632 | D00218 | hsa5142 | D02655 | hsa5159 | D06414 | hsa1956 | D09733 |
| hsa377677 | D00218 | hsa5143 | D02655 | hsa6714 | D06414 | hsa2064 | D09733 |
| hsa759 | D00218 | hsa5144 | D02655 | hsa7525 | D06414 | hsa2066 | D09733 |
| hsa760 | D00218 | hsa1436 | D02664 | hsa1719 | D06503 | hsa3480 | D09746 |
| hsa761 | D00218 | hsa7153 | D02697 | hsa200895 | D06503 | hsa43 | D09750 |
| hsa762 | D00218 | hsa7155 | D02697 | hsa2618 | D06503 | hsa1803 | D09753 |
| hsa763 | D00218 | hsa7153 | D02698 | hsa7298 | D06503 | hsa1803 | D09756 |
| hsa765 | D00218 | hsa7155 | D02698 | hsa1803 | D06553 | hsa5742 | D09760 |
| hsa766 | D00218 | hsa5743 | D02709 | hsa5141 | D06575 | hsa5743 | D09760 |
| hsa767 | D00218 | hsa2475 | D02714 | hsa5142 | D06575 | hsa2548 | D09779 |
| hsa768 | D00218 | hsa43 | D02729 | hsa5143 | D06575 | hsa2595 | D09779 |
| hsa771 | D00218 | hsa8654 | D02731 | hsa5144 | D06575 | hsa8972 | D09779 |
| hsa7498 | D00224 | hsa124975 | D02755 | hsa1803 | D06578 | hsa1803 | D09780 |
| hsa10846 | D00227 | hsa2678 | D02755 | hsa4860 | D06596 | hsa3716 | D09783 |
| hsa27115 | D00227 | hsa2686 | D02755 | hsa5321 | D06600 | hsa3717 | D09783 |
| hsa50940 | D00227 | hsa2687 | D02755 | hsa5742 | D06606 | hsa3718 | D09783 |
| hsa5136 | D00227 | hsa7150 | D02756 | hsa5743 | D06606 | hsa7297 | D09783 |
| hsa5137 | D00227 | hsa7153 | D02756 | hsa4047 | D06609 | hsa7498 | D09786 |
| hsa5138 | D00227 | hsa7155 | D02756 | hsa2064 | D06610 | hsa2159 | D09817 |
| hsa5139 | D00227 | hsa231 | D02835 | hsa2984 | D06612 | hsa10846 | D09822 |
| hsa5140 | D00227 | hsa1017 | D02880 | hsa1513 | D06634 | hsa27115 | D09822 |
| hsa5141 | D00227 | hsa1019 | D02880 | hsa10013 | D06637 | hsa50940 | D09822 |
| hsa5142 | D00227 | hsa1021 | D02880 | hsa10014 | D06637 | hsa5136 | D09822 |
| hsa5143 | D00227 | hsa1022 | D02880 | hsa3065 | D06637 | hsa5137 | D09822 |
| hsa5144 | D00227 | hsa983 | D02880 | hsa3066 | D06637 | hsa5138 | D09822 |
| hsa5150 | D00227 | hsa7498 | D02896 | hsa51564 | D06637 | hsa5139 | D09822 |
| hsa5151 | D00227 | hsa5139 | D02933 | hsa55869 | D06637 | hsa5140 | D09822 |
| hsa5153 | D00227 | hsa5140 | D02933 | hsa79885 | D06637 | hsa5141 | D09822 |
| hsa8622 | D00227 | hsa4881 | D02935 | hsa83933 | D06637 | hsa5142 | D09822 |
| hsa5139 | D00231 | hsa4882 | D02935 | hsa8841 | D06637 | hsa5143 | D09822 |
| hsa5140 | D00231 | hsa3815 | D02937 | hsa9734 | D06637 | hsa5144 | D09822 |
| hsa1636 | D00251 | hsa5578 | D02970 | hsa9759 | D06637 | hsa5150 | D09822 |
| hsa476 | D00297 | hsa5141 | D02985 | hsa1803 | D06645 | hsa5151 | D09822 |
| hsa477 | D00297 | hsa5142 | D02985 | hsa2475 | D06669 | hsa5153 | D09822 |
| hsa478 | D00297 | hsa5143 | D02985 | hsa5321 | D06674 | hsa8622 | D09822 |
| hsa480 | D00297 | hsa5144 | D02985 | hsa2321 | D06678 | hsa43 | D09835 |
| hsa476 | D00298 | hsa3717 | D03003 | hsa2324 | D06678 | hsa495 | D09837 |
| hsa477 | D00298 | hsa3718 | D03003 | hsa3791 | D06678 | hsa496 | D09837 |
| hsa478 | D00298 | hsa3717 | D03004 | hsa3815 | D06678 | hsa5139 | D09843 |
| hsa480 | D00298 | hsa3718 | D03004 | hsa5156 | D06678 | hsa5140 | D09843 |
| hsa5141 | D00302 | hsa240 | D03010 | hsa5159 | D06678 | hsa476 | D09847 |
| hsa8654 | D00302 | hsa6646 | D03012 | hsa5979 | D06678 | hsa477 | D09847 |
| hsa18 | D00304 | hsa8435 | D03012 | hsa2147 | D06880 | hsa478 | D09847 |
| hsa2571 | D00304 | hsa1786 | D03021 | hsa476 | D06881 | hsa480 | D09847 |
| hsa2572 | D00304 | hsa1788 | D03021 | hsa477 | D06881 | hsa240 | D09850 |
| hsa7915 | D00304 | hsa1789 | D03021 | hsa478 | D06881 | hsa3815 | D09864 |
| hsa5743 | D00315 | hsa1595 | D03025 | hsa480 | D06881 | hsa4233 | D09864 |
| hsa6716 | D00321 | hsa4313 | D03061 | hsa1728 | D07064 | hsa558 | D09864 |
| hsa5742 | D00330 | hsa4318 | D03061 | hsa79001 | D07064 | hsa3815 | D09865 |
| hsa5743 | D00330 | hsa5159 | D03065 | hsa1803 | D07080 | hsa4233 | D09865 |
| hsa6241 | D00341 | hsa10846 | D03075 | hsa2147 | D07082 | hsa558 | D09865 |
| hsa495 | D00355 | hsa27115 | D03075 | hsa2159 | D07086 | hsa1017 | D09868 |
| hsa496 | D00355 | hsa50940 | D03075 | hsa5141 | D07088 | hsa1019 | D09868 |
| hsa3156 | D00359 | hsa5136 | D03075 | hsa5142 | D07088 | hsa1021 | D09868 |
| hsa1636 | D00362 | hsa5137 | D03075 | hsa5143 | D07088 | hsa1022 | D09868 |
| hsa10846 | D00371 | hsa5138 | D03075 | hsa5144 | D07088 | hsa983 | D09868 |
| hsa27115 | D00371 | hsa5139 | D03075 | hsa10846 | D07089 | hsa5663 | D09869 |
| hsa50940 | D00371 | hsa5140 | D03075 | hsa27115 | D07089 | hsa1551 | D09881 |
| hsa5136 | D00371 | hsa5141 | D03075 | hsa50940 | D07089 | hsa1576 | D09881 |
| hsa5137 | D00371 | hsa5142 | D03075 | hsa5136 | D07089 | hsa1577 | D09881 |
| hsa5138 | D00371 | hsa5143 | D03075 | hsa5137 | D07089 | hsa64816 | D09881 |
| hsa5139 | D00371 | hsa5144 | D03075 | hsa5138 | D07089 | hsa1956 | D09883 |
| hsa5140 | D00371 | hsa5150 | D03075 | hsa5139 | D07089 | hsa2064 | D09883 |
| hsa5141 | D00371 | hsa5151 | D03075 | hsa5140 | D07089 | hsa2066 | D09883 |
| hsa5142 | D00371 | hsa5153 | D03075 | hsa5141 | D07089 | hsa7357 | D09893 |
| hsa5143 | D00371 | hsa8622 | D03075 | hsa5142 | D07089 | hsa7357 | D09894 |
| hsa5144 | D00371 | hsa1636 | D03077 | hsa5143 | D07089 | hsa3480 | D09908 |
| hsa5150 | D00371 | hsa240 | D03080 | hsa5144 | D07089 | hsa142 | D09913 |
| hsa5151 | D00371 | hsa1644 | D03082 | hsa5150 | D07089 | hsa412 | D09915 |
| hsa5153 | D00371 | hsa6715 | D03107 | hsa5151 | D07089 | hsa1080 | D09916 |
| hsa8622 | D00371 | hsa6093 | D03115 | hsa5153 | D07089 | hsa43 | D09917 |
| hsa1636 | D00383 | hsa9475 | D03115 | hsa8622 | D07089 | hsa43 | D09918 |
| hsa18 | D00399 | hsa5243 | D03128 | hsa7153 | D07100 | hsa2260 | D09919 |
| hsa2571 | D00399 | hsa2147 | D03136 | hsa7155 | D07100 | hsa2261 | D09919 |
| hsa2572 | D00399 | hsa5693 | D03150 | hsa2224 | D07119 | hsa2263 | D09919 |
| hsa7915 | D00399 | hsa1723 | D03154 | hsa2224 | D07123 | hsa2264 | D09919 |
| hsa7173 | D00401 | hsa3067 | D03158 | hsa1373 | D07130 | hsa2321 | D09919 |
| hsa1584 | D00410 | hsa5742 | D03163 | hsa79001 | D07131 | hsa2324 | D09919 |
| hsa240 | D00414 | hsa5743 | D03163 | hsa79001 | D07134 | hsa3791 | D09919 |
| hsa5139 | D00417 | hsa5972 | D03208 | hsa79001 | D07135 | hsa5979 | D09919 |
| hsa5140 | D00417 | hsa2159 | D03213 | hsa79001 | D07136 | hsa2260 | D09920 |
| hsa1586 | D00420 | hsa8654 | D03217 | hsa79001 | D07137 | hsa2261 | D09920 |
| hsa1636 | D00421 | hsa2321 | D03218 | hsa6916 | D07140 | hsa2263 | D09920 |
| hsa5742 | D00425 | hsa2324 | D03218 | hsa5742 | D07141 | hsa2264 | D09920 |
| hsa5743 | D00425 | hsa3791 | D03218 | hsa5743 | D07141 | hsa2321 | D09920 |
| hsa5742 | D00428 | hsa7153 | D03220 | hsa5742 | D07142 | hsa2324 | D09920 |
| hsa5743 | D00428 | hsa7155 | D03220 | hsa2147 | D07143 | hsa3791 | D09920 |
| hsa3156 | D00434 | hsa7150 | D03225 | hsa2147 | D07144 | hsa5979 | D09920 |
| hsa495 | D00455 | hsa3643 | D03230 | hsa476 | D07147 | hsa2159 | D09923 |
| hsa496 | D00455 | hsa2224 | D03234 | hsa477 | D07147 | hsa3480 | D09925 |
| hsa1636 | D00459 | hsa4128 | D03239 | hsa478 | D07147 | hsa2321 | D09926 |
| hsa5742 | D00463 | hsa4129 | D03239 | hsa480 | D07147 | hsa834 | D09938 |
| hsa5743 | D00463 | hsa43 | D03239 | hsa7173 | D07231 | hsa5156 | D09939 |
| hsa43 | D00469 | hsa590 | D03239 | hsa3098 | D07257 | hsa4233 | D09941 |
| hsa43 | D00487 | hsa1312 | D03241 | hsa3099 | D07257 | hsa3791 | D09945 |
| hsa4128 | D00505 | hsa4128 | D03248 | hsa3101 | D07257 | hsa2984 | D09948 |
| hsa4129 | D00505 | hsa3643 | D03250 | hsa80201 | D07257 | hsa25 | D09950 |
| hsa5742 | D00510 | hsa25 | D03252 | hsa262 | D07258 | hsa25 | D09951 |
| hsa5743 | D00510 | hsa6714 | D03252 | hsa1588 | D07260 | hsa2322 | D09955 |
| hsa2977 | D00517 | hsa5742 | D03254 | hsa5742 | D07267 | hsa2322 | D09956 |
| hsa2982 | D00517 | hsa5743 | D03254 | hsa5743 | D07267 | hsa3716 | D09959 |
| hsa2983 | D00517 | hsa2064 | D03257 | hsa5742 | D07268 | hsa3717 | D09959 |
| hsa11238 | D00518 | hsa8654 | D03260 | hsa5743 | D07268 | hsa3716 | D09960 |
| hsa23632 | D00518 | hsa3480 | D03297 | hsa5742 | D07269 | hsa3717 | D09960 |
| hsa377677 | D00518 | hsa4881 | D03328 | hsa5743 | D07269 | hsa3716 | D09970 |
| hsa759 | D00518 | hsa6476 | D03342 | hsa2224 | D07281 | hsa3717 | D09970 |
| hsa760 | D00518 | hsa3990 | D03343 | hsa5742 | D07294 | hsa3718 | D09970 |
| hsa761 | D00518 | hsa4023 | D03343 | hsa5743 | D07294 | hsa7297 | D09970 |
| hsa762 | D00518 | hsa4311 | D03349 | hsa4128 | D07337 | hsa121278 | D09973 |
| hsa763 | D00518 | hsa1956 | D03350 | hsa4129 | D07337 | hsa7166 | D09973 |
| hsa765 | D00518 | hsa2064 | D03350 | hsa4128 | D07338 | hsa121278 | D09974 |
| hsa766 | D00518 | hsa2065 | D03350 | hsa4129 | D07338 | hsa7166 | D09974 |
| hsa767 | D00518 | hsa2066 | D03350 | hsa3067 | D07405 | hsa121278 | D09975 |
| hsa768 | D00518 | hsa2346 | D03372 | hsa5742 | D07421 | hsa7166 | D09975 |
| hsa771 | D00518 | hsa6240 | D03378 | hsa5743 | D07421 | hsa2064 | D09980 |
| hsa5138 | D00528 | hsa4128 | D03409 | hsa10846 | D07425 | hsa8654 | D09989 |
| hsa5139 | D00528 | hsa4129 | D03409 | hsa27115 | D07425 | hsa673 | D09996 |
| hsa5140 | D00528 | hsa5742 | D03410 | hsa50940 | D07425 | hsa211 | D10003 |
| hsa5141 | D00528 | hsa5743 | D03410 | hsa5136 | D07425 | hsa212 | D10003 |
| hsa5142 | D00528 | hsa1636 | D03440 | hsa5137 | D07425 | hsa834 | D10004 |
| hsa5143 | D00528 | hsa6646 | D03447 | hsa5138 | D07425 | hsa5742 | D10017 |
| hsa5144 | D00528 | hsa8435 | D03447 | hsa5139 | D07425 | hsa5743 | D10017 |
| hsa5153 | D00528 | hsa1956 | D03455 | hsa5140 | D07425 | hsa1956 | D10018 |
| hsa8654 | D00528 | hsa5141 | D03516 | hsa5141 | D07425 | hsa10013 | D10019 |
| hsa18 | D00535 | hsa5142 | D03516 | hsa5142 | D07425 | hsa10014 | D10019 |
| hsa760 | D00537 | hsa5143 | D03516 | hsa5143 | D07425 | hsa3065 | D10019 |
| hsa762 | D00537 | hsa5144 | D03516 | hsa5144 | D07425 | hsa3066 | D10019 |
| hsa1644 | D00558 | hsa4312 | D03517 | hsa5150 | D07425 | hsa51564 | D10019 |
| hsa1733 | D00562 | hsa4317 | D03517 | hsa5151 | D07425 | hsa55869 | D10019 |
| hsa1734 | D00562 | hsa4318 | D03517 | hsa5153 | D07425 | hsa79885 | D10019 |
| hsa7173 | D00562 | hsa4322 | D03517 | hsa8622 | D07425 | hsa83933 | D10019 |
| hsa1728 | D00564 | hsa5422 | D03546 | hsa10846 | D07439 | hsa8841 | D10019 |
| hsa79001 | D00564 | hsa6240 | D03546 | hsa27115 | D07439 | hsa9734 | D10019 |
| hsa5742 | D00566 | hsa107 | D03584 | hsa50940 | D07439 | hsa9759 | D10019 |
| hsa5743 | D00566 | hsa108 | D03584 | hsa5136 | D07439 | hsa5604 | D10024 |
| hsa5743 | D00567 | hsa109 | D03584 | hsa5137 | D07439 | hsa5605 | D10024 |
| hsa5743 | D00568 | hsa111 | D03584 | hsa5138 | D07439 | hsa290 | D10026 |
| hsa1588 | D00574 | hsa112 | D03584 | hsa5139 | D07439 | hsa8654 | D10027 |
| hsa7298 | D00584 | hsa113 | D03584 | hsa5140 | D07439 | hsa1956 | D10031 |
| hsa5562 | D00595 | hsa114 | D03584 | hsa5141 | D07439 | hsa3480 | D10056 |
| hsa5563 | D00595 | hsa115 | D03584 | hsa5142 | D07439 | hsa3065 | D10060 |
| hsa1636 | D00620 | hsa196883 | D03584 | hsa5143 | D07439 | hsa8841 | D10060 |
| hsa1636 | D00621 | hsa55811 | D03584 | hsa5144 | D07439 | hsa2321 | D10062 |
| hsa1636 | D00622 | hsa3156 | D03601 | hsa5150 | D07439 | hsa2324 | D10062 |
| hsa1636 | D00623 | hsa7153 | D03602 | hsa5151 | D07439 | hsa3791 | D10062 |
| hsa1636 | D00624 | hsa7155 | D03602 | hsa5153 | D07439 | hsa3815 | D10062 |
| hsa2548 | D00625 | hsa3156 | D03643 | hsa8622 | D07439 | hsa5156 | D10062 |
| hsa2595 | D00625 | hsa7941 | D03650 | hsa5742 | D07443 | hsa5159 | D10062 |
| hsa8972 | D00625 | hsa240 | D03652 | hsa5743 | D07443 | hsa5979 | D10062 |
| hsa476 | D00631 | hsa5743 | D03652 | hsa5139 | D07455 | hsa4881 | D10063 |
| hsa477 | D00631 | hsa8654 | D03657 | hsa5140 | D07455 | hsa4882 | D10063 |
| hsa478 | D00631 | hsa1969 | D03658 | hsa79001 | D07457 | hsa673 | D10064 |
| hsa480 | D00631 | hsa25 | D03658 | hsa1719 | D07472 | hsa2346 | D10070 |
| hsa476 | D00645 | hsa2534 | D03658 | hsa200895 | D07472 | hsa4486 | D10074 |
| hsa477 | D00645 | hsa3815 | D03658 | hsa2618 | D07472 | hsa2475 | D10076 |
| hsa478 | D00645 | hsa3932 | D03658 | hsa7298 | D07472 | hsa10038 | D10079 |
| hsa480 | D00645 | hsa5159 | D03658 | hsa3156 | D07474 | hsa10039 | D10079 |
| hsa760 | D00652 | hsa6714 | D03658 | hsa1636 | D07499 | hsa142 | D10079 |
| hsa11238 | D00653 | hsa7525 | D03658 | hsa240 | D07516 | hsa143 | D10079 |
| hsa23632 | D00653 | hsa6916 | D03661 | hsa5742 | D07516 | hsa3065 | D10084 |
| hsa377677 | D00653 | hsa6916 | D03663 | hsa5743 | D07516 | hsa8841 | D10084 |
| hsa759 | D00653 | hsa1786 | D03665 | hsa240 | D07517 | hsa6790 | D10085 |
| hsa760 | D00653 | hsa1788 | D03665 | hsa5742 | D07517 | hsa6790 | D10086 |
| hsa761 | D00653 | hsa1789 | D03665 | hsa5743 | D07517 | hsa7153 | D10090 |
| hsa762 | D00653 | hsa1991 | D03686 | hsa7173 | D07519 | hsa7155 | D10090 |
| hsa763 | D00653 | hsa5743 | D03689 | hsa476 | D07520 | hsa7153 | D10091 |
| hsa765 | D00653 | hsa2147 | D03692 | hsa477 | D07520 | hsa7155 | D10091 |
| hsa766 | D00653 | hsa5743 | D03710 | hsa478 | D07520 | hsa2321 | D10095 |
| hsa767 | D00653 | hsa5743 | D03712 | hsa480 | D07520 | hsa2324 | D10095 |
| hsa768 | D00653 | hsa5743 | D03714 | hsa5742 | D07541 | hsa3791 | D10095 |
| hsa771 | D00653 | hsa5742 | D03715 | hsa5743 | D07541 | hsa3815 | D10095 |
| hsa11238 | D00655 | hsa5743 | D03715 | hsa10846 | D07546 | hsa5156 | D10095 |
| hsa23632 | D00655 | hsa5743 | D03716 | hsa27115 | D07546 | hsa5159 | D10095 |
| hsa377677 | D00655 | hsa5743 | D03717 | hsa50940 | D07546 | hsa5979 | D10095 |
| hsa759 | D00655 | hsa5742 | D03718 | hsa5136 | D07546 | hsa2322 | D10102 |
| hsa760 | D00655 | hsa5743 | D03718 | hsa5137 | D07546 | hsa5156 | D10102 |
| hsa761 | D00655 | hsa2339 | D03720 | hsa5138 | D07546 | hsa5159 | D10102 |
| hsa762 | D00655 | hsa2342 | D03720 | hsa5139 | D07546 | hsa2322 | D10103 |
| hsa763 | D00655 | hsa2147 | D03722 | hsa5140 | D07546 | hsa5156 | D10103 |
| hsa765 | D00655 | hsa2147 | D03728 | hsa5141 | D07546 | hsa5159 | D10103 |
| hsa766 | D00655 | hsa7153 | D03730 | hsa5142 | D07546 | hsa673 | D10104 |
| hsa767 | D00655 | hsa7155 | D03730 | hsa5143 | D07546 | hsa94 | D10106 |
| hsa768 | D00655 | hsa4129 | D03731 | hsa5144 | D07546 | hsa5689 | D10110 |
| hsa771 | D00655 | hsa4129 | D03733 | hsa5150 | D07546 | hsa5693 | D10110 |
| hsa43 | D00667 | hsa6646 | D03734 | hsa5151 | D07546 | hsa495 | D10120 |
| hsa43 | D00670 | hsa8435 | D03734 | hsa5153 | D07546 | hsa496 | D10120 |
| hsa10846 | D00691 | hsa6646 | D03735 | hsa8622 | D07546 | hsa1586 | D10125 |
| hsa27115 | D00691 | hsa8435 | D03735 | hsa476 | D07555 | hsa5693 | D10130 |
| hsa50940 | D00691 | hsa1432 | D03736 | hsa477 | D07555 | hsa5693 | D10131 |
| hsa5136 | D00691 | hsa5600 | D03736 | hsa478 | D07555 | hsa1080 | D10134 |
| hsa5137 | D00691 | hsa5603 | D03736 | hsa480 | D07555 | hsa2260 | D10137 |
| hsa5138 | D00691 | hsa6300 | D03736 | hsa476 | D07556 | hsa2321 | D10137 |
| hsa5139 | D00691 | hsa5972 | D03738 | hsa477 | D07556 | hsa2324 | D10137 |
| hsa5140 | D00691 | hsa5972 | D03741 | hsa478 | D07556 | hsa3791 | D10137 |
| hsa5141 | D00691 | hsa5972 | D03743 | hsa480 | D07556 | hsa3815 | D10137 |
| hsa5142 | D00691 | hsa5972 | D03745 | hsa7498 | D07564 | hsa5159 | D10137 |
| hsa5143 | D00691 | hsa4023 | D03747 | hsa5340 | D07568 | hsa5894 | D10137 |
| hsa5144 | D00691 | hsa1588 | D03749 | hsa2977 | D07577 | hsa5979 | D10137 |
| hsa5150 | D00691 | hsa43 | D03751 | hsa2982 | D07577 | hsa673 | D10137 |
| hsa5151 | D00691 | hsa1636 | D03752 | hsa2983 | D07577 | hsa7010 | D10137 |
| hsa5153 | D00691 | hsa1636 | D03753 | hsa5742 | D07579 | hsa2260 | D10138 |
| hsa8622 | D00691 | hsa1636 | D03756 | hsa5743 | D07579 | hsa2321 | D10138 |
| hsa124 | D00707 | hsa1636 | D03758 | hsa5742 | D07580 | hsa2324 | D10138 |
| hsa125 | D00707 | hsa1636 | D03760 | hsa5743 | D07580 | hsa3791 | D10138 |
| hsa126 | D00707 | hsa1636 | D03763 | hsa5742 | D07581 | hsa3815 | D10138 |
| hsa18 | D00710 | hsa1636 | D03765 | hsa5743 | D07581 | hsa5159 | D10138 |
| hsa2571 | D00710 | hsa1636 | D03767 | hsa5742 | D07582 | hsa5894 | D10138 |
| hsa2572 | D00710 | hsa1636 | D03769 | hsa5743 | D07582 | hsa5979 | D10138 |
| hsa7915 | D00710 | hsa1636 | D03772 | hsa5138 | D07603 | hsa673 | D10138 |
| hsa10846 | D00718 | hsa1636 | D03773 | hsa5139 | D07603 | hsa7010 | D10138 |
| hsa27115 | D00718 | hsa1636 | D03775 | hsa5140 | D07603 | hsa10038 | D10140 |
| hsa50940 | D00718 | hsa1636 | D03776 | hsa5141 | D07603 | hsa142 | D10140 |
| hsa5136 | D00718 | hsa1588 | D03778 | hsa5142 | D07603 | hsa3716 | D10141 |
| hsa5137 | D00718 | hsa659 | D03779 | hsa5143 | D07603 | hsa3716 | D10142 |
| hsa5138 | D00718 | hsa1588 | D03781 | hsa5144 | D07603 | hsa1586 | D10146 |
| hsa5139 | D00718 | hsa1588 | D03784 | hsa5153 | D07603 | hsa2158 | D10150 |
| hsa5140 | D00718 | hsa1588 | D03786 | hsa8654 | D07603 | hsa5347 | D10154 |
| hsa5141 | D00718 | hsa1621 | D03787 | hsa3290 | D07615 | hsa5347 | D10155 |
| hsa5142 | D00718 | hsa1991 | D03788 | hsa7173 | D07616 | hsa10038 | D10157 |
| hsa5143 | D00718 | hsa4313 | D03793 | hsa3156 | D07661 | hsa10039 | D10157 |
| hsa5144 | D00718 | hsa4318 | D03793 | hsa1800 | D07698 | hsa142 | D10157 |
| hsa5150 | D00718 | hsa4312 | D03795 | hsa1636 | D07699 | hsa143 | D10157 |
| hsa5151 | D00718 | hsa4313 | D03795 | hsa7153 | D07776 | hsa4129 | D10158 |
| hsa5153 | D00718 | hsa4314 | D03795 | hsa7155 | D07776 | hsa5599 | D10168 |
| hsa8622 | D00718 | hsa4316 | D03795 | hsa1636 | D07781 | hsa5601 | D10168 |
| hsa495 | D00724 | hsa4318 | D03795 | hsa7153 | D07807 | hsa5602 | D10168 |
| hsa496 | D00724 | hsa4321 | D03795 | hsa7155 | D07807 | hsa1723 | D10172 |
| hsa1723 | D00749 | hsa4313 | D03797 | hsa5742 | D07816 | hsa4233 | D10173 |
| hsa3614 | D00752 | hsa4314 | D03797 | hsa5743 | D07816 | hsa5604 | D10175 |
| hsa3615 | D00752 | hsa4318 | D03797 | hsa5742 | D07817 | hsa5605 | D10175 |
| hsa2475 | D00753 | hsa4322 | D03797 | hsa5743 | D07817 | hsa5604 | D10176 |
| hsa7054 | D00762 | hsa4323 | D03797 | hsa5742 | D07818 | hsa5605 | D10176 |
| hsa1312 | D00781 | hsa79001 | D03798 | hsa5743 | D07818 | hsa1803 | D10178 |
| hsa4129 | D00785 | hsa4312 | D03800 | hsa5742 | D07819 | hsa1803 | D10179 |
| hsa1312 | D00786 | hsa4313 | D03800 | hsa5743 | D07819 | hsa5347 | D10182 |
| hsa5742 | D00810 | hsa4317 | D03800 | hsa43 | D07869 | hsa5347 | D10183 |
| hsa5743 | D00810 | hsa4318 | D03800 | hsa11238 | D07871 | hsa5290 | D10189 |
| hsa5742 | D00813 | hsa4323 | D03800 | hsa23632 | D07871 | hsa5291 | D10189 |
| hsa5743 | D00813 | hsa4312 | D03802 | hsa377677 | D07871 | hsa5293 | D10189 |
| hsa4128 | D00826 | hsa4313 | D03802 | hsa759 | D07871 | hsa2321 | D10190 |
| hsa4129 | D00826 | hsa4318 | D03802 | hsa760 | D07871 | hsa2324 | D10190 |
| hsa5742 | D00827 | hsa231 | D03803 | hsa761 | D07871 | hsa3791 | D10190 |
| hsa5743 | D00827 | hsa231 | D03805 | hsa762 | D07871 | hsa4129 | D10191 |
| hsa4953 | D00829 | hsa231 | D03806 | hsa763 | D07871 | hsa25 | D10202 |
| hsa3156 | D00887 | hsa231 | D03807 | hsa765 | D07871 | hsa4067 | D10202 |
| hsa3156 | D00889 | hsa3156 | D03816 | hsa766 | D07871 | hsa6240 | D10222 |
| hsa3156 | D00892 | hsa6715 | D03820 | hsa767 | D07871 | hsa695 | D10223 |
| hsa3156 | D00893 | hsa6716 | D03820 | hsa768 | D07871 | hsa3791 | D10224 |
| hsa5742 | D00903 | hsa43 | D03822 | hsa771 | D07871 | hsa4233 | D10224 |
| hsa5743 | D00903 | hsa590 | D03822 | hsa5141 | D07879 | hsa4311 | D10225 |
| hsa5742 | D00904 | hsa43 | D03823 | hsa5142 | D07879 | hsa3815 | D10229 |
| hsa5743 | D00904 | hsa43 | D03826 | hsa5143 | D07879 | hsa5742 | D10254 |
| hsa2224 | D00939 | hsa1719 | D03828 | hsa5144 | D07879 | hsa5743 | D10254 |
| hsa2224 | D00941 | hsa200895 | D03828 | hsa4953 | D07883 | hsa5139 | D10255 |
| hsa2224 | D00942 | hsa2618 | D03828 | hsa5743 | D07888 | hsa5140 | D10255 |
| hsa5562 | D00944 | hsa7298 | D03828 | hsa1636 | D07892 | hsa1803 | D10262 |
| hsa5563 | D00944 | hsa11238 | D03845 | hsa7153 | D07901 | hsa3716 | D10308 |
| hsa1588 | D00960 | hsa23632 | D03845 | hsa7155 | D07901 | hsa3717 | D10308 |
| hsa1588 | D00963 | hsa377677 | D03845 | hsa1956 | D07907 | hsa3716 | D10315 |
| hsa1588 | D00964 | hsa759 | D03845 | hsa495 | D07917 | hsa3717 | D10315 |
| hsa3480 | D00967 | hsa760 | D03845 | hsa496 | D07917 | hsa1803 | D10317 |
| hsa5742 | D00968 | hsa761 | D03845 | hsa1588 | D07940 | hsa5693 | D10318 |
| hsa5743 | D00968 | hsa762 | D03845 | hsa6093 | D07941 | hsa10013 | D10319 |
| hsa5743 | D00969 | hsa763 | D03845 | hsa9475 | D07941 | hsa10014 | D10319 |
| hsa5742 | D00970 | hsa765 | D03845 | hsa10846 | D07961 | hsa3065 | D10319 |
| hsa5743 | D00970 | hsa766 | D03845 | hsa27115 | D07961 | hsa3066 | D10319 |
| hsa43 | D00994 | hsa767 | D03845 | hsa50940 | D07961 | hsa51564 | D10319 |
| hsa43 | D00995 | hsa768 | D03845 | hsa5136 | D07961 | hsa55869 | D10319 |
| hsa43 | D00998 | hsa771 | D03845 | hsa5137 | D07961 | hsa79885 | D10319 |
| hsa43 | D01001 | hsa240 | D03882 | hsa5138 | D07961 | hsa83933 | D10319 |
| hsa5743 | D01049 | hsa5138 | D03898 | hsa5139 | D07961 | hsa8841 | D10319 |
| hsa7150 | D01061 | hsa7153 | D03899 | hsa5140 | D07961 | hsa9734 | D10319 |
| hsa7298 | D01064 | hsa7155 | D03899 | hsa5141 | D07961 | hsa9759 | D10319 |
| hsa1636 | D01069 | hsa4311 | D03929 | hsa5142 | D07961 | hsa10013 | D10321 |
| hsa4311 | D01070 | hsa3818 | D03931 | hsa5143 | D07961 | hsa10014 | D10321 |
| hsa5742 | D01090 | hsa5321 | D03938 | hsa5144 | D07961 | hsa3065 | D10321 |
| hsa5743 | D01090 | hsa1719 | D03942 | hsa5150 | D07961 | hsa3066 | D10321 |
| hsa4129 | D01097 | hsa200895 | D03942 | hsa5151 | D07961 | hsa51564 | D10321 |
| hsa1636 | D01119 | hsa7150 | D03954 | hsa5153 | D07961 | hsa55869 | D10321 |
| hsa5742 | D01122 | hsa5243 | D03968 | hsa8622 | D07961 | hsa79885 | D10321 |
| hsa5743 | D01122 | hsa7150 | D03977 | hsa6240 | D07966 | hsa83933 | D10321 |
| hsa5139 | D01133 | hsa7153 | D03977 | hsa79001 | D07969 | hsa8841 | D10321 |
| hsa5140 | D01133 | hsa7155 | D03977 | hsa7298 | D07974 | hsa9734 | D10321 |
| hsa6715 | D01134 | hsa240 | D03990 | hsa3156 | D07983 | hsa9759 | D10321 |
| hsa6716 | D01134 | hsa1806 | D03998 | hsa1636 | D07992 | hsa10013 | D10322 |
| hsa5340 | D01136 | hsa5139 | D04004 | hsa3816 | D08004 | hsa10014 | D10322 |
| hsa6240 | D01155 | hsa5140 | D04004 | hsa3817 | D08004 | hsa3065 | D10322 |
| hsa3283 | D01180 | hsa10846 | D04006 | hsa3818 | D08004 | hsa3066 | D10322 |
| hsa3284 | D01180 | hsa27115 | D04006 | hsa7153 | D08024 | hsa51564 | D10322 |
| hsa5742 | D01183 | hsa50940 | D04006 | hsa7155 | D08024 | hsa55869 | D10322 |
| hsa5743 | D01183 | hsa5136 | D04006 | hsa8639 | D08044 | hsa79885 | D10322 |
| hsa11238 | D01196 | hsa5137 | D04006 | hsa2224 | D08056 | hsa83933 | D10322 |
| hsa23632 | D01196 | hsa5138 | D04006 | hsa5742 | D08058 | hsa8841 | D10322 |
| hsa377677 | D01196 | hsa5139 | D04006 | hsa5743 | D08058 | hsa9734 | D10322 |
| hsa759 | D01196 | hsa5140 | D04006 | hsa5742 | D08059 | hsa9759 | D10322 |
| hsa760 | D01196 | hsa5141 | D04006 | hsa5743 | D08059 | hsa25 | D10334 |
| hsa761 | D01196 | hsa5142 | D04006 | hsa7153 | D08062 | hsa7010 | D10334 |
| hsa762 | D01196 | hsa5143 | D04006 | hsa7155 | D08062 | hsa255738 | D10335 |
| hsa763 | D01196 | hsa5144 | D04006 | hsa25 | D08066 | hsa2064 | D10344 |
| hsa765 | D01196 | hsa5150 | D04006 | hsa3815 | D08066 | hsa3716 | D10358 |
| hsa766 | D01196 | hsa5151 | D04006 | hsa5156 | D08066 | hsa3717 | D10358 |
| hsa767 | D01196 | hsa5153 | D04006 | hsa1636 | D08068 | hsa3717 | D10365 |
| hsa768 | D01196 | hsa8622 | D04006 | hsa2224 | D08073 | hsa7150 | D10367 |
| hsa771 | D01196 | hsa5579 | D04014 | hsa3643 | D08080 | hsa1019 | D10372 |
| hsa5139 | D01198 | hsa1956 | D04023 | hsa4128 | D08085 | hsa1021 | D10372 |
| hsa5140 | D01198 | hsa1956 | D04024 | hsa4129 | D08085 | hsa10000 | D10381 |
| hsa6646 | D01202 | hsa2064 | D04024 | hsa7150 | D08086 | hsa207 | D10381 |
| hsa8435 | D01202 | hsa2064 | D04025 | hsa43 | D08094 | hsa208 | D10381 |
| hsa7498 | D01206 | hsa5406 | D04028 | hsa5742 | D08102 | hsa10000 | D10382 |
| hsa495 | D01207 | hsa5407 | D04028 | hsa5743 | D08102 | hsa207 | D10382 |
| hsa496 | D01207 | hsa5408 | D04028 | hsa5742 | D08103 | hsa208 | D10382 |
| hsa10846 | D01220 | hsa8513 | D04028 | hsa5743 | D08103 | hsa7153 | D10383 |
| hsa27115 | D01220 | hsa2159 | D04029 | hsa5742 | D08104 | hsa7155 | D10383 |
| hsa50940 | D01220 | hsa7150 | D04031 | hsa5743 | D08104 | hsa7153 | D10384 |
| hsa5136 | D01220 | hsa3283 | D04035 | hsa26279 | D08107 | hsa7155 | D10384 |
| hsa5137 | D01220 | hsa3284 | D04035 | hsa30814 | D08107 | hsa4843 | D10386 |
| hsa5138 | D01220 | hsa3551 | D04050 | hsa391013 | D08107 | hsa2260 | D10396 |
| hsa5139 | D01220 | hsa5770 | D04050 | hsa50487 | D08107 | hsa2261 | D10396 |
| hsa5140 | D01220 | hsa4881 | D04051 | hsa5319 | D08107 | hsa2263 | D10396 |
| hsa5141 | D01220 | hsa4882 | D04051 | hsa5320 | D08107 | hsa2321 | D10396 |
| hsa5142 | D01220 | hsa495 | D04056 | hsa5322 | D08107 | hsa2324 | D10396 |
| hsa5143 | D01220 | hsa496 | D04056 | hsa64600 | D08107 | hsa3791 | D10396 |
| hsa5144 | D01220 | hsa6716 | D04066 | hsa81579 | D08107 | hsa5156 | D10396 |
| hsa5150 | D01220 | hsa4128 | D04092 | hsa8399 | D08107 | hsa5159 | D10396 |
| hsa5151 | D01220 | hsa4129 | D04092 | hsa84647 | D08107 | hsa1111 | D10397 |
| hsa5153 | D01220 | hsa5742 | D04102 | hsa1956 | D08108 | hsa25 | D10399 |
| hsa6916 | D01220 | hsa5743 | D04102 | hsa2064 | D08108 | hsa7010 | D10399 |
| hsa8622 | D01220 | hsa7153 | D04107 | hsa1636 | D08131 | hsa5604 | D10405 |
| hsa7298 | D01223 | hsa7155 | D04107 | hsa5742 | D08149 | hsa5605 | D10405 |
| hsa43 | D01228 | hsa240 | D04151 | hsa5743 | D08149 | hsa5156 | D10411 |
| hsa10846 | D01238 | hsa2159 | D04183 | hsa5742 | D08162 | hsa834 | D10416 |
| hsa27115 | D01238 | hsa5141 | D04185 | hsa5743 | D08162 | hsa4843 | D10419 |
| hsa50940 | D01238 | hsa5142 | D04185 | hsa26279 | D08221 | hsa7150 | D10422 |
| hsa5136 | D01238 | hsa5143 | D04185 | hsa30814 | D08221 | hsa2321 | D10423 |
| hsa5137 | D01238 | hsa5144 | D04185 | hsa391013 | D08221 | hsa5156 | D10423 |
| hsa5138 | D01238 | hsa7298 | D04197 | hsa50487 | D08221 | hsa5159 | D10423 |
| hsa5139 | D01238 | hsa4860 | D04245 | hsa5319 | D08221 | hsa6714 | D10423 |
| hsa5140 | D01238 | hsa5515 | D04262 | hsa5320 | D08221 | hsa6790 | D10423 |
| hsa5141 | D01238 | hsa5516 | D04262 | hsa5322 | D08221 | hsa6795 | D10423 |
| hsa5142 | D01238 | hsa5531 | D04262 | hsa64600 | D08221 | hsa9212 | D10423 |
| hsa5143 | D01238 | hsa7153 | D04262 | hsa81579 | D08221 | hsa5604 | D10426 |
| hsa5144 | D01238 | hsa7155 | D04262 | hsa8399 | D08221 | hsa5605 | D10426 |
| hsa5150 | D01238 | hsa3931 | D04268 | hsa84647 | D08221 | hsa7150 | D10427 |
| hsa5151 | D01238 | hsa4023 | D04268 | hsa7153 | D08224 | hsa127550 | D10429 |
| hsa5153 | D01238 | hsa6916 | D04276 | hsa7155 | D08224 | hsa4860 | D10431 |
| hsa8622 | D01238 | hsa43 | D04292 | hsa1636 | D08225 | hsa4860 | D10432 |
| hsa476 | D01240 | hsa1636 | D04312 | hsa10846 | D08238 | hsa7046 | D10437 |
| hsa477 | D01240 | hsa7941 | D04368 | hsa27115 | D08238 | hsa1956 | D10439 |
| hsa478 | D01240 | hsa590 | D04435 | hsa50940 | D08238 | hsa1675 | D10440 |
| hsa480 | D01240 | hsa8639 | D04458 | hsa5136 | D08238 | hsa2064 | D10446 |
| hsa7298 | D01244 | hsa3643 | D04475 | hsa5137 | D08238 | hsa238 | D10450 |
| hsa5743 | D01252 | hsa3643 | D04477 | hsa5138 | D08238 | hsa5580 | D10456 |
| hsa1312 | D01259 | hsa2224 | D04486 | hsa5139 | D08238 | hsa5580 | D10457 |
| hsa7153 | D01264 | hsa5742 | D04490 | hsa5140 | D08238 | hsa6093 | D10463 |
| hsa7155 | D01264 | hsa5743 | D04490 | hsa5141 | D08238 | hsa9475 | D10463 |
| hsa5742 | D01271 | hsa6715 | D04498 | hsa5142 | D08238 | hsa7150 | D10464 |
| hsa5743 | D01271 | hsa6716 | D04498 | hsa5143 | D08238 | hsa3791 | D10465 |
| hsa7153 | D01275 | hsa7153 | D04498 | hsa5144 | D08238 | hsa4233 | D10465 |
| hsa7155 | D01275 | hsa7155 | D04498 | hsa5150 | D08238 | hsa495 | D10466 |
| hsa1728 | D01280 | hsa79644 | D04498 | hsa5151 | D08238 | hsa496 | D10466 |
| hsa79001 | D01280 | hsa5139 | D04508 | hsa5153 | D08238 | hsa2159 | D10471 |
| hsa7298 | D01309 | hsa5140 | D04508 | hsa8622 | D08238 | hsa3643 | D10473 |
| hsa2977 | D01320 | hsa2346 | D04525 | hsa43 | D08261 | hsa2260 | D10481 |
| hsa5742 | D01325 | hsa5139 | D04529 | hsa5743 | D08275 | hsa2261 | D10481 |
| hsa5743 | D01325 | hsa5140 | D04529 | hsa2322 | D08279 | hsa2263 | D10481 |
| hsa5742 | D01344 | hsa5834 | D04537 | hsa25 | D08279 | hsa2321 | D10481 |
| hsa5743 | D01344 | hsa5836 | D04537 | hsa6790 | D08279 | hsa2324 | D10481 |
| hsa6240 | D01370 | hsa5837 | D04537 | hsa6795 | D08279 | hsa3791 | D10481 |
| hsa476 | D01379 | hsa3643 | D04539 | hsa9212 | D08279 | hsa5156 | D10481 |
| hsa477 | D01379 | hsa3643 | D04540 | hsa5139 | D08294 | hsa5159 | D10481 |
| hsa478 | D01379 | hsa3643 | D04541 | hsa5140 | D08294 | hsa2158 | D10484 |
| hsa480 | D01379 | hsa3643 | D04542 | hsa5742 | D08324 | hsa5604 | D10486 |
| hsa5139 | D01385 | hsa3643 | D04543 | hsa5743 | D08324 | hsa5605 | D10486 |
| hsa5140 | D01385 | hsa3643 | D04544 | hsa6916 | D08327 | hsa1803 | D10502 |
| hsa5141 | D01385 | hsa3643 | D04545 | hsa126129 | D08340 | hsa1956 | D10514 |
| hsa5142 | D01385 | hsa3643 | D04546 | hsa1374 | D08340 | hsa2064 | D10514 |
| hsa5143 | D01385 | hsa3643 | D04547 | hsa1375 | D08340 | hsa2066 | D10514 |
| hsa5144 | D01385 | hsa3643 | D04548 | hsa1376 | D08340 | hsa2224 | D10515 |
| hsa3614 | D01392 | hsa3643 | D04549 | hsa2322 | D08344 | hsa43 | D10529 |
| hsa3615 | D01392 | hsa3643 | D04550 | hsa25 | D08344 | hsa238 | D10542 |
| hsa5742 | D01397 | hsa3643 | D04551 | hsa6790 | D08344 | hsa2475 | D10543 |
| hsa5743 | D01397 | hsa5139 | D04628 | hsa6795 | D08344 | hsa5290 | D10543 |
| hsa7153 | D01404 | hsa5140 | D04628 | hsa9212 | D08344 | hsa5291 | D10543 |
| hsa7155 | D01404 | hsa6715 | D04646 | hsa4128 | D08349 | hsa5293 | D10543 |
| hsa240 | D01410 | hsa6716 | D04646 | hsa4129 | D08349 | hsa238 | D10551 |
| hsa5742 | D01410 | hsa4129 | D04681 | hsa5562 | D08351 | hsa2475 | D10552 |
| hsa5743 | D01410 | hsa7153 | D04685 | hsa5563 | D08351 | hsa5290 | D10552 |
| hsa7150 | D01432 | hsa7155 | D04685 | hsa5562 | D08352 | hsa5291 | D10552 |
| hsa25 | D01441 | hsa2322 | D04696 | hsa5563 | D08352 | hsa5293 | D10552 |
| hsa3815 | D01441 | hsa5139 | D04720 | hsa79001 | D08354 | hsa5293 | D10555 |
| hsa5156 | D01441 | hsa5140 | D04720 | hsa5742 | D08363 | hsa5294 | D10555 |
| hsa5138 | D01453 | hsa5139 | D04751 | hsa5743 | D08363 | hsa255738 | D10557 |
| hsa5139 | D01453 | hsa5140 | D04751 | hsa5742 | D08364 | hsa5293 | D10560 |
| hsa5140 | D01453 | hsa2339 | D04768 | hsa5743 | D08364 | hsa3156 | D10568 |
| hsa5141 | D01453 | hsa2342 | D04768 | hsa1312 | D08369 | hsa5290 | D10584 |
| hsa5142 | D01453 | hsa240 | D04770 | hsa5742 | D08374 | hsa5291 | D10584 |
| hsa5143 | D01453 | hsa7153 | D04783 | hsa5743 | D08374 | hsa5293 | D10584 |
| hsa5144 | D01453 | hsa7155 | D04783 | hsa5742 | D08375 | hsa3718 | D10585 |
| hsa5153 | D01453 | hsa7153 | D04791 | hsa5743 | D08375 | hsa112398 | D10593 |
| hsa8654 | D01453 | hsa7155 | D04791 | hsa7153 | D08386 | hsa112399 | D10593 |
| hsa5742 | D01475 | hsa283208 | D04793 | hsa7155 | D08386 | hsa54583 | D10593 |
| hsa5743 | D01475 | hsa5033 | D04793 | hsa4128 | D08392 | hsa1719 | D10596 |
| hsa5742 | D01513 | hsa8974 | D04793 | hsa4128 | D08393 | hsa200895 | D10596 |
| hsa5743 | D01513 | hsa7150 | D04822 | hsa3156 | D08410 | hsa2618 | D10596 |
| hsa5742 | D01527 | hsa1956 | D04862 | hsa5742 | D08427 | hsa7298 | D10596 |
| hsa5742 | D01545 | hsa5743 | D04863 | hsa5743 | D08427 | hsa5604 | D10604 |
| hsa5743 | D01545 | hsa3480 | D04870 | hsa5742 | D08434 | hsa5605 | D10604 |
| hsa9536 | D01545 | hsa3251 | D04931 | hsa5743 | D08434 | hsa5290 | D10609 |
| hsa5742 | D01547 | hsa3614 | D04936 | hsa4128 | D08453 | hsa5291 | D10609 |
| hsa5743 | D01547 | hsa3615 | D04936 | hsa4129 | D08453 | hsa5293 | D10609 |
| hsa1636 | D01549 | hsa7298 | D04964 | hsa495 | D08463 | hsa5604 | D10615 |
| hsa5742 | D01557 | hsa5562 | D04966 | hsa496 | D08463 | hsa5605 | D10615 |
| hsa5743 | D01557 | hsa5563 | D04966 | hsa4311 | D08464 | hsa2475 | D10616 |
| hsa5742 | D01565 | hsa3290 | D04987 | hsa4129 | D08469 | hsa5290 | D10616 |
| hsa5743 | D01565 | hsa1584 | D05019 | hsa2224 | D08484 | hsa5291 | D10616 |
| hsa43 | D01572 | hsa2322 | D05029 | hsa3156 | D08492 | hsa5293 | D10616 |
| hsa5742 | D01578 | hsa3791 | D05029 | hsa5742 | D08501 | hsa2185 | D10618 |
| hsa5743 | D01578 | hsa3815 | D05029 | hsa5743 | D08501 | hsa2185 | D10619 |
| hsa5742 | D01581 | hsa5156 | D05029 | hsa3791 | D08503 | hsa92 | D10620 |
| hsa5743 | D01581 | hsa5159 | D05029 | hsa3815 | D08503 | hsa93 | D10620 |
| hsa5742 | D01582 | hsa5578 | D05029 | hsa5156 | D08503 | hsa255738 | D10621 |
| hsa5743 | D01582 | hsa5579 | D05029 | hsa5159 | D08503 | hsa3717 | D10630 |
| hsa5141 | D01630 | hsa5582 | D05029 | hsa8654 | D08514 | hsa2475 | D10635 |
| hsa5142 | D01630 | hsa7357 | D05032 | hsa1803 | D08516 | hsa5290 | D10635 |
| hsa5143 | D01630 | hsa4129 | D05033 | hsa2322 | D08524 | hsa5291 | D10635 |
| hsa5144 | D01630 | hsa4128 | D05039 | hsa2324 | D08524 | hsa5293 | D10635 |
| hsa1644 | D01653 | hsa4128 | D05040 | hsa3791 | D08524 | hsa169355 | D10640 |
| hsa2548 | D01665 | hsa3614 | D05094 | hsa3815 | D08524 | hsa3620 | D10640 |
| hsa2595 | D01665 | hsa3615 | D05094 | hsa5159 | D08524 | hsa10000 | D10641 |
| hsa8972 | D01665 | hsa3614 | D05095 | hsa5894 | D08524 | hsa207 | D10641 |
| hsa1636 | D01667 | hsa3615 | D05095 | hsa5979 | D08524 | hsa208 | D10641 |
| hsa3816 | D01672 | hsa3614 | D05096 | hsa673 | D08524 | hsa7150 | D10642 |
| hsa3817 | D01672 | hsa3615 | D05096 | hsa1636 | D08529 | hsa1019 | D10652 |
| hsa3818 | D01672 | hsa5742 | D05143 | hsa3791 | D08544 | hsa1021 | D10652 |
| hsa5742 | D01675 | hsa5743 | D05143 | hsa3815 | D08544 | hsa3716 | D10653 |
| hsa5743 | D01675 | hsa4881 | D05147 | hsa5156 | D08544 | hsa3717 | D10653 |
| hsa6916 | D01683 | hsa4881 | D05148 | hsa5159 | D08544 | hsa3718 | D10653 |
| hsa6916 | D01684 | hsa3242 | D05177 | hsa2321 | D08552 | hsa5743 | D10656 |
| hsa231 | D01688 | hsa5742 | D05181 | hsa2322 | D08552 | hsa8694 | D10657 |
| hsa5139 | D01690 | hsa5743 | D05181 | hsa2324 | D08552 | hsa1432 | D10658 |
| hsa5140 | D01690 | hsa1504 | D05183 | hsa3791 | D08552 | hsa5600 | D10658 |
| hsa107 | D01697 | hsa1991 | D05183 | hsa3815 | D08552 | hsa5603 | D10658 |
| hsa108 | D01697 | hsa440387 | D05183 | hsa5156 | D08552 | hsa6300 | D10658 |
| hsa109 | D01697 | hsa5644 | D05183 | hsa5159 | D08552 | hsa1432 | D10659 |
| hsa111 | D01697 | hsa5645 | D05183 | hsa5979 | D08552 | hsa5600 | D10659 |
| hsa112 | D01697 | hsa5646 | D05183 | hsa43 | D08555 | hsa5603 | D10659 |
| hsa113 | D01697 | hsa43 | D05215 | hsa5530 | D08556 | hsa6300 | D10659 |
| hsa114 | D01697 | hsa4023 | D05255 | hsa5532 | D08556 | hsa10013 | D10661 |
| hsa115 | D01697 | hsa495 | D05259 | hsa5533 | D08556 | hsa8694 | D10664 |
| hsa196883 | D01697 | hsa496 | D05259 | hsa1636 | D08566 | hsa10000 | D10674 |
| hsa55811 | D01697 | hsa495 | D05261 | hsa3251 | D08603 | hsa207 | D10674 |
| hsa5141 | D01704 | hsa496 | D05261 | hsa1803 | D08616 | hsa208 | D10674 |
| hsa5142 | D01704 | hsa126129 | D05292 | hsa7150 | D08618 | hsa1019 | D10688 |
| hsa5143 | D01704 | hsa1374 | D05292 | hsa3480 | D08620 | hsa1021 | D10688 |
| hsa5144 | D01704 | hsa1375 | D05292 | hsa4128 | D08625 | hsa47 | D10691 |
| hsa5742 | D01709 | hsa5742 | D05319 | hsa4129 | D08625 | hsa4233 | D10696 |
| hsa5743 | D01709 | hsa5743 | D05319 | hsa1803 | D08631 | hsa2322 | D10709 |
| hsa10846 | D01712 | hsa2263 | D05338 | hsa5743 | D08657 | hsa558 | D10709 |
| hsa27115 | D01712 | hsa1956 | D05350 | hsa18 | D08667 | hsa487 | D10715 |
| hsa50940 | D01712 | hsa495 | D05353 | hsa2571 | D08667 | hsa488 | D10715 |
| hsa5136 | D01712 | hsa496 | D05353 | hsa2572 | D08667 | hsa489 | D10715 |
| hsa5137 | D01712 | hsa2321 | D05380 | hsa7915 | D08667 | hsa2475 | D10718 |
| hsa5138 | D01712 | hsa2324 | D05380 | hsa8654 | D08668 | hsa5290 | D10718 |
| hsa5139 | D01712 | hsa3791 | D05380 | hsa1728 | D08682 | hsa5291 | D10718 |
| hsa5140 | D01712 | hsa3815 | D05380 | hsa79001 | D08682 | hsa5293 | D10718 |
| hsa5141 | D01712 | hsa5156 | D05380 | hsa1636 | D08688 | hsa3716 | D10721 |
| hsa5142 | D01712 | hsa5159 | D05380 | hsa2224 | D08689 | hsa3717 | D10721 |
| hsa5143 | D01712 | hsa7150 | D05385 | hsa43 | D08838 | hsa3718 | D10721 |
| hsa5144 | D01712 | hsa1956 | D05399 | hsa7153 | D08854 | hsa3716 | D10728 |
| hsa5150 | D01712 | hsa2618 | D05400 | hsa7155 | D08854 | hsa695 | D10730 |
| hsa5151 | D01712 | hsa10846 | D05429 | hsa2159 | D08858 | hsa695 | D10731 |
| hsa5153 | D01712 | hsa27115 | D05429 | hsa4322 | D08859 | hsa10038 | D10732 |
| hsa8622 | D01712 | hsa50940 | D05429 | hsa6868 | D08859 | hsa10039 | D10732 |
| hsa231 | D01715 | hsa5136 | D05429 | hsa5141 | D08860 | hsa142 | D10732 |
| hsa5742 | D01718 | hsa5137 | D05429 | hsa5142 | D08860 | hsa143 | D10732 |
| hsa7153 | D01747 | hsa5138 | D05429 | hsa5143 | D08860 | hsa10038 | D10733 |
| hsa7155 | D01747 | hsa5139 | D05429 | hsa5144 | D08860 | hsa10039 | D10733 |
| hsa5742 | D01765 | hsa5140 | D05429 | hsa5663 | D08869 | hsa142 | D10733 |
| hsa5743 | D01765 | hsa5141 | D05429 | hsa10013 | D08870 | hsa143 | D10733 |
| hsa5742 | D01767 | hsa5142 | D05429 | hsa10014 | D08870 | hsa6093 | D10737 |
| hsa5743 | D01767 | hsa5143 | D05429 | hsa3065 | D08870 | hsa9475 | D10737 |
| hsa10846 | D01771 | hsa5144 | D05429 | hsa3066 | D08870 | hsa6093 | D10738 |
| hsa27115 | D01771 | hsa5150 | D05429 | hsa51564 | D08870 | hsa9475 | D10738 |
| hsa50940 | D01771 | hsa5151 | D05429 | hsa55869 | D08870 | hsa23621 | D10739 |
| hsa5136 | D01771 | hsa5153 | D05429 | hsa79885 | D08870 | hsa25825 | D10739 |
| hsa5137 | D01771 | hsa8622 | D05429 | hsa83933 | D08870 | hsa1956 | D10766 |
| hsa5138 | D01771 | hsa126129 | D05442 | hsa8841 | D08870 | hsa952 | D10777 |
| hsa5139 | D01771 | hsa1374 | D05442 | hsa9734 | D08870 | hsa7153 | D10787 |
| hsa5140 | D01771 | hsa1375 | D05442 | hsa9759 | D08870 | hsa7155 | D10787 |
| hsa5141 | D01771 | hsa1376 | D05442 | hsa7153 | D08871 | hsa5290 | D10797 |
| hsa5142 | D01771 | hsa2064 | D05446 | hsa7155 | D08871 | hsa5293 | D10797 |
| hsa5143 | D01771 | hsa5742 | D05451 | hsa2159 | D08873 | hsa5290 | D10798 |
| hsa5144 | D01771 | hsa5743 | D05451 | hsa2260 | D08878 | hsa5293 | D10798 |
| hsa5150 | D01771 | hsa79001 | D05457 | hsa3791 | D08878 | hsa2322 | D10800 |
| hsa5151 | D01771 | hsa4128 | D05458 | hsa5693 | D08880 | hsa558 | D10800 |
| hsa5153 | D01771 | hsa4129 | D05458 | hsa2321 | D08881 | hsa7298 | D10803 |
| hsa8622 | D01771 | hsa4128 | D05459 | hsa2324 | D08881 | hsa55512 | D10820 |
| hsa240 | D01773 | hsa4129 | D05459 | hsa3791 | D08881 | hsa55627 | D10820 |
| hsa5141 | D01783 | hsa5141 | D05474 | hsa2321 | D08883 | hsa6609 | D10820 |
| hsa5142 | D01783 | hsa5142 | D05474 | hsa2324 | D08883 | hsa6610 | D10820 |
| hsa5143 | D01783 | hsa5143 | D05474 | hsa3791 | D08883 | hsa1312 | D10825 |
| hsa5144 | D01783 | hsa5144 | D05474 | hsa2475 | D08900 | hsa4129 | D10829 |
| hsa7298 | D01784 | hsa26 | D05479 | hsa495 | D08903 | hsa3818 | D10845 |
| hsa5742 | D01788 | hsa4842 | D05479 | hsa496 | D08903 | hsa715 | D10845 |
| hsa5743 | D01788 | hsa4843 | D05479 | hsa2261 | D08907 | hsa716 | D10845 |
| hsa2990 | D01805 | hsa4846 | D05479 | hsa2321 | D08907 | hsa1956 | D10858 |
| hsa2977 | D01810 | hsa5530 | D05480 | hsa2322 | D08907 | hsa1956 | D10859 |
| hsa2982 | D01810 | hsa5532 | D05480 | hsa2324 | D08907 | hsa3791 | D10862 |
| hsa2983 | D01810 | hsa5533 | D05480 | hsa3791 | D08907 | hsa4233 | D10862 |
| hsa5742 | D01811 | hsa6916 | D05502 | hsa3815 | D08907 | hsa7010 | D10862 |
| hsa5743 | D01811 | hsa5742 | D05511 | hsa5159 | D08907 | hsa238 | D10866 |
| hsa11238 | D01822 | hsa5743 | D05511 | hsa2159 | D08913 | hsa5290 | D10867 |
| hsa23632 | D01822 | hsa5742 | D05512 | hsa2950 | D08917 | hsa5293 | D10867 |
| hsa377677 | D01822 | hsa5743 | D05512 | hsa2321 | D08947 | hsa3716 | D10871 |
| hsa759 | D01822 | hsa5742 | D05513 | hsa2324 | D08947 | hsa3716 | D10872 |
| hsa760 | D01822 | hsa5743 | D05513 | hsa3791 | D08947 | hsa5141 | D10873 |
| hsa761 | D01822 | hsa7153 | D05522 | hsa3815 | D08947 | hsa5142 | D10873 |
| hsa762 | D01822 | hsa7155 | D05522 | hsa5156 | D08947 | hsa5143 | D10873 |
| hsa763 | D01822 | hsa1719 | D05589 | hsa5159 | D08947 | hsa5144 | D10873 |
| hsa765 | D01822 | hsa200895 | D05589 | hsa5979 | D08947 | hsa1019 | D10883 |
| hsa766 | D01822 | hsa43 | D05590 | hsa1956 | D08950 | hsa1021 | D10883 |
| hsa767 | D01822 | hsa5742 | D05598 | hsa2064 | D08950 | hsa695 | D10893 |
| hsa768 | D01822 | hsa5743 | D05598 | hsa2066 | D08950 | hsa1956 | D10898 |
| hsa771 | D01822 | hsa3643 | D05622 | hsa25 | D08953 | hsa2064 | D10898 |
| hsa5742 | D01823 | hsa6916 | D05727 | hsa3815 | D08953 | hsa2066 | D10898 |
| hsa5743 | D01823 | hsa7941 | D05728 | hsa5156 | D08953 | hsa3418 | D10901 |
| hsa6093 | D01840 | hsa5141 | D05744 | hsa5159 | D08953 | hsa10013 | D10918 |
| hsa9475 | D01840 | hsa5142 | D05744 | hsa1513 | D08955 | hsa5293 | D10924 |
| hsa5742 | D01841 | hsa5143 | D05744 | hsa1432 | D08963 | hsa238 | D10926 |
| hsa5743 | D01841 | hsa5144 | D05744 | hsa2645 | D08970 | hsa4914 | D10926 |
| hsa231 | D01842 | hsa5289 | D05784 | hsa834 | D08978 | hsa4915 | D10926 |
| hsa2159 | D01844 | hsa5579 | D05784 | hsa7153 | D08981 | hsa4916 | D10926 |
| hsa1806 | D01846 | hsa5580 | D05784 | hsa7155 | D08981 | hsa2260 | D10927 |
| hsa3156 | D01862 | hsa5581 | D05784 | hsa1803 | D08996 | hsa2261 | D10927 |
| hsa5742 | D01866 | hsa5289 | D05785 | hsa5663 | D09010 | hsa2263 | D10927 |
| hsa5743 | D01866 | hsa5579 | D05785 | hsa4842 | D09018 | hsa2264 | D10927 |
| hsa7153 | D01885 | hsa5580 | D05785 | hsa4843 | D09018 | hsa2065 | D10943 |
| hsa7155 | D01885 | hsa5581 | D05785 | hsa4846 | D09018 | hsa3480 | D10943 |
| hsa4128 | D01888 | hsa2322 | D05819 | hsa5141 | D09020 | hsa3716 | D10944 |
| hsa4129 | D01888 | hsa3791 | D05819 | hsa5142 | D09020 | hsa3716 | D10945 |
| hsa5139 | D01896 | hsa3815 | D05819 | hsa5143 | D09020 | hsa1956 | D10958 |
| hsa5140 | D01896 | hsa231 | D05893 | hsa5144 | D09020 | hsa1432 | D10959 |
| hsa3290 | D01899 | hsa495 | D05900 | hsa5530 | D09033 | hsa5600 | D10959 |
| hsa1636 | D01900 | hsa496 | D05900 | hsa5532 | D09033 | hsa5603 | D10959 |
| hsa6240 | D01907 | hsa495 | D05901 | hsa5533 | D09033 | hsa6300 | D10959 |
| hsa7150 | D01911 | hsa496 | D05901 | hsa5972 | D09038 | hsa4907 | D10963 |
| hsa7153 | D01911 | hsa495 | D05906 | hsa2224 | D09198 | hsa2984 | D10976 |
| hsa7155 | D01911 | hsa496 | D05906 | hsa5406 | D09208 | hsa10013 | D10977 |
| hsa3156 | D01915 | hsa43 | D05981 | hsa102 | D09320 | hsa10014 | D10977 |
| hsa1991 | D01918 | hsa2322 | D06005 | hsa6868 | D09320 | hsa3065 | D10977 |
| hsa495 | D01920 | hsa3815 | D06005 | hsa1803 | D09326 | hsa3066 | D10977 |
| hsa496 | D01920 | hsa5159 | D06005 | hsa7150 | D09327 | hsa51564 | D10977 |
| hsa2224 | D01968 | hsa5243 | D06008 | hsa3480 | D09328 | hsa55869 | D10977 |
| hsa4311 | D01970 | hsa2475 | D06068 | hsa1803 | D09333 | hsa79885 | D10977 |
| hsa476 | D01972 | hsa5742 | D06072 | hsa1803 | D09334 | hsa83933 | D10977 |
| hsa477 | D01972 | hsa5743 | D06072 | hsa10013 | D09338 | hsa8841 | D10977 |
| hsa478 | D01972 | hsa5742 | D06073 | hsa10014 | D09338 | hsa9734 | D10977 |
| hsa480 | D01972 | hsa5743 | D06073 | hsa3065 | D09338 | hsa9759 | D10977 |
| hsa5743 | D01974 | hsa1636 | D06076 | hsa3066 | D09338 | hsa1019 | D10979 |
| hsa1956 | D01977 | hsa10846 | D06103 | hsa51564 | D09338 | hsa1021 | D10979 |
| hsa2147 | D01981 | hsa27115 | D06103 | hsa55869 | D09338 | hsa10038 | D10982 |
| hsa495 | D01984 | hsa50940 | D06103 | hsa79885 | D09338 | hsa10039 | D10982 |
| hsa496 | D01984 | hsa5136 | D06103 | hsa83933 | D09338 | hsa142 | D10982 |
| hsa8654 | D02008 | hsa5137 | D06103 | hsa8841 | D09338 | hsa143 | D10982 |
| hsa10846 | D02017 | hsa5138 | D06103 | hsa9734 | D09338 | hsa5530 | D10983 |
| hsa27115 | D02017 | hsa5139 | D06103 | hsa9759 | D09338 | hsa5532 | D10983 |
| hsa50940 | D02017 | hsa5140 | D06103 | hsa495 | D09339 | hsa5533 | D10983 |
| hsa5136 | D02017 | hsa5141 | D06103 | hsa496 | D09339 | hsa7150 | D10985 |
| hsa5137 | D02017 | hsa5142 | D06103 | hsa3480 | D09345 | hsa4217 | D10988 |
| hsa5138 | D02017 | hsa5143 | D06103 | hsa6850 | D09347 | hsa10013 | D10993 |
| hsa5139 | D02017 | hsa5144 | D06103 | hsa6850 | D09348 | hsa10014 | D10993 |
| hsa5140 | D02017 | hsa5150 | D06103 | hsa2984 | D09355 | hsa3065 | D10993 |
| hsa5141 | D02017 | hsa5151 | D06103 | hsa3065 | D09357 | hsa3066 | D10993 |
| hsa5142 | D02017 | hsa5153 | D06103 | hsa3066 | D09357 | hsa51564 | D10993 |
| hsa5143 | D02017 | hsa8622 | D06103 | hsa8841 | D09357 | hsa55869 | D10993 |
| hsa5144 | D02017 | hsa10846 | D06104 | hsa3791 | D09371 | hsa79885 | D10993 |
| hsa5150 | D02017 | hsa27115 | D06104 | hsa5663 | D09377 | hsa83933 | D10993 |
| hsa5151 | D02017 | hsa50940 | D06104 | hsa1432 | D09386 | hsa8841 | D10993 |
| hsa5153 | D02017 | hsa5136 | D06104 | hsa2159 | D09546 | hsa9734 | D10993 |
| hsa8622 | D02017 | hsa5137 | D06104 | hsa1803 | D09566 | hsa9759 | D10993 |
| hsa3290 | D02028 | hsa5138 | D06104 | hsa5742 | D09568 | hsa3716 | D10994 |
| hsa5139 | D02042 | hsa5139 | D06104 | hsa5743 | D09568 | hsa3716 | D10995 |
| hsa5140 | D02042 | hsa5140 | D06104 | hsa2977 | D09572 | hsa6121 | D11008 |
| hsa3990 | D02047 | hsa5141 | D06104 | hsa2982 | D09572 | hsa5290 | D11011 |
| hsa4023 | D02047 | hsa5142 | D06104 | hsa2983 | D09572 | hsa1803 | D11023 |
| hsa43 | D02068 | hsa5143 | D06104 | hsa2260 | D09589 | hsa2159 | D11029 |
| hsa5139 | D02084 | hsa5144 | D06104 | hsa3791 | D09589 | hsa6093 | D11030 |
| hsa5140 | D02084 | hsa5150 | D06104 | hsa768 | D09593 | hsa9475 | D11030 |
| hsa5139 | D02085 | hsa5151 | D06104 | hsa1432 | D09602 | hsa6093 | D11031 |
| hsa5140 | D02085 | hsa5153 | D06104 | hsa5600 | D09602 | hsa9475 | D11031 |
| hsa5742 | D02110 | hsa8622 | D06104 | hsa5603 | D09602 | hsa5594 | D11038 |
| hsa5743 | D02110 | hsa3251 | D06109 | hsa6300 | D09602 | hsa5595 | D11038 |
| hsa1719 | D02115 | hsa3614 | D06130 | hsa1432 | D09603 | hsa1080 | D11041 |
| hsa200895 | D02115 | hsa3615 | D06130 | hsa5600 | D09603 | hsa3418 | D11044 |
| hsa7153 | D02166 | hsa10846 | D06132 | hsa5603 | D09603 | hsa3716 | D11046 |
| hsa7155 | D02166 | hsa27115 | D06132 | hsa6300 | D09603 | hsa3717 | D11046 |
| hsa7150 | D02168 | hsa50940 | D06132 | hsa1017 | D09604 | hsa3718 | D11046 |
| hsa43 | D02173 | hsa5136 | D06132 | hsa1020 | D09604 | hsa3716 | D11048 |
| hsa43 | D02193 | hsa5137 | D06132 | hsa1025 | D09604 | hsa169355 | D11049 |
| hsa590 | D02193 | hsa5138 | D06132 | hsa983 | D09604 | hsa3620 | D11049 |
| hsa1800 | D02194 | hsa5139 | D06132 | hsa2548 | D09605 | hsa2977 | D11051 |
| hsa5562 | D02206 | hsa5140 | D06132 | hsa2548 | D09606 | hsa2982 | D11051 |
| hsa5563 | D02206 | hsa5141 | D06132 | hsa3791 | D09618 | hsa2983 | D11051 |
| hsa7153 | D02214 | hsa5142 | D06132 | hsa4233 | D09618 | hsa673 | D11053 |
| hsa7155 | D02214 | hsa5143 | D06132 | hsa8654 | D09621 | hsa1585 | D11061 |
| hsa10846 | D02218 | hsa5144 | D06132 | hsa8654 | D09622 | hsa695 | D11070 |
| hsa27115 | D02218 | hsa5150 | D06132 | hsa1803 | D09625 | hsa4233 | D11073 |
| hsa50940 | D02218 | hsa5151 | D06132 | hsa7015 | D09629 | hsa10000 | D11074 |
| hsa5136 | D02218 | hsa5153 | D06132 | hsa7015 | D09630 | hsa207 | D11074 |
| hsa5137 | D02218 | hsa8622 | D06132 | hsa768 | D09632 | hsa208 | D11074 |
| hsa5138 | D02218 | hsa2159 | D06142 | hsa2321 | D09635 | hsa112398 | D11078 |
| hsa5139 | D02218 | hsa1719 | D06238 | hsa2324 | D09635 | hsa112399 | D11078 |
| hsa5140 | D02218 | hsa200895 | D06238 | hsa3791 | D09635 | hsa54583 | D11078 |
| hsa5141 | D02218 | hsa1719 | D06239 | hsa5156 | D09635 | hsa7498 | D11081 |
| hsa5142 | D02218 |  |  |  |  |  |  |

Table2. The overall of drug-target interactions for ion channels class on new dataset 1

| **drug** | **target** | **drug** | **target** | **drug** | **target** | **drug** | **target** |
| --- | --- | --- | --- | --- | --- | --- | --- |
| hsa2554 | D00058 | hsa2556 | D00701 | hsa6328 | D02098 | hsa170572 | D05685 |
| hsa2555 | D00058 | hsa2557 | D00701 | hsa6329 | D02098 | hsa200909 | D05685 |
| hsa2556 | D00058 | hsa2558 | D00701 | hsa6331 | D02098 | hsa285242 | D05685 |
| hsa2557 | D00058 | hsa2559 | D00701 | hsa6334 | D02098 | hsa3359 | D05685 |
| hsa2558 | D00058 | hsa2560 | D00701 | hsa6335 | D02098 | hsa9177 | D05685 |
| hsa2559 | D00058 | hsa2561 | D00701 | hsa6336 | D02098 | hsa1134 | D05703 |
| hsa2560 | D00058 | hsa2562 | D00701 | hsa2902 | D02102 | hsa1140 | D05703 |
| hsa2561 | D00058 | hsa2563 | D00701 | hsa2903 | D02102 | hsa1144 | D05703 |
| hsa2562 | D00058 | hsa2564 | D00701 | hsa2904 | D02102 | hsa1145 | D05703 |
| hsa2563 | D00058 | hsa2565 | D00701 | hsa2905 | D02102 | hsa1146 | D05703 |
| hsa2564 | D00058 | hsa2566 | D00701 | hsa2906 | D02102 | hsa2902 | D05714 |
| hsa2565 | D00058 | hsa2567 | D00701 | hsa11280 | D02103 | hsa2903 | D05714 |
| hsa2566 | D00058 | hsa2568 | D00701 | hsa6323 | D02103 | hsa2904 | D05714 |
| hsa2567 | D00058 | hsa55879 | D00701 | hsa6326 | D02103 | hsa2905 | D05714 |
| hsa2568 | D00058 | hsa2554 | D00706 | hsa6328 | D02103 | hsa2906 | D05714 |
| hsa55879 | D00058 | hsa2555 | D00706 | hsa6329 | D02103 | hsa11280 | D05775 |
| hsa79054 | D00064 | hsa2556 | D00706 | hsa6331 | D02103 | hsa6323 | D05775 |
| hsa8989 | D00064 | hsa2557 | D00706 | hsa6334 | D02103 | hsa6326 | D05775 |
| hsa11280 | D00110 | hsa2558 | D00706 | hsa6335 | D02103 | hsa6328 | D05775 |
| hsa3757 | D00110 | hsa2559 | D00706 | hsa6336 | D02103 | hsa6329 | D05775 |
| hsa6323 | D00110 | hsa2560 | D00706 | hsa170572 | D02130 | hsa6331 | D05775 |
| hsa6326 | D00110 | hsa2561 | D00706 | hsa200909 | D02130 | hsa6334 | D05775 |
| hsa6328 | D00110 | hsa2562 | D00706 | hsa285242 | D02130 | hsa6335 | D05775 |
| hsa6329 | D00110 | hsa2563 | D00706 | hsa3359 | D02130 | hsa6336 | D05775 |
| hsa6331 | D00110 | hsa2564 | D00706 | hsa9177 | D02130 | hsa1136 | D06016 |
| hsa6334 | D00110 | hsa2565 | D00706 | hsa11280 | D02182 | hsa1137 | D06016 |
| hsa6335 | D00110 | hsa2566 | D00706 | hsa3757 | D02182 | hsa1141 | D06016 |
| hsa6336 | D00110 | hsa2567 | D00706 | hsa6323 | D02182 | hsa1143 | D06016 |
| hsa11280 | D00199 | hsa2568 | D00706 | hsa6326 | D02182 | hsa2554 | D06106 |
| hsa6323 | D00199 | hsa55879 | D00706 | hsa6328 | D02182 | hsa2555 | D06106 |
| hsa6326 | D00199 | hsa8911 | D00710 | hsa6329 | D02182 | hsa2556 | D06106 |
| hsa6328 | D00199 | hsa8912 | D00710 | hsa6331 | D02182 | hsa2557 | D06106 |
| hsa6329 | D00199 | hsa8913 | D00710 | hsa6334 | D02182 | hsa2558 | D06106 |
| hsa6331 | D00199 | hsa2902 | D00711 | hsa6335 | D02182 | hsa2559 | D06106 |
| hsa6334 | D00199 | hsa2903 | D00711 | hsa6336 | D02182 | hsa2560 | D06106 |
| hsa6335 | D00199 | hsa2904 | D00711 | hsa1134 | D02202 | hsa2561 | D06106 |
| hsa6336 | D00199 | hsa2905 | D00711 | hsa1135 | D02202 | hsa2562 | D06106 |
| hsa2554 | D00225 | hsa2906 | D00711 | hsa1136 | D02202 | hsa2563 | D06106 |
| hsa2555 | D00225 | hsa2554 | D00712 | hsa1137 | D02202 | hsa2564 | D06106 |
| hsa2556 | D00225 | hsa2554 | D00713 | hsa1138 | D02202 | hsa2565 | D06106 |
| hsa2557 | D00225 | hsa2555 | D00713 | hsa1139 | D02202 | hsa2566 | D06106 |
| hsa2558 | D00225 | hsa2556 | D00713 | hsa1140 | D02202 | hsa2567 | D06106 |
| hsa2559 | D00225 | hsa2557 | D00713 | hsa1141 | D02202 | hsa2568 | D06106 |
| hsa2560 | D00225 | hsa2558 | D00713 | hsa1142 | D02202 | hsa55879 | D06106 |
| hsa2561 | D00225 | hsa2559 | D00713 | hsa1143 | D02202 | hsa2902 | D06146 |
| hsa2562 | D00225 | hsa2560 | D00713 | hsa1144 | D02202 | hsa2903 | D06146 |
| hsa2563 | D00225 | hsa2561 | D00713 | hsa1145 | D02202 | hsa2904 | D06146 |
| hsa2564 | D00225 | hsa2562 | D00713 | hsa1146 | D02202 | hsa2905 | D06146 |
| hsa2565 | D00225 | hsa2563 | D00713 | hsa55584 | D02202 | hsa2906 | D06146 |
| hsa2566 | D00225 | hsa2564 | D00713 | hsa57053 | D02202 | hsa11280 | D06172 |
| hsa2567 | D00225 | hsa2565 | D00713 | hsa8973 | D02202 | hsa6323 | D06172 |
| hsa2568 | D00225 | hsa2566 | D00713 | hsa1135 | D02204 | hsa6326 | D06172 |
| hsa55879 | D00225 | hsa2567 | D00713 | hsa1136 | D02204 | hsa6328 | D06172 |
| hsa7442 | D00250 | hsa2568 | D00713 | hsa1137 | D02204 | hsa6329 | D06172 |
| hsa11280 | D00252 | hsa55879 | D00713 | hsa1138 | D02204 | hsa6331 | D06172 |
| hsa6323 | D00252 | hsa2554 | D00714 | hsa1139 | D02204 | hsa6334 | D06172 |
| hsa6326 | D00252 | hsa2555 | D00714 | hsa1141 | D02204 | hsa6335 | D06172 |
| hsa6328 | D00252 | hsa2556 | D00714 | hsa1142 | D02204 | hsa6336 | D06172 |
| hsa6329 | D00252 | hsa2557 | D00714 | hsa1143 | D02204 | hsa3767 | D06177 |
| hsa6331 | D00252 | hsa2558 | D00714 | hsa55584 | D02204 | hsa2904 | D06204 |
| hsa6334 | D00252 | hsa2559 | D00714 | hsa57053 | D02204 | hsa3757 | D06206 |
| hsa6335 | D00252 | hsa2560 | D00714 | hsa8973 | D02204 | hsa170572 | D06253 |
| hsa6336 | D00252 | hsa2561 | D00714 | hsa1135 | D02207 | hsa200909 | D06253 |
| hsa2554 | D00267 | hsa2562 | D00714 | hsa1136 | D02207 | hsa285242 | D06253 |
| hsa2555 | D00267 | hsa2563 | D00714 | hsa1137 | D02207 | hsa3359 | D06253 |
| hsa2556 | D00267 | hsa2564 | D00714 | hsa1138 | D02207 | hsa9177 | D06253 |
| hsa2557 | D00267 | hsa2565 | D00714 | hsa1139 | D02207 | hsa1137 | D06282 |
| hsa2558 | D00267 | hsa2566 | D00714 | hsa1141 | D02207 | hsa1141 | D06282 |
| hsa2559 | D00267 | hsa2567 | D00714 | hsa1142 | D02207 | hsa170572 | D06353 |
| hsa2560 | D00267 | hsa2568 | D00714 | hsa1143 | D02207 | hsa200909 | D06353 |
| hsa2561 | D00267 | hsa55879 | D00714 | hsa55584 | D02207 | hsa285242 | D06353 |
| hsa2562 | D00267 | hsa170572 | D00725 | hsa57053 | D02207 | hsa3359 | D06353 |
| hsa2563 | D00267 | hsa200909 | D00725 | hsa8973 | D02207 | hsa9177 | D06353 |
| hsa2564 | D00267 | hsa285242 | D00725 | hsa11280 | D02220 | hsa774 | D06363 |
| hsa2565 | D00267 | hsa3359 | D00725 | hsa6323 | D02220 | hsa7442 | D06388 |
| hsa2566 | D00267 | hsa9177 | D00725 | hsa6326 | D02220 | hsa170572 | D06396 |
| hsa2567 | D00267 | hsa3750 | D00730 | hsa6328 | D02220 | hsa200909 | D06396 |
| hsa2568 | D00267 | hsa3751 | D00730 | hsa6329 | D02220 | hsa285242 | D06396 |
| hsa55879 | D00267 | hsa3752 | D00730 | hsa6331 | D02220 | hsa3359 | D06396 |
| hsa3767 | D00271 | hsa11280 | D00732 | hsa6334 | D02220 | hsa9177 | D06396 |
| hsa3359 | D00274 | hsa6323 | D00732 | hsa6335 | D02220 | hsa11280 | D06517 |
| hsa2554 | D00280 | hsa6326 | D00732 | hsa6336 | D02220 | hsa6323 | D06517 |
| hsa2555 | D00280 | hsa6328 | D00732 | hsa11280 | D02239 | hsa6326 | D06517 |
| hsa2556 | D00280 | hsa6329 | D00732 | hsa6323 | D02239 | hsa6328 | D06517 |
| hsa2557 | D00280 | hsa6331 | D00732 | hsa6326 | D02239 | hsa6329 | D06517 |
| hsa2558 | D00280 | hsa6334 | D00732 | hsa6328 | D02239 | hsa6331 | D06517 |
| hsa2559 | D00280 | hsa6335 | D00732 | hsa6329 | D02239 | hsa6334 | D06517 |
| hsa2560 | D00280 | hsa6336 | D00732 | hsa6331 | D02239 | hsa6335 | D06517 |
| hsa2561 | D00280 | hsa11280 | D00733 | hsa6334 | D02239 | hsa6336 | D06517 |
| hsa2562 | D00280 | hsa6323 | D00733 | hsa6335 | D02239 | hsa3783 | D06640 |
| hsa2563 | D00280 | hsa6326 | D00733 | hsa6336 | D02239 | hsa3736 | D06652 |
| hsa2564 | D00280 | hsa6328 | D00733 | hsa2554 | D02252 | hsa3736 | D06653 |
| hsa2565 | D00280 | hsa6329 | D00733 | hsa2555 | D02252 | hsa2890 | D06656 |
| hsa2566 | D00280 | hsa6331 | D00733 | hsa2556 | D02252 | hsa2891 | D06656 |
| hsa2567 | D00280 | hsa6334 | D00733 | hsa2557 | D02252 | hsa2892 | D06656 |
| hsa2568 | D00280 | hsa6335 | D00733 | hsa2558 | D02252 | hsa2893 | D06656 |
| hsa55879 | D00280 | hsa6336 | D00733 | hsa2559 | D02252 | hsa2897 | D06656 |
| hsa170572 | D00283 | hsa6323 | D00737 | hsa2560 | D02252 | hsa2898 | D06656 |
| hsa200909 | D00283 | hsa6326 | D00737 | hsa2561 | D02252 | hsa2899 | D06656 |
| hsa285242 | D00283 | hsa6328 | D00737 | hsa2562 | D02252 | hsa2900 | D06656 |
| hsa3359 | D00283 | hsa6329 | D00737 | hsa2563 | D02252 | hsa2901 | D06656 |
| hsa9177 | D00283 | hsa6331 | D00737 | hsa2564 | D02252 | hsa3741 | D06665 |
| hsa2554 | D00293 | hsa6334 | D00737 | hsa2565 | D02252 | hsa3752 | D06665 |
| hsa2555 | D00293 | hsa6335 | D00737 | hsa2566 | D02252 | hsa6331 | D06665 |
| hsa2556 | D00293 | hsa6336 | D00737 | hsa2567 | D02252 | hsa2554 | D06887 |
| hsa2557 | D00293 | hsa11280 | D00738 | hsa2568 | D02252 | hsa2555 | D06887 |
| hsa2558 | D00293 | hsa6323 | D00738 | hsa55879 | D02252 | hsa2556 | D06887 |
| hsa2559 | D00293 | hsa6326 | D00738 | hsa2554 | D02253 | hsa2557 | D06887 |
| hsa2560 | D00293 | hsa6328 | D00738 | hsa2555 | D02253 | hsa2558 | D06887 |
| hsa2561 | D00293 | hsa6329 | D00738 | hsa2556 | D02253 | hsa2559 | D06887 |
| hsa2562 | D00293 | hsa6331 | D00738 | hsa2557 | D02253 | hsa2560 | D06887 |
| hsa2563 | D00293 | hsa6334 | D00738 | hsa2558 | D02253 | hsa2561 | D06887 |
| hsa2564 | D00293 | hsa6335 | D00738 | hsa2559 | D02253 | hsa2562 | D06887 |
| hsa2565 | D00293 | hsa6336 | D00738 | hsa2560 | D02253 | hsa2563 | D06887 |
| hsa2566 | D00293 | hsa11280 | D00739 | hsa2561 | D02253 | hsa2564 | D06887 |
| hsa2567 | D00293 | hsa6323 | D00739 | hsa2562 | D02253 | hsa2565 | D06887 |
| hsa2568 | D00293 | hsa6326 | D00739 | hsa2563 | D02253 | hsa2566 | D06887 |
| hsa55879 | D00293 | hsa6328 | D00739 | hsa2564 | D02253 | hsa2567 | D06887 |
| hsa3767 | D00294 | hsa6329 | D00739 | hsa2565 | D02253 | hsa2568 | D06887 |
| hsa11280 | D00303 | hsa6331 | D00739 | hsa2566 | D02253 | hsa55879 | D06887 |
| hsa6323 | D00303 | hsa6334 | D00739 | hsa2567 | D02253 | hsa2902 | D07058 |
| hsa6326 | D00303 | hsa6335 | D00739 | hsa2568 | D02253 | hsa2903 | D07058 |
| hsa6328 | D00303 | hsa6336 | D00739 | hsa55879 | D02253 | hsa2904 | D07058 |
| hsa6329 | D00303 | hsa11280 | D00740 | hsa11280 | D02272 | hsa2905 | D07058 |
| hsa6331 | D00303 | hsa6323 | D00740 | hsa6323 | D02272 | hsa2906 | D07058 |
| hsa6334 | D00303 | hsa6326 | D00740 | hsa6326 | D02272 | hsa779 | D07094 |
| hsa6335 | D00303 | hsa6328 | D00740 | hsa6328 | D02272 | hsa170572 | D07129 |
| hsa6336 | D00303 | hsa6329 | D00740 | hsa6329 | D02272 | hsa200909 | D07129 |
| hsa8911 | D00304 | hsa6331 | D00740 | hsa6331 | D02272 | hsa285242 | D07129 |
| hsa8912 | D00304 | hsa6334 | D00740 | hsa6334 | D02272 | hsa3359 | D07129 |
| hsa8913 | D00304 | hsa6335 | D00740 | hsa6335 | D02272 | hsa9177 | D07129 |
| hsa2554 | D00311 | hsa6336 | D00740 | hsa6336 | D02272 | hsa10021 | D07165 |
| hsa2555 | D00311 | hsa11280 | D00741 | hsa1134 | D02275 | hsa348980 | D07165 |
| hsa2556 | D00311 | hsa6323 | D00741 | hsa1140 | D02275 | hsa57657 | D07165 |
| hsa2557 | D00311 | hsa6326 | D00741 | hsa1144 | D02275 | hsa610 | D07165 |
| hsa2558 | D00311 | hsa6328 | D00741 | hsa1145 | D02275 | hsa170572 | D07175 |
| hsa2559 | D00311 | hsa6329 | D00741 | hsa1146 | D02275 | hsa200909 | D07175 |
| hsa2560 | D00311 | hsa6331 | D00741 | hsa2554 | D02283 | hsa285242 | D07175 |
| hsa2561 | D00311 | hsa6334 | D00741 | hsa2555 | D02283 | hsa3359 | D07175 |
| hsa2562 | D00311 | hsa6335 | D00741 | hsa2556 | D02283 | hsa9177 | D07175 |
| hsa2563 | D00311 | hsa6336 | D00741 | hsa2557 | D02283 | hsa775 | D07185 |
| hsa2564 | D00311 | hsa1135 | D00758 | hsa2558 | D02283 | hsa776 | D07185 |
| hsa2565 | D00311 | hsa1136 | D00758 | hsa2559 | D02283 | hsa778 | D07185 |
| hsa2566 | D00311 | hsa1137 | D00758 | hsa2560 | D02283 | hsa779 | D07185 |
| hsa2567 | D00311 | hsa1138 | D00758 | hsa2561 | D02283 | hsa1135 | D07272 |
| hsa2568 | D00311 | hsa1139 | D00758 | hsa2562 | D02283 | hsa1136 | D07272 |
| hsa55879 | D00311 | hsa1141 | D00758 | hsa2563 | D02283 | hsa1137 | D07272 |
| hsa775 | D00319 | hsa1142 | D00758 | hsa2564 | D02283 | hsa1138 | D07272 |
| hsa776 | D00319 | hsa1143 | D00758 | hsa2565 | D02283 | hsa1139 | D07272 |
| hsa778 | D00319 | hsa55584 | D00758 | hsa2566 | D02283 | hsa1141 | D07272 |
| hsa779 | D00319 | hsa57053 | D00758 | hsa2567 | D02283 | hsa1142 | D07272 |
| hsa2554 | D00329 | hsa8973 | D00758 | hsa2568 | D02283 | hsa1143 | D07272 |
| hsa2555 | D00329 | hsa1135 | D00759 | hsa55879 | D02283 | hsa55584 | D07272 |
| hsa2556 | D00329 | hsa1136 | D00759 | hsa775 | D02356 | hsa57053 | D07272 |
| hsa2557 | D00329 | hsa1137 | D00759 | hsa776 | D02356 | hsa8973 | D07272 |
| hsa2558 | D00329 | hsa1138 | D00759 | hsa778 | D02356 | hsa1135 | D07273 |
| hsa2559 | D00329 | hsa1139 | D00759 | hsa779 | D02356 | hsa1136 | D07273 |
| hsa2560 | D00329 | hsa1141 | D00759 | hsa1134 | D02364 | hsa1137 | D07273 |
| hsa2561 | D00329 | hsa1142 | D00759 | hsa1135 | D02364 | hsa1138 | D07273 |
| hsa2562 | D00329 | hsa1143 | D00759 | hsa1136 | D02364 | hsa1139 | D07273 |
| hsa2563 | D00329 | hsa55584 | D00759 | hsa1137 | D02364 | hsa1141 | D07273 |
| hsa2564 | D00329 | hsa57053 | D00759 | hsa1138 | D02364 | hsa1142 | D07273 |
| hsa2565 | D00329 | hsa8973 | D00759 | hsa1139 | D02364 | hsa1143 | D07273 |
| hsa2566 | D00329 | hsa1135 | D00760 | hsa1140 | D02364 | hsa55584 | D07273 |
| hsa2567 | D00329 | hsa1136 | D00760 | hsa1141 | D02364 | hsa57053 | D07273 |
| hsa2568 | D00329 | hsa1137 | D00760 | hsa1142 | D02364 | hsa8973 | D07273 |
| hsa55879 | D00329 | hsa1138 | D00760 | hsa1143 | D02364 | hsa2554 | D07298 |
| hsa55799 | D00332 | hsa1139 | D00760 | hsa1144 | D02364 | hsa2555 | D07298 |
| hsa781 | D00332 | hsa1141 | D00760 | hsa1145 | D02364 | hsa2556 | D07298 |
| hsa9254 | D00332 | hsa1142 | D00760 | hsa1146 | D02364 | hsa2557 | D07298 |
| hsa93589 | D00332 | hsa1143 | D00760 | hsa55584 | D02364 | hsa2558 | D07298 |
| hsa3767 | D00336 | hsa55584 | D00760 | hsa57053 | D02364 | hsa2559 | D07298 |
| hsa2554 | D00338 | hsa57053 | D00760 | hsa8973 | D02364 | hsa2560 | D07298 |
| hsa2555 | D00338 | hsa8973 | D00760 | hsa3767 | D02385 | hsa2561 | D07298 |
| hsa2556 | D00338 | hsa1134 | D00761 | hsa2902 | D02410 | hsa2562 | D07298 |
| hsa2557 | D00338 | hsa1140 | D00761 | hsa2903 | D02410 | hsa2563 | D07298 |
| hsa2558 | D00338 | hsa1144 | D00761 | hsa2904 | D02410 | hsa2564 | D07298 |
| hsa2559 | D00338 | hsa1145 | D00761 | hsa2905 | D02410 | hsa2565 | D07298 |
| hsa2560 | D00338 | hsa1146 | D00761 | hsa2906 | D02410 | hsa2566 | D07298 |
| hsa2561 | D00338 | hsa1135 | D00763 | hsa11280 | D02456 | hsa2567 | D07298 |
| hsa2562 | D00338 | hsa1136 | D00763 | hsa6323 | D02456 | hsa2568 | D07298 |
| hsa2563 | D00338 | hsa1137 | D00763 | hsa6326 | D02456 | hsa55879 | D07298 |
| hsa2564 | D00338 | hsa1138 | D00763 | hsa6328 | D02456 | hsa6328 | D07299 |
| hsa2565 | D00338 | hsa1139 | D00763 | hsa6329 | D02456 | hsa6335 | D07299 |
| hsa2566 | D00338 | hsa1141 | D00763 | hsa6331 | D02456 | hsa6336 | D07299 |
| hsa2567 | D00338 | hsa1142 | D00763 | hsa6334 | D02456 | hsa2902 | D07306 |
| hsa2568 | D00338 | hsa1143 | D00763 | hsa6335 | D02456 | hsa2903 | D07306 |
| hsa55879 | D00338 | hsa55584 | D00763 | hsa6336 | D02456 | hsa2904 | D07306 |
| hsa775 | D00349 | hsa57053 | D00763 | hsa3752 | D02537 | hsa2905 | D07306 |
| hsa776 | D00349 | hsa8973 | D00763 | hsa3757 | D02537 | hsa2906 | D07306 |
| hsa778 | D00349 | hsa1135 | D00764 | hsa3760 | D02537 | hsa2554 | D07326 |
| hsa779 | D00349 | hsa1136 | D00764 | hsa3762 | D02537 | hsa2555 | D07326 |
| hsa11280 | D00354 | hsa1137 | D00764 | hsa3784 | D02537 | hsa2556 | D07326 |
| hsa6323 | D00354 | hsa1138 | D00764 | hsa775 | D02537 | hsa2557 | D07326 |
| hsa6326 | D00354 | hsa1139 | D00764 | hsa776 | D02537 | hsa2558 | D07326 |
| hsa6328 | D00354 | hsa1141 | D00764 | hsa778 | D02537 | hsa2559 | D07326 |
| hsa6329 | D00354 | hsa1142 | D00764 | hsa779 | D02537 | hsa2560 | D07326 |
| hsa6331 | D00354 | hsa1143 | D00764 | hsa2554 | D02594 | hsa2561 | D07326 |
| hsa6334 | D00354 | hsa55584 | D00764 | hsa2555 | D02594 | hsa2562 | D07326 |
| hsa6335 | D00354 | hsa57053 | D00764 | hsa2556 | D02594 | hsa2563 | D07326 |
| hsa6336 | D00354 | hsa8973 | D00764 | hsa2557 | D02594 | hsa2564 | D07326 |
| hsa11280 | D00358 | hsa1135 | D00765 | hsa2558 | D02594 | hsa2565 | D07326 |
| hsa6323 | D00358 | hsa1136 | D00765 | hsa2559 | D02594 | hsa2566 | D07326 |
| hsa6326 | D00358 | hsa1137 | D00765 | hsa2560 | D02594 | hsa2567 | D07326 |
| hsa6328 | D00358 | hsa1138 | D00765 | hsa2561 | D02594 | hsa2568 | D07326 |
| hsa6329 | D00358 | hsa1139 | D00765 | hsa2562 | D02594 | hsa55879 | D07326 |
| hsa6331 | D00358 | hsa1141 | D00765 | hsa2563 | D02594 | hsa2554 | D07328 |
| hsa6334 | D00358 | hsa1142 | D00765 | hsa2564 | D02594 | hsa2555 | D07328 |
| hsa6335 | D00358 | hsa1143 | D00765 | hsa2565 | D02594 | hsa2556 | D07328 |
| hsa6336 | D00358 | hsa55584 | D00765 | hsa2566 | D02594 | hsa2557 | D07328 |
| hsa2554 | D00365 | hsa57053 | D00765 | hsa2567 | D02594 | hsa2558 | D07328 |
| hsa2555 | D00365 | hsa8973 | D00765 | hsa2568 | D02594 | hsa2559 | D07328 |
| hsa2556 | D00365 | hsa1134 | D00766 | hsa55879 | D02594 | hsa2560 | D07328 |
| hsa2557 | D00365 | hsa1140 | D00766 | hsa116443 | D02599 | hsa2561 | D07328 |
| hsa2558 | D00365 | hsa1144 | D00766 | hsa116444 | D02599 | hsa2562 | D07328 |
| hsa2559 | D00365 | hsa1145 | D00766 | hsa2902 | D02599 | hsa2563 | D07328 |
| hsa2560 | D00365 | hsa1146 | D00766 | hsa2903 | D02599 | hsa2564 | D07328 |
| hsa2561 | D00365 | hsa1135 | D00767 | hsa2904 | D02599 | hsa2565 | D07328 |
| hsa2562 | D00365 | hsa1136 | D00767 | hsa2905 | D02599 | hsa2566 | D07328 |
| hsa2563 | D00365 | hsa1137 | D00767 | hsa2906 | D02599 | hsa2567 | D07328 |
| hsa2564 | D00365 | hsa1138 | D00767 | hsa2554 | D02616 | hsa2568 | D07328 |
| hsa2565 | D00365 | hsa1139 | D00767 | hsa2555 | D02616 | hsa55879 | D07328 |
| hsa2566 | D00365 | hsa1141 | D00767 | hsa2556 | D02616 | hsa2554 | D07330 |
| hsa2567 | D00365 | hsa1142 | D00767 | hsa2557 | D02616 | hsa2902 | D07441 |
| hsa2568 | D00365 | hsa1143 | D00767 | hsa2558 | D02616 | hsa2903 | D07441 |
| hsa55879 | D00365 | hsa55584 | D00767 | hsa2559 | D02616 | hsa2904 | D07441 |
| hsa2554 | D00370 | hsa57053 | D00767 | hsa2560 | D02616 | hsa2905 | D07441 |
| hsa2555 | D00370 | hsa8973 | D00767 | hsa2561 | D02616 | hsa2906 | D07441 |
| hsa2556 | D00370 | hsa116443 | D00774 | hsa2562 | D02616 | hsa6337 | D07447 |
| hsa2557 | D00370 | hsa116444 | D00774 | hsa2563 | D02616 | hsa6338 | D07447 |
| hsa2558 | D00370 | hsa2902 | D00774 | hsa2564 | D02616 | hsa6340 | D07447 |
| hsa2559 | D00370 | hsa2903 | D00774 | hsa2565 | D02616 | hsa775 | D07450 |
| hsa2560 | D00370 | hsa2904 | D00774 | hsa2566 | D02616 | hsa776 | D07450 |
| hsa2561 | D00370 | hsa2905 | D00774 | hsa2567 | D02616 | hsa778 | D07450 |
| hsa2562 | D00370 | hsa2906 | D00774 | hsa2568 | D02616 | hsa779 | D07450 |
| hsa2563 | D00370 | hsa2890 | D00775 | hsa55879 | D02616 | hsa11280 | D07468 |
| hsa2564 | D00370 | hsa2891 | D00775 | hsa2554 | D02617 | hsa6323 | D07468 |
| hsa2565 | D00370 | hsa2892 | D00775 | hsa2555 | D02617 | hsa6326 | D07468 |
| hsa2566 | D00370 | hsa2893 | D00775 | hsa2556 | D02617 | hsa6328 | D07468 |
| hsa2567 | D00370 | hsa2897 | D00775 | hsa2557 | D02617 | hsa6329 | D07468 |
| hsa2568 | D00370 | hsa2898 | D00775 | hsa2558 | D02617 | hsa6331 | D07468 |
| hsa55879 | D00370 | hsa2899 | D00775 | hsa2559 | D02617 | hsa6334 | D07468 |
| hsa11280 | D00375 | hsa2900 | D00775 | hsa2560 | D02617 | hsa6335 | D07468 |
| hsa6323 | D00375 | hsa2901 | D00775 | hsa2561 | D02617 | hsa6336 | D07468 |
| hsa6326 | D00375 | hsa2902 | D00775 | hsa2562 | D02617 | hsa170572 | D07481 |
| hsa6328 | D00375 | hsa2903 | D00775 | hsa2563 | D02617 | hsa200909 | D07481 |
| hsa6329 | D00375 | hsa2904 | D00775 | hsa2564 | D02617 | hsa285242 | D07481 |
| hsa6331 | D00375 | hsa2905 | D00775 | hsa2565 | D02617 | hsa3359 | D07481 |
| hsa6334 | D00375 | hsa2906 | D00775 | hsa2566 | D02617 | hsa9177 | D07481 |
| hsa6335 | D00375 | hsa2902 | D00777 | hsa2567 | D02617 | hsa775 | D07494 |
| hsa6336 | D00375 | hsa2903 | D00777 | hsa2568 | D02617 | hsa776 | D07494 |
| hsa2554 | D00376 | hsa2904 | D00777 | hsa55879 | D02617 | hsa778 | D07494 |
| hsa2555 | D00376 | hsa2905 | D00777 | hsa2554 | D02618 | hsa779 | D07494 |
| hsa2556 | D00376 | hsa2906 | D00777 | hsa2555 | D02618 | hsa775 | D07509 |
| hsa2557 | D00376 | hsa2902 | D00848 | hsa2556 | D02618 | hsa776 | D07509 |
| hsa2558 | D00376 | hsa2903 | D00848 | hsa2557 | D02618 | hsa778 | D07509 |
| hsa2559 | D00376 | hsa2904 | D00848 | hsa2558 | D02618 | hsa779 | D07509 |
| hsa2560 | D00376 | hsa2905 | D00848 | hsa2559 | D02618 | hsa3741 | D07520 |
| hsa2561 | D00376 | hsa2906 | D00848 | hsa2560 | D02618 | hsa3752 | D07520 |
| hsa2562 | D00376 | hsa1134 | D00999 | hsa2561 | D02618 | hsa3757 | D07520 |
| hsa2563 | D00376 | hsa1135 | D00999 | hsa2562 | D02618 | hsa3760 | D07520 |
| hsa2564 | D00376 | hsa1136 | D00999 | hsa2563 | D02618 | hsa3762 | D07520 |
| hsa2565 | D00376 | hsa1137 | D00999 | hsa2564 | D02618 | hsa3764 | D07520 |
| hsa2566 | D00376 | hsa1138 | D00999 | hsa2565 | D02618 | hsa3767 | D07520 |
| hsa2567 | D00376 | hsa1139 | D00999 | hsa2566 | D02618 | hsa3768 | D07520 |
| hsa2568 | D00376 | hsa1140 | D00999 | hsa2567 | D02618 | hsa3784 | D07520 |
| hsa55879 | D00376 | hsa1141 | D00999 | hsa2568 | D02618 | hsa775 | D07520 |
| hsa3767 | D00379 | hsa1142 | D00999 | hsa55879 | D02618 | hsa776 | D07520 |
| hsa3767 | D00380 | hsa1143 | D00999 | hsa2554 | D02624 | hsa778 | D07520 |
| hsa6337 | D00386 | hsa1144 | D00999 | hsa2555 | D02624 | hsa779 | D07520 |
| hsa6338 | D00386 | hsa1145 | D00999 | hsa2556 | D02624 | hsa8911 | D07520 |
| hsa6340 | D00386 | hsa1146 | D00999 | hsa2557 | D02624 | hsa8912 | D07520 |
| hsa2554 | D00387 | hsa55584 | D00999 | hsa2558 | D02624 | hsa8913 | D07520 |
| hsa2555 | D00387 | hsa57053 | D00999 | hsa2559 | D02624 | hsa11280 | D07552 |
| hsa2556 | D00387 | hsa8973 | D00999 | hsa2560 | D02624 | hsa6323 | D07552 |
| hsa2557 | D00387 | hsa775 | D01007 | hsa2561 | D02624 | hsa6326 | D07552 |
| hsa2558 | D00387 | hsa776 | D01007 | hsa2562 | D02624 | hsa6328 | D07552 |
| hsa2559 | D00387 | hsa778 | D01007 | hsa2563 | D02624 | hsa6329 | D07552 |
| hsa2560 | D00387 | hsa779 | D01007 | hsa2564 | D02624 | hsa6331 | D07552 |
| hsa2561 | D00387 | hsa3757 | D01026 | hsa2565 | D02624 | hsa6334 | D07552 |
| hsa2562 | D00387 | hsa11280 | D01041 | hsa2566 | D02624 | hsa6335 | D07552 |
| hsa2563 | D00387 | hsa6323 | D01041 | hsa2567 | D02624 | hsa6336 | D07552 |
| hsa2564 | D00387 | hsa6326 | D01041 | hsa2568 | D02624 | hsa2902 | D07589 |
| hsa2565 | D00387 | hsa6328 | D01041 | hsa55879 | D02624 | hsa2903 | D07589 |
| hsa2566 | D00387 | hsa6329 | D01041 | hsa8911 | D02630 | hsa2904 | D07589 |
| hsa2567 | D00387 | hsa6331 | D01041 | hsa8912 | D02630 | hsa2905 | D07589 |
| hsa2568 | D00387 | hsa6334 | D01041 | hsa8913 | D02630 | hsa2906 | D07589 |
| hsa55879 | D00387 | hsa6335 | D01041 | hsa170572 | D02632 | hsa11280 | D07595 |
| hsa8911 | D00399 | hsa6336 | D01041 | hsa200909 | D02632 | hsa6323 | D07595 |
| hsa8912 | D00399 | hsa2554 | D01071 | hsa285242 | D02632 | hsa6326 | D07595 |
| hsa8913 | D00399 | hsa2555 | D01071 | hsa3359 | D02632 | hsa6328 | D07595 |
| hsa8911 | D00404 | hsa2556 | D01071 | hsa9177 | D02632 | hsa6329 | D07595 |
| hsa8912 | D00404 | hsa2557 | D01071 | hsa8911 | D02633 | hsa6331 | D07595 |
| hsa8913 | D00404 | hsa2558 | D01071 | hsa8912 | D02633 | hsa6334 | D07595 |
| hsa3767 | D00418 | hsa2559 | D01071 | hsa8913 | D02633 | hsa6335 | D07595 |
| hsa2554 | D00430 | hsa2560 | D01071 | hsa2554 | D02640 | hsa6336 | D07595 |
| hsa2555 | D00430 | hsa2561 | D01071 | hsa2555 | D02640 | hsa11280 | D07678 |
| hsa2556 | D00430 | hsa2562 | D01071 | hsa2556 | D02640 | hsa6323 | D07678 |
| hsa2557 | D00430 | hsa2563 | D01071 | hsa2557 | D02640 | hsa6326 | D07678 |
| hsa2558 | D00430 | hsa2564 | D01071 | hsa2558 | D02640 | hsa6328 | D07678 |
| hsa2559 | D00430 | hsa2565 | D01071 | hsa2559 | D02640 | hsa6329 | D07678 |
| hsa2560 | D00430 | hsa2566 | D01071 | hsa2560 | D02640 | hsa6331 | D07678 |
| hsa2561 | D00430 | hsa2567 | D01071 | hsa2561 | D02640 | hsa6334 | D07678 |
| hsa2562 | D00430 | hsa2568 | D01071 | hsa2562 | D02640 | hsa6335 | D07678 |
| hsa2563 | D00430 | hsa55879 | D01071 | hsa2563 | D02640 | hsa6336 | D07678 |
| hsa2564 | D00430 | hsa775 | D01104 | hsa2564 | D02640 | hsa2554 | D07725 |
| hsa2565 | D00430 | hsa776 | D01104 | hsa2565 | D02640 | hsa1134 | D07770 |
| hsa2566 | D00430 | hsa778 | D01104 | hsa2566 | D02640 | hsa1135 | D07770 |
| hsa2567 | D00430 | hsa779 | D01104 | hsa2567 | D02640 | hsa1136 | D07770 |
| hsa2568 | D00430 | hsa3767 | D01111 | hsa2568 | D02640 | hsa1137 | D07770 |
| hsa55879 | D00430 | hsa775 | D01145 | hsa55879 | D02640 | hsa1138 | D07770 |
| hsa775 | D00437 | hsa776 | D01145 | hsa2890 | D02696 | hsa1139 | D07770 |
| hsa776 | D00437 | hsa778 | D01145 | hsa2891 | D02696 | hsa1140 | D07770 |
| hsa778 | D00437 | hsa779 | D01145 | hsa2892 | D02696 | hsa1141 | D07770 |
| hsa779 | D00437 | hsa2554 | D01150 | hsa2893 | D02696 | hsa1142 | D07770 |
| hsa775 | D00438 | hsa775 | D01151 | hsa55799 | D02716 | hsa1143 | D07770 |
| hsa776 | D00438 | hsa776 | D01151 | hsa781 | D02716 | hsa1144 | D07770 |
| hsa778 | D00438 | hsa778 | D01151 | hsa9254 | D02716 | hsa1145 | D07770 |
| hsa779 | D00438 | hsa779 | D01151 | hsa93589 | D02716 | hsa1146 | D07770 |
| hsa170572 | D00456 | hsa11280 | D01152 | hsa11280 | D02762 | hsa55584 | D07770 |
| hsa200909 | D00456 | hsa6323 | D01152 | hsa6323 | D02762 | hsa57053 | D07770 |
| hsa285242 | D00456 | hsa6326 | D01152 | hsa6326 | D02762 | hsa8973 | D07770 |
| hsa3359 | D00456 | hsa6328 | D01152 | hsa6328 | D02762 | hsa2554 | D07784 |
| hsa9177 | D00456 | hsa6329 | D01152 | hsa6329 | D02762 | hsa2555 | D07784 |
| hsa2554 | D00457 | hsa6331 | D01152 | hsa6331 | D02762 | hsa2556 | D07784 |
| hsa2555 | D00457 | hsa6334 | D01152 | hsa6334 | D02762 | hsa2557 | D07784 |
| hsa2556 | D00457 | hsa6335 | D01152 | hsa6335 | D02762 | hsa2558 | D07784 |
| hsa2557 | D00457 | hsa6336 | D01152 | hsa6336 | D02762 | hsa2559 | D07784 |
| hsa2558 | D00457 | hsa774 | D01173 | hsa2554 | D02770 | hsa2560 | D07784 |
| hsa2559 | D00457 | hsa775 | D01173 | hsa2555 | D02770 | hsa2561 | D07784 |
| hsa2560 | D00457 | hsa776 | D01173 | hsa2556 | D02770 | hsa2562 | D07784 |
| hsa2561 | D00457 | hsa778 | D01173 | hsa2557 | D02770 | hsa2563 | D07784 |
| hsa2562 | D00457 | hsa779 | D01173 | hsa2558 | D02770 | hsa2564 | D07784 |
| hsa2563 | D00457 | hsa2554 | D01230 | hsa2559 | D02770 | hsa2565 | D07784 |
| hsa2564 | D00457 | hsa2555 | D01230 | hsa2560 | D02770 | hsa2566 | D07784 |
| hsa2565 | D00457 | hsa2556 | D01230 | hsa2561 | D02770 | hsa2567 | D07784 |
| hsa2566 | D00457 | hsa2557 | D01230 | hsa2562 | D02770 | hsa2568 | D07784 |
| hsa2567 | D00457 | hsa2558 | D01230 | hsa2563 | D02770 | hsa55879 | D07784 |
| hsa2568 | D00457 | hsa2559 | D01230 | hsa2564 | D02770 | hsa775 | D07845 |
| hsa55879 | D00457 | hsa2560 | D01230 | hsa2565 | D02770 | hsa776 | D07845 |
| hsa2554 | D00464 | hsa2561 | D01230 | hsa2566 | D02770 | hsa778 | D07845 |
| hsa2555 | D00464 | hsa2562 | D01230 | hsa2567 | D02770 | hsa779 | D07845 |
| hsa2556 | D00464 | hsa2563 | D01230 | hsa2568 | D02770 | hsa170572 | D07867 |
| hsa2557 | D00464 | hsa2564 | D01230 | hsa55879 | D02770 | hsa200909 | D07867 |
| hsa2558 | D00464 | hsa2565 | D01230 | hsa2554 | D02771 | hsa285242 | D07867 |
| hsa2559 | D00464 | hsa2566 | D01230 | hsa2555 | D02771 | hsa3359 | D07867 |
| hsa2560 | D00464 | hsa2567 | D01230 | hsa2556 | D02771 | hsa9177 | D07867 |
| hsa2561 | D00464 | hsa2568 | D01230 | hsa2557 | D02771 | hsa775 | D07886 |
| hsa2562 | D00464 | hsa55879 | D01230 | hsa2558 | D02771 | hsa776 | D07886 |
| hsa2563 | D00464 | hsa11280 | D01243 | hsa2559 | D02771 | hsa778 | D07886 |
| hsa2564 | D00464 | hsa6323 | D01243 | hsa2560 | D02771 | hsa779 | D07886 |
| hsa2565 | D00464 | hsa6326 | D01243 | hsa2561 | D02771 | hsa11280 | D07894 |
| hsa2566 | D00464 | hsa6328 | D01243 | hsa2562 | D02771 | hsa6323 | D07894 |
| hsa2567 | D00464 | hsa6329 | D01243 | hsa2563 | D02771 | hsa6326 | D07894 |
| hsa2568 | D00464 | hsa6331 | D01243 | hsa2564 | D02771 | hsa6328 | D07894 |
| hsa55879 | D00464 | hsa6334 | D01243 | hsa2565 | D02771 | hsa6329 | D07894 |
| hsa2554 | D00470 | hsa6335 | D01243 | hsa2566 | D02771 | hsa6331 | D07894 |
| hsa2555 | D00470 | hsa6336 | D01243 | hsa2567 | D02771 | hsa6334 | D07894 |
| hsa2556 | D00470 | hsa2554 | D01245 | hsa2568 | D02771 | hsa6335 | D07894 |
| hsa2557 | D00470 | hsa2555 | D01245 | hsa55879 | D02771 | hsa6336 | D07894 |
| hsa2558 | D00470 | hsa2556 | D01245 | hsa2902 | D02780 | hsa775 | D07943 |
| hsa2559 | D00470 | hsa2557 | D01245 | hsa2903 | D02780 | hsa776 | D07943 |
| hsa2560 | D00470 | hsa2558 | D01245 | hsa2904 | D02780 | hsa778 | D07943 |
| hsa2561 | D00470 | hsa2559 | D01245 | hsa2905 | D02780 | hsa779 | D07943 |
| hsa2562 | D00470 | hsa2560 | D01245 | hsa2906 | D02780 | hsa11280 | D07962 |
| hsa2563 | D00470 | hsa2561 | D01245 | hsa170572 | D02829 | hsa6323 | D07962 |
| hsa2564 | D00470 | hsa2562 | D01245 | hsa200909 | D02829 | hsa6326 | D07962 |
| hsa2565 | D00470 | hsa2563 | D01245 | hsa285242 | D02829 | hsa6328 | D07962 |
| hsa2566 | D00470 | hsa2564 | D01245 | hsa3359 | D02829 | hsa6329 | D07962 |
| hsa2567 | D00470 | hsa2565 | D01245 | hsa9177 | D02829 | hsa6331 | D07962 |
| hsa2568 | D00470 | hsa2566 | D01245 | hsa2554 | D02833 | hsa6334 | D07962 |
| hsa55879 | D00470 | hsa2567 | D01245 | hsa2555 | D02833 | hsa6335 | D07962 |
| hsa11280 | D00477 | hsa2568 | D01245 | hsa2556 | D02833 | hsa6336 | D07962 |
| hsa6323 | D00477 | hsa55879 | D01245 | hsa2557 | D02833 | hsa8911 | D07971 |
| hsa6326 | D00477 | hsa2554 | D01253 | hsa2558 | D02833 | hsa8912 | D07971 |
| hsa6328 | D00477 | hsa2555 | D01253 | hsa2559 | D02833 | hsa8913 | D07971 |
| hsa6329 | D00477 | hsa2556 | D01253 | hsa2560 | D02833 | hsa11280 | D07993 |
| hsa6331 | D00477 | hsa2557 | D01253 | hsa2561 | D02833 | hsa6323 | D07993 |
| hsa6334 | D00477 | hsa2558 | D01253 | hsa2562 | D02833 | hsa6326 | D07993 |
| hsa6335 | D00477 | hsa2559 | D01253 | hsa2563 | D02833 | hsa6328 | D07993 |
| hsa6336 | D00477 | hsa2560 | D01253 | hsa2564 | D02833 | hsa6329 | D07993 |
| hsa1135 | D00492 | hsa2561 | D01253 | hsa2565 | D02833 | hsa6331 | D07993 |
| hsa1136 | D00492 | hsa2562 | D01253 | hsa2566 | D02833 | hsa6334 | D07993 |
| hsa1137 | D00492 | hsa2563 | D01253 | hsa2567 | D02833 | hsa6335 | D07993 |
| hsa1138 | D00492 | hsa2564 | D01253 | hsa2568 | D02833 | hsa6336 | D07993 |
| hsa1139 | D00492 | hsa2565 | D01253 | hsa55879 | D02833 | hsa775 | D08009 |
| hsa1141 | D00492 | hsa2566 | D01253 | hsa1137 | D02839 | hsa776 | D08009 |
| hsa1142 | D00492 | hsa2567 | D01253 | hsa1141 | D02839 | hsa778 | D08009 |
| hsa1143 | D00492 | hsa2568 | D01253 | hsa11280 | D02906 | hsa779 | D08009 |
| hsa55584 | D00492 | hsa55879 | D01253 | hsa6323 | D02906 | hsa3757 | D08060 |
| hsa57053 | D00492 | hsa2554 | D01254 | hsa6326 | D02906 | hsa10021 | D08095 |
| hsa8973 | D00492 | hsa2555 | D01254 | hsa6328 | D02906 | hsa348980 | D08095 |
| hsa775 | D00495 | hsa2556 | D01254 | hsa6329 | D02906 | hsa57657 | D08095 |
| hsa776 | D00495 | hsa2557 | D01254 | hsa6331 | D02906 | hsa610 | D08095 |
| hsa778 | D00495 | hsa2558 | D01254 | hsa6334 | D02906 | hsa2902 | D08098 |
| hsa779 | D00495 | hsa2559 | D01254 | hsa6335 | D02906 | hsa2903 | D08098 |
| hsa2554 | D00499 | hsa2560 | D01254 | hsa6336 | D02906 | hsa2904 | D08098 |
| hsa2555 | D00499 | hsa2561 | D01254 | hsa11280 | D02907 | hsa2905 | D08098 |
| hsa2556 | D00499 | hsa2562 | D01254 | hsa6323 | D02907 | hsa2906 | D08098 |
| hsa2557 | D00499 | hsa2563 | D01254 | hsa6326 | D02907 | hsa775 | D08111 |
| hsa2558 | D00499 | hsa2564 | D01254 | hsa6328 | D02907 | hsa776 | D08111 |
| hsa2559 | D00499 | hsa2565 | D01254 | hsa6329 | D02907 | hsa778 | D08111 |
| hsa2560 | D00499 | hsa2566 | D01254 | hsa6331 | D02907 | hsa779 | D08111 |
| hsa2561 | D00499 | hsa2567 | D01254 | hsa6334 | D02907 | hsa11280 | D08116 |
| hsa2562 | D00499 | hsa2568 | D01254 | hsa6335 | D02907 | hsa6323 | D08116 |
| hsa2563 | D00499 | hsa55879 | D01254 | hsa6336 | D02907 | hsa6326 | D08116 |
| hsa2564 | D00499 | hsa2554 | D01268 | hsa11280 | D02910 | hsa6328 | D08116 |
| hsa2565 | D00499 | hsa2555 | D01268 | hsa3752 | D02910 | hsa6329 | D08116 |
| hsa2566 | D00499 | hsa2556 | D01268 | hsa3757 | D02910 | hsa6331 | D08116 |
| hsa2567 | D00499 | hsa2557 | D01268 | hsa3760 | D02910 | hsa6334 | D08116 |
| hsa2568 | D00499 | hsa2558 | D01268 | hsa3762 | D02910 | hsa6335 | D08116 |
| hsa55879 | D00499 | hsa2559 | D01268 | hsa3764 | D02910 | hsa6336 | D08116 |
| hsa2554 | D00500 | hsa2560 | D01268 | hsa3767 | D02910 | hsa2902 | D08121 |
| hsa2555 | D00500 | hsa2561 | D01268 | hsa3768 | D02910 | hsa2903 | D08121 |
| hsa2556 | D00500 | hsa2562 | D01268 | hsa3784 | D02910 | hsa2904 | D08121 |
| hsa2557 | D00500 | hsa2563 | D01268 | hsa6323 | D02910 | hsa2905 | D08121 |
| hsa2558 | D00500 | hsa2564 | D01268 | hsa6326 | D02910 | hsa2906 | D08121 |
| hsa2559 | D00500 | hsa2565 | D01268 | hsa6328 | D02910 | hsa2902 | D08122 |
| hsa2560 | D00500 | hsa2566 | D01268 | hsa6329 | D02910 | hsa2903 | D08122 |
| hsa2561 | D00500 | hsa2567 | D01268 | hsa6331 | D02910 | hsa2904 | D08122 |
| hsa2562 | D00500 | hsa2568 | D01268 | hsa6334 | D02910 | hsa2905 | D08122 |
| hsa2563 | D00500 | hsa55879 | D01268 | hsa6335 | D02910 | hsa2906 | D08122 |
| hsa2564 | D00500 | hsa2554 | D01278 | hsa6336 | D02910 | hsa11280 | D08127 |
| hsa2565 | D00500 | hsa2555 | D01278 | hsa775 | D02914 | hsa6323 | D08127 |
| hsa2566 | D00500 | hsa2556 | D01278 | hsa776 | D02914 | hsa6326 | D08127 |
| hsa2567 | D00500 | hsa2557 | D01278 | hsa778 | D02914 | hsa6328 | D08127 |
| hsa2568 | D00500 | hsa2558 | D01278 | hsa779 | D02914 | hsa6329 | D08127 |
| hsa55879 | D00500 | hsa2559 | D01278 | hsa11280 | D02969 | hsa6331 | D08127 |
| hsa2554 | D00506 | hsa2560 | D01278 | hsa6323 | D02969 | hsa6334 | D08127 |
| hsa2555 | D00506 | hsa2561 | D01278 | hsa6326 | D02969 | hsa6335 | D08127 |
| hsa2556 | D00506 | hsa2562 | D01278 | hsa6328 | D02969 | hsa6336 | D08127 |
| hsa2557 | D00506 | hsa2563 | D01278 | hsa6329 | D02969 | hsa1134 | D08138 |
| hsa2558 | D00506 | hsa2564 | D01278 | hsa6331 | D02969 | hsa1135 | D08138 |
| hsa2559 | D00506 | hsa2565 | D01278 | hsa6334 | D02969 | hsa1136 | D08138 |
| hsa2560 | D00506 | hsa2566 | D01278 | hsa6335 | D02969 | hsa1137 | D08138 |
| hsa2561 | D00506 | hsa2567 | D01278 | hsa6336 | D02969 | hsa1138 | D08138 |
| hsa2562 | D00506 | hsa2568 | D01278 | hsa2902 | D02973 | hsa1139 | D08138 |
| hsa2563 | D00506 | hsa55879 | D01278 | hsa2903 | D02973 | hsa1140 | D08138 |
| hsa2564 | D00506 | hsa2554 | D01279 | hsa2904 | D02973 | hsa1141 | D08138 |
| hsa2565 | D00506 | hsa2555 | D01279 | hsa2905 | D02973 | hsa1142 | D08138 |
| hsa2566 | D00506 | hsa2556 | D01279 | hsa2906 | D02973 | hsa1143 | D08138 |
| hsa2567 | D00506 | hsa2557 | D01279 | hsa11280 | D02991 | hsa1144 | D08138 |
| hsa2568 | D00506 | hsa2558 | D01279 | hsa6323 | D02991 | hsa1145 | D08138 |
| hsa55879 | D00506 | hsa2559 | D01279 | hsa6326 | D02991 | hsa1146 | D08138 |
| hsa11280 | D00512 | hsa2560 | D01279 | hsa6328 | D02991 | hsa55584 | D08138 |
| hsa6323 | D00512 | hsa2561 | D01279 | hsa6329 | D02991 | hsa57053 | D08138 |
| hsa6326 | D00512 | hsa2562 | D01279 | hsa6331 | D02991 | hsa8973 | D08138 |
| hsa6328 | D00512 | hsa2563 | D01279 | hsa6334 | D02991 | hsa2554 | D08145 |
| hsa6329 | D00512 | hsa2564 | D01279 | hsa6335 | D02991 | hsa2555 | D08145 |
| hsa6331 | D00512 | hsa2565 | D01279 | hsa6336 | D02991 | hsa2556 | D08145 |
| hsa6334 | D00512 | hsa2566 | D01279 | hsa3757 | D03037 | hsa2557 | D08145 |
| hsa6335 | D00512 | hsa2567 | D01279 | hsa3784 | D03037 | hsa2558 | D08145 |
| hsa6336 | D00512 | hsa2568 | D01279 | hsa170572 | D03060 | hsa2559 | D08145 |
| hsa3757 | D00521 | hsa55879 | D01279 | hsa200909 | D03060 | hsa2560 | D08145 |
| hsa2554 | D00530 | hsa2554 | D01286 | hsa285242 | D03060 | hsa2561 | D08145 |
| hsa2554 | D00531 | hsa2555 | D01286 | hsa3359 | D03060 | hsa2562 | D08145 |
| hsa2555 | D00531 | hsa2556 | D01286 | hsa9177 | D03060 | hsa2563 | D08145 |
| hsa2556 | D00531 | hsa2557 | D01286 | hsa775 | D03067 | hsa2564 | D08145 |
| hsa2557 | D00531 | hsa2558 | D01286 | hsa776 | D03067 | hsa2565 | D08145 |
| hsa2558 | D00531 | hsa2559 | D01286 | hsa778 | D03067 | hsa2566 | D08145 |
| hsa2559 | D00531 | hsa2560 | D01286 | hsa779 | D03067 | hsa2567 | D08145 |
| hsa2560 | D00531 | hsa2561 | D01286 | hsa170572 | D03073 | hsa2568 | D08145 |
| hsa2561 | D00531 | hsa2562 | D01286 | hsa200909 | D03073 | hsa55879 | D08145 |
| hsa2562 | D00531 | hsa2563 | D01286 | hsa285242 | D03073 | hsa775 | D08155 |
| hsa2563 | D00531 | hsa2564 | D01286 | hsa3359 | D03073 | hsa776 | D08155 |
| hsa2564 | D00531 | hsa2565 | D01286 | hsa9177 | D03073 | hsa778 | D08155 |
| hsa2565 | D00531 | hsa2566 | D01286 | hsa2904 | D03100 | hsa779 | D08155 |
| hsa2566 | D00531 | hsa2567 | D01286 | hsa2554 | D03155 | hsa2902 | D08174 |
| hsa2567 | D00531 | hsa2568 | D01286 | hsa2555 | D03155 | hsa2903 | D08174 |
| hsa2568 | D00531 | hsa55879 | D01286 | hsa2556 | D03155 | hsa2904 | D08174 |
| hsa55879 | D00531 | hsa11280 | D01287 | hsa2557 | D03155 | hsa2905 | D08174 |
| hsa2554 | D00532 | hsa6323 | D01287 | hsa2558 | D03155 | hsa2906 | D08174 |
| hsa2555 | D00532 | hsa6326 | D01287 | hsa2559 | D03155 | hsa11280 | D08181 |
| hsa2556 | D00532 | hsa6328 | D01287 | hsa2560 | D03155 | hsa6323 | D08181 |
| hsa2557 | D00532 | hsa6329 | D01287 | hsa2561 | D03155 | hsa6326 | D08181 |
| hsa2558 | D00532 | hsa6331 | D01287 | hsa2562 | D03155 | hsa6328 | D08181 |
| hsa2559 | D00532 | hsa6334 | D01287 | hsa2563 | D03155 | hsa6329 | D08181 |
| hsa2560 | D00532 | hsa6335 | D01287 | hsa2564 | D03155 | hsa6331 | D08181 |
| hsa2561 | D00532 | hsa6336 | D01287 | hsa2565 | D03155 | hsa6334 | D08181 |
| hsa2562 | D00532 | hsa2554 | D01292 | hsa2566 | D03155 | hsa6335 | D08181 |
| hsa2563 | D00532 | hsa2555 | D01292 | hsa2567 | D03155 | hsa6336 | D08181 |
| hsa2564 | D00532 | hsa2556 | D01292 | hsa2568 | D03155 | hsa2902 | D08195 |
| hsa2565 | D00532 | hsa2557 | D01292 | hsa55879 | D03155 | hsa2903 | D08195 |
| hsa2566 | D00532 | hsa2558 | D01292 | hsa2554 | D03180 | hsa2904 | D08195 |
| hsa2567 | D00532 | hsa2559 | D01292 | hsa2555 | D03180 | hsa2905 | D08195 |
| hsa2568 | D00532 | hsa2560 | D01292 | hsa2556 | D03180 | hsa2906 | D08195 |
| hsa55879 | D00532 | hsa2561 | D01292 | hsa2557 | D03180 | hsa11280 | D08215 |
| hsa11280 | D00533 | hsa2562 | D01292 | hsa2558 | D03180 | hsa6323 | D08215 |
| hsa6323 | D00533 | hsa2563 | D01292 | hsa2559 | D03180 | hsa6326 | D08215 |
| hsa6326 | D00533 | hsa2564 | D01292 | hsa2560 | D03180 | hsa6328 | D08215 |
| hsa6328 | D00533 | hsa2565 | D01292 | hsa2561 | D03180 | hsa6329 | D08215 |
| hsa6329 | D00533 | hsa2566 | D01292 | hsa2562 | D03180 | hsa6331 | D08215 |
| hsa6331 | D00533 | hsa2567 | D01292 | hsa2563 | D03180 | hsa6334 | D08215 |
| hsa6334 | D00533 | hsa2568 | D01292 | hsa2564 | D03180 | hsa6335 | D08215 |
| hsa6335 | D00533 | hsa55879 | D01292 | hsa2565 | D03180 | hsa6336 | D08215 |
| hsa6336 | D00533 | hsa2554 | D01293 | hsa2566 | D03180 | hsa775 | D08217 |
| hsa116443 | D00536 | hsa2555 | D01293 | hsa2567 | D03180 | hsa776 | D08217 |
| hsa116444 | D00536 | hsa2556 | D01293 | hsa2568 | D03180 | hsa778 | D08217 |
| hsa2902 | D00536 | hsa2557 | D01293 | hsa55879 | D03180 | hsa779 | D08217 |
| hsa2903 | D00536 | hsa2558 | D01293 | hsa1134 | D03365 | hsa8911 | D08217 |
| hsa2904 | D00536 | hsa2559 | D01293 | hsa1135 | D03365 | hsa8912 | D08217 |
| hsa2905 | D00536 | hsa2560 | D01293 | hsa1136 | D03365 | hsa8913 | D08217 |
| hsa2906 | D00536 | hsa2561 | D01293 | hsa1137 | D03365 | hsa775 | D08270 |
| hsa11280 | D00537 | hsa2562 | D01293 | hsa1138 | D03365 | hsa776 | D08270 |
| hsa2554 | D00537 | hsa2563 | D01293 | hsa1139 | D03365 | hsa778 | D08270 |
| hsa2555 | D00537 | hsa2564 | D01293 | hsa1140 | D03365 | hsa779 | D08270 |
| hsa2556 | D00537 | hsa2565 | D01293 | hsa1141 | D03365 | hsa1179 | D08275 |
| hsa2557 | D00537 | hsa2566 | D01293 | hsa1142 | D03365 | hsa22802 | D08275 |
| hsa2558 | D00537 | hsa2567 | D01293 | hsa1143 | D03365 | hsa9635 | D08275 |
| hsa2559 | D00537 | hsa2568 | D01293 | hsa1144 | D03365 | hsa7442 | D08282 |
| hsa2560 | D00537 | hsa55879 | D01293 | hsa1145 | D03365 | hsa2554 | D08283 |
| hsa2561 | D00537 | hsa8911 | D01303 | hsa1146 | D03365 | hsa2555 | D08283 |
| hsa2562 | D00537 | hsa8912 | D01303 | hsa55584 | D03365 | hsa2556 | D08283 |
| hsa2563 | D00537 | hsa8913 | D01303 | hsa57053 | D03365 | hsa2557 | D08283 |
| hsa2564 | D00537 | hsa2554 | D01310 | hsa8973 | D03365 | hsa2558 | D08283 |
| hsa2565 | D00537 | hsa2555 | D01310 | hsa11280 | D03492 | hsa2559 | D08283 |
| hsa2566 | D00537 | hsa2556 | D01310 | hsa6323 | D03492 | hsa2560 | D08283 |
| hsa2567 | D00537 | hsa2557 | D01310 | hsa6326 | D03492 | hsa2561 | D08283 |
| hsa2568 | D00537 | hsa2558 | D01310 | hsa6328 | D03492 | hsa2562 | D08283 |
| hsa2890 | D00537 | hsa2559 | D01310 | hsa6329 | D03492 | hsa2563 | D08283 |
| hsa2891 | D00537 | hsa2560 | D01310 | hsa6331 | D03492 | hsa2564 | D08283 |
| hsa2892 | D00537 | hsa2561 | D01310 | hsa6334 | D03492 | hsa2565 | D08283 |
| hsa2893 | D00537 | hsa2562 | D01310 | hsa6335 | D03492 | hsa2566 | D08283 |
| hsa2897 | D00537 | hsa2563 | D01310 | hsa6336 | D03492 | hsa2567 | D08283 |
| hsa2898 | D00537 | hsa2564 | D01310 | hsa170572 | D03495 | hsa2568 | D08283 |
| hsa2899 | D00537 | hsa2565 | D01310 | hsa200909 | D03495 | hsa55879 | D08283 |
| hsa2900 | D00537 | hsa2566 | D01310 | hsa285242 | D03495 | hsa116443 | D08305 |
| hsa2901 | D00537 | hsa2567 | D01310 | hsa3359 | D03495 | hsa116444 | D08305 |
| hsa55879 | D00537 | hsa2568 | D01310 | hsa9177 | D03495 | hsa2902 | D08305 |
| hsa6323 | D00537 | hsa55879 | D01310 | hsa170572 | D03496 | hsa2903 | D08305 |
| hsa6326 | D00537 | hsa2554 | D01316 | hsa200909 | D03496 | hsa2904 | D08305 |
| hsa6328 | D00537 | hsa2555 | D01316 | hsa285242 | D03496 | hsa2905 | D08305 |
| hsa6329 | D00537 | hsa2556 | D01316 | hsa3359 | D03496 | hsa2906 | D08305 |
| hsa6331 | D00537 | hsa2557 | D01316 | hsa9177 | D03496 | hsa11280 | D08311 |
| hsa6334 | D00537 | hsa2558 | D01316 | hsa775 | D03536 | hsa6323 | D08311 |
| hsa6335 | D00537 | hsa2559 | D01316 | hsa776 | D03536 | hsa6326 | D08311 |
| hsa6336 | D00537 | hsa2560 | D01316 | hsa778 | D03536 | hsa6328 | D08311 |
| hsa775 | D00537 | hsa2561 | D01316 | hsa779 | D03536 | hsa6329 | D08311 |
| hsa776 | D00537 | hsa2562 | D01316 | hsa343450 | D03547 | hsa6331 | D08311 |
| hsa778 | D00537 | hsa2563 | D01316 | hsa3750 | D03547 | hsa6334 | D08311 |
| hsa779 | D00537 | hsa2564 | D01316 | hsa3751 | D03547 | hsa6335 | D08311 |
| hsa11280 | D00538 | hsa2565 | D01316 | hsa3752 | D03547 | hsa6336 | D08311 |
| hsa6323 | D00538 | hsa2566 | D01316 | hsa3756 | D03547 | hsa11280 | D08319 |
| hsa6326 | D00538 | hsa2567 | D01316 | hsa3757 | D03547 | hsa6323 | D08319 |
| hsa6328 | D00538 | hsa2568 | D01316 | hsa2554 | D03562 | hsa6326 | D08319 |
| hsa6329 | D00538 | hsa55879 | D01316 | hsa2555 | D03562 | hsa6328 | D08319 |
| hsa6331 | D00538 | hsa11280 | D01326 | hsa2556 | D03562 | hsa6329 | D08319 |
| hsa6334 | D00538 | hsa6323 | D01326 | hsa2557 | D03562 | hsa6331 | D08319 |
| hsa6335 | D00538 | hsa6326 | D01326 | hsa2558 | D03562 | hsa6334 | D08319 |
| hsa6336 | D00538 | hsa6328 | D01326 | hsa2559 | D03562 | hsa6335 | D08319 |
| hsa8911 | D00538 | hsa6329 | D01326 | hsa2560 | D03562 | hsa6336 | D08319 |
| hsa8912 | D00538 | hsa6331 | D01326 | hsa2561 | D03562 | hsa775 | D08340 |
| hsa8913 | D00538 | hsa6334 | D01326 | hsa2562 | D03562 | hsa776 | D08340 |
| hsa8911 | D00539 | hsa6335 | D01326 | hsa2563 | D03562 | hsa778 | D08340 |
| hsa8912 | D00539 | hsa6336 | D01326 | hsa2564 | D03562 | hsa779 | D08340 |
| hsa8913 | D00539 | hsa2554 | D01328 | hsa2565 | D03562 | hsa2554 | D08356 |
| hsa2554 | D00548 | hsa2555 | D01328 | hsa2566 | D03562 | hsa2555 | D08356 |
| hsa2554 | D00549 | hsa2556 | D01328 | hsa2567 | D03562 | hsa2556 | D08356 |
| hsa2555 | D00549 | hsa2557 | D01328 | hsa2568 | D03562 | hsa2557 | D08356 |
| hsa2556 | D00549 | hsa2558 | D01328 | hsa55879 | D03562 | hsa2558 | D08356 |
| hsa2557 | D00549 | hsa2559 | D01328 | hsa775 | D03655 | hsa2559 | D08356 |
| hsa2558 | D00549 | hsa2560 | D01328 | hsa776 | D03655 | hsa2560 | D08356 |
| hsa2559 | D00549 | hsa2561 | D01328 | hsa778 | D03655 | hsa2561 | D08356 |
| hsa2560 | D00549 | hsa2562 | D01328 | hsa779 | D03655 | hsa2562 | D08356 |
| hsa2561 | D00549 | hsa2563 | D01328 | hsa170572 | D03662 | hsa2563 | D08356 |
| hsa2562 | D00549 | hsa2564 | D01328 | hsa200909 | D03662 | hsa2564 | D08356 |
| hsa2563 | D00549 | hsa2565 | D01328 | hsa285242 | D03662 | hsa2565 | D08356 |
| hsa2564 | D00549 | hsa2566 | D01328 | hsa3359 | D03662 | hsa2566 | D08356 |
| hsa2565 | D00549 | hsa2567 | D01328 | hsa9177 | D03662 | hsa2567 | D08356 |
| hsa2566 | D00549 | hsa2568 | D01328 | hsa2902 | D03679 | hsa2568 | D08356 |
| hsa2567 | D00549 | hsa55879 | D01328 | hsa2903 | D03679 | hsa55879 | D08356 |
| hsa2568 | D00549 | hsa2554 | D01354 | hsa2904 | D03679 | hsa11280 | D08377 |
| hsa55879 | D00549 | hsa2555 | D01354 | hsa2905 | D03679 | hsa6323 | D08377 |
| hsa2554 | D00550 | hsa2556 | D01354 | hsa2906 | D03679 | hsa6326 | D08377 |
| hsa2555 | D00550 | hsa2557 | D01354 | hsa3757 | D03732 | hsa6328 | D08377 |
| hsa2556 | D00550 | hsa2558 | D01354 | hsa2554 | D03737 | hsa6329 | D08377 |
| hsa2557 | D00550 | hsa2559 | D01354 | hsa2555 | D03737 | hsa6331 | D08377 |
| hsa2558 | D00550 | hsa2560 | D01354 | hsa2556 | D03737 | hsa6334 | D08377 |
| hsa2559 | D00550 | hsa2561 | D01354 | hsa2557 | D03737 | hsa6335 | D08377 |
| hsa2560 | D00550 | hsa2562 | D01354 | hsa2558 | D03737 | hsa6336 | D08377 |
| hsa2561 | D00550 | hsa2563 | D01354 | hsa2559 | D03737 | hsa11280 | D08394 |
| hsa2562 | D00550 | hsa2564 | D01354 | hsa2560 | D03737 | hsa6323 | D08394 |
| hsa2563 | D00550 | hsa2565 | D01354 | hsa2561 | D03737 | hsa6326 | D08394 |
| hsa2564 | D00550 | hsa2566 | D01354 | hsa2562 | D03737 | hsa6328 | D08394 |
| hsa2565 | D00550 | hsa2567 | D01354 | hsa2563 | D03737 | hsa6329 | D08394 |
| hsa2566 | D00550 | hsa2568 | D01354 | hsa2564 | D03737 | hsa6331 | D08394 |
| hsa2567 | D00550 | hsa55879 | D01354 | hsa2565 | D03737 | hsa6334 | D08394 |
| hsa2568 | D00550 | hsa3767 | D01356 | hsa2566 | D03737 | hsa6335 | D08394 |
| hsa55879 | D00550 | hsa775 | D01369 | hsa2567 | D03737 | hsa6336 | D08394 |
| hsa11280 | D00551 | hsa776 | D01369 | hsa2568 | D03737 | hsa11280 | D08407 |
| hsa6323 | D00551 | hsa778 | D01369 | hsa55879 | D03737 | hsa6323 | D08407 |
| hsa6326 | D00551 | hsa779 | D01369 | hsa2902 | D03742 | hsa6326 | D08407 |
| hsa6328 | D00551 | hsa2554 | D01372 | hsa2903 | D03742 | hsa6328 | D08407 |
| hsa6329 | D00551 | hsa2555 | D01372 | hsa2904 | D03742 | hsa6329 | D08407 |
| hsa6331 | D00551 | hsa2556 | D01372 | hsa2905 | D03742 | hsa6331 | D08407 |
| hsa6334 | D00551 | hsa2557 | D01372 | hsa2906 | D03742 | hsa6334 | D08407 |
| hsa6335 | D00551 | hsa2558 | D01372 | hsa2902 | D03744 | hsa6335 | D08407 |
| hsa6336 | D00551 | hsa2559 | D01372 | hsa2903 | D03744 | hsa6336 | D08407 |
| hsa11280 | D00552 | hsa2560 | D01372 | hsa2904 | D03744 | hsa11280 | D08421 |
| hsa6323 | D00552 | hsa2561 | D01372 | hsa2905 | D03744 | hsa6323 | D08421 |
| hsa6326 | D00552 | hsa2562 | D01372 | hsa2906 | D03744 | hsa6326 | D08421 |
| hsa6328 | D00552 | hsa2563 | D01372 | hsa2902 | D03746 | hsa6328 | D08421 |
| hsa6329 | D00552 | hsa2564 | D01372 | hsa2903 | D03746 | hsa6329 | D08421 |
| hsa6331 | D00552 | hsa2565 | D01372 | hsa2904 | D03746 | hsa6331 | D08421 |
| hsa6334 | D00552 | hsa2566 | D01372 | hsa2905 | D03746 | hsa6334 | D08421 |
| hsa6335 | D00552 | hsa2567 | D01372 | hsa2906 | D03746 | hsa6335 | D08421 |
| hsa6336 | D00552 | hsa2568 | D01372 | hsa775 | D03830 | hsa6336 | D08421 |
| hsa11280 | D00553 | hsa55879 | D01372 | hsa776 | D03830 | hsa11280 | D08422 |
| hsa6323 | D00553 | hsa2554 | D01382 | hsa778 | D03830 | hsa6323 | D08422 |
| hsa6326 | D00553 | hsa2555 | D01382 | hsa779 | D03830 | hsa6326 | D08422 |
| hsa6328 | D00553 | hsa2556 | D01382 | hsa2902 | D03878 | hsa6328 | D08422 |
| hsa6329 | D00553 | hsa2557 | D01382 | hsa2903 | D03878 | hsa6329 | D08422 |
| hsa6331 | D00553 | hsa2558 | D01382 | hsa2904 | D03878 | hsa6331 | D08422 |
| hsa6334 | D00553 | hsa2559 | D01382 | hsa2905 | D03878 | hsa6334 | D08422 |
| hsa6335 | D00553 | hsa2560 | D01382 | hsa2906 | D03878 | hsa6335 | D08422 |
| hsa6336 | D00553 | hsa2561 | D01382 | hsa3752 | D03914 | hsa6336 | D08422 |
| hsa2554 | D00555 | hsa2562 | D01382 | hsa3757 | D03914 | hsa11280 | D08435 |
| hsa2555 | D00555 | hsa2563 | D01382 | hsa3760 | D03914 | hsa6323 | D08435 |
| hsa2556 | D00555 | hsa2564 | D01382 | hsa3762 | D03914 | hsa6326 | D08435 |
| hsa2557 | D00555 | hsa2565 | D01382 | hsa3767 | D03914 | hsa6328 | D08435 |
| hsa2558 | D00555 | hsa2566 | D01382 | hsa3784 | D03914 | hsa6329 | D08435 |
| hsa2559 | D00555 | hsa2567 | D01382 | hsa775 | D03914 | hsa6331 | D08435 |
| hsa2560 | D00555 | hsa2568 | D01382 | hsa776 | D03914 | hsa6334 | D08435 |
| hsa2561 | D00555 | hsa55879 | D01382 | hsa778 | D03914 | hsa6335 | D08435 |
| hsa2562 | D00555 | hsa2554 | D01408 | hsa779 | D03914 | hsa6336 | D08435 |
| hsa2563 | D00555 | hsa2555 | D01408 | hsa11280 | D03991 | hsa775 | D08441 |
| hsa2564 | D00555 | hsa2556 | D01408 | hsa6323 | D03991 | hsa776 | D08441 |
| hsa2565 | D00555 | hsa2557 | D01408 | hsa6326 | D03991 | hsa778 | D08441 |
| hsa2566 | D00555 | hsa2558 | D01408 | hsa6328 | D03991 | hsa779 | D08441 |
| hsa2567 | D00555 | hsa2559 | D01408 | hsa6329 | D03991 | hsa11280 | D08448 |
| hsa2568 | D00555 | hsa2560 | D01408 | hsa6331 | D03991 | hsa6323 | D08448 |
| hsa55879 | D00555 | hsa2561 | D01408 | hsa6334 | D03991 | hsa6326 | D08448 |
| hsa170572 | D00563 | hsa2562 | D01408 | hsa6335 | D03991 | hsa6328 | D08448 |
| hsa200909 | D00563 | hsa2563 | D01408 | hsa6336 | D03991 | hsa6329 | D08448 |
| hsa285242 | D00563 | hsa2564 | D01408 | hsa11280 | D04048 | hsa6331 | D08448 |
| hsa3359 | D00563 | hsa2565 | D01408 | hsa6323 | D04048 | hsa6334 | D08448 |
| hsa9177 | D00563 | hsa2566 | D01408 | hsa6326 | D04048 | hsa6335 | D08448 |
| hsa3767 | D00594 | hsa2567 | D01408 | hsa6328 | D04048 | hsa6336 | D08448 |
| hsa1134 | D00611 | hsa2568 | D01408 | hsa6329 | D04048 | hsa11280 | D08458 |
| hsa1135 | D00611 | hsa55879 | D01408 | hsa6331 | D04048 | hsa6323 | D08458 |
| hsa1136 | D00611 | hsa11280 | D01450 | hsa6334 | D04048 | hsa6326 | D08458 |
| hsa1137 | D00611 | hsa6323 | D01450 | hsa6335 | D04048 | hsa6328 | D08458 |
| hsa1138 | D00611 | hsa6326 | D01450 | hsa6336 | D04048 | hsa6329 | D08458 |
| hsa1139 | D00611 | hsa6328 | D01450 | hsa6323 | D04095 | hsa6331 | D08458 |
| hsa1140 | D00611 | hsa6329 | D01450 | hsa6326 | D04095 | hsa6334 | D08458 |
| hsa1141 | D00611 | hsa6331 | D01450 | hsa6328 | D04095 | hsa6335 | D08458 |
| hsa1142 | D00611 | hsa6334 | D01450 | hsa6329 | D04095 | hsa6336 | D08458 |
| hsa1143 | D00611 | hsa6335 | D01450 | hsa6331 | D04095 | hsa11280 | D08459 |
| hsa1144 | D00611 | hsa6336 | D01450 | hsa6334 | D04095 | hsa6323 | D08459 |
| hsa1145 | D00611 | hsa11280 | D01455 | hsa6335 | D04095 | hsa6326 | D08459 |
| hsa1146 | D00611 | hsa6323 | D01455 | hsa6336 | D04095 | hsa6328 | D08459 |
| hsa55584 | D00611 | hsa6326 | D01455 | hsa3736 | D04127 | hsa6329 | D08459 |
| hsa57053 | D00611 | hsa6328 | D01455 | hsa3737 | D04127 | hsa6331 | D08459 |
| hsa8973 | D00611 | hsa6329 | D01455 | hsa3738 | D04127 | hsa6334 | D08459 |
| hsa57053 | D00612 | hsa6331 | D01455 | hsa3739 | D04127 | hsa6335 | D08459 |
| hsa775 | D00615 | hsa6334 | D01455 | hsa3741 | D04127 | hsa6336 | D08459 |
| hsa776 | D00615 | hsa6335 | D01455 | hsa3742 | D04127 | hsa170572 | D08466 |
| hsa778 | D00615 | hsa6336 | D01455 | hsa3743 | D04127 | hsa200909 | D08466 |
| hsa779 | D00615 | hsa2554 | D01514 | hsa3744 | D04127 | hsa285242 | D08466 |
| hsa775 | D00616 | hsa2555 | D01514 | hsa3745 | D04127 | hsa3359 | D08466 |
| hsa776 | D00616 | hsa2556 | D01514 | hsa3746 | D04127 | hsa9177 | D08466 |
| hsa778 | D00616 | hsa2557 | D01514 | hsa3747 | D04127 | hsa2554 | D08481 |
| hsa779 | D00616 | hsa2558 | D01514 | hsa3748 | D04127 | hsa2555 | D08481 |
| hsa775 | D00617 | hsa2559 | D01514 | hsa3749 | D04127 | hsa2556 | D08481 |
| hsa776 | D00617 | hsa2560 | D01514 | hsa3750 | D04127 | hsa2557 | D08481 |
| hsa778 | D00617 | hsa2561 | D01514 | hsa3751 | D04127 | hsa2558 | D08481 |
| hsa779 | D00617 | hsa2562 | D01514 | hsa3752 | D04127 | hsa2559 | D08481 |
| hsa775 | D00618 | hsa2563 | D01514 | hsa9312 | D04127 | hsa2560 | D08481 |
| hsa776 | D00618 | hsa2564 | D01514 | hsa2890 | D04131 | hsa2561 | D08481 |
| hsa778 | D00618 | hsa2565 | D01514 | hsa2891 | D04131 | hsa2562 | D08481 |
| hsa779 | D00618 | hsa2566 | D01514 | hsa2892 | D04131 | hsa2563 | D08481 |
| hsa775 | D00619 | hsa2567 | D01514 | hsa2893 | D04131 | hsa2564 | D08481 |
| hsa776 | D00619 | hsa2568 | D01514 | hsa3779 | D04192 | hsa2565 | D08481 |
| hsa778 | D00619 | hsa55879 | D01514 | hsa3784 | D04192 | hsa2566 | D08481 |
| hsa779 | D00619 | hsa11280 | D01519 | hsa3785 | D04192 | hsa2567 | D08481 |
| hsa775 | D00629 | hsa6323 | D01519 | hsa3786 | D04192 | hsa2568 | D08481 |
| hsa776 | D00629 | hsa6326 | D01519 | hsa56479 | D04192 | hsa55879 | D08481 |
| hsa778 | D00629 | hsa6328 | D01519 | hsa9132 | D04192 | hsa11280 | D08490 |
| hsa779 | D00629 | hsa6329 | D01519 | hsa775 | D04193 | hsa6323 | D08490 |
| hsa3741 | D00631 | hsa6331 | D01519 | hsa776 | D04193 | hsa6326 | D08490 |
| hsa3752 | D00631 | hsa6334 | D01519 | hsa778 | D04193 | hsa6328 | D08490 |
| hsa3757 | D00631 | hsa6335 | D01519 | hsa779 | D04193 | hsa6329 | D08490 |
| hsa3760 | D00631 | hsa6336 | D01519 | hsa2554 | D04257 | hsa6331 | D08490 |
| hsa3762 | D00631 | hsa775 | D01553 | hsa2555 | D04257 | hsa6334 | D08490 |
| hsa3764 | D00631 | hsa776 | D01553 | hsa2556 | D04257 | hsa6335 | D08490 |
| hsa3767 | D00631 | hsa778 | D01553 | hsa2557 | D04257 | hsa6336 | D08490 |
| hsa3768 | D00631 | hsa779 | D01553 | hsa2558 | D04257 | hsa2554 | D08507 |
| hsa3784 | D00631 | hsa11280 | D01554 | hsa2559 | D04257 | hsa2555 | D08507 |
| hsa775 | D00631 | hsa6323 | D01554 | hsa2560 | D04257 | hsa2556 | D08507 |
| hsa776 | D00631 | hsa6326 | D01554 | hsa2561 | D04257 | hsa2557 | D08507 |
| hsa778 | D00631 | hsa6328 | D01554 | hsa2562 | D04257 | hsa2558 | D08507 |
| hsa779 | D00631 | hsa6329 | D01554 | hsa2563 | D04257 | hsa2559 | D08507 |
| hsa8911 | D00631 | hsa6331 | D01554 | hsa2564 | D04257 | hsa2560 | D08507 |
| hsa8912 | D00631 | hsa6334 | D01554 | hsa2565 | D04257 | hsa2561 | D08507 |
| hsa8913 | D00631 | hsa6335 | D01554 | hsa2566 | D04257 | hsa2562 | D08507 |
| hsa11280 | D00636 | hsa6336 | D01554 | hsa2567 | D04257 | hsa2563 | D08507 |
| hsa3752 | D00636 | hsa775 | D01562 | hsa2568 | D04257 | hsa2564 | D08507 |
| hsa3757 | D00636 | hsa776 | D01562 | hsa55879 | D04257 | hsa2565 | D08507 |
| hsa3760 | D00636 | hsa778 | D01562 | hsa775 | D04260 | hsa2566 | D08507 |
| hsa3762 | D00636 | hsa779 | D01562 | hsa776 | D04260 | hsa2567 | D08507 |
| hsa3764 | D00636 | hsa2554 | D01564 | hsa778 | D04260 | hsa2568 | D08507 |
| hsa3767 | D00636 | hsa2555 | D01564 | hsa779 | D04260 | hsa55879 | D08507 |
| hsa3768 | D00636 | hsa2556 | D01564 | hsa2554 | D04282 | hsa3757 | D08525 |
| hsa3784 | D00636 | hsa2557 | D01564 | hsa2555 | D04282 | hsa2902 | D08596 |
| hsa6323 | D00636 | hsa2558 | D01564 | hsa2556 | D04282 | hsa2903 | D08596 |
| hsa6326 | D00636 | hsa2559 | D01564 | hsa2557 | D04282 | hsa2904 | D08596 |
| hsa6328 | D00636 | hsa2560 | D01564 | hsa2558 | D04282 | hsa2905 | D08596 |
| hsa6329 | D00636 | hsa2561 | D01564 | hsa2559 | D04282 | hsa2906 | D08596 |
| hsa6331 | D00636 | hsa2562 | D01564 | hsa2560 | D04282 | hsa1135 | D08655 |
| hsa6334 | D00636 | hsa2563 | D01564 | hsa2561 | D04282 | hsa1136 | D08655 |
| hsa6335 | D00636 | hsa2564 | D01564 | hsa2562 | D04282 | hsa1137 | D08655 |
| hsa6336 | D00636 | hsa2565 | D01564 | hsa2563 | D04282 | hsa1138 | D08655 |
| hsa11280 | D00637 | hsa2566 | D01564 | hsa2564 | D04282 | hsa1139 | D08655 |
| hsa6323 | D00637 | hsa2567 | D01564 | hsa2565 | D04282 | hsa1141 | D08655 |
| hsa6326 | D00637 | hsa2568 | D01564 | hsa2566 | D04282 | hsa1142 | D08655 |
| hsa6328 | D00637 | hsa55879 | D01564 | hsa2567 | D04282 | hsa1143 | D08655 |
| hsa6329 | D00637 | hsa2554 | D01593 | hsa2568 | D04282 | hsa55584 | D08655 |
| hsa6331 | D00637 | hsa2555 | D01593 | hsa55879 | D04282 | hsa57053 | D08655 |
| hsa6334 | D00637 | hsa2556 | D01593 | hsa2554 | D04300 | hsa8973 | D08655 |
| hsa6335 | D00637 | hsa2557 | D01593 | hsa2555 | D04300 | hsa8911 | D08667 |
| hsa6336 | D00637 | hsa2558 | D01593 | hsa2556 | D04300 | hsa8912 | D08667 |
| hsa11280 | D00638 | hsa2559 | D01593 | hsa2557 | D04300 | hsa8913 | D08667 |
| hsa6323 | D00638 | hsa2560 | D01593 | hsa2558 | D04300 | hsa1137 | D08669 |
| hsa6326 | D00638 | hsa2561 | D01593 | hsa2559 | D04300 | hsa1141 | D08669 |
| hsa6328 | D00638 | hsa2562 | D01593 | hsa1134 | D04303 | hsa774 | D08686 |
| hsa6329 | D00638 | hsa2563 | D01593 | hsa1140 | D04303 | hsa2554 | D08690 |
| hsa6331 | D00638 | hsa2564 | D01593 | hsa1144 | D04303 | hsa2555 | D08690 |
| hsa6334 | D00638 | hsa2565 | D01593 | hsa1145 | D04303 | hsa2556 | D08690 |
| hsa6335 | D00638 | hsa2566 | D01593 | hsa1146 | D04303 | hsa2557 | D08690 |
| hsa6336 | D00638 | hsa2567 | D01593 | hsa2902 | D04308 | hsa2558 | D08690 |
| hsa11280 | D00639 | hsa2568 | D01593 | hsa2903 | D04308 | hsa2559 | D08690 |
| hsa6323 | D00639 | hsa55879 | D01593 | hsa2904 | D04308 | hsa2560 | D08690 |
| hsa6326 | D00639 | hsa3758 | D01603 | hsa2905 | D04308 | hsa2561 | D08690 |
| hsa6328 | D00639 | hsa775 | D01604 | hsa2906 | D04308 | hsa2562 | D08690 |
| hsa6329 | D00639 | hsa776 | D01604 | hsa9177 | D04370 | hsa2563 | D08690 |
| hsa6331 | D00639 | hsa778 | D01604 | hsa170572 | D04642 | hsa2564 | D08690 |
| hsa6334 | D00639 | hsa779 | D01604 | hsa200909 | D04642 | hsa2565 | D08690 |
| hsa6335 | D00639 | hsa170572 | D01613 | hsa285242 | D04642 | hsa2566 | D08690 |
| hsa6336 | D00639 | hsa200909 | D01613 | hsa3359 | D04642 | hsa2567 | D08690 |
| hsa11280 | D00640 | hsa285242 | D01613 | hsa9177 | D04642 | hsa2568 | D08690 |
| hsa6323 | D00640 | hsa3359 | D01613 | hsa2554 | D04650 | hsa55879 | D08690 |
| hsa6326 | D00640 | hsa9177 | D01613 | hsa2555 | D04650 | hsa2554 | D08840 |
| hsa6328 | D00640 | hsa2554 | D01657 | hsa2556 | D04650 | hsa2555 | D08840 |
| hsa6329 | D00640 | hsa2555 | D01657 | hsa2557 | D04650 | hsa2556 | D08840 |
| hsa6331 | D00640 | hsa2556 | D01657 | hsa2558 | D04650 | hsa2557 | D08840 |
| hsa6334 | D00640 | hsa2557 | D01657 | hsa2559 | D04650 | hsa2558 | D08840 |
| hsa6335 | D00640 | hsa2558 | D01657 | hsa2560 | D04650 | hsa2559 | D08840 |
| hsa6336 | D00640 | hsa2559 | D01657 | hsa2561 | D04650 | hsa2560 | D08840 |
| hsa11280 | D00642 | hsa2560 | D01657 | hsa2562 | D04650 | hsa2561 | D08840 |
| hsa6323 | D00642 | hsa2561 | D01657 | hsa2563 | D04650 | hsa2562 | D08840 |
| hsa6326 | D00642 | hsa2562 | D01657 | hsa2564 | D04650 | hsa2563 | D08840 |
| hsa6328 | D00642 | hsa2563 | D01657 | hsa2565 | D04650 | hsa2564 | D08840 |
| hsa6329 | D00642 | hsa2564 | D01657 | hsa2566 | D04650 | hsa2565 | D08840 |
| hsa6331 | D00642 | hsa2565 | D01657 | hsa2567 | D04650 | hsa2566 | D08840 |
| hsa6334 | D00642 | hsa2566 | D01657 | hsa2568 | D04650 | hsa2567 | D08840 |
| hsa6335 | D00642 | hsa2567 | D01657 | hsa55879 | D04650 | hsa2568 | D08840 |
| hsa6336 | D00642 | hsa2568 | D01657 | hsa775 | D04657 | hsa55879 | D08840 |
| hsa11280 | D00643 | hsa55879 | D01657 | hsa776 | D04657 | hsa775 | D08892 |
| hsa6323 | D00643 | hsa7224 | D01705 | hsa778 | D04657 | hsa776 | D08892 |
| hsa6326 | D00643 | hsa2554 | D01740 | hsa779 | D04657 | hsa778 | D08892 |
| hsa6328 | D00643 | hsa2555 | D01740 | hsa2554 | D04721 | hsa779 | D08892 |
| hsa6329 | D00643 | hsa2556 | D01740 | hsa2555 | D04721 | hsa1137 | D08935 |
| hsa6331 | D00643 | hsa2557 | D01740 | hsa2556 | D04721 | hsa1141 | D08935 |
| hsa6334 | D00643 | hsa2558 | D01740 | hsa2557 | D04721 | hsa2890 | D08964 |
| hsa6335 | D00643 | hsa2559 | D01740 | hsa2558 | D04721 | hsa2891 | D08964 |
| hsa6336 | D00643 | hsa2560 | D01740 | hsa2559 | D04721 | hsa2892 | D08964 |
| hsa3757 | D00647 | hsa2561 | D01740 | hsa2560 | D04721 | hsa2893 | D08964 |
| hsa3757 | D00648 | hsa2562 | D01740 | hsa2561 | D04721 | hsa1137 | D08987 |
| hsa6337 | D00649 | hsa2563 | D01740 | hsa2562 | D04721 | hsa1141 | D08987 |
| hsa6338 | D00649 | hsa2564 | D01740 | hsa2563 | D04721 | hsa2890 | D09035 |
| hsa6340 | D00649 | hsa2565 | D01740 | hsa2564 | D04721 | hsa2891 | D09035 |
| hsa9177 | D00677 | hsa2566 | D01740 | hsa2565 | D04721 | hsa2892 | D09035 |
| hsa170572 | D00678 | hsa2567 | D01740 | hsa2566 | D04721 | hsa2893 | D09035 |
| hsa200909 | D00678 | hsa2568 | D01740 | hsa2567 | D04721 | hsa11280 | D09215 |
| hsa285242 | D00678 | hsa55879 | D01740 | hsa2568 | D04721 | hsa6323 | D09215 |
| hsa3359 | D00678 | hsa2554 | D01744 | hsa55879 | D04721 | hsa6326 | D09215 |
| hsa9177 | D00678 | hsa2555 | D01744 | hsa2902 | D04728 | hsa6328 | D09215 |
| hsa2554 | D00693 | hsa2556 | D01744 | hsa2903 | D04728 | hsa6329 | D09215 |
| hsa2555 | D00693 | hsa2557 | D01744 | hsa2904 | D04728 | hsa6331 | D09215 |
| hsa2556 | D00693 | hsa2558 | D01744 | hsa2905 | D04728 | hsa6334 | D09215 |
| hsa2557 | D00693 | hsa2559 | D01744 | hsa2906 | D04728 | hsa6335 | D09215 |
| hsa2558 | D00693 | hsa2560 | D01744 | hsa11280 | D04774 | hsa6336 | D09215 |
| hsa2559 | D00693 | hsa2561 | D01744 | hsa6323 | D04774 | hsa1137 | D09367 |
| hsa2560 | D00693 | hsa2562 | D01744 | hsa6326 | D04774 | hsa1141 | D09367 |
| hsa2561 | D00693 | hsa2563 | D01744 | hsa6328 | D04774 | hsa1137 | D09368 |
| hsa2562 | D00693 | hsa2564 | D01744 | hsa6329 | D04774 | hsa1141 | D09368 |
| hsa2563 | D00693 | hsa2565 | D01744 | hsa6331 | D04774 | hsa1137 | D09382 |
| hsa2564 | D00693 | hsa2566 | D01744 | hsa6334 | D04774 | hsa1141 | D09382 |
| hsa2565 | D00693 | hsa2567 | D01744 | hsa6335 | D04774 | hsa1137 | D09383 |
| hsa2566 | D00693 | hsa2568 | D01744 | hsa6336 | D04774 | hsa1141 | D09383 |
| hsa2567 | D00693 | hsa55879 | D01744 | hsa1181 | D04790 | hsa55799 | D09539 |
| hsa2568 | D00693 | hsa2554 | D01758 | hsa79054 | D04849 | hsa781 | D09539 |
| hsa55879 | D00693 | hsa2555 | D01758 | hsa8989 | D04849 | hsa9254 | D09539 |
| hsa2554 | D00694 | hsa2556 | D01758 | hsa2554 | D04882 | hsa93589 | D09539 |
| hsa2555 | D00694 | hsa2557 | D01758 | hsa2555 | D04882 | hsa3784 | D09569 |
| hsa2556 | D00694 | hsa2558 | D01758 | hsa2556 | D04882 | hsa3785 | D09569 |
| hsa2557 | D00694 | hsa2559 | D01758 | hsa2557 | D04882 | hsa3786 | D09569 |
| hsa2558 | D00694 | hsa2560 | D01758 | hsa2558 | D04882 | hsa56479 | D09569 |
| hsa2559 | D00694 | hsa2561 | D01758 | hsa2559 | D04882 | hsa9132 | D09569 |
| hsa2560 | D00694 | hsa2562 | D01758 | hsa2560 | D04882 | hsa11280 | D09612 |
| hsa2561 | D00694 | hsa2563 | D01758 | hsa2561 | D04882 | hsa6323 | D09612 |
| hsa2562 | D00694 | hsa2564 | D01758 | hsa2562 | D04882 | hsa6326 | D09612 |
| hsa2563 | D00694 | hsa2565 | D01758 | hsa2563 | D04882 | hsa6328 | D09612 |
| hsa2564 | D00694 | hsa2566 | D01758 | hsa2564 | D04882 | hsa6329 | D09612 |
| hsa2565 | D00694 | hsa2567 | D01758 | hsa2565 | D04882 | hsa6331 | D09612 |
| hsa2566 | D00694 | hsa2568 | D01758 | hsa2566 | D04882 | hsa6334 | D09612 |
| hsa2567 | D00694 | hsa55879 | D01758 | hsa2567 | D04882 | hsa6335 | D09612 |
| hsa2568 | D00694 | hsa11280 | D01768 | hsa2568 | D04882 | hsa6336 | D09612 |
| hsa55879 | D00694 | hsa6323 | D01768 | hsa55879 | D04882 | hsa170572 | D09749 |
| hsa2554 | D00695 | hsa6326 | D01768 | hsa2902 | D04905 | hsa200909 | D09749 |
| hsa2555 | D00695 | hsa6328 | D01768 | hsa2903 | D04905 | hsa285242 | D09749 |
| hsa2556 | D00695 | hsa6329 | D01768 | hsa2904 | D04905 | hsa3359 | D09749 |
| hsa2557 | D00695 | hsa6331 | D01768 | hsa2905 | D04905 | hsa9177 | D09749 |
| hsa2558 | D00695 | hsa6334 | D01768 | hsa2906 | D04905 | hsa3757 | D09757 |
| hsa2559 | D00695 | hsa6335 | D01768 | hsa79054 | D04918 | hsa3768 | D09757 |
| hsa2560 | D00695 | hsa6336 | D01768 | hsa8989 | D04918 | hsa3784 | D09757 |
| hsa2561 | D00695 | hsa11280 | D01785 | hsa3757 | D04955 | hsa3757 | D09758 |
| hsa2562 | D00695 | hsa6323 | D01785 | hsa2554 | D04985 | hsa3784 | D09758 |
| hsa2563 | D00695 | hsa6326 | D01785 | hsa775 | D05024 | hsa3757 | D09759 |
| hsa2564 | D00695 | hsa6328 | D01785 | hsa776 | D05024 | hsa775 | D09789 |
| hsa2565 | D00695 | hsa6329 | D01785 | hsa778 | D05024 | hsa776 | D09789 |
| hsa2566 | D00695 | hsa6331 | D01785 | hsa779 | D05024 | hsa778 | D09789 |
| hsa2567 | D00695 | hsa6334 | D01785 | hsa8911 | D05024 | hsa779 | D09789 |
| hsa2568 | D00695 | hsa6335 | D01785 | hsa8912 | D05024 | hsa2902 | D09917 |
| hsa55879 | D00695 | hsa6336 | D01785 | hsa8913 | D05024 | hsa2903 | D09917 |
| hsa2554 | D00696 | hsa3767 | D01810 | hsa2554 | D05028 | hsa2904 | D09917 |
| hsa2555 | D00696 | hsa170572 | D01838 | hsa2555 | D05028 | hsa2905 | D09917 |
| hsa2556 | D00696 | hsa200909 | D01838 | hsa2556 | D05028 | hsa2906 | D09917 |
| hsa2557 | D00696 | hsa285242 | D01838 | hsa2557 | D05028 | hsa2902 | D09918 |
| hsa2558 | D00696 | hsa3359 | D01838 | hsa2558 | D05028 | hsa2903 | D09918 |
| hsa2559 | D00696 | hsa9177 | D01838 | hsa2559 | D05028 | hsa2904 | D09918 |
| hsa2560 | D00696 | hsa775 | D01849 | hsa2560 | D05028 | hsa2905 | D09918 |
| hsa2561 | D00696 | hsa776 | D01849 | hsa2561 | D05028 | hsa2906 | D09918 |
| hsa2562 | D00696 | hsa778 | D01849 | hsa2562 | D05028 | hsa2890 | D09931 |
| hsa2563 | D00696 | hsa779 | D01849 | hsa2563 | D05028 | hsa2891 | D09931 |
| hsa2564 | D00696 | hsa3767 | D01854 | hsa2564 | D05028 | hsa2892 | D09931 |
| hsa2565 | D00696 | hsa3752 | D01856 | hsa2565 | D05028 | hsa2893 | D09931 |
| hsa2566 | D00696 | hsa3757 | D01856 | hsa2566 | D05028 | hsa1137 | D10111 |
| hsa2567 | D00696 | hsa3768 | D01856 | hsa2567 | D05028 | hsa1141 | D10111 |
| hsa2568 | D00696 | hsa775 | D01908 | hsa2568 | D05028 | hsa1137 | D10112 |
| hsa55879 | D00696 | hsa776 | D01908 | hsa55879 | D05028 | hsa1141 | D10112 |
| hsa2554 | D00697 | hsa778 | D01908 | hsa6331 | D05077 | hsa170572 | D10170 |
| hsa2555 | D00697 | hsa779 | D01908 | hsa2902 | D05145 | hsa200909 | D10170 |
| hsa2556 | D00697 | hsa775 | D01933 | hsa2903 | D05145 | hsa285242 | D10170 |
| hsa2557 | D00697 | hsa776 | D01933 | hsa2904 | D05145 | hsa3359 | D10170 |
| hsa2558 | D00697 | hsa778 | D01933 | hsa2905 | D05145 | hsa9177 | D10170 |
| hsa2559 | D00697 | hsa779 | D01933 | hsa2906 | D05145 | hsa170572 | D10171 |
| hsa2560 | D00697 | hsa11280 | D01967 | hsa1134 | D05156 | hsa200909 | D10171 |
| hsa2561 | D00697 | hsa6323 | D01967 | hsa1135 | D05156 | hsa285242 | D10171 |
| hsa2562 | D00697 | hsa6326 | D01967 | hsa1136 | D05156 | hsa3359 | D10171 |
| hsa2563 | D00697 | hsa6328 | D01967 | hsa1137 | D05156 | hsa9177 | D10171 |
| hsa2564 | D00697 | hsa6329 | D01967 | hsa1138 | D05156 | hsa170572 | D10184 |
| hsa2565 | D00697 | hsa6331 | D01967 | hsa1139 | D05156 | hsa200909 | D10184 |
| hsa2566 | D00697 | hsa6334 | D01967 | hsa1140 | D05156 | hsa285242 | D10184 |
| hsa2567 | D00697 | hsa6335 | D01967 | hsa1141 | D05156 | hsa3359 | D10184 |
| hsa2568 | D00697 | hsa6336 | D01967 | hsa1142 | D05156 | hsa9177 | D10184 |
| hsa55879 | D00697 | hsa775 | D01969 | hsa1143 | D05156 | hsa170572 | D10185 |
| hsa2554 | D00698 | hsa776 | D01969 | hsa1144 | D05156 | hsa200909 | D10185 |
| hsa2555 | D00698 | hsa778 | D01969 | hsa1145 | D05156 | hsa285242 | D10185 |
| hsa2556 | D00698 | hsa779 | D01969 | hsa1146 | D05156 | hsa3359 | D10185 |
| hsa2557 | D00698 | hsa7224 | D02013 | hsa55584 | D05156 | hsa9177 | D10185 |
| hsa2558 | D00698 | hsa170572 | D02016 | hsa57053 | D05156 | hsa2554 | D10194 |
| hsa2559 | D00698 | hsa200909 | D02016 | hsa8973 | D05156 | hsa2555 | D10194 |
| hsa2560 | D00698 | hsa285242 | D02016 | hsa1134 | D05157 | hsa2556 | D10194 |
| hsa2561 | D00698 | hsa3359 | D02016 | hsa1135 | D05157 | hsa2557 | D10194 |
| hsa2562 | D00698 | hsa9177 | D02016 | hsa1136 | D05157 | hsa2558 | D10194 |
| hsa2563 | D00698 | hsa170572 | D02041 | hsa1137 | D05157 | hsa2559 | D10194 |
| hsa2564 | D00698 | hsa200909 | D02041 | hsa1138 | D05157 | hsa2560 | D10194 |
| hsa2565 | D00698 | hsa285242 | D02041 | hsa1139 | D05157 | hsa2561 | D10194 |
| hsa2566 | D00698 | hsa3359 | D02041 | hsa1140 | D05157 | hsa2562 | D10194 |
| hsa2567 | D00698 | hsa9177 | D02041 | hsa1141 | D05157 | hsa2563 | D10194 |
| hsa2568 | D00698 | hsa775 | D02045 | hsa1142 | D05157 | hsa2564 | D10194 |
| hsa55879 | D00698 | hsa776 | D02045 | hsa1143 | D05157 | hsa2565 | D10194 |
| hsa2554 | D00699 | hsa778 | D02045 | hsa1144 | D05157 | hsa2566 | D10194 |
| hsa2555 | D00699 | hsa779 | D02045 | hsa1145 | D05157 | hsa2567 | D10194 |
| hsa2556 | D00699 | hsa11280 | D02086 | hsa1146 | D05157 | hsa2568 | D10194 |
| hsa2557 | D00699 | hsa6323 | D02086 | hsa55584 | D05157 | hsa55879 | D10194 |
| hsa2558 | D00699 | hsa6326 | D02086 | hsa57053 | D05157 | hsa6261 | D10304 |
| hsa2559 | D00699 | hsa6328 | D02086 | hsa8973 | D05157 | hsa6262 | D10304 |
| hsa2560 | D00699 | hsa6329 | D02086 | hsa7442 | D05249 | hsa6263 | D10304 |
| hsa2561 | D00699 | hsa6331 | D02086 | hsa170572 | D05343 | hsa6261 | D10305 |
| hsa2562 | D00699 | hsa6334 | D02086 | hsa200909 | D05343 | hsa6262 | D10305 |
| hsa2563 | D00699 | hsa6335 | D02086 | hsa285242 | D05343 | hsa6263 | D10305 |
| hsa2564 | D00699 | hsa6336 | D02086 | hsa3359 | D05343 | hsa7442 | D10370 |
| hsa2565 | D00699 | hsa6331 | D02087 | hsa9177 | D05343 | hsa2741 | D10470 |
| hsa2566 | D00699 | hsa11280 | D02088 | hsa170572 | D05348 | hsa1139 | D10613 |
| hsa2567 | D00699 | hsa6323 | D02088 | hsa200909 | D05348 | hsa1139 | D10626 |
| hsa2568 | D00699 | hsa6326 | D02088 | hsa285242 | D05348 | hsa1139 | D10693 |
| hsa55879 | D00699 | hsa6328 | D02088 | hsa3359 | D05348 | hsa1139 | D10702 |
| hsa2554 | D00700 | hsa6329 | D02088 | hsa9177 | D05348 | hsa2890 | D10780 |
| hsa2555 | D00700 | hsa6331 | D02088 | hsa775 | D05442 | hsa2891 | D10780 |
| hsa2556 | D00700 | hsa6334 | D02088 | hsa776 | D05442 | hsa2892 | D10780 |
| hsa2557 | D00700 | hsa6335 | D02088 | hsa778 | D05442 | hsa2893 | D10780 |
| hsa2558 | D00700 | hsa6336 | D02088 | hsa779 | D05442 | hsa1139 | D10789 |
| hsa2559 | D00700 | hsa3359 | D02092 | hsa2902 | D05447 | hsa781 | D10790 |
| hsa2560 | D00700 | hsa11280 | D02096 | hsa2903 | D05447 | hsa9254 | D10790 |
| hsa2561 | D00700 | hsa6323 | D02096 | hsa2904 | D05447 | hsa5027 | D10799 |
| hsa2562 | D00700 | hsa6326 | D02096 | hsa2905 | D05447 | hsa775 | D10932 |
| hsa2563 | D00700 | hsa6328 | D02096 | hsa2906 | D05447 | hsa776 | D10932 |
| hsa2564 | D00700 | hsa6329 | D02096 | hsa2902 | D05453 | hsa778 | D10932 |
| hsa2565 | D00700 | hsa6331 | D02096 | hsa2903 | D05453 | hsa779 | D10932 |
| hsa2566 | D00700 | hsa6334 | D02096 | hsa2904 | D05453 | hsa170572 | D11056 |
| hsa2567 | D00700 | hsa6335 | D02096 | hsa2905 | D05453 | hsa200909 | D11056 |
| hsa2568 | D00700 | hsa6336 | D02096 | hsa2906 | D05453 | hsa285242 | D11056 |
| hsa55879 | D00700 | hsa11280 | D02098 | hsa3767 | D05482 | hsa3359 | D11056 |
| hsa2554 | D00701 | hsa6323 | D02098 | hsa2554 | D05621 | hsa9177 | D11056 |
| hsa2555 | D00701 | hsa6326 | D02098 |  |  |  |  |

Table3. The overall of drug-target interactions for GPCRs class on new dataset 1

| **drug** | **target** | **drug** | **target** | **drug** | **target** | **drug** | **target** |
| --- | --- | --- | --- | --- | --- | --- | --- |
| hsa134 | D00045 | hsa5739 | D01551 | hsa152 | D04375 | hsa5739 | D08065 |
| hsa135 | D00045 | hsa146 | D01571 | hsa2798 | D04405 | hsa185 | D08067 |
| hsa136 | D00045 | hsa147 | D01571 | hsa2492 | D04429 | hsa153 | D08074 |
| hsa140 | D00045 | hsa148 | D01571 | hsa2492 | D04430 | hsa154 | D08074 |
| hsa2550 | D00058 | hsa146 | D01573 | hsa1128 | D04434 | hsa155 | D08074 |
| hsa9568 | D00058 | hsa147 | D01573 | hsa1129 | D04434 | hsa153 | D08078 |
| hsa1812 | D00059 | hsa148 | D01573 | hsa1131 | D04434 | hsa154 | D08078 |
| hsa1813 | D00059 | hsa150 | D01573 | hsa1132 | D04434 | hsa153 | D08090 |
| hsa1814 | D00059 | hsa151 | D01573 | hsa1133 | D04434 | hsa154 | D08090 |
| hsa1815 | D00059 | hsa152 | D01573 | hsa11255 | D04445 | hsa155 | D08090 |
| hsa1816 | D00059 | hsa153 | D01573 | hsa3269 | D04445 | hsa3269 | D08091 |
| hsa146 | D00076 | hsa154 | D01573 | hsa3274 | D04445 | hsa154 | D08092 |
| hsa147 | D00076 | hsa155 | D01573 | hsa4988 | D04461 | hsa154 | D08093 |
| hsa148 | D00076 | hsa154 | D01589 | hsa1128 | D04479 | hsa1813 | D08094 |
| hsa150 | D00076 | hsa11255 | D01592 | hsa1129 | D04479 | hsa3356 | D08099 |
| hsa151 | D00076 | hsa3269 | D01592 | hsa1131 | D04479 | hsa4985 | D08100 |
| hsa152 | D00076 | hsa1128 | D01600 | hsa1132 | D04479 | hsa4986 | D08100 |
| hsa153 | D00076 | hsa1129 | D01600 | hsa1133 | D04479 | hsa4988 | D08100 |
| hsa154 | D00076 | hsa1131 | D01600 | hsa1812 | D04488 | hsa4985 | D08101 |
| hsa155 | D00076 | hsa1132 | D01600 | hsa1813 | D04488 | hsa4986 | D08101 |
| hsa5732 | D00079 | hsa1133 | D01600 | hsa624 | D04492 | hsa4988 | D08101 |
| hsa5737 | D00081 | hsa150 | D01603 | hsa3269 | D04494 | hsa3269 | D08105 |
| hsa5021 | D00089 | hsa151 | D01603 | hsa3274 | D04494 | hsa146 | D08106 |
| hsa146 | D00095 | hsa152 | D01603 | hsa2862 | D04497 | hsa147 | D08106 |
| hsa147 | D00095 | hsa3356 | D01624 | hsa6915 | D04500 | hsa148 | D08106 |
| hsa148 | D00095 | hsa3269 | D01627 | hsa6915 | D04501 | hsa153 | D08106 |
| hsa150 | D00095 | hsa887 | D01631 | hsa151 | D04514 | hsa154 | D08106 |
| hsa151 | D00095 | hsa154 | D01635 | hsa3274 | D04517 | hsa2798 | D08113 |
| hsa152 | D00095 | hsa1128 | D01648 | hsa146 | D04531 | hsa146 | D08115 |
| hsa153 | D00095 | hsa1129 | D01648 | hsa147 | D04531 | hsa147 | D08115 |
| hsa154 | D00095 | hsa1131 | D01648 | hsa148 | D04531 | hsa148 | D08115 |
| hsa155 | D00095 | hsa1132 | D01648 | hsa146 | D04532 | hsa153 | D08115 |
| hsa552 | D00101 | hsa1133 | D01648 | hsa147 | D04532 | hsa154 | D08115 |
| hsa553 | D00101 | hsa5724 | D01652 | hsa148 | D04532 | hsa155 | D08115 |
| hsa554 | D00101 | hsa3269 | D01654 | hsa3350 | D04533 | hsa3269 | D08117 |
| hsa5739 | D00106 | hsa146 | D01674 | hsa3351 | D04533 | hsa3269 | D08118 |
| hsa1128 | D00113 | hsa147 | D01674 | hsa3352 | D04533 | hsa4988 | D08121 |
| hsa1129 | D00113 | hsa148 | D01674 | hsa3354 | D04533 | hsa4988 | D08122 |
| hsa1131 | D00113 | hsa2798 | D01685 | hsa3355 | D04533 | hsa4988 | D08123 |
| hsa1132 | D00113 | hsa146 | D01691 | hsa3350 | D04611 | hsa154 | D08124 |
| hsa1133 | D00113 | hsa147 | D01691 | hsa154 | D04625 | hsa150 | D08126 |
| hsa2642 | D00116 | hsa148 | D01691 | hsa154 | D04626 | hsa151 | D08126 |
| hsa146 | D00124 | hsa153 | D01691 | hsa135 | D04641 | hsa152 | D08126 |
| hsa147 | D00124 | hsa154 | D01691 | hsa2798 | D04644 | hsa1813 | D08132 |
| hsa148 | D00124 | hsa155 | D01691 | hsa4986 | D04649 | hsa1814 | D08132 |
| hsa150 | D00124 | hsa148 | D01692 | hsa6752 | D04666 | hsa1815 | D08132 |
| hsa151 | D00124 | hsa3274 | D01698 | hsa6755 | D04666 | hsa150 | D08141 |
| hsa152 | D00124 | hsa1131 | D01699 | hsa3274 | D04680 | hsa151 | D08141 |
| hsa153 | D00124 | hsa134 | D01712 | hsa3350 | D04683 | hsa152 | D08141 |
| hsa154 | D00124 | hsa136 | D01712 | hsa1813 | D04693 | hsa4985 | D08144 |
| hsa155 | D00124 | hsa3269 | D01713 | hsa1813 | D04694 | hsa4986 | D08144 |
| hsa1813 | D00136 | hsa3269 | D01717 | hsa154 | D04701 | hsa4988 | D08144 |
| hsa1128 | D00138 | hsa153 | D01741 | hsa154 | D04703 | hsa185 | D08146 |
| hsa1129 | D00138 | hsa1813 | D01742 | hsa153 | D04708 | hsa1813 | D08148 |
| hsa1131 | D00138 | hsa1813 | D01745 | hsa153 | D04710 | hsa3356 | D08148 |
| hsa1132 | D00138 | hsa154 | D01748 | hsa4988 | D04716 | hsa1128 | D08157 |
| hsa1133 | D00138 | hsa134 | D01771 | hsa1268 | D04717 | hsa1129 | D08157 |
| hsa4158 | D00146 | hsa135 | D01771 | hsa5724 | D04724 | hsa1131 | D08157 |
| hsa1128 | D00147 | hsa136 | D01771 | hsa554 | D04752 | hsa1132 | D08157 |
| hsa1129 | D00147 | hsa3269 | D01782 | hsa4988 | D04764 | hsa1133 | D08157 |
| hsa1131 | D00147 | hsa3269 | D01786 | hsa150 | D04765 | hsa1128 | D08160 |
| hsa1132 | D00147 | hsa153 | D01794 | hsa151 | D04765 | hsa1129 | D08160 |
| hsa1133 | D00147 | hsa154 | D01794 | hsa152 | D04765 | hsa1131 | D08160 |
| hsa185 | D00150 | hsa155 | D01794 | hsa3356 | D04788 | hsa1132 | D08160 |
| hsa186 | D00150 | hsa153 | D01795 | hsa3274 | D04794 | hsa1133 | D08160 |
| hsa7201 | D00176 | hsa154 | D01795 | hsa1813 | D04820 | hsa3269 | D08161 |
| hsa5731 | D00180 | hsa155 | D01795 | hsa3356 | D04820 | hsa3269 | D08163 |
| hsa1131 | D00193 | hsa3269 | D01801 | hsa2798 | D04823 | hsa150 | D08165 |
| hsa4988 | D00195 | hsa153 | D01806 | hsa3973 | D04824 | hsa151 | D08165 |
| hsa134 | D00227 | hsa154 | D01806 | hsa6869 | D04860 | hsa152 | D08165 |
| hsa136 | D00227 | hsa155 | D01806 | hsa1128 | D04868 | hsa4543 | D08170 |
| hsa1128 | D00232 | hsa1128 | D01815 | hsa1129 | D04868 | hsa4544 | D08170 |
| hsa1129 | D00232 | hsa1129 | D01815 | hsa1131 | D04868 | hsa1813 | D08172 |
| hsa1131 | D00232 | hsa1131 | D01815 | hsa1132 | D04868 | hsa4988 | D08182 |
| hsa1132 | D00232 | hsa1132 | D01815 | hsa1133 | D04868 | hsa3269 | D08183 |
| hsa1133 | D00232 | hsa1133 | D01815 | hsa150 | D04883 | hsa146 | D08192 |
| hsa3269 | D00234 | hsa887 | D01818 | hsa151 | D04883 | hsa147 | D08192 |
| hsa153 | D00235 | hsa146 | D01830 | hsa152 | D04883 | hsa148 | D08192 |
| hsa2550 | D00241 | hsa147 | D01830 | hsa146 | D04888 | hsa150 | D08192 |
| hsa9568 | D00241 | hsa148 | D01830 | hsa147 | D04888 | hsa151 | D08192 |
| hsa799 | D00249 | hsa153 | D01830 | hsa148 | D04888 | hsa152 | D08192 |
| hsa146 | D00255 | hsa154 | D01830 | hsa153 | D04888 | hsa4988 | D08195 |
| hsa147 | D00255 | hsa2798 | D01831 | hsa154 | D04888 | hsa146 | D08201 |
| hsa148 | D00255 | hsa154 | D01835 | hsa155 | D04888 | hsa147 | D08201 |
| hsa153 | D00255 | hsa3360 | D01838 | hsa146 | D04889 | hsa148 | D08201 |
| hsa154 | D00255 | hsa153 | D01847 | hsa147 | D04889 | hsa150 | D08205 |
| hsa1128 | D00270 | hsa554 | D01855 | hsa148 | D04889 | hsa151 | D08205 |
| hsa1129 | D00270 | hsa886 | D01858 | hsa153 | D04889 | hsa152 | D08205 |
| hsa1131 | D00270 | hsa887 | D01858 | hsa154 | D04889 | hsa146 | D08206 |
| hsa1132 | D00270 | hsa1813 | D01860 | hsa155 | D04889 | hsa147 | D08206 |
| hsa1133 | D00270 | hsa3274 | D01861 | hsa4988 | D04924 | hsa148 | D08206 |
| hsa150 | D00270 | hsa5731 | D01869 | hsa154 | D04954 | hsa150 | D08206 |
| hsa151 | D00270 | hsa1128 | D01871 | hsa1813 | D04965 | hsa151 | D08206 |
| hsa152 | D00270 | hsa1129 | D01871 | hsa1128 | D04970 | hsa152 | D08206 |
| hsa1813 | D00270 | hsa1131 | D01871 | hsa1129 | D04970 | hsa153 | D08206 |
| hsa3269 | D00270 | hsa1132 | D01871 | hsa1131 | D04970 | hsa154 | D08206 |
| hsa3356 | D00270 | hsa1133 | D01871 | hsa1132 | D04970 | hsa155 | D08206 |
| hsa3357 | D00270 | hsa1131 | D01875 | hsa1133 | D04970 | hsa146 | D08207 |
| hsa3358 | D00270 | hsa4986 | D01879 | hsa4988 | D04973 | hsa147 | D08207 |
| hsa3356 | D00274 | hsa4988 | D01879 | hsa3269 | D04979 | hsa148 | D08207 |
| hsa3360 | D00274 | hsa146 | D01887 | hsa3269 | D04980 | hsa150 | D08207 |
| hsa150 | D00281 | hsa147 | D01887 | hsa3274 | D05004 | hsa151 | D08207 |
| hsa151 | D00281 | hsa148 | D01887 | hsa4985 | D05007 | hsa152 | D08207 |
| hsa152 | D00281 | hsa5732 | D01891 | hsa1813 | D05008 | hsa1128 | D08209 |
| hsa150 | D00283 | hsa5733 | D01891 | hsa1813 | D05010 | hsa1129 | D08209 |
| hsa151 | D00283 | hsa6915 | D01896 | hsa153 | D05011 | hsa1131 | D08209 |
| hsa152 | D00283 | hsa1813 | D01898 | hsa1128 | D05012 | hsa1132 | D08209 |
| hsa1815 | D00283 | hsa3269 | D01902 | hsa1129 | D05012 | hsa1133 | D08209 |
| hsa3356 | D00283 | hsa3356 | D01902 | hsa1131 | D05012 | hsa146 | D08212 |
| hsa3358 | D00283 | hsa3357 | D01902 | hsa1132 | D05012 | hsa147 | D08212 |
| hsa4158 | D00284 | hsa3358 | D01902 | hsa1133 | D05012 | hsa148 | D08212 |
| hsa554 | D00291 | hsa153 | D01903 | hsa3356 | D05015 | hsa150 | D08216 |
| hsa3274 | D00295 | hsa1812 | D01903 | hsa3357 | D05015 | hsa151 | D08216 |
| hsa3269 | D00300 | hsa153 | D01906 | hsa3358 | D05015 | hsa152 | D08216 |
| hsa1268 | D00306 | hsa154 | D01906 | hsa1128 | D05035 | hsa146 | D08220 |
| hsa1269 | D00306 | hsa155 | D01906 | hsa1129 | D05035 | hsa147 | D08220 |
| hsa146 | D00308 | hsa185 | D01922 | hsa1131 | D05035 | hsa148 | D08220 |
| hsa147 | D00308 | hsa7201 | D01925 | hsa1132 | D05035 | hsa1813 | D08226 |
| hsa148 | D00308 | hsa1131 | D01929 | hsa1133 | D05035 | hsa10800 | D08229 |
| hsa1813 | D00308 | hsa1813 | D01939 | hsa154 | D05037 | hsa4988 | D08233 |
| hsa3274 | D00318 | hsa3356 | D01939 | hsa4988 | D05048 | hsa4988 | D08234 |
| hsa4988 | D00320 | hsa1128 | D01946 | hsa2862 | D05053 | hsa1813 | D08235 |
| hsa5737 | D00356 | hsa1129 | D01946 | hsa1241 | D05085 | hsa1814 | D08235 |
| hsa185 | D00357 | hsa1131 | D01946 | hsa56413 | D05085 | hsa1815 | D08235 |
| hsa3269 | D00364 | hsa1132 | D01946 | hsa150 | D05087 | hsa3360 | D08236 |
| hsa552 | D00366 | hsa1133 | D01946 | hsa151 | D05087 | hsa146 | D08239 |
| hsa553 | D00366 | hsa146 | D01952 | hsa152 | D05087 | hsa147 | D08239 |
| hsa554 | D00366 | hsa147 | D01952 | hsa1268 | D05099 | hsa148 | D08239 |
| hsa134 | D00371 | hsa148 | D01952 | hsa1269 | D05099 | hsa2798 | D08241 |
| hsa136 | D00371 | hsa150 | D01952 | hsa3356 | D05107 | hsa3356 | D08244 |
| hsa1813 | D00373 | hsa151 | D01952 | hsa4985 | D05111 | hsa4985 | D08246 |
| hsa1812 | D00374 | hsa152 | D01952 | hsa4986 | D05111 | hsa4986 | D08246 |
| hsa1813 | D00374 | hsa153 | D01952 | hsa4988 | D05111 | hsa4988 | D08246 |
| hsa153 | D00378 | hsa154 | D01952 | hsa4985 | D05113 | hsa4986 | D08247 |
| hsa154 | D00378 | hsa155 | D01952 | hsa4986 | D05113 | hsa4988 | D08247 |
| hsa1813 | D00390 | hsa1128 | D01955 | hsa4988 | D05113 | hsa4986 | D08248 |
| hsa1128 | D00397 | hsa1129 | D01955 | hsa1813 | D05124 | hsa4988 | D08248 |
| hsa1129 | D00397 | hsa1131 | D01955 | hsa153 | D05127 | hsa4985 | D08249 |
| hsa1131 | D00397 | hsa1132 | D01955 | hsa6869 | D05152 | hsa4986 | D08249 |
| hsa1132 | D00397 | hsa1133 | D01955 | hsa3269 | D05196 | hsa4988 | D08249 |
| hsa1133 | D00397 | hsa153 | D01958 | hsa146 | D05206 | hsa146 | D08253 |
| hsa185 | D00400 | hsa154 | D01958 | hsa147 | D05206 | hsa147 | D08253 |
| hsa1813 | D00403 | hsa5737 | D01964 | hsa148 | D05206 | hsa148 | D08253 |
| hsa150 | D00405 | hsa148 | D01965 | hsa150 | D05206 | hsa3351 | D08255 |
| hsa151 | D00405 | hsa3351 | D01973 | hsa151 | D05206 | hsa3352 | D08255 |
| hsa152 | D00405 | hsa3352 | D01973 | hsa152 | D05206 | hsa146 | D08257 |
| hsa10800 | D00411 | hsa1128 | D01976 | hsa153 | D05206 | hsa147 | D08257 |
| hsa3351 | D00415 | hsa1129 | D01976 | hsa154 | D05206 | hsa148 | D08257 |
| hsa3352 | D00415 | hsa1131 | D01976 | hsa155 | D05206 | hsa3356 | D08257 |
| hsa5732 | D00419 | hsa1132 | D01976 | hsa4988 | D05217 | hsa3358 | D08257 |
| hsa5733 | D00419 | hsa1133 | D01976 | hsa146 | D05228 | hsa146 | D08271 |
| hsa5734 | D00419 | hsa154 | D01978 | hsa147 | D05228 | hsa147 | D08271 |
| hsa3274 | D00422 | hsa1128 | D01987 | hsa148 | D05228 | hsa148 | D08271 |
| hsa1813 | D00426 | hsa1129 | D01987 | hsa150 | D05228 | hsa4988 | D08273 |
| hsa3356 | D00426 | hsa1131 | D01987 | hsa151 | D05228 | hsa146 | D08284 |
| hsa153 | D00432 | hsa1132 | D01987 | hsa152 | D05228 | hsa147 | D08284 |
| hsa154 | D00432 | hsa1133 | D01987 | hsa153 | D05228 | hsa148 | D08284 |
| hsa3274 | D00440 | hsa3350 | D01992 | hsa154 | D05228 | hsa150 | D08284 |
| hsa6752 | D00442 | hsa3360 | D01994 | hsa155 | D05228 | hsa151 | D08284 |
| hsa3351 | D00451 | hsa1812 | D02004 | hsa6752 | D05230 | hsa152 | D08284 |
| hsa3352 | D00451 | hsa1813 | D02004 | hsa185 | D05246 | hsa153 | D08284 |
| hsa146 | D00454 | hsa7201 | D02007 | hsa2492 | D05258 | hsa154 | D08284 |
| hsa147 | D00454 | hsa185 | D02014 | hsa3973 | D05258 | hsa155 | D08284 |
| hsa148 | D00454 | hsa186 | D02014 | hsa1128 | D05276 | hsa3269 | D08293 |
| hsa1813 | D00454 | hsa134 | D02017 | hsa1129 | D05276 | hsa1813 | D08297 |
| hsa1814 | D00454 | hsa135 | D02017 | hsa1131 | D05276 | hsa3269 | D08297 |
| hsa1815 | D00454 | hsa6344 | D02021 | hsa1132 | D05276 | hsa3356 | D08297 |
| hsa3269 | D00454 | hsa1813 | D02022 | hsa1133 | D05276 | hsa3357 | D08297 |
| hsa3356 | D00454 | hsa150 | D02034 | hsa154 | D05277 | hsa3358 | D08297 |
| hsa3357 | D00454 | hsa151 | D02034 | hsa3274 | D05306 | hsa154 | D08300 |
| hsa3358 | D00454 | hsa152 | D02034 | hsa3274 | D05307 | hsa3269 | D08305 |
| hsa3362 | D00454 | hsa1813 | D02035 | hsa4985 | D05312 | hsa153 | D08318 |
| hsa1813 | D00458 | hsa1813 | D02037 | hsa4986 | D05312 | hsa154 | D08318 |
| hsa3356 | D00458 | hsa2693 | D02040 | hsa4988 | D05312 | hsa155 | D08318 |
| hsa1128 | D00465 | hsa5739 | D02048 | hsa1813 | D05339 | hsa148 | D08322 |
| hsa1129 | D00465 | hsa1128 | D02064 | hsa3356 | D05339 | hsa4988 | D08323 |
| hsa1131 | D00465 | hsa1129 | D02064 | hsa1813 | D05340 | hsa1128 | D08325 |
| hsa1132 | D00465 | hsa1131 | D02064 | hsa3356 | D05340 | hsa1129 | D08325 |
| hsa1133 | D00465 | hsa1132 | D02064 | hsa153 | D05345 | hsa1131 | D08325 |
| hsa1813 | D00479 | hsa1133 | D02064 | hsa154 | D05345 | hsa1812 | D08339 |
| hsa3269 | D00480 | hsa153 | D02066 | hsa155 | D05345 | hsa1813 | D08339 |
| hsa1128 | D00481 | hsa154 | D02066 | hsa1128 | D05360 | hsa1813 | D08341 |
| hsa1129 | D00481 | hsa155 | D02066 | hsa1129 | D05360 | hsa1813 | D08342 |
| hsa1131 | D00481 | hsa3269 | D02067 | hsa1131 | D05360 | hsa4988 | D08343 |
| hsa1132 | D00481 | hsa1128 | D02069 | hsa1132 | D05360 | hsa3269 | D08353 |
| hsa1133 | D00481 | hsa1129 | D02069 | hsa1133 | D05360 | hsa3269 | D08355 |
| hsa4988 | D00482 | hsa1131 | D02069 | hsa5745 | D05364 | hsa146 | D08358 |
| hsa153 | D00483 | hsa1132 | D02069 | hsa5746 | D05364 | hsa147 | D08358 |
| hsa154 | D00483 | hsa1133 | D02069 | hsa154 | D05366 | hsa148 | D08358 |
| hsa155 | D00483 | hsa1128 | D02070 | hsa146 | D05395 | hsa150 | D08358 |
| hsa146 | D00485 | hsa1129 | D02070 | hsa147 | D05395 | hsa151 | D08358 |
| hsa147 | D00485 | hsa1131 | D02070 | hsa148 | D05395 | hsa152 | D08358 |
| hsa148 | D00485 | hsa1132 | D02070 | hsa3356 | D05395 | hsa146 | D08362 |
| hsa150 | D00485 | hsa1133 | D02070 | hsa3357 | D05395 | hsa147 | D08362 |
| hsa151 | D00485 | hsa1128 | D02071 | hsa3358 | D05395 | hsa148 | D08362 |
| hsa152 | D00485 | hsa1129 | D02071 | hsa1128 | D05418 | hsa150 | D08362 |
| hsa153 | D00485 | hsa1131 | D02071 | hsa1129 | D05418 | hsa151 | D08362 |
| hsa154 | D00485 | hsa1132 | D02071 | hsa1131 | D05418 | hsa152 | D08362 |
| hsa155 | D00485 | hsa1133 | D02071 | hsa1132 | D05418 | hsa146 | D08365 |
| hsa1813 | D00493 | hsa5731 | D02073 | hsa1133 | D05418 | hsa147 | D08365 |
| hsa3269 | D00494 | hsa150 | D02076 | hsa134 | D05429 | hsa148 | D08365 |
| hsa4986 | D00498 | hsa151 | D02076 | hsa136 | D05429 | hsa146 | D08366 |
| hsa4988 | D00498 | hsa152 | D02076 | hsa1128 | D05452 | hsa147 | D08366 |
| hsa1812 | D00502 | hsa153 | D02081 | hsa1129 | D05452 | hsa148 | D08366 |
| hsa1813 | D00502 | hsa154 | D02081 | hsa1131 | D05452 | hsa146 | D08368 |
| hsa1813 | D00503 | hsa155 | D02081 | hsa1132 | D05452 | hsa147 | D08368 |
| hsa146 | D00507 | hsa185 | D02082 | hsa1133 | D05452 | hsa148 | D08368 |
| hsa147 | D00507 | hsa3269 | D02089 | hsa4985 | D05462 | hsa150 | D08368 |
| hsa148 | D00507 | hsa3269 | D02090 | hsa4986 | D05462 | hsa151 | D08368 |
| hsa150 | D00507 | hsa3269 | D02091 | hsa4988 | D05462 | hsa152 | D08368 |
| hsa151 | D00507 | hsa3356 | D02092 | hsa146 | D05466 | hsa153 | D08368 |
| hsa152 | D00507 | hsa3360 | D02092 | hsa147 | D05466 | hsa154 | D08368 |
| hsa146 | D00509 | hsa154 | D02093 | hsa148 | D05466 | hsa155 | D08368 |
| hsa147 | D00509 | hsa4985 | D02095 | hsa150 | D05466 | hsa1129 | D08376 |
| hsa148 | D00509 | hsa4986 | D02095 | hsa151 | D05466 | hsa1131 | D08376 |
| hsa150 | D00509 | hsa4988 | D02095 | hsa152 | D05466 | hsa1128 | D08382 |
| hsa151 | D00509 | hsa1813 | D02100 | hsa153 | D05466 | hsa1129 | D08382 |
| hsa152 | D00509 | hsa3356 | D02100 | hsa154 | D05466 | hsa1131 | D08382 |
| hsa146 | D00511 | hsa4988 | D02101 | hsa155 | D05466 | hsa1132 | D08382 |
| hsa147 | D00511 | hsa4988 | D02102 | hsa3360 | D05471 | hsa1133 | D08382 |
| hsa148 | D00511 | hsa4985 | D02104 | hsa154 | D05476 | hsa1128 | D08384 |
| hsa153 | D00513 | hsa4986 | D02104 | hsa1129 | D05478 | hsa1129 | D08384 |
| hsa154 | D00513 | hsa4988 | D02104 | hsa1131 | D05478 | hsa1131 | D08384 |
| hsa155 | D00513 | hsa4158 | D02105 | hsa3350 | D05523 | hsa1132 | D08384 |
| hsa150 | D00514 | hsa6752 | D02108 | hsa3351 | D05523 | hsa1133 | D08384 |
| hsa151 | D00514 | hsa6753 | D02108 | hsa3352 | D05523 | hsa1812 | D08385 |
| hsa152 | D00514 | hsa6755 | D02108 | hsa3354 | D05523 | hsa1813 | D08385 |
| hsa3269 | D00520 | hsa146 | D02109 | hsa3355 | D05523 | hsa3350 | D08385 |
| hsa3269 | D00521 | hsa147 | D02109 | hsa3356 | D05523 | hsa3356 | D08385 |
| hsa185 | D00522 | hsa148 | D02109 | hsa3357 | D05523 | hsa154 | D08387 |
| hsa185 | D00523 | hsa150 | D02109 | hsa3358 | D05523 | hsa1128 | D08389 |
| hsa1128 | D00524 | hsa151 | D02109 | hsa1128 | D05535 | hsa1129 | D08389 |
| hsa1129 | D00524 | hsa152 | D02109 | hsa1129 | D05535 | hsa1131 | D08389 |
| hsa1131 | D00524 | hsa153 | D02109 | hsa1131 | D05535 | hsa1132 | D08389 |
| hsa1132 | D00524 | hsa154 | D02109 | hsa1132 | D05535 | hsa1133 | D08389 |
| hsa1133 | D00524 | hsa155 | D02109 | hsa1133 | D05535 | hsa1128 | D08395 |
| hsa1129 | D00525 | hsa4986 | D02111 | hsa1813 | D05575 | hsa1129 | D08395 |
| hsa1131 | D00525 | hsa4988 | D02111 | hsa1814 | D05575 | hsa1131 | D08395 |
| hsa134 | D00528 | hsa2798 | D02116 | hsa1815 | D05575 | hsa1132 | D08395 |
| hsa135 | D00528 | hsa2692 | D02117 | hsa153 | D05587 | hsa1133 | D08395 |
| hsa10800 | D00529 | hsa2642 | D02118 | hsa64805 | D05597 | hsa3350 | D08397 |
| hsa1131 | D00540 | hsa154 | D02147 | hsa2798 | D05599 | hsa3351 | D08397 |
| hsa1813 | D00559 | hsa3269 | D02148 | hsa2550 | D05621 | hsa3352 | D08397 |
| hsa1814 | D00559 | hsa146 | D02149 | hsa9568 | D05621 | hsa3354 | D08397 |
| hsa1815 | D00559 | hsa147 | D02149 | hsa1128 | D05628 | hsa3355 | D08397 |
| hsa1813 | D00560 | hsa148 | D02149 | hsa1129 | D05628 | hsa3356 | D08397 |
| hsa1814 | D00560 | hsa150 | D02149 | hsa1131 | D05628 | hsa3357 | D08397 |
| hsa1813 | D00561 | hsa151 | D02149 | hsa1132 | D05628 | hsa3358 | D08397 |
| hsa3356 | D00561 | hsa152 | D02149 | hsa1133 | D05628 | hsa10800 | D08408 |
| hsa150 | D00563 | hsa153 | D02149 | hsa4988 | D05632 | hsa146 | D08411 |
| hsa151 | D00563 | hsa154 | D02149 | hsa146 | D05637 | hsa147 | D08411 |
| hsa152 | D00563 | hsa155 | D02149 | hsa147 | D05637 | hsa148 | D08411 |
| hsa3356 | D00563 | hsa153 | D02150 | hsa148 | D05637 | hsa1128 | D08418 |
| hsa3357 | D00563 | hsa154 | D02150 | hsa150 | D05637 | hsa1129 | D08418 |
| hsa3358 | D00563 | hsa155 | D02150 | hsa151 | D05637 | hsa1131 | D08418 |
| hsa2798 | D00573 | hsa154 | D02151 | hsa152 | D05637 | hsa1132 | D08418 |
| hsa153 | D00597 | hsa1812 | D02162 | hsa146 | D05648 | hsa1133 | D08418 |
| hsa153 | D00598 | hsa1813 | D02162 | hsa147 | D05648 | hsa1128 | D08419 |
| hsa153 | D00599 | hsa1813 | D02163 | hsa148 | D05648 | hsa1129 | D08419 |
| hsa154 | D00599 | hsa1129 | D02200 | hsa150 | D05648 | hsa1131 | D08419 |
| hsa155 | D00599 | hsa1131 | D02200 | hsa151 | D05648 | hsa1132 | D08419 |
| hsa146 | D00600 | hsa4988 | D02205 | hsa152 | D05648 | hsa1133 | D08419 |
| hsa147 | D00600 | hsa1813 | D02208 | hsa153 | D05648 | hsa154 | D08424 |
| hsa148 | D00600 | hsa3351 | D02211 | hsa154 | D05648 | hsa1128 | D08425 |
| hsa153 | D00600 | hsa3352 | D02211 | hsa155 | D05648 | hsa1129 | D08425 |
| hsa154 | D00600 | hsa1128 | D02212 | hsa146 | D05649 | hsa1131 | D08425 |
| hsa153 | D00601 | hsa1129 | D02212 | hsa147 | D05649 | hsa1132 | D08425 |
| hsa153 | D00602 | hsa1131 | D02212 | hsa148 | D05649 | hsa1133 | D08425 |
| hsa154 | D00602 | hsa1132 | D02212 | hsa150 | D05649 | hsa1128 | D08426 |
| hsa155 | D00602 | hsa1133 | D02212 | hsa151 | D05649 | hsa1129 | D08426 |
| hsa153 | D00603 | hsa1813 | D02213 | hsa152 | D05649 | hsa1131 | D08426 |
| hsa154 | D00603 | hsa4986 | D02227 | hsa153 | D05649 | hsa1132 | D08426 |
| hsa150 | D00604 | hsa4988 | D02227 | hsa154 | D05649 | hsa1133 | D08426 |
| hsa151 | D00604 | hsa3269 | D02234 | hsa155 | D05649 | hsa1813 | D08430 |
| hsa152 | D00604 | hsa554 | D02235 | hsa3269 | D05660 | hsa3269 | D08432 |
| hsa150 | D00605 | hsa1812 | D02236 | hsa4986 | D05667 | hsa1128 | D08441 |
| hsa151 | D00605 | hsa1813 | D02236 | hsa4988 | D05667 | hsa1129 | D08441 |
| hsa152 | D00605 | hsa4985 | D02238 | hsa1813 | D05677 | hsa1131 | D08441 |
| hsa150 | D00606 | hsa4986 | D02238 | hsa1813 | D05682 | hsa1132 | D08441 |
| hsa146 | D00607 | hsa4988 | D02238 | hsa1814 | D05682 | hsa1133 | D08441 |
| hsa147 | D00607 | hsa3269 | D02245 | hsa1813 | D05690 | hsa153 | D08443 |
| hsa148 | D00607 | hsa1128 | D02246 | hsa154 | D05691 | hsa154 | D08443 |
| hsa150 | D00607 | hsa1129 | D02246 | hsa3274 | D05699 | hsa155 | D08443 |
| hsa151 | D00607 | hsa1131 | D02246 | hsa135 | D05711 | hsa146 | D08449 |
| hsa152 | D00607 | hsa1132 | D02246 | hsa154 | D05718 | hsa147 | D08449 |
| hsa146 | D00608 | hsa1133 | D02246 | hsa6915 | D05727 | hsa148 | D08449 |
| hsa147 | D00608 | hsa1128 | D02247 | hsa154 | D05730 | hsa150 | D08449 |
| hsa148 | D00608 | hsa1129 | D02247 | hsa1268 | D05731 | hsa151 | D08449 |
| hsa146 | D00609 | hsa1131 | D02247 | hsa5732 | D05732 | hsa152 | D08449 |
| hsa147 | D00609 | hsa1132 | D02247 | hsa2693 | D05734 | hsa153 | D08449 |
| hsa148 | D00609 | hsa1133 | D02247 | hsa3356 | D05738 | hsa154 | D08449 |
| hsa146 | D00610 | hsa1813 | D02248 | hsa3358 | D05738 | hsa155 | D08449 |
| hsa147 | D00610 | hsa3269 | D02249 | hsa3351 | D05740 | hsa1813 | D08456 |
| hsa148 | D00610 | hsa6752 | D02250 | hsa3352 | D05740 | hsa3356 | D08456 |
| hsa1812 | D00613 | hsa146 | D02268 | hsa3269 | D05742 | hsa1813 | D08457 |
| hsa185 | D00626 | hsa147 | D02268 | hsa1813 | D05768 | hsa4988 | D08473 |
| hsa185 | D00627 | hsa148 | D02268 | hsa1128 | D05779 | hsa154 | D08474 |
| hsa153 | D00632 | hsa150 | D02268 | hsa1129 | D05779 | hsa150 | D08482 |
| hsa1812 | D00633 | hsa151 | D02268 | hsa1131 | D05779 | hsa151 | D08482 |
| hsa1813 | D00633 | hsa152 | D02268 | hsa1132 | D05779 | hsa152 | D08482 |
| hsa1814 | D00633 | hsa4988 | D02271 | hsa1133 | D05779 | hsa3351 | D08485 |
| hsa1815 | D00633 | hsa154 | D02281 | hsa154 | D05792 | hsa3352 | D08485 |
| hsa1816 | D00633 | hsa1129 | D02292 | hsa185 | D05801 | hsa150 | D08487 |
| hsa153 | D00634 | hsa134 | D02300 | hsa3350 | D05804 | hsa151 | D08487 |
| hsa153 | D00635 | hsa135 | D02300 | hsa134 | D05818 | hsa152 | D08487 |
| hsa146 | D00636 | hsa136 | D02300 | hsa4157 | D05827 | hsa1813 | D08489 |
| hsa147 | D00636 | hsa140 | D02300 | hsa4158 | D05827 | hsa1814 | D08489 |
| hsa148 | D00636 | hsa3269 | D02327 | hsa4159 | D05827 | hsa1815 | D08489 |
| hsa153 | D00636 | hsa153 | D02338 | hsa4160 | D05827 | hsa3274 | D08494 |
| hsa153 | D00644 | hsa1813 | D02340 | hsa4161 | D05827 | hsa3274 | D08495 |
| hsa1131 | D00646 | hsa3356 | D02340 | hsa154 | D05832 | hsa3269 | D08497 |
| hsa3269 | D00659 | hsa153 | D02342 | hsa1813 | D05832 | hsa5724 | D08497 |
| hsa1131 | D00661 | hsa5737 | D02343 | hsa155 | D05879 | hsa3356 | D08508 |
| hsa3269 | D00662 | hsa146 | D02349 | hsa1815 | D05891 | hsa2692 | D08509 |
| hsa3269 | D00663 | hsa147 | D02349 | hsa154 | D05902 | hsa150 | D08511 |
| hsa3269 | D00664 | hsa148 | D02349 | hsa4986 | D05907 | hsa151 | D08511 |
| hsa3269 | D00665 | hsa150 | D02349 | hsa4988 | D05938 | hsa152 | D08511 |
| hsa3269 | D00666 | hsa151 | D02349 | hsa3274 | D05939 | hsa1909 | D08517 |
| hsa3269 | D00668 | hsa152 | D02349 | hsa3360 | D05941 | hsa1131 | D08522 |
| hsa3269 | D00669 | hsa153 | D02349 | hsa6915 | D05970 | hsa2692 | D08523 |
| hsa3269 | D00671 | hsa154 | D02349 | hsa6870 | D05996 | hsa153 | D08525 |
| hsa3269 | D00672 | hsa155 | D02349 | hsa1128 | D06000 | hsa154 | D08525 |
| hsa3274 | D00673 | hsa1813 | D02354 | hsa4988 | D06007 | hsa1813 | D08549 |
| hsa3351 | D00674 | hsa3350 | D02357 | hsa185 | D06010 | hsa1813 | D08558 |
| hsa3352 | D00674 | hsa3356 | D02357 | hsa3269 | D06012 | hsa146 | D08560 |
| hsa3351 | D00675 | hsa3357 | D02357 | hsa153 | D06014 | hsa147 | D08560 |
| hsa3352 | D00675 | hsa3358 | D02357 | hsa154 | D06014 | hsa148 | D08560 |
| hsa3351 | D00676 | hsa3363 | D02357 | hsa155 | D06014 | hsa3350 | D08561 |
| hsa3352 | D00676 | hsa153 | D02358 | hsa1128 | D06015 | hsa146 | D08569 |
| hsa146 | D00679 | hsa154 | D02359 | hsa1129 | D06015 | hsa147 | D08569 |
| hsa147 | D00679 | hsa3356 | D02363 | hsa1131 | D06015 | hsa148 | D08569 |
| hsa148 | D00679 | hsa2798 | D02369 | hsa1132 | D06015 | hsa154 | D08570 |
| hsa3350 | D00679 | hsa1813 | D02371 | hsa1133 | D06015 | hsa552 | D08571 |
| hsa146 | D00680 | hsa1813 | D02372 | hsa134 | D06019 | hsa553 | D08571 |
| hsa147 | D00680 | hsa3356 | D02372 | hsa846 | D06020 | hsa554 | D08571 |
| hsa148 | D00680 | hsa153 | D02374 | hsa3269 | D06021 | hsa153 | D08572 |
| hsa150 | D00680 | hsa154 | D02374 | hsa9340 | D06053 | hsa154 | D08572 |
| hsa151 | D00680 | hsa155 | D02374 | hsa3360 | D06056 | hsa155 | D08572 |
| hsa152 | D00680 | hsa80834 | D02381 | hsa1128 | D06061 | hsa146 | D08578 |
| hsa3350 | D00681 | hsa83756 | D02381 | hsa1129 | D06061 | hsa147 | D08578 |
| hsa3356 | D00681 | hsa10800 | D02384 | hsa1131 | D06061 | hsa148 | D08578 |
| hsa3357 | D00681 | hsa150 | D02388 | hsa1132 | D06061 | hsa1813 | D08585 |
| hsa3358 | D00681 | hsa151 | D02388 | hsa1133 | D06061 | hsa3269 | D08587 |
| hsa3363 | D00681 | hsa152 | D02388 | hsa3269 | D06063 | hsa1813 | D08590 |
| hsa5737 | D00682 | hsa153 | D02389 | hsa5745 | D06078 | hsa5737 | D08591 |
| hsa154 | D00683 | hsa154 | D02389 | hsa134 | D06103 | hsa64805 | D08594 |
| hsa154 | D00684 | hsa155 | D02389 | hsa136 | D06103 | hsa1128 | D08595 |
| hsa154 | D00685 | hsa154 | D02396 | hsa134 | D06104 | hsa1129 | D08595 |
| hsa154 | D00686 | hsa154 | D02404 | hsa136 | D06104 | hsa1131 | D08595 |
| hsa154 | D00687 | hsa150 | D02407 | hsa7201 | D06120 | hsa1132 | D08595 |
| hsa154 | D00688 | hsa151 | D02407 | hsa1241 | D06140 | hsa1133 | D08595 |
| hsa134 | D00691 | hsa152 | D02407 | hsa56413 | D06140 | hsa4988 | D08597 |
| hsa136 | D00691 | hsa1128 | D02417 | hsa4988 | D06147 | hsa153 | D08598 |
| hsa1813 | D00702 | hsa1129 | D02417 | hsa3274 | D06157 | hsa154 | D08598 |
| hsa3350 | D00702 | hsa1131 | D02417 | hsa153 | D06162 | hsa155 | D08598 |
| hsa1128 | D00715 | hsa1132 | D02417 | hsa154 | D06162 | hsa153 | D08600 |
| hsa1129 | D00715 | hsa1133 | D02417 | hsa155 | D06162 | hsa154 | D08600 |
| hsa1131 | D00715 | hsa3269 | D02419 | hsa1128 | D06175 | hsa150 | D08611 |
| hsa1132 | D00715 | hsa1128 | D02535 | hsa1129 | D06175 | hsa151 | D08611 |
| hsa1133 | D00715 | hsa1129 | D02535 | hsa1131 | D06175 | hsa152 | D08611 |
| hsa1128 | D00716 | hsa1131 | D02535 | hsa1132 | D06175 | hsa146 | D08614 |
| hsa1129 | D00716 | hsa1132 | D02535 | hsa1133 | D06175 | hsa147 | D08614 |
| hsa1131 | D00716 | hsa1133 | D02535 | hsa153 | D06176 | hsa148 | D08614 |
| hsa1132 | D00716 | hsa146 | D02537 | hsa154 | D06176 | hsa150 | D08614 |
| hsa1133 | D00716 | hsa147 | D02537 | hsa155 | D06176 | hsa151 | D08614 |
| hsa1128 | D00717 | hsa148 | D02537 | hsa1128 | D06192 | hsa152 | D08614 |
| hsa1129 | D00717 | hsa153 | D02537 | hsa1129 | D06192 | hsa4988 | D08623 |
| hsa1131 | D00717 | hsa146 | D02568 | hsa1131 | D06192 | hsa146 | D08624 |
| hsa1128 | D00719 | hsa147 | D02568 | hsa1132 | D06192 | hsa147 | D08624 |
| hsa1129 | D00719 | hsa148 | D02568 | hsa1133 | D06192 | hsa148 | D08624 |
| hsa1131 | D00719 | hsa3350 | D02568 | hsa4988 | D06208 | hsa150 | D08624 |
| hsa1132 | D00719 | hsa150 | D02569 | hsa5739 | D06213 | hsa151 | D08624 |
| hsa1133 | D00719 | hsa151 | D02569 | hsa1128 | D06217 | hsa152 | D08624 |
| hsa1128 | D00720 | hsa152 | D02569 | hsa1129 | D06217 | hsa3356 | D08626 |
| hsa1129 | D00720 | hsa3350 | D02569 | hsa1131 | D06217 | hsa3357 | D08626 |
| hsa1131 | D00720 | hsa3350 | D02576 | hsa1132 | D06217 | hsa3358 | D08626 |
| hsa1132 | D00720 | hsa3350 | D02577 | hsa1133 | D06217 | hsa5739 | D08628 |
| hsa1133 | D00720 | hsa3356 | D02577 | hsa146 | D06234 | hsa154 | D08629 |
| hsa1128 | D00721 | hsa3357 | D02577 | hsa147 | D06234 | hsa2798 | D08635 |
| hsa1129 | D00721 | hsa3358 | D02577 | hsa148 | D06234 | hsa1813 | D08636 |
| hsa1131 | D00721 | hsa3358 | D02578 | hsa3269 | D06246 | hsa1813 | D08637 |
| hsa1132 | D00721 | hsa4543 | D02578 | hsa2798 | D06247 | hsa1128 | D08638 |
| hsa1133 | D00721 | hsa4544 | D02578 | hsa2798 | D06248 | hsa1129 | D08638 |
| hsa3274 | D00721 | hsa7201 | D02588 | hsa3356 | D06253 | hsa1131 | D08638 |
| hsa1128 | D00722 | hsa5724 | D02590 | hsa3357 | D06253 | hsa1132 | D08638 |
| hsa1129 | D00722 | hsa3269 | D02599 | hsa3358 | D06253 | hsa1133 | D08638 |
| hsa1131 | D00722 | hsa1128 | D02603 | hsa4988 | D06268 | hsa4986 | D08639 |
| hsa1132 | D00722 | hsa1129 | D02603 | hsa2492 | D06269 | hsa4988 | D08639 |
| hsa1133 | D00722 | hsa1131 | D02603 | hsa1128 | D06273 | hsa3269 | D08645 |
| hsa1128 | D00723 | hsa1132 | D02603 | hsa1131 | D06273 | hsa134 | D08646 |
| hsa1129 | D00723 | hsa1133 | D02603 | hsa5737 | D06274 | hsa135 | D08646 |
| hsa1131 | D00723 | hsa1812 | D02604 | hsa6915 | D06280 | hsa136 | D08646 |
| hsa1813 | D00726 | hsa1813 | D02604 | hsa6751 | D06281 | hsa140 | D08646 |
| hsa4985 | D00729 | hsa1813 | D02605 | hsa6752 | D06281 | hsa134 | D08647 |
| hsa4986 | D00729 | hsa1813 | D02608 | hsa6753 | D06281 | hsa135 | D08647 |
| hsa4988 | D00729 | hsa1813 | D02609 | hsa6754 | D06281 | hsa136 | D08647 |
| hsa146 | D00743 | hsa1813 | D02610 | hsa6755 | D06281 | hsa140 | D08647 |
| hsa147 | D00743 | hsa1812 | D02612 | hsa6869 | D06293 | hsa3269 | D08648 |
| hsa148 | D00743 | hsa1813 | D02612 | hsa1234 | D06297 | hsa2798 | D08649 |
| hsa146 | D00756 | hsa1812 | D02613 | hsa6869 | D06317 | hsa1128 | D08653 |
| hsa147 | D00756 | hsa1813 | D02613 | hsa3350 | D06327 | hsa1129 | D08653 |
| hsa148 | D00756 | hsa153 | D02614 | hsa153 | D06328 | hsa1131 | D08653 |
| hsa146 | D00757 | hsa1813 | D02622 | hsa153 | D06329 | hsa1132 | D08653 |
| hsa147 | D00757 | hsa1813 | D02623 | hsa1128 | D06330 | hsa1133 | D08653 |
| hsa148 | D00757 | hsa1813 | D02625 | hsa1132 | D06330 | hsa5737 | D08661 |
| hsa150 | D00757 | hsa1813 | D02626 | hsa1128 | D06331 | hsa146 | D08662 |
| hsa151 | D00757 | hsa1813 | D02627 | hsa1132 | D06331 | hsa147 | D08662 |
| hsa152 | D00757 | hsa1813 | D02634 | hsa3356 | D06343 | hsa148 | D08662 |
| hsa1128 | D00765 | hsa1812 | D02641 | hsa3357 | D06343 | hsa150 | D08683 |
| hsa1129 | D00765 | hsa1813 | D02641 | hsa3358 | D06343 | hsa151 | D08683 |
| hsa1131 | D00765 | hsa1812 | D02644 | hsa150 | D06344 | hsa152 | D08683 |
| hsa1132 | D00765 | hsa1813 | D02644 | hsa151 | D06344 | hsa146 | D08684 |
| hsa1133 | D00765 | hsa1813 | D02645 | hsa152 | D06344 | hsa147 | D08684 |
| hsa64805 | D00769 | hsa1815 | D02656 | hsa3360 | D06353 | hsa148 | D08684 |
| hsa3269 | D00774 | hsa3356 | D02656 | hsa3274 | D06355 | hsa150 | D08684 |
| hsa150 | D00776 | hsa1813 | D02666 | hsa154 | D06374 | hsa151 | D08684 |
| hsa151 | D00776 | hsa3356 | D02666 | hsa146 | D06380 | hsa152 | D08684 |
| hsa152 | D00776 | hsa3357 | D02666 | hsa148 | D06380 | hsa150 | D08685 |
| hsa1128 | D00778 | hsa3358 | D02666 | hsa148 | D06394 | hsa151 | D08685 |
| hsa1129 | D00778 | hsa1813 | D02670 | hsa4986 | D06395 | hsa152 | D08685 |
| hsa1131 | D00778 | hsa1813 | D02671 | hsa3360 | D06396 | hsa1813 | D08687 |
| hsa1132 | D00778 | hsa3356 | D02671 | hsa2492 | D06400 | hsa3356 | D08687 |
| hsa1133 | D00778 | hsa1813 | D02675 | hsa2740 | D06404 | hsa1812 | D08691 |
| hsa1128 | D00779 | hsa3356 | D02675 | hsa4986 | D06405 | hsa1813 | D08691 |
| hsa1129 | D00779 | hsa3357 | D02675 | hsa2492 | D06457 | hsa1812 | D08692 |
| hsa1131 | D00779 | hsa3358 | D02675 | hsa3973 | D06457 | hsa1813 | D08692 |
| hsa1132 | D00779 | hsa1812 | D02676 | hsa2492 | D06459 | hsa1812 | D08693 |
| hsa1133 | D00779 | hsa1813 | D02676 | hsa3973 | D06459 | hsa1813 | D08693 |
| hsa1813 | D00780 | hsa1814 | D02676 | hsa6752 | D06495 | hsa3269 | D08768 |
| hsa1128 | D00782 | hsa1815 | D02676 | hsa4158 | D06544 | hsa80834 | D08836 |
| hsa1129 | D00782 | hsa1816 | D02676 | hsa5745 | D06546 | hsa83756 | D08836 |
| hsa1131 | D00782 | hsa1812 | D02680 | hsa3269 | D06552 | hsa1128 | D08837 |
| hsa1132 | D00782 | hsa1813 | D02680 | hsa1234 | D06557 | hsa1129 | D08837 |
| hsa1133 | D00782 | hsa3350 | D02680 | hsa1813 | D06566 | hsa1131 | D08837 |
| hsa1813 | D00784 | hsa3356 | D02680 | hsa3350 | D06566 | hsa1132 | D08837 |
| hsa1814 | D00784 | hsa1813 | D02681 | hsa1813 | D06567 | hsa1133 | D08837 |
| hsa1815 | D00784 | hsa1813 | D02682 | hsa3350 | D06567 | hsa2740 | D08843 |
| hsa1128 | D00787 | hsa1813 | D02683 | hsa4157 | D06569 | hsa155 | D08850 |
| hsa1129 | D00787 | hsa3356 | D02686 | hsa4158 | D06569 | hsa155 | D08851 |
| hsa1131 | D00787 | hsa3357 | D02686 | hsa4159 | D06569 | hsa2693 | D08856 |
| hsa1132 | D00787 | hsa3358 | D02686 | hsa4160 | D06569 | hsa2550 | D08861 |
| hsa1133 | D00787 | hsa1813 | D02688 | hsa4161 | D06569 | hsa9568 | D08861 |
| hsa1812 | D00788 | hsa3350 | D02688 | hsa6869 | D06574 | hsa80834 | D08862 |
| hsa1813 | D00788 | hsa3356 | D02688 | hsa6869 | D06597 | hsa83756 | D08862 |
| hsa1128 | D00789 | hsa3357 | D02688 | hsa3358 | D06613 | hsa185 | D08864 |
| hsa1129 | D00789 | hsa3358 | D02688 | hsa4988 | D06618 | hsa185 | D08865 |
| hsa1131 | D00789 | hsa3362 | D02688 | hsa153 | D06622 | hsa2492 | D08895 |
| hsa1132 | D00789 | hsa4543 | D02689 | hsa146 | D06623 | hsa2798 | D08901 |
| hsa1133 | D00789 | hsa4544 | D02689 | hsa147 | D06623 | hsa2912 | D08908 |
| hsa150 | D00789 | hsa2492 | D02692 | hsa148 | D06623 | hsa1129 | D08923 |
| hsa151 | D00789 | hsa3973 | D02692 | hsa1813 | D06623 | hsa1131 | D08923 |
| hsa152 | D00789 | hsa886 | D02693 | hsa1814 | D06623 | hsa624 | D08937 |
| hsa1813 | D00789 | hsa155 | D02702 | hsa1815 | D06623 | hsa5729 | D08940 |
| hsa3269 | D00789 | hsa5731 | D02705 | hsa3269 | D06623 | hsa154 | D08945 |
| hsa3356 | D00789 | hsa886 | D02710 | hsa3356 | D06623 | hsa146 | D08966 |
| hsa3357 | D00789 | hsa5737 | D02719 | hsa3357 | D06623 | hsa147 | D08966 |
| hsa3358 | D00789 | hsa5739 | D02720 | hsa3358 | D06623 | hsa148 | D08966 |
| hsa1812 | D00790 | hsa5739 | D02721 | hsa3362 | D06623 | hsa3356 | D08969 |
| hsa1813 | D00790 | hsa5731 | D02722 | hsa3356 | D06632 | hsa7852 | D08971 |
| hsa1813 | D00791 | hsa5731 | D02723 | hsa3356 | D06633 | hsa146 | D08982 |
| hsa1813 | D00792 | hsa5737 | D02724 | hsa153 | D06646 | hsa147 | D08982 |
| hsa1813 | D00793 | hsa5732 | D02725 | hsa2692 | D06655 | hsa148 | D08982 |
| hsa1813 | D00794 | hsa5732 | D02726 | hsa10800 | D06659 | hsa150 | D08982 |
| hsa3356 | D00794 | hsa5737 | D02727 | hsa3357 | D06660 | hsa151 | D08982 |
| hsa1813 | D00795 | hsa1813 | D02729 | hsa3358 | D06660 | hsa152 | D08982 |
| hsa3356 | D00795 | hsa3360 | D02730 | hsa1234 | D06670 | hsa153 | D08982 |
| hsa1813 | D00796 | hsa10800 | D02732 | hsa150 | D06671 | hsa154 | D08982 |
| hsa1813 | D00797 | hsa2798 | D02738 | hsa151 | D06671 | hsa155 | D08982 |
| hsa1813 | D00798 | hsa10800 | D02739 | hsa152 | D06671 | hsa64805 | D08983 |
| hsa1813 | D00799 | hsa10800 | D02741 | hsa552 | D06672 | hsa3577 | D08984 |
| hsa1813 | D00800 | hsa1128 | D02750 | hsa553 | D06672 | hsa3579 | D08984 |
| hsa3269 | D00814 | hsa3269 | D02760 | hsa554 | D06672 | hsa5021 | D08986 |
| hsa146 | D00819 | hsa153 | D02765 | hsa1128 | D06877 | hsa6869 | D08988 |
| hsa147 | D00819 | hsa154 | D02765 | hsa1131 | D06878 | hsa134 | D08989 |
| hsa148 | D00819 | hsa155 | D02765 | hsa1812 | D07065 | hsa846 | D08991 |
| hsa3356 | D00819 | hsa3350 | D02767 | hsa1813 | D07065 | hsa135 | D09003 |
| hsa3358 | D00819 | hsa3356 | D02767 | hsa1128 | D07070 | hsa2912 | D09008 |
| hsa3356 | D00820 | hsa3357 | D02767 | hsa1129 | D07070 | hsa2913 | D09008 |
| hsa3357 | D00820 | hsa3358 | D02767 | hsa1131 | D07070 | hsa1268 | D09009 |
| hsa3358 | D00820 | hsa134 | D02769 | hsa1132 | D07070 | hsa2692 | D09015 |
| hsa4988 | D00835 | hsa135 | D02769 | hsa1133 | D07070 | hsa64805 | D09017 |
| hsa4986 | D00836 | hsa136 | D02769 | hsa3274 | D07072 | hsa3360 | D09205 |
| hsa4988 | D00836 | hsa140 | D02769 | hsa1909 | D07077 | hsa154 | D09318 |
| hsa4986 | D00837 | hsa146 | D02774 | hsa1131 | D07078 | hsa154 | D09319 |
| hsa4988 | D00837 | hsa147 | D02774 | hsa1128 | D07079 | hsa2798 | D09335 |
| hsa4986 | D00838 | hsa148 | D02774 | hsa1129 | D07079 | hsa2798 | D09336 |
| hsa4988 | D00838 | hsa1812 | D02775 | hsa1131 | D07079 | hsa155 | D09344 |
| hsa4988 | D00839 | hsa2862 | D02801 | hsa1132 | D07079 | hsa1268 | D09349 |
| hsa4988 | D00840 | hsa3351 | D02824 | hsa1133 | D07079 | hsa3350 | D09358 |
| hsa4988 | D00841 | hsa3352 | D02824 | hsa1128 | D07081 | hsa3350 | D09359 |
| hsa4988 | D00842 | hsa3351 | D02825 | hsa1129 | D07081 | hsa1268 | D09362 |
| hsa4985 | D00843 | hsa3352 | D02825 | hsa1131 | D07081 | hsa1268 | D09363 |
| hsa4986 | D00843 | hsa3351 | D02826 | hsa1132 | D07081 | hsa1813 | D09366 |
| hsa4988 | D00843 | hsa3352 | D02826 | hsa1133 | D07081 | hsa1814 | D09366 |
| hsa4988 | D00844 | hsa153 | D02834 | hsa1128 | D07083 | hsa3350 | D09366 |
| hsa4988 | D00845 | hsa154 | D02834 | hsa1129 | D07083 | hsa6869 | D09378 |
| hsa4985 | D00847 | hsa155 | D02834 | hsa1131 | D07083 | hsa4543 | D09388 |
| hsa4986 | D00847 | hsa3356 | D02836 | hsa1132 | D07083 | hsa4544 | D09388 |
| hsa4988 | D00847 | hsa10800 | D02846 | hsa1133 | D07083 | hsa10203 | D09391 |
| hsa1813 | D00987 | hsa10800 | D02848 | hsa1128 | D07084 | hsa10203 | D09392 |
| hsa2798 | D00988 | hsa10800 | D02849 | hsa1129 | D07084 | hsa1813 | D09397 |
| hsa2798 | D00989 | hsa10800 | D02850 | hsa1131 | D07084 | hsa1814 | D09397 |
| hsa2798 | D00990 | hsa10800 | D02851 | hsa1132 | D07084 | hsa3350 | D09397 |
| hsa146 | D00996 | hsa5731 | D02860 | hsa1133 | D07084 | hsa2798 | D09400 |
| hsa147 | D00996 | hsa1128 | D02876 | hsa148 | D07097 | hsa1128 | D09402 |
| hsa148 | D00996 | hsa1129 | D02876 | hsa1128 | D07099 | hsa1129 | D09402 |
| hsa150 | D00996 | hsa1131 | D02876 | hsa1129 | D07099 | hsa1131 | D09402 |
| hsa151 | D00996 | hsa4988 | D02878 | hsa1131 | D07099 | hsa1132 | D09402 |
| hsa152 | D00996 | hsa3356 | D02893 | hsa1132 | D07099 | hsa1133 | D09402 |
| hsa153 | D00996 | hsa3358 | D02893 | hsa1133 | D07099 | hsa155 | D09535 |
| hsa154 | D00996 | hsa146 | D02901 | hsa1813 | D07101 | hsa3269 | D09570 |
| hsa155 | D00996 | hsa147 | D02901 | hsa1813 | D07102 | hsa10203 | D09600 |
| hsa146 | D00997 | hsa148 | D02901 | hsa4985 | D07113 | hsa799 | D09600 |
| hsa147 | D00997 | hsa146 | D02910 | hsa4986 | D07113 | hsa10203 | D09601 |
| hsa148 | D00997 | hsa147 | D02910 | hsa4988 | D07113 | hsa799 | D09601 |
| hsa150 | D00997 | hsa148 | D02910 | hsa4988 | D07122 | hsa64805 | D09607 |
| hsa151 | D00997 | hsa153 | D02910 | hsa148 | D07124 | hsa64805 | D09608 |
| hsa152 | D00997 | hsa185 | D02939 | hsa3269 | D07125 | hsa1394 | D09610 |
| hsa1128 | D00999 | hsa186 | D02939 | hsa4986 | D07132 | hsa5732 | D09613 |
| hsa1129 | D00999 | hsa4988 | D02941 | hsa4988 | D07132 | hsa5732 | D09614 |
| hsa1131 | D00999 | hsa4988 | D02942 | hsa6915 | D07140 | hsa3356 | D09645 |
| hsa1132 | D00999 | hsa3269 | D02950 | hsa153 | D07156 | hsa6869 | D09650 |
| hsa1133 | D00999 | hsa134 | D02964 | hsa154 | D07156 | hsa6869 | D09651 |
| hsa1128 | D01000 | hsa6869 | D02968 | hsa155 | D07156 | hsa134 | D09684 |
| hsa1129 | D01000 | hsa150 | D02972 | hsa1909 | D07171 | hsa3360 | D09693 |
| hsa1131 | D01000 | hsa151 | D02972 | hsa153 | D07181 | hsa3360 | D09694 |
| hsa1132 | D01000 | hsa152 | D02972 | hsa154 | D07181 | hsa1394 | D09695 |
| hsa1133 | D01000 | hsa5732 | D02975 | hsa155 | D07181 | hsa154 | D09696 |
| hsa1128 | D01002 | hsa153 | D02976 | hsa153 | D07182 | hsa154 | D09697 |
| hsa1129 | D01002 | hsa154 | D02981 | hsa154 | D07182 | hsa3350 | D09698 |
| hsa1131 | D01002 | hsa552 | D02983 | hsa155 | D07182 | hsa3350 | D09699 |
| hsa1132 | D01002 | hsa553 | D02983 | hsa153 | D07183 | hsa3269 | D09705 |
| hsa1133 | D01002 | hsa554 | D02983 | hsa154 | D07183 | hsa135 | D09717 |
| hsa1131 | D01003 | hsa1813 | D02995 | hsa155 | D07183 | hsa2740 | D09723 |
| hsa1128 | D01004 | hsa3350 | D02995 | hsa153 | D07184 | hsa2740 | D09729 |
| hsa1129 | D01004 | hsa3356 | D02995 | hsa154 | D07184 | hsa155 | D09732 |
| hsa1131 | D01004 | hsa3358 | D02995 | hsa155 | D07184 | hsa1128 | D09752 |
| hsa1132 | D01004 | hsa3362 | D02995 | hsa3269 | D07194 | hsa1129 | D09752 |
| hsa1133 | D01004 | hsa3363 | D02995 | hsa3269 | D07195 | hsa1131 | D09752 |
| hsa1131 | D01005 | hsa150 | D03002 | hsa3269 | D07196 | hsa1132 | D09752 |
| hsa1128 | D01006 | hsa151 | D03002 | hsa3269 | D07197 | hsa1133 | D09752 |
| hsa1129 | D01006 | hsa152 | D03002 | hsa3269 | D07198 | hsa1233 | D09761 |
| hsa1131 | D01006 | hsa3356 | D03005 | hsa1813 | D07217 | hsa2149 | D09765 |
| hsa1128 | D01007 | hsa5021 | D03008 | hsa1129 | D07226 | hsa2149 | D09766 |
| hsa1129 | D01007 | hsa1909 | D03009 | hsa1131 | D07226 | hsa799 | D09771 |
| hsa1131 | D01007 | hsa1128 | D03011 | hsa552 | D07227 | hsa6915 | D09774 |
| hsa1132 | D01007 | hsa1129 | D03011 | hsa553 | D07227 | hsa148 | D09784 |
| hsa1133 | D01007 | hsa1131 | D03011 | hsa554 | D07227 | hsa2550 | D09791 |
| hsa150 | D01008 | hsa1132 | D03011 | hsa5021 | D07228 | hsa9568 | D09791 |
| hsa151 | D01008 | hsa1133 | D03011 | hsa5021 | D07229 | hsa146 | D09794 |
| hsa152 | D01008 | hsa3351 | D03014 | hsa2798 | D07259 | hsa147 | D09794 |
| hsa146 | D01017 | hsa3352 | D03014 | hsa4988 | D07285 | hsa148 | D09794 |
| hsa147 | D01017 | hsa134 | D03051 | hsa4988 | D07286 | hsa150 | D09794 |
| hsa148 | D01017 | hsa1128 | D03076 | hsa1128 | D07301 | hsa151 | D09794 |
| hsa150 | D01017 | hsa1129 | D03076 | hsa1129 | D07301 | hsa152 | D09794 |
| hsa151 | D01017 | hsa1131 | D03076 | hsa1131 | D07301 | hsa153 | D09794 |
| hsa152 | D01017 | hsa1132 | D03076 | hsa1132 | D07301 | hsa154 | D09794 |
| hsa153 | D01017 | hsa1133 | D03076 | hsa1133 | D07301 | hsa155 | D09794 |
| hsa154 | D01017 | hsa1128 | D03087 | hsa1128 | D07303 | hsa5021 | D09797 |
| hsa155 | D01017 | hsa1129 | D03087 | hsa1129 | D07303 | hsa4157 | D09801 |
| hsa146 | D01019 | hsa1131 | D03087 | hsa1131 | D07303 | hsa4158 | D09801 |
| hsa147 | D01019 | hsa1132 | D03087 | hsa1132 | D07303 | hsa4159 | D09801 |
| hsa148 | D01019 | hsa1133 | D03087 | hsa1133 | D07303 | hsa4160 | D09801 |
| hsa150 | D01019 | hsa1128 | D03088 | hsa1813 | D07305 | hsa4161 | D09801 |
| hsa151 | D01019 | hsa1129 | D03088 | hsa1814 | D07305 | hsa3269 | D09808 |
| hsa152 | D01019 | hsa1131 | D03088 | hsa3356 | D07307 | hsa1128 | D09819 |
| hsa146 | D01020 | hsa1132 | D03088 | hsa3358 | D07307 | hsa1129 | D09819 |
| hsa147 | D01020 | hsa1133 | D03088 | hsa1813 | D07309 | hsa1131 | D09819 |
| hsa148 | D01020 | hsa11255 | D03102 | hsa1813 | D07310 | hsa1132 | D09819 |
| hsa146 | D01021 | hsa3269 | D03102 | hsa1814 | D07310 | hsa1133 | D09819 |
| hsa147 | D01021 | hsa135 | D03120 | hsa1813 | D07312 | hsa150 | D09819 |
| hsa148 | D01021 | hsa3350 | D03121 | hsa1814 | D07316 | hsa151 | D09819 |
| hsa148 | D01022 | hsa4988 | D03156 | hsa3358 | D07316 | hsa152 | D09819 |
| hsa146 | D01023 | hsa1813 | D03165 | hsa1128 | D07349 | hsa1813 | D09819 |
| hsa147 | D01023 | hsa3269 | D03166 | hsa1129 | D07349 | hsa3269 | D09819 |
| hsa148 | D01023 | hsa154 | D03170 | hsa1131 | D07349 | hsa3356 | D09819 |
| hsa146 | D01024 | hsa153 | D03177 | hsa1132 | D07349 | hsa3357 | D09819 |
| hsa147 | D01024 | hsa154 | D03177 | hsa1133 | D07349 | hsa3358 | D09819 |
| hsa148 | D01024 | hsa155 | D03177 | hsa154 | D07377 | hsa552 | D09820 |
| hsa146 | D01025 | hsa5732 | D03187 | hsa4988 | D07385 | hsa553 | D09820 |
| hsa147 | D01025 | hsa153 | D03195 | hsa3269 | D07398 | hsa554 | D09820 |
| hsa148 | D01025 | hsa4986 | D03197 | hsa3269 | D07401 | hsa6915 | D09824 |
| hsa153 | D01025 | hsa4988 | D03197 | hsa3269 | D07402 | hsa146 | D09842 |
| hsa154 | D01025 | hsa154 | D03198 | hsa3269 | D07403 | hsa147 | D09842 |
| hsa155 | D01025 | hsa1234 | D03210 | hsa3269 | D07404 | hsa148 | D09842 |
| hsa153 | D01026 | hsa135 | D03212 | hsa3269 | D07406 | hsa150 | D09842 |
| hsa154 | D01026 | hsa1813 | D03214 | hsa3269 | D07407 | hsa151 | D09842 |
| hsa64805 | D01028 | hsa1814 | D03214 | hsa5724 | D07407 | hsa152 | D09842 |
| hsa1812 | D01044 | hsa2862 | D03216 | hsa4157 | D07417 | hsa153 | D09842 |
| hsa1813 | D01044 | hsa6869 | D03221 | hsa4158 | D07417 | hsa154 | D09842 |
| hsa1813 | D01051 | hsa1128 | D03264 | hsa4159 | D07417 | hsa155 | D09842 |
| hsa1128 | D01077 | hsa1129 | D03264 | hsa4160 | D07417 | hsa153 | D09843 |
| hsa1129 | D01077 | hsa1131 | D03264 | hsa4161 | D07417 | hsa154 | D09843 |
| hsa1131 | D01077 | hsa1132 | D03264 | hsa6751 | D07431 | hsa155 | D09843 |
| hsa1132 | D01077 | hsa1133 | D03264 | hsa6752 | D07431 | hsa7201 | D09845 |
| hsa1133 | D01077 | hsa2798 | D03267 | hsa6753 | D07431 | hsa2149 | D09866 |
| hsa3269 | D01096 | hsa5732 | D03269 | hsa6754 | D07431 | hsa2149 | D09867 |
| hsa1813 | D01101 | hsa1128 | D03274 | hsa6755 | D07431 | hsa11255 | D09870 |
| hsa1129 | D01103 | hsa1129 | D03274 | hsa146 | D07451 | hsa11255 | D09871 |
| hsa1131 | D01103 | hsa1131 | D03274 | hsa147 | D07451 | hsa1813 | D09876 |
| hsa1813 | D01105 | hsa1132 | D03274 | hsa148 | D07451 | hsa3350 | D09876 |
| hsa886 | D01113 | hsa1133 | D03274 | hsa153 | D07451 | hsa3356 | D09876 |
| hsa3269 | D01117 | hsa150 | D03274 | hsa154 | D07451 | hsa1234 | D09878 |
| hsa1128 | D01118 | hsa151 | D03274 | hsa3269 | D07458 | hsa729230 | D09878 |
| hsa1129 | D01118 | hsa152 | D03274 | hsa3269 | D07459 | hsa1234 | D09879 |
| hsa1131 | D01118 | hsa1813 | D03274 | hsa1812 | D07460 | hsa729230 | D09879 |
| hsa1132 | D01118 | hsa3269 | D03274 | hsa1813 | D07460 | hsa1812 | D09886 |
| hsa1133 | D01118 | hsa3356 | D03274 | hsa150 | D07461 | hsa1813 | D09886 |
| hsa6915 | D01123 | hsa3357 | D03274 | hsa151 | D07461 | hsa1814 | D09886 |
| hsa5729 | D01128 | hsa3358 | D03274 | hsa152 | D07461 | hsa1815 | D09886 |
| hsa6915 | D01128 | hsa1128 | D03276 | hsa154 | D07463 | hsa1816 | D09886 |
| hsa1813 | D01130 | hsa1129 | D03276 | hsa146 | D07465 | hsa1812 | D09887 |
| hsa3274 | D01131 | hsa1131 | D03276 | hsa147 | D07465 | hsa1813 | D09887 |
| hsa3269 | D01143 | hsa1132 | D03276 | hsa148 | D07465 | hsa1814 | D09887 |
| hsa1131 | D01148 | hsa1133 | D03276 | hsa153 | D07465 | hsa1815 | D09887 |
| hsa146 | D01163 | hsa3269 | D03285 | hsa154 | D07465 | hsa1816 | D09887 |
| hsa147 | D01163 | hsa799 | D03287 | hsa5021 | D07475 | hsa2740 | D09889 |
| hsa148 | D01163 | hsa3269 | D03290 | hsa1128 | D07477 | hsa3360 | D09933 |
| hsa150 | D01163 | hsa2692 | D03326 | hsa1129 | D07477 | hsa3360 | D09934 |
| hsa151 | D01163 | hsa5745 | D03358 | hsa1131 | D07477 | hsa4157 | D09937 |
| hsa152 | D01163 | hsa64805 | D03359 | hsa1132 | D07477 | hsa4158 | D09937 |
| hsa1813 | D01164 | hsa3269 | D03360 | hsa1133 | D07477 | hsa4159 | D09937 |
| hsa1814 | D01164 | hsa64805 | D03361 | hsa3269 | D07482 | hsa4160 | D09937 |
| hsa3350 | D01164 | hsa2693 | D03373 | hsa3269 | D07483 | hsa4161 | D09937 |
| hsa3356 | D01164 | hsa5737 | D03399 | hsa154 | D07489 | hsa2912 | D09949 |
| hsa1128 | D01165 | hsa154 | D03402 | hsa134 | D07491 | hsa2913 | D09949 |
| hsa1129 | D01165 | hsa4988 | D03405 | hsa3269 | D07492 | hsa1813 | D09953 |
| hsa1131 | D01165 | hsa146 | D03415 | hsa3269 | D07493 | hsa1813 | D09954 |
| hsa1132 | D01165 | hsa147 | D03415 | hsa153 | D07496 | hsa3061 | D09964 |
| hsa1133 | D01165 | hsa148 | D03415 | hsa154 | D07496 | hsa3062 | D09964 |
| hsa3269 | D01172 | hsa153 | D03415 | hsa155 | D07496 | hsa5732 | D09968 |
| hsa3269 | D01174 | hsa154 | D03415 | hsa1128 | D07511 | hsa5732 | D09969 |
| hsa1128 | D01175 | hsa886 | D03442 | hsa1129 | D07511 | hsa3356 | D09976 |
| hsa1129 | D01175 | hsa886 | D03443 | hsa1131 | D07511 | hsa3356 | D09977 |
| hsa1131 | D01175 | hsa153 | D03449 | hsa1132 | D07511 | hsa2693 | D09981 |
| hsa1132 | D01175 | hsa3269 | D03475 | hsa1133 | D07511 | hsa2693 | D09982 |
| hsa1133 | D01175 | hsa3973 | D03478 | hsa1128 | D07513 | hsa6751 | D09988 |
| hsa1813 | D01176 | hsa153 | D03490 | hsa1129 | D07513 | hsa6752 | D09988 |
| hsa1814 | D01176 | hsa3269 | D03491 | hsa1131 | D07513 | hsa6753 | D09988 |
| hsa3356 | D01176 | hsa1812 | D03494 | hsa1132 | D07513 | hsa6754 | D09988 |
| hsa4988 | D01177 | hsa1813 | D03494 | hsa1133 | D07513 | hsa6755 | D09988 |
| hsa153 | D01182 | hsa153 | D03502 | hsa11255 | D07522 | hsa5729 | D09990 |
| hsa154 | D01182 | hsa154 | D03502 | hsa3269 | D07522 | hsa135 | D09991 |
| hsa155 | D01182 | hsa155 | D03502 | hsa153 | D07526 | hsa6608 | D09992 |
| hsa3274 | D01187 | hsa3274 | D03503 | hsa154 | D07534 | hsa5739 | D09994 |
| hsa5739 | D01188 | hsa846 | D03504 | hsa153 | D07537 | hsa1813 | D09997 |
| hsa3269 | D01192 | hsa846 | D03505 | hsa154 | D07537 | hsa3350 | D09997 |
| hsa3274 | D01193 | hsa3356 | D03506 | hsa155 | D07537 | hsa3356 | D09997 |
| hsa1128 | D01201 | hsa3358 | D03506 | hsa1909 | D07538 | hsa1901 | D10001 |
| hsa1129 | D01201 | hsa11255 | D03518 | hsa1910 | D07538 | hsa3356 | D10006 |
| hsa1131 | D01201 | hsa5739 | D03522 | hsa150 | D07540 | hsa154 | D10020 |
| hsa1132 | D01201 | hsa1813 | D03534 | hsa151 | D07540 | hsa1394 | D10022 |
| hsa1133 | D01201 | hsa3269 | D03535 | hsa152 | D07540 | hsa2740 | D10025 |
| hsa185 | D01204 | hsa1812 | D03556 | hsa3269 | D07543 | hsa3577 | D10075 |
| hsa150 | D01205 | hsa1813 | D03556 | hsa3269 | D07547 | hsa3579 | D10075 |
| hsa151 | D01205 | hsa4988 | D03579 | hsa146 | D07550 | hsa3061 | D10082 |
| hsa152 | D01205 | hsa4988 | D03580 | hsa147 | D07550 | hsa3062 | D10082 |
| hsa554 | D01213 | hsa154 | D03587 | hsa148 | D07550 | hsa3362 | D10099 |
| hsa146 | D01224 | hsa1394 | D03591 | hsa4988 | D07557 | hsa3362 | D10100 |
| hsa147 | D01224 | hsa1395 | D03591 | hsa146 | D07560 | hsa5021 | D10117 |
| hsa148 | D01224 | hsa1394 | D03592 | hsa147 | D07560 | hsa5021 | D10118 |
| hsa150 | D01224 | hsa1395 | D03592 | hsa148 | D07560 | hsa6608 | D10119 |
| hsa151 | D01224 | hsa4158 | D03593 | hsa1813 | D07563 | hsa11255 | D10128 |
| hsa152 | D01224 | hsa4986 | D03618 | hsa153 | D07590 | hsa11255 | D10129 |
| hsa153 | D01224 | hsa4988 | D03618 | hsa154 | D07590 | hsa1909 | D10135 |
| hsa154 | D01224 | hsa3269 | D03621 | hsa155 | D07590 | hsa1910 | D10135 |
| hsa155 | D01224 | hsa3269 | D03622 | hsa1813 | D07593 | hsa154 | D10145 |
| hsa1813 | D01226 | hsa6915 | D03642 | hsa3350 | D07593 | hsa6751 | D10147 |
| hsa1909 | D01227 | hsa146 | D03648 | hsa134 | D07603 | hsa6752 | D10147 |
| hsa1910 | D01227 | hsa147 | D03648 | hsa135 | D07603 | hsa6753 | D10147 |
| hsa1128 | D01231 | hsa148 | D03648 | hsa154 | D07608 | hsa6755 | D10147 |
| hsa1129 | D01231 | hsa1131 | D03654 | hsa3269 | D07617 | hsa3360 | D10152 |
| hsa1131 | D01231 | hsa150 | D03672 | hsa4988 | D07620 | hsa4988 | D10162 |
| hsa1132 | D01231 | hsa151 | D03672 | hsa153 | D07624 | hsa4988 | D10163 |
| hsa1133 | D01231 | hsa152 | D03672 | hsa154 | D07624 | hsa3358 | D10170 |
| hsa552 | D01236 | hsa624 | D03677 | hsa155 | D07624 | hsa3358 | D10171 |
| hsa554 | D01236 | hsa5029 | D03685 | hsa153 | D07660 | hsa135 | D10174 |
| hsa3269 | D01242 | hsa3269 | D03693 | hsa3269 | D07662 | hsa1128 | D10180 |
| hsa154 | D01263 | hsa150 | D03702 | hsa2798 | D07665 | hsa1129 | D10180 |
| hsa1131 | D01269 | hsa151 | D03702 | hsa1131 | D07667 | hsa1131 | D10180 |
| hsa146 | D01277 | hsa152 | D03702 | hsa3269 | D07679 | hsa1132 | D10180 |
| hsa147 | D01277 | hsa3269 | D03704 | hsa1909 | D07682 | hsa1133 | D10180 |
| hsa148 | D01277 | hsa1128 | D03711 | hsa3269 | D07684 | hsa1128 | D10181 |
| hsa150 | D01277 | hsa1129 | D03711 | hsa1812 | D07686 | hsa1129 | D10181 |
| hsa151 | D01277 | hsa1131 | D03711 | hsa1813 | D07686 | hsa1131 | D10181 |
| hsa152 | D01277 | hsa1132 | D03711 | hsa1812 | D07687 | hsa1132 | D10181 |
| hsa153 | D01277 | hsa1133 | D03711 | hsa1813 | D07687 | hsa1133 | D10181 |
| hsa154 | D01277 | hsa4988 | D03725 | hsa3350 | D07700 | hsa3350 | D10184 |
| hsa155 | D01277 | hsa153 | D03729 | hsa3351 | D07700 | hsa3350 | D10185 |
| hsa146 | D01290 | hsa154 | D03729 | hsa3352 | D07700 | hsa4988 | D10188 |
| hsa147 | D01290 | hsa155 | D03729 | hsa3354 | D07700 | hsa4988 | D10199 |
| hsa148 | D01290 | hsa153 | D03759 | hsa3355 | D07700 | hsa154 | D10219 |
| hsa3269 | D01295 | hsa154 | D03759 | hsa3356 | D07700 | hsa154 | D10220 |
| hsa1128 | D01297 | hsa155 | D03759 | hsa3357 | D07700 | hsa3269 | D10230 |
| hsa1129 | D01297 | hsa4985 | D03783 | hsa3358 | D07700 | hsa1813 | D10309 |
| hsa1131 | D01297 | hsa4986 | D03783 | hsa3360 | D07700 | hsa3350 | D10309 |
| hsa1132 | D01297 | hsa4988 | D03783 | hsa3350 | D07701 | hsa3356 | D10309 |
| hsa1133 | D01297 | hsa4988 | D03809 | hsa3351 | D07701 | hsa6608 | D10324 |
| hsa146 | D01307 | hsa1128 | D03814 | hsa3352 | D07701 | hsa6608 | D10325 |
| hsa147 | D01307 | hsa1129 | D03814 | hsa3354 | D07701 | hsa5729 | D10326 |
| hsa148 | D01307 | hsa1131 | D03814 | hsa3355 | D07701 | hsa1812 | D10329 |
| hsa1128 | D01315 | hsa1132 | D03814 | hsa3356 | D07701 | hsa1813 | D10329 |
| hsa1129 | D01315 | hsa1133 | D03814 | hsa3357 | D07701 | hsa3356 | D10329 |
| hsa1131 | D01315 | hsa1128 | D03824 | hsa3358 | D07701 | hsa2862 | D10330 |
| hsa1132 | D01315 | hsa1129 | D03824 | hsa3360 | D07701 | hsa3579 | D10332 |
| hsa1133 | D01315 | hsa1131 | D03824 | hsa154 | D07713 | hsa2864 | D10336 |
| hsa3269 | D01317 | hsa1132 | D03824 | hsa1813 | D07718 | hsa3355 | D10338 |
| hsa1128 | D01318 | hsa1133 | D03824 | hsa64805 | D07729 | hsa3061 | D10345 |
| hsa1129 | D01318 | hsa4988 | D03825 | hsa5737 | D07730 | hsa3062 | D10345 |
| hsa1131 | D01318 | hsa3269 | D03854 | hsa3269 | D07734 | hsa4988 | D10349 |
| hsa1132 | D01318 | hsa1128 | D03858 | hsa4988 | D07740 | hsa1813 | D10355 |
| hsa1133 | D01318 | hsa1129 | D03858 | hsa1909 | D07741 | hsa1814 | D10355 |
| hsa1813 | D01321 | hsa1131 | D03858 | hsa552 | D07748 | hsa3350 | D10355 |
| hsa3350 | D01321 | hsa1132 | D03858 | hsa554 | D07748 | hsa3356 | D10355 |
| hsa3351 | D01321 | hsa1133 | D03858 | hsa3356 | D07756 | hsa135 | D10362 |
| hsa3352 | D01321 | hsa1128 | D03859 | hsa3358 | D07756 | hsa1813 | D10364 |
| hsa3354 | D01321 | hsa1129 | D03859 | hsa1128 | D07759 | hsa1814 | D10364 |
| hsa3355 | D01321 | hsa1131 | D03859 | hsa1129 | D07759 | hsa3350 | D10364 |
| hsa3269 | D01324 | hsa1132 | D03859 | hsa1131 | D07759 | hsa3356 | D10364 |
| hsa3269 | D01332 | hsa1133 | D03859 | hsa1132 | D07759 | hsa4988 | D10371 |
| hsa146 | D01333 | hsa4988 | D03860 | hsa1133 | D07759 | hsa4988 | D10375 |
| hsa147 | D01333 | hsa5029 | D03864 | hsa3269 | D07765 | hsa10803 | D10378 |
| hsa148 | D01333 | hsa153 | D03879 | hsa146 | D07775 | hsa10803 | D10379 |
| hsa3269 | D01336 | hsa153 | D03880 | hsa147 | D07775 | hsa3579 | D10387 |
| hsa5739 | D01337 | hsa153 | D03881 | hsa148 | D07775 | hsa6869 | D10391 |
| hsa4985 | D01340 | hsa146 | D03887 | hsa3269 | D07790 | hsa4985 | D10403 |
| hsa4986 | D01340 | hsa147 | D03887 | hsa150 | D07795 | hsa4986 | D10403 |
| hsa4988 | D01340 | hsa148 | D03887 | hsa151 | D07795 | hsa4988 | D10403 |
| hsa3269 | D01343 | hsa150 | D03887 | hsa152 | D07795 | hsa8324 | D10410 |
| hsa146 | D01347 | hsa151 | D03887 | hsa3269 | D07803 | hsa3579 | D10420 |
| hsa147 | D01347 | hsa152 | D03887 | hsa1128 | D07804 | hsa3355 | D10424 |
| hsa148 | D01347 | hsa3274 | D03889 | hsa1129 | D07804 | hsa5739 | D10430 |
| hsa150 | D01347 | hsa154 | D03891 | hsa1131 | D07804 | hsa155 | D10433 |
| hsa151 | D01347 | hsa1812 | D03891 | hsa1132 | D07804 | hsa4987 | D10436 |
| hsa152 | D01347 | hsa1813 | D03891 | hsa1133 | D07804 | hsa4988 | D10436 |
| hsa1813 | D01348 | hsa1814 | D03891 | hsa4988 | D07809 | hsa5737 | D10441 |
| hsa154 | D01349 | hsa1815 | D03891 | hsa4988 | D07810 | hsa4988 | D10478 |
| hsa5737 | D01352 | hsa1816 | D03891 | hsa1128 | D07820 | hsa4988 | D10479 |
| hsa4988 | D01355 | hsa154 | D03892 | hsa1129 | D07820 | hsa59350 | D10488 |
| hsa150 | D01358 | hsa1812 | D03892 | hsa1131 | D07820 | hsa134 | D10493 |
| hsa151 | D01358 | hsa1813 | D03892 | hsa1128 | D07821 | hsa1812 | D10495 |
| hsa152 | D01358 | hsa1814 | D03892 | hsa1129 | D07821 | hsa1813 | D10495 |
| hsa1128 | D01359 | hsa1815 | D03892 | hsa1131 | D07821 | hsa3356 | D10495 |
| hsa1129 | D01359 | hsa1816 | D03892 | hsa1132 | D07821 | hsa6751 | D10497 |
| hsa1131 | D01359 | hsa3269 | D03893 | hsa1133 | D07821 | hsa6752 | D10497 |
| hsa1132 | D01359 | hsa135 | D03898 | hsa1128 | D07823 | hsa6753 | D10497 |
| hsa1133 | D01359 | hsa1394 | D03905 | hsa1129 | D07823 | hsa6755 | D10497 |
| hsa154 | D01360 | hsa1395 | D03905 | hsa1131 | D07823 | hsa3269 | D10510 |
| hsa154 | D01362 | hsa146 | D03914 | hsa1132 | D07823 | hsa1813 | D10516 |
| hsa146 | D01369 | hsa147 | D03914 | hsa1133 | D07823 | hsa1814 | D10516 |
| hsa147 | D01369 | hsa148 | D03914 | hsa4988 | D07831 | hsa3350 | D10516 |
| hsa148 | D01369 | hsa153 | D03914 | hsa4988 | D07832 | hsa3356 | D10516 |
| hsa153 | D01369 | hsa1812 | D03937 | hsa146 | D07833 | hsa1128 | D10545 |
| hsa154 | D01373 | hsa1816 | D03937 | hsa147 | D07833 | hsa1129 | D10545 |
| hsa1128 | D01377 | hsa1909 | D03953 | hsa148 | D07833 | hsa1131 | D10545 |
| hsa1129 | D01377 | hsa3269 | D03963 | hsa150 | D07833 | hsa1132 | D10545 |
| hsa1131 | D01377 | hsa2912 | D03966 | hsa151 | D07833 | hsa1133 | D10545 |
| hsa1132 | D01377 | hsa1128 | D03970 | hsa152 | D07833 | hsa154 | D10545 |
| hsa1133 | D01377 | hsa1129 | D03970 | hsa146 | D07834 | hsa1128 | D10546 |
| hsa4988 | D01383 | hsa1131 | D03970 | hsa147 | D07834 | hsa1129 | D10546 |
| hsa10800 | D01385 | hsa1132 | D03970 | hsa148 | D07834 | hsa1131 | D10546 |
| hsa5739 | D01385 | hsa1133 | D03970 | hsa150 | D07834 | hsa1132 | D10546 |
| hsa146 | D01386 | hsa1128 | D03979 | hsa151 | D07834 | hsa1133 | D10546 |
| hsa147 | D01386 | hsa1129 | D03979 | hsa152 | D07834 | hsa154 | D10546 |
| hsa148 | D01386 | hsa1131 | D03979 | hsa1813 | D07835 | hsa185 | D10556 |
| hsa150 | D01386 | hsa1132 | D03979 | hsa1813 | D07836 | hsa2693 | D10562 |
| hsa151 | D01386 | hsa1133 | D03979 | hsa3351 | D07837 | hsa2693 | D10563 |
| hsa152 | D01386 | hsa3351 | D03982 | hsa3352 | D07837 | hsa6751 | D10566 |
| hsa153 | D01386 | hsa3352 | D03982 | hsa3351 | D07838 | hsa6752 | D10566 |
| hsa154 | D01386 | hsa3351 | D03983 | hsa3352 | D07838 | hsa6753 | D10566 |
| hsa155 | D01386 | hsa3352 | D03983 | hsa1129 | D07853 | hsa6755 | D10566 |
| hsa153 | D01390 | hsa5731 | D03999 | hsa3269 | D07853 | hsa2864 | D10567 |
| hsa154 | D01390 | hsa134 | D04006 | hsa3269 | D07854 | hsa7201 | D10594 |
| hsa155 | D01390 | hsa135 | D04006 | hsa3356 | D07854 | hsa4988 | D10612 |
| hsa4988 | D01399 | hsa136 | D04006 | hsa3357 | D07854 | hsa5739 | D10628 |
| hsa1813 | D01412 | hsa146 | D04018 | hsa3358 | D07854 | hsa5729 | D10631 |
| hsa154 | D01428 | hsa147 | D04018 | hsa3269 | D07860 | hsa6608 | D10636 |
| hsa4988 | D01435 | hsa148 | D04018 | hsa4988 | D07861 | hsa5734 | D10638 |
| hsa146 | D01444 | hsa150 | D04018 | hsa3269 | D07862 | hsa4988 | D10648 |
| hsa147 | D01444 | hsa151 | D04018 | hsa4986 | D07863 | hsa2693 | D10660 |
| hsa148 | D01444 | hsa152 | D04018 | hsa4988 | D07863 | hsa10203 | D10662 |
| hsa153 | D01444 | hsa153 | D04018 | hsa4986 | D07864 | hsa10203 | D10663 |
| hsa154 | D01444 | hsa154 | D04018 | hsa4988 | D07864 | hsa6608 | D10671 |
| hsa155 | D01444 | hsa155 | D04018 | hsa1813 | D07868 | hsa10203 | D10673 |
| hsa146 | D01445 | hsa146 | D04026 | hsa1812 | D07870 | hsa846 | D10676 |
| hsa147 | D01445 | hsa147 | D04026 | hsa1813 | D07870 | hsa846 | D10677 |
| hsa148 | D01445 | hsa148 | D04026 | hsa1814 | D07870 | hsa4988 | D10690 |
| hsa1813 | D01447 | hsa150 | D04026 | hsa1815 | D07870 | hsa4988 | D10692 |
| hsa1813 | D01448 | hsa151 | D04026 | hsa1816 | D07870 | hsa5739 | D10703 |
| hsa1128 | D01451 | hsa152 | D04026 | hsa146 | D07874 | hsa3358 | D10710 |
| hsa1129 | D01451 | hsa153 | D04026 | hsa147 | D07874 | hsa3362 | D10710 |
| hsa1131 | D01451 | hsa154 | D04026 | hsa148 | D07874 | hsa5739 | D10725 |
| hsa1132 | D01451 | hsa155 | D04026 | hsa3269 | D07875 | hsa6608 | D10729 |
| hsa1133 | D01451 | hsa1128 | D04034 | hsa3269 | D07878 | hsa59340 | D10735 |
| hsa5737 | D01452 | hsa1129 | D04034 | hsa3351 | D07887 | hsa59340 | D10736 |
| hsa134 | D01453 | hsa1131 | D04034 | hsa3352 | D07887 | hsa4988 | D10740 |
| hsa135 | D01453 | hsa1132 | D04034 | hsa3269 | D07889 | hsa6869 | D10742 |
| hsa153 | D01454 | hsa1133 | D04034 | hsa3269 | D07890 | hsa3362 | D10747 |
| hsa154 | D01454 | hsa150 | D04034 | hsa3269 | D07900 | hsa11255 | D10749 |
| hsa155 | D01454 | hsa151 | D04034 | hsa4986 | D07904 | hsa4988 | D10811 |
| hsa3269 | D01460 | hsa152 | D04034 | hsa4988 | D07904 | hsa4985 | D10812 |
| hsa1813 | D01462 | hsa1813 | D04034 | hsa146 | D07905 | hsa4986 | D10812 |
| hsa1814 | D01462 | hsa3269 | D04034 | hsa147 | D07905 | hsa4988 | D10812 |
| hsa1815 | D01462 | hsa3356 | D04034 | hsa148 | D07905 | hsa64805 | D10823 |
| hsa1128 | D01463 | hsa3357 | D04034 | hsa150 | D07905 | hsa64805 | D10824 |
| hsa1129 | D01463 | hsa3358 | D04034 | hsa151 | D07905 | hsa1131 | D10853 |
| hsa1131 | D01463 | hsa1813 | D04038 | hsa152 | D07905 | hsa5745 | D10885 |
| hsa1132 | D01463 | hsa185 | D04040 | hsa146 | D07906 | hsa6869 | D10895 |
| hsa1133 | D01463 | hsa3269 | D04055 | hsa147 | D07906 | hsa2912 | D10896 |
| hsa3274 | D01467 | hsa3356 | D04055 | hsa148 | D07906 | hsa2913 | D10896 |
| hsa1813 | D01468 | hsa1128 | D04057 | hsa3350 | D07906 | hsa1901 | D10904 |
| hsa146 | D01469 | hsa1129 | D04057 | hsa153 | D07916 | hsa1903 | D10904 |
| hsa147 | D01469 | hsa1131 | D04057 | hsa4988 | D07929 | hsa53637 | D10904 |
| hsa148 | D01469 | hsa154 | D04072 | hsa146 | D07931 | hsa8698 | D10904 |
| hsa153 | D01469 | hsa1241 | D04074 | hsa147 | D07931 | hsa9294 | D10904 |
| hsa154 | D01469 | hsa56413 | D04074 | hsa148 | D07931 | hsa1901 | D10905 |
| hsa153 | D01471 | hsa1128 | D04087 | hsa150 | D07931 | hsa1903 | D10905 |
| hsa1813 | D01477 | hsa1129 | D04087 | hsa151 | D07931 | hsa53637 | D10905 |
| hsa3269 | D01477 | hsa1131 | D04087 | hsa152 | D07931 | hsa8698 | D10905 |
| hsa3356 | D01477 | hsa1132 | D04087 | hsa153 | D07931 | hsa9294 | D10905 |
| hsa3357 | D01477 | hsa1133 | D04087 | hsa154 | D07931 | hsa1268 | D10915 |
| hsa3358 | D01477 | hsa1812 | D04097 | hsa155 | D07931 | hsa1269 | D10915 |
| hsa3269 | D01478 | hsa1813 | D04097 | hsa4985 | D07937 | hsa10203 | D10928 |
| hsa4988 | D01481 | hsa1814 | D04097 | hsa4986 | D07937 | hsa1901 | D10930 |
| hsa1813 | D01482 | hsa1815 | D04097 | hsa4988 | D07937 | hsa1903 | D10930 |
| hsa146 | D01485 | hsa1816 | D04097 | hsa3356 | D07945 | hsa53637 | D10930 |
| hsa147 | D01485 | hsa3274 | D04098 | hsa3357 | D07945 | hsa8698 | D10930 |
| hsa148 | D01485 | hsa1128 | D04116 | hsa3358 | D07945 | hsa9294 | D10930 |
| hsa1813 | D01485 | hsa1129 | D04116 | hsa1812 | D07946 | hsa1131 | D10938 |
| hsa3356 | D01485 | hsa1131 | D04116 | hsa1128 | D07951 | hsa1131 | D10939 |
| hsa3357 | D01485 | hsa1132 | D04116 | hsa1129 | D07951 | hsa5732 | D10965 |
| hsa3358 | D01485 | hsa1133 | D04116 | hsa1131 | D07951 | hsa5732 | D10966 |
| hsa1128 | D01491 | hsa153 | D04120 | hsa1132 | D07951 | hsa1901 | D10967 |
| hsa1129 | D01491 | hsa154 | D04120 | hsa1133 | D07951 | hsa1903 | D10967 |
| hsa1131 | D01491 | hsa155 | D04120 | hsa2798 | D07957 | hsa53637 | D10967 |
| hsa1132 | D01491 | hsa2740 | D04121 | hsa3269 | D07958 | hsa8698 | D10967 |
| hsa1133 | D01491 | hsa6869 | D04122 | hsa1812 | D07976 | hsa9294 | D10967 |
| hsa153 | D01492 | hsa150 | D04124 | hsa1813 | D07976 | hsa1901 | D10968 |
| hsa154 | D01492 | hsa151 | D04124 | hsa1813 | D07977 | hsa1903 | D10968 |
| hsa4986 | D01500 | hsa152 | D04124 | hsa154 | D07990 | hsa53637 | D10968 |
| hsa4988 | D01500 | hsa185 | D04126 | hsa3351 | D07997 | hsa8698 | D10968 |
| hsa153 | D01504 | hsa186 | D04126 | hsa3352 | D07997 | hsa9294 | D10968 |
| hsa154 | D01504 | hsa1909 | D04128 | hsa2798 | D08010 | hsa2740 | D10970 |
| hsa155 | D01504 | hsa552 | D04137 | hsa4889 | D08026 | hsa140 | D10971 |
| hsa1813 | D01505 | hsa3356 | D04148 | hsa2798 | D08027 | hsa729230 | D10972 |
| hsa153 | D01512 | hsa3357 | D04148 | hsa146 | D08029 | hsa1128 | D10978 |
| hsa154 | D01512 | hsa3358 | D04148 | hsa147 | D08029 | hsa1129 | D10978 |
| hsa155 | D01512 | hsa2915 | D04155 | hsa148 | D08029 | hsa1131 | D10978 |
| hsa1813 | D01520 | hsa154 | D04157 | hsa150 | D08029 | hsa1132 | D10978 |
| hsa1128 | D01521 | hsa148 | D04184 | hsa151 | D08029 | hsa1133 | D10978 |
| hsa1129 | D01521 | hsa1901 | D04187 | hsa152 | D08029 | hsa1128 | D10989 |
| hsa1131 | D01521 | hsa153 | D04190 | hsa150 | D08031 | hsa1129 | D10989 |
| hsa1132 | D01521 | hsa154 | D04190 | hsa1813 | D08035 | hsa1131 | D10989 |
| hsa1133 | D01521 | hsa155 | D04190 | hsa154 | D08039 | hsa1132 | D10989 |
| hsa1813 | D01522 | hsa150 | D04223 | hsa11255 | D08040 | hsa1133 | D10989 |
| hsa1128 | D01532 | hsa151 | D04223 | hsa3269 | D08040 | hsa154 | D11009 |
| hsa1129 | D01532 | hsa152 | D04223 | hsa3274 | D08040 | hsa6752 | D11033 |
| hsa1131 | D01532 | hsa185 | D04243 | hsa59340 | D08040 | hsa150 | D11056 |
| hsa1132 | D01532 | hsa3351 | D04264 | hsa3269 | D08041 | hsa151 | D11056 |
| hsa1133 | D01532 | hsa3352 | D04264 | hsa4988 | D08045 | hsa152 | D11056 |
| hsa3350 | D01537 | hsa2798 | D04302 | hsa4988 | D08046 | hsa3356 | D11056 |
| hsa1128 | D01538 | hsa3350 | D04314 | hsa4988 | D08047 | hsa3357 | D11056 |
| hsa1129 | D01538 | hsa3356 | D04320 | hsa3269 | D08054 | hsa3358 | D11056 |
| hsa1131 | D01538 | hsa3357 | D04320 | hsa1812 | D08057 | hsa846 | D11063 |
| hsa1132 | D01538 | hsa3358 | D04320 | hsa1813 | D08057 | hsa6869 | D11065 |
| hsa1133 | D01538 | hsa2798 | D04361 | hsa146 | D08064 | hsa1901 | D11072 |
| hsa1813 | D01548 | hsa150 | D04375 | hsa147 | D08064 | hsa53637 | D11072 |
| hsa1814 | D01548 | hsa151 | D04375 | hsa148 | D08064 | hsa1815 | D01548 |

Table4. The overall of drug-target interactions for nuclear receptors class on new dataset 1

| **drug** | **protein** | **drug** | **protein** | **drug** | **protein** | **drug** | **protein** |
| --- | --- | --- | --- | --- | --- | --- | --- |
| hsa5241 | D00066 | hsa5241 | D01217 | hsa2099 | D03675 | hsa2908 | D07495 |
| hsa2099 | D00067 | hsa2908 | D01229 | hsa2100 | D03675 | hsa367 | D07536 |
| hsa2100 | D00067 | hsa2908 | D01239 | hsa367 | D03675 | hsa7421 | D07578 |
| hsa367 | D00075 | hsa2099 | D01265 | hsa2908 | D03691 | hsa367 | D07670 |
| hsa2908 | D00088 | hsa2100 | D01265 | hsa2908 | D03696 | hsa5241 | D07670 |
| hsa5914 | D00094 | hsa2908 | D01266 | hsa2908 | D03697 | hsa2908 | D07715 |
| hsa5915 | D00094 | hsa2908 | D01272 | hsa4306 | D03698 | hsa2908 | D07717 |
| hsa5916 | D00094 | hsa2908 | D01273 | hsa4306 | D03699 | hsa2908 | D07719 |
| hsa2099 | D00105 | hsa5465 | D01274 | hsa7067 | D03750 | hsa5465 | D07724 |
| hsa2100 | D00105 | hsa5468 | D01274 | hsa7068 | D03750 | hsa2099 | D07726 |
| hsa7421 | D00122 | hsa2099 | D01294 | hsa4306 | D03792 | hsa2100 | D07726 |
| hsa7421 | D00129 | hsa5241 | D01294 | hsa5241 | D03799 | hsa2908 | D07749 |
| hsa9971 | D00163 | hsa367 | D01299 | hsa2908 | D03812 | hsa367 | D07766 |
| hsa5914 | D00164 | hsa5241 | D01299 | hsa2908 | D03813 | hsa367 | D07783 |
| hsa5915 | D00164 | hsa367 | D01301 | hsa2099 | D03911 | hsa4306 | D07792 |
| hsa5916 | D00164 | hsa2908 | D01308 | hsa2100 | D03911 | hsa2908 | D07796 |
| hsa2908 | D00165 | hsa2908 | D01327 | hsa2099 | D03912 | hsa2908 | D07797 |
| hsa5241 | D00182 | hsa367 | D01329 | hsa2100 | D03912 | hsa2908 | D07798 |
| hsa2099 | D00185 | hsa2908 | D01357 | hsa4306 | D03917 | hsa2908 | D07799 |
| hsa2100 | D00185 | hsa5465 | D01366 | hsa5241 | D03917 | hsa2908 | D07800 |
| hsa7421 | D00187 | hsa2908 | D01367 | hsa7421 | D03930 | hsa2908 | D07801 |
| hsa7421 | D00188 | hsa367 | D01368 | hsa5468 | D03941 | hsa2908 | D07802 |
| hsa2908 | D00244 | hsa5241 | D01374 | hsa5468 | D03996 | hsa2099 | D07826 |
| hsa2908 | D00246 | hsa367 | D01375 | hsa2099 | D04041 | hsa2100 | D07826 |
| hsa2099 | D00269 | hsa2908 | D01387 | hsa2100 | D04041 | hsa2908 | D07827 |
| hsa2100 | D00269 | hsa2908 | D01402 | hsa2099 | D04061 | hsa2099 | D07918 |
| hsa5465 | D00279 | hsa2099 | D01413 | hsa2100 | D04061 | hsa2100 | D07918 |
| hsa2099 | D00289 | hsa2100 | D01413 | hsa2099 | D04063 | hsa2099 | D07919 |
| hsa367 | D00289 | hsa5241 | D01414 | hsa2100 | D04063 | hsa2100 | D07919 |
| hsa5241 | D00289 | hsa5914 | D01418 | hsa2099 | D04064 | hsa2099 | D07920 |
| hsa2908 | D00292 | hsa2908 | D01434 | hsa2100 | D04064 | hsa2100 | D07920 |
| hsa7421 | D00299 | hsa2908 | D01442 | hsa2099 | D04065 | hsa2099 | D07921 |
| hsa2099 | D00312 | hsa2908 | D01464 | hsa2100 | D04065 | hsa2100 | D07921 |
| hsa2100 | D00312 | hsa7421 | D01472 | hsa2099 | D04071 | hsa2099 | D07928 |
| hsa5914 | D00316 | hsa367 | D01476 | hsa2100 | D04071 | hsa2099 | D07939 |
| hsa5915 | D00316 | hsa2908 | D01510 | hsa2099 | D04104 | hsa5241 | D07939 |
| hsa5916 | D00316 | hsa5914 | D01516 | hsa5241 | D04104 | hsa4306 | D07967 |
| hsa6256 | D00316 | hsa5915 | D01516 | hsa5468 | D04132 | hsa2908 | D07972 |
| hsa6257 | D00316 | hsa5916 | D01516 | hsa5914 | D04162 | hsa2908 | D07973 |
| hsa6258 | D00316 | hsa7421 | D01518 | hsa5915 | D04162 | hsa2908 | D07975 |
| hsa2908 | D00324 | hsa367 | D01533 | hsa5916 | D04162 | hsa2908 | D07980 |
| hsa2908 | D00325 | hsa367 | D01534 | hsa2908 | D04201 | hsa2908 | D07981 |
| hsa367 | D00327 | hsa5241 | D01580 | hsa2908 | D04208 | hsa2908 | D08036 |
| hsa2908 | D00328 | hsa2099 | D01602 | hsa2908 | D04214 | hsa5241 | D08052 |
| hsa5465 | D00334 | hsa2100 | D01602 | hsa2908 | D04217 | hsa5241 | D08053 |
| hsa5914 | D00348 | hsa2908 | D01615 | hsa2908 | D04218 | hsa7067 | D08125 |
| hsa5915 | D00348 | hsa2099 | D01616 | hsa2908 | D04219 | hsa7068 | D08125 |
| hsa5916 | D00348 | hsa2100 | D01616 | hsa2908 | D04221 | hsa7067 | D08128 |
| hsa2908 | D00385 | hsa367 | D01616 | hsa2908 | D04227 | hsa7068 | D08128 |
| hsa4306 | D00386 | hsa2099 | D01617 | hsa2908 | D04228 | hsa7067 | D08129 |
| hsa367 | D00389 | hsa2100 | D01617 | hsa2908 | D04244 | hsa7068 | D08129 |
| hsa5468 | D00395 | hsa2908 | D01619 | hsa5241 | D04315 | hsa2908 | D08147 |
| hsa2908 | D00407 | hsa5914 | D01621 | hsa5241 | D04316 | hsa5241 | D08166 |
| hsa367 | D00408 | hsa5915 | D01621 | hsa2908 | D04409 | hsa5241 | D08167 |
| hsa4306 | D00443 | hsa5916 | D01621 | hsa2908 | D04467 | hsa367 | D08193 |
| hsa367 | D00444 | hsa2908 | D01632 | hsa2099 | D04496 | hsa367 | D08196 |
| hsa367 | D00462 | hsa2908 | D01637 | hsa2100 | D04496 | hsa367 | D08197 |
| hsa2908 | D00472 | hsa2099 | D01639 | hsa5914 | D04636 | hsa2908 | D08227 |
| hsa2908 | D00473 | hsa2099 | D01641 | hsa5915 | D04636 | hsa367 | D08250 |
| hsa367 | D00490 | hsa2100 | D01641 | hsa5916 | D04636 | hsa367 | D08251 |
| hsa2099 | D00554 | hsa7421 | D01662 | hsa2099 | D04672 | hsa9971 | D08277 |
| hsa5465 | D00565 | hsa2908 | D01689 | hsa2100 | D04672 | hsa5241 | D08281 |
| hsa2099 | D00575 | hsa2908 | D01703 | hsa5241 | D04885 | hsa5241 | D08285 |
| hsa2100 | D00575 | hsa2908 | D01708 | hsa367 | D04947 | hsa2099 | D08301 |
| hsa2099 | D00576 | hsa367 | D01737 | hsa2908 | D05000 | hsa2100 | D08301 |
| hsa2099 | D00577 | hsa2908 | D01743 | hsa2908 | D05001 | hsa5468 | D08378 |
| hsa2100 | D00577 | hsa2908 | D01764 | hsa2908 | D05002 | hsa2099 | D08409 |
| hsa2908 | D00585 | hsa2908 | D01820 | hsa5241 | D05003 | hsa2100 | D08409 |
| hsa5241 | D00585 | hsa2908 | D01825 | hsa4306 | D05020 | hsa367 | D08409 |
| hsa367 | D00586 | hsa2099 | D01853 | hsa367 | D05025 | hsa2908 | D08412 |
| hsa5468 | D00596 | hsa2100 | D01853 | hsa5468 | D05030 | hsa2908 | D08413 |
| hsa7421 | D00628 | hsa2908 | D01886 | hsa5465 | D05091 | hsa2908 | D08414 |
| hsa2908 | D00689 | hsa4306 | D01943 | hsa5468 | D05091 | hsa2908 | D08415 |
| hsa2908 | D00690 | hsa2908 | D01948 | hsa2099 | D05106 | hsa2908 | D08416 |
| hsa9971 | D00734 | hsa2099 | D01953 | hsa2100 | D05106 | hsa2908 | D08429 |
| hsa2908 | D00751 | hsa2100 | D01953 | hsa367 | D05116 | hsa5241 | D08429 |
| hsa2099 | D00898 | hsa5468 | D01971 | hsa5468 | D05150 | hsa5241 | D08431 |
| hsa2100 | D00898 | hsa2099 | D01986 | hsa367 | D05174 | hsa2099 | D08465 |
| hsa7421 | D00930 | hsa2100 | D01986 | hsa2099 | D05192 | hsa2100 | D08465 |
| hsa5468 | D00945 | hsa2099 | D01989 | hsa2100 | D05192 | hsa2908 | D08476 |
| hsa2099 | D00946 | hsa2100 | D01989 | hsa5241 | D05209 | hsa5914 | D08477 |
| hsa2100 | D00946 | hsa2908 | D01998 | hsa5465 | D05397 | hsa5915 | D08477 |
| hsa2099 | D00948 | hsa2099 | D02018 | hsa5468 | D05397 | hsa5916 | D08477 |
| hsa2100 | D00948 | hsa2100 | D02018 | hsa2908 | D05601 | hsa5468 | D08491 |
| hsa5241 | D00949 | hsa2908 | D02032 | hsa2908 | D05603 | hsa7421 | D08554 |
| hsa5241 | D00950 | hsa2908 | D02156 | hsa4306 | D05640 | hsa2099 | D08559 |
| hsa5241 | D00951 | hsa2908 | D02174 | hsa367 | D05674 | hsa2100 | D08559 |
| hsa5241 | D00952 | hsa2099 | D02217 | hsa5241 | D05679 | hsa367 | D08573 |
| hsa5241 | D00953 | hsa2100 | D02217 | hsa2908 | D05719 | hsa367 | D08574 |
| hsa5241 | D00954 | hsa2908 | D02286 | hsa2908 | D05729 | hsa2908 | D08610 |
| hsa367 | D00955 | hsa2908 | D02287 | hsa5468 | D05739 | hsa2099 | D08620 |
| hsa367 | D00956 | hsa2908 | D02288 | hsa367 | D05837 | hsa2100 | D08620 |
| hsa367 | D00957 | hsa2908 | D02289 | hsa4306 | D05912 | hsa2908 | D08660 |
| hsa367 | D00958 | hsa2099 | D02367 | hsa5241 | D06003 | hsa5465 | D08845 |
| hsa367 | D00959 | hsa5241 | D02367 | hsa367 | D06085 | hsa5468 | D08845 |
| hsa367 | D00961 | hsa2908 | D02591 | hsa367 | D06086 | hsa7421 | D08868 |
| hsa2099 | D00962 | hsa2908 | D02592 | hsa367 | D06087 | hsa5465 | D08890 |
| hsa2100 | D00962 | hsa5914 | D02754 | hsa5465 | D06102 | hsa2099 | D08910 |
| hsa367 | D00965 | hsa5915 | D02754 | hsa2908 | D06135 | hsa2100 | D08910 |
| hsa2099 | D00966 | hsa5916 | D02754 | hsa2908 | D06171 | hsa5465 | D08949 |
| hsa2100 | D00966 | hsa6256 | D02754 | hsa367 | D06191 | hsa2099 | D08958 |
| hsa2099 | D00967 | hsa6257 | D02754 | hsa2908 | D06201 | hsa5465 | D09350 |
| hsa2100 | D00967 | hsa6258 | D02754 | hsa2908 | D06216 | hsa5467 | D09350 |
| hsa2908 | D00972 | hsa2099 | D02758 | hsa2099 | D06245 | hsa5468 | D09350 |
| hsa2908 | D00973 | hsa2100 | D02758 | hsa2100 | D06245 | hsa9971 | D09360 |
| hsa2908 | D00975 | hsa5914 | D02815 | hsa2908 | D06315 | hsa5916 | D09365 |
| hsa2908 | D00976 | hsa5915 | D02815 | hsa367 | D06357 | hsa7068 | D09381 |
| hsa2908 | D00977 | hsa5916 | D02815 | hsa7067 | D06482 | hsa5241 | D09567 |
| hsa2908 | D00978 | hsa6256 | D02815 | hsa7068 | D06482 | hsa5241 | D09571 |
| hsa2908 | D00979 | hsa6257 | D02815 | hsa5914 | D06543 | hsa5468 | D09579 |
| hsa2908 | D00980 | hsa6258 | D02815 | hsa5915 | D06543 | hsa5241 | D09687 |
| hsa2908 | D00981 | hsa5241 | D02840 | hsa5916 | D06543 | hsa2908 | D09796 |
| hsa2908 | D00982 | hsa5241 | D02881 | hsa2099 | D06551 | hsa2099 | D09834 |
| hsa2908 | D00983 | hsa2099 | D02993 | hsa2100 | D06551 | hsa2100 | D09834 |
| hsa2908 | D00984 | hsa2100 | D02993 | hsa2100 | D06631 | hsa2100 | D09899 |
| hsa2908 | D00985 | hsa5241 | D02996 | hsa5465 | D06647 | hsa5241 | D09972 |
| hsa4306 | D00986 | hsa2099 | D03062 | hsa5467 | D06647 | hsa2099 | D10008 |
| hsa7421 | D01009 | hsa2100 | D03062 | hsa5468 | D06647 | hsa2100 | D10008 |
| hsa7067 | D01010 | hsa367 | D03079 | hsa2908 | D06673 | hsa5241 | D10016 |
| hsa7068 | D01010 | hsa6256 | D03106 | hsa2908 | D06876 | hsa2908 | D10136 |
| hsa7067 | D01011 | hsa6257 | D03106 | hsa2908 | D07073 | hsa7421 | D10198 |
| hsa7068 | D01011 | hsa6258 | D03106 | hsa5241 | D07096 | hsa367 | D10218 |
| hsa7067 | D01012 | hsa367 | D03144 | hsa2908 | D07116 | hsa367 | D10221 |
| hsa7068 | D01012 | hsa367 | D03145 | hsa367 | D07127 | hsa4306 | D10528 |
| hsa7421 | D01098 | hsa2908 | D03301 | hsa5465 | D07187 | hsa7421 | D10565 |
| hsa5915 | D01112 | hsa2908 | D03325 | hsa2908 | D07201 | hsa2099 | D10606 |
| hsa5916 | D01112 | hsa367 | D03338 | hsa2908 | D07202 | hsa2100 | D10606 |
| hsa6257 | D01112 | hsa4306 | D03363 | hsa2908 | D07203 | hsa2908 | D10617 |
| hsa6258 | D01112 | hsa5468 | D03493 | hsa7067 | D07214 | hsa4306 | D10633 |
| hsa4306 | D01115 | hsa367 | D03515 | hsa7068 | D07214 | hsa2908 | D10634 |
| hsa7421 | D01125 | hsa5465 | D03521 | hsa5241 | D07220 | hsa9971 | D10699 |
| hsa5914 | D01132 | hsa2908 | D03541 | hsa2099 | D07221 | hsa5465 | D10711 |
| hsa5915 | D01132 | hsa2099 | D03551 | hsa2100 | D07221 | hsa2099 | D10876 |
| hsa5916 | D01132 | hsa2100 | D03551 | hsa5241 | D07222 | hsa2100 | D10876 |
| hsa6257 | D01132 | hsa367 | D03551 | hsa5241 | D07223 | hsa5241 | D10986 |
| hsa367 | D01149 | hsa2908 | D03561 | hsa2908 | D07230 | hsa5241 | D10987 |
| hsa5241 | D01159 | hsa2908 | D03594 | hsa2099 | D07434 | hsa2908 | D11000 |
| hsa2099 | D01161 | hsa5468 | D03653 | hsa367 | D07456 | hsa367 | D11040 |
| hsa2100 | D01161 | hsa2908 | D03671 | hsa2908 | D07464 | hsa367 | D11045 |
| hsa5465 | D01208 |  |  |  |  |  |  |

Table5. The overall of drug-target interactions for Cytokines and receptors class on new dataset 1

| **drug** | **protein** | **drug** | **protein** | **drug** | **protein** | **drug** | **protein** |
| --- | --- | --- | --- | --- | --- | --- | --- |
| hsa4049 | D00742 | hsa7099 | D04043 | hsa10673 | D08877 | hsa729230 | D09879 |
| hsa7124 | D00742 | hsa3458 | D04242 | hsa958 | D08896 | hsa8795 | D09888 |
| hsa3454 | D00745 | hsa7124 | D04358 | hsa958 | D08942 | hsa51330 | D09896 |
| hsa3455 | D00745 | hsa3606 | D04487 | hsa7852 | D08971 | hsa3578 | D09897 |
| hsa3454 | D00746 | hsa3454 | D04552 | hsa3569 | D08976 | hsa4803 | D09907 |
| hsa3455 | D00746 | hsa3455 | D04552 | hsa7124 | D08976 | hsa3553 | D09911 |
| hsa3459 | D00747 | hsa3454 | D04553 | hsa3577 | D08984 | hsa1438 | D09930 |
| hsa3460 | D00747 | hsa3455 | D04553 | hsa3579 | D08984 | hsa7292 | D09943 |
| hsa3559 | D00748 | hsa3454 | D04554 | hsa3567 | D08985 | hsa7124 | D09944 |
| hsa3560 | D00748 | hsa3455 | D04554 | hsa4352 | D08990 | hsa2057 | D09946 |
| hsa3561 | D00748 | hsa943 | D04612 | hsa4352 | D09024 | hsa2057 | D09947 |
| hsa7124 | D00754 | hsa51284 | D04619 | hsa3559 | D09190 | hsa3605 | D09967 |
| hsa5617 | D00780 | hsa7124 | D04687 | hsa3592 | D09214 | hsa3596 | D09979 |
| hsa5617 | D00987 | hsa4049 | D04690 | hsa3593 | D09214 | hsa3604 | D09984 |
| hsa5617 | D01348 | hsa4050 | D04690 | hsa51561 | D09214 | hsa2057 | D09998 |
| hsa3552 | D01583 | hsa7124 | D04690 | hsa3553 | D09315 | hsa2057 | D10000 |
| hsa3553 | D01583 | hsa1441 | D04695 | hsa8795 | D09329 | hsa23765 | D10061 |
| hsa3569 | D01583 | hsa3563 | D04695 | hsa50615 | D09332 | hsa3605 | D10071 |
| hsa3586 | D01583 | hsa3592 | D04748 | hsa4803 | D09387 | hsa3577 | D10075 |
| hsa6347 | D01583 | hsa3593 | D04748 | hsa7099 | D09573 | hsa3579 | D10075 |
| hsa7124 | D01583 | hsa51284 | D04787 | hsa5228 | D09574 | hsa163702 | D10078 |
| hsa51284 | D02500 | hsa8797 | D04858 | hsa7422 | D09574 | hsa3569 | D10080 |
| hsa3570 | D02596 | hsa3479 | D04862 | hsa7423 | D09574 | hsa10673 | D10083 |
| hsa7124 | D02597 | hsa2690 | D04870 | hsa943 | D09587 | hsa3082 | D10123 |
| hsa7124 | D02598 | hsa3567 | D04923 | hsa3592 | D09588 | hsa3570 | D10161 |
| hsa2690 | D02691 | hsa3953 | D05014 | hsa3593 | D09588 | hsa284 | D10177 |
| hsa3559 | D02743 | hsa1441 | D05036 | hsa51561 | D09588 | hsa285 | D10177 |
| hsa3560 | D02743 | hsa3563 | D05036 | hsa50616 | D09615 | hsa4049 | D10187 |
| hsa3561 | D02743 | hsa1438 | D05066 | hsa7040 | D09620 | hsa1441 | D10242 |
| hsa3454 | D02744 | hsa3563 | D05090 | hsa7042 | D09620 | hsa1441 | D10310 |
| hsa3455 | D02744 | hsa7124 | D05146 | hsa7043 | D09620 | hsa7124 | D10320 |
| hsa3454 | D02745 | hsa7124 | D05263 | hsa3596 | D09633 | hsa3579 | D10332 |
| hsa3455 | D02745 | hsa3590 | D05266 | hsa1435 | D09635 | hsa970 | D10341 |
| hsa3454 | D02747 | hsa7422 | D05386 | hsa3082 | D09659 | hsa970 | D10342 |
| hsa3455 | D02747 | hsa1441 | D05389 | hsa7098 | D09661 | hsa2660 | D10347 |
| hsa3454 | D02748 | hsa7124 | D05393 | hsa3439 | D09662 | hsa3481 | D10352 |
| hsa3455 | D02748 | hsa2690 | D05394 | hsa3440 | D09662 | hsa3566 | D10354 |
| hsa3559 | D02749 | hsa7422 | D05697 | hsa3441 | D09662 | hsa7100 | D10368 |
| hsa3560 | D02749 | hsa1438 | D05712 | hsa3442 | D09662 | hsa10803 | D10378 |
| hsa3561 | D02749 | hsa959 | D05777 | hsa3443 | D09662 | hsa10803 | D10379 |
| hsa3554 | D02934 | hsa1438 | D05803 | hsa3444 | D09662 | hsa3579 | D10387 |
| hsa7850 | D02934 | hsa2690 | D05884 | hsa3445 | D09662 | hsa285 | D10395 |
| hsa10673 | D02979 | hsa958 | D06071 | hsa3446 | D09662 | hsa51561 | D10400 |
| hsa3559 | D03058 | hsa959 | D06193 | hsa3447 | D09662 | hsa3627 | D10404 |
| hsa10673 | D03068 | hsa1234 | D06297 | hsa3448 | D09662 | hsa3579 | D10420 |
| hsa6347 | D03116 | hsa1435 | D06402 | hsa3449 | D09662 | hsa51561 | D10438 |
| hsa5617 | D03165 | hsa7422 | D06409 | hsa3451 | D09662 | hsa4352 | D10476 |
| hsa1234 | D03210 | hsa3592 | D06556 | hsa3452 | D09662 | hsa3454 | D10483 |
| hsa2057 | D03231 | hsa3593 | D06556 | hsa3439 | D09668 | hsa3455 | D10483 |
| hsa2057 | D03232 | hsa51561 | D06556 | hsa3440 | D09668 | hsa51311 | D10716 |
| hsa1441 | D03235 | hsa1234 | D06557 | hsa3441 | D09668 | hsa5228 | D10819 |
| hsa1441 | D03245 | hsa8795 | D06611 | hsa3442 | D09668 | hsa7422 | D10819 |
| hsa1441 | D03247 | hsa64127 | D06619 | hsa3443 | D09668 | hsa7423 | D10819 |
| hsa2690 | D03297 | hsa7099 | D06619 | hsa3444 | D09668 | hsa2057 | D10846 |
| hsa3454 | D03304 | hsa3552 | D06635 | hsa3445 | D09668 | hsa2057 | D10847 |
| hsa3455 | D03304 | hsa3553 | D06635 | hsa3446 | D09668 | hsa3605 | D10902 |
| hsa3454 | D03305 | hsa1234 | D06670 | hsa3447 | D09668 | hsa51561 | D10912 |
| hsa3455 | D03305 | hsa1441 | D06889 | hsa3448 | D09668 | hsa8074 | D10913 |
| hsa3459 | D03357 | hsa7124 | D07436 | hsa3449 | D09668 | hsa729230 | D10972 |
| hsa3460 | D03357 | hsa1435 | D08552 | hsa3451 | D09668 | hsa51284 | D11003 |
| hsa7124 | D03441 | hsa7132 | D08562 | hsa3452 | D09668 | hsa1441 | D11026 |
| hsa3559 | D03639 | hsa7133 | D08562 | hsa3569 | D09669 | hsa3454 | D11027 |
| hsa3563 | D03645 | hsa3459 | D08805 | hsa10673 | D09704 | hsa3455 | D11027 |
| hsa1271 | D03647 | hsa3460 | D08805 | hsa8741 | D09704 | hsa2660 | D11047 |
| hsa2057 | D03651 | hsa54106 | D08841 | hsa1233 | D09761 | hsa51561 | D11052 |
| hsa3559 | D03682 | hsa54106 | D08842 | hsa7124 | D09813 | hsa3570 | D11079 |
| hsa3560 | D03682 | hsa3454 | D08844 | hsa3568 | D09874 | hsa133396 | D11080 |
| hsa3561 | D03682 | hsa3455 | D08844 | hsa6347 | D09877 | hsa3454 | D11082 |
| hsa8600 | D03684 | hsa3596 | D08857 | hsa1234 | D09878 | hsa3455 | D11082 |
| hsa4352 | D03978 | hsa4050 | D08866 | hsa729230 | D09878 | hsa7422 | D11083 |
| hsa2057 | D04032 | hsa7422 | D08874 | hsa1234 | D09879 |  |  |

Table6. The overall of drug-target interactions for Cell surface molecules and ligands class on new dataset 1

| **drug** | **protein** | **drug** | **protein** | **drug** | **protein** | **drug** | **protein** |
| --- | --- | --- | --- | --- | --- | --- | --- |
| hsa3674 | D01029 | hsa3127 | D04318 | hsa634 | D06350 | hsa915 | D09207 |
| hsa3690 | D01029 | hsa3133 | D04318 | hsa90273 | D06350 | hsa916 | D09207 |
| hsa3674 | D02778 | hsa3134 | D04318 | hsa1048 | D06351 | hsa917 | D09207 |
| hsa3690 | D02778 | hsa3135 | D04318 | hsa1084 | D06351 | hsa931 | D09314 |
| hsa965 | D02800 | hsa931 | D04489 | hsa1087 | D06351 | hsa931 | D09321 |
| hsa1043 | D02802 | hsa1048 | D04524 | hsa1088 | D06351 | hsa915 | D09325 |
| hsa3383 | D02811 | hsa1084 | D04524 | hsa1089 | D06351 | hsa916 | D09325 |
| hsa3123 | D02967 | hsa1087 | D04524 | hsa4680 | D06351 | hsa917 | D09325 |
| hsa3125 | D02967 | hsa1088 | D04524 | hsa634 | D06351 | hsa930 | D09325 |
| hsa3126 | D02967 | hsa1089 | D04524 | hsa90273 | D06351 | hsa57823 | D09337 |
| hsa3127 | D02967 | hsa4680 | D04524 | hsa920 | D06356 | hsa3685 | D09342 |
| hsa1048 | D02977 | hsa634 | D04524 | hsa921 | D06381 | hsa3690 | D09342 |
| hsa1084 | D02977 | hsa90273 | D04524 | hsa2208 | D06463 | hsa920 | D09575 |
| hsa1087 | D02977 | hsa1493 | D04603 | hsa3676 | D06590 | hsa3685 | D09631 |
| hsa1088 | D02977 | hsa3674 | D04659 | hsa3674 | D06649 | hsa4582 | D09700 |
| hsa1089 | D02977 | hsa3690 | D04659 | hsa3690 | D06649 | hsa4582 | D09895 |
| hsa4582 | D02977 | hsa3674 | D04660 | hsa1493 | D06657 | hsa3695 | D09901 |
| hsa4680 | D02977 | hsa3690 | D04660 | hsa3676 | D06886 | hsa3685 | D09903 |
| hsa634 | D02977 | hsa3674 | D04785 | hsa3674 | D06888 | hsa3690 | D09903 |
| hsa90273 | D02977 | hsa3690 | D04785 | hsa3690 | D06888 | hsa3693 | D09903 |
| hsa931 | D02994 | hsa915 | D05092 | hsa3676 | D08083 | hsa4684 | D09927 |
| hsa933 | D03066 | hsa916 | D05092 | hsa3695 | D08083 | hsa3685 | D09929 |
| hsa6401 | D03114 | hsa917 | D05092 | hsa3674 | D08607 | hsa3690 | D09929 |
| hsa6402 | D03114 | hsa931 | D05218 | hsa3690 | D08607 | hsa933 | D09932 |
| hsa6403 | D03114 | hsa3674 | D05267 | hsa931 | D08621 | hsa4345 | D09961 |
| hsa941 | D03203 | hsa3690 | D05267 | hsa931 | D08622 | hsa3676 | D10028 |
| hsa942 | D03203 | hsa94025 | D05269 | hsa933 | D08933 | hsa3688 | D10028 |
| hsa941 | D03222 | hsa920 | D05610 | hsa1048 | D08936 | hsa3695 | D10028 |
| hsa942 | D03222 | hsa3674 | D05772 | hsa1084 | D08936 | hsa5133 | D10316 |
| hsa945 | D03259 | hsa3690 | D05772 | hsa1087 | D08936 | hsa6403 | D10356 |
| hsa920 | D03420 | hsa3674 | D05834 | hsa1088 | D08936 | hsa3683 | D10374 |
| hsa3685 | D03497 | hsa3690 | D05834 | hsa1089 | D08936 | hsa5133 | D10390 |
| hsa3690 | D03497 | hsa914 | D05847 | hsa4680 | D08936 | hsa3683 | D10392 |
| hsa3693 | D03497 | hsa1048 | D06028 | hsa634 | D08936 | hsa3802 | D10444 |
| hsa4072 | D03958 | hsa1084 | D06028 | hsa90273 | D08936 | hsa3803 | D10444 |
| hsa3683 | D03959 | hsa1087 | D06028 | hsa972 | D08944 | hsa3804 | D10444 |
| hsa3674 | D03971 | hsa1088 | D06028 | hsa915 | D08959 | hsa931 | D10482 |
| hsa3690 | D03971 | hsa1089 | D06028 | hsa916 | D08959 | hsa6401 | D10487 |
| hsa3383 | D04000 | hsa4582 | D06028 | hsa917 | D08959 | hsa8829 | D10494 |
| hsa933 | D04036 | hsa4680 | D06028 | hsa3683 | D08993 | hsa3676 | D10540 |
| hsa3683 | D04045 | hsa634 | D06028 | hsa3684 | D08993 | hsa3695 | D10540 |
| hsa3689 | D04045 | hsa90273 | D06028 | hsa3689 | D08993 | hsa5133 | D10574 |
| hsa941 | D04295 | hsa6403 | D06194 | hsa915 | D09013 | hsa29126 | D10773 |
| hsa3105 | D04318 | hsa915 | D06314 | hsa916 | D09013 | hsa29126 | D10808 |
| hsa3106 | D04318 | hsa916 | D06314 | hsa917 | D09013 | hsa29126 | D10817 |
| hsa3107 | D04318 | hsa917 | D06314 | hsa4072 | D09027 | hsa1048 | D10917 |
| hsa3108 | D04318 | hsa3678 | D06319 | hsa931 | D09031 | hsa1084 | D10917 |
| hsa3109 | D04318 | hsa3688 | D06319 | hsa3683 | D09190 | hsa1087 | D10917 |
| hsa3111 | D04318 | hsa3674 | D06335 | hsa914 | D09190 | hsa1088 | D10917 |
| hsa3112 | D04318 | hsa3690 | D06335 | hsa915 | D09190 | hsa1089 | D10917 |
| hsa3113 | D04318 | hsa933 | D06348 | hsa916 | D09190 | hsa4680 | D10917 |
| hsa3115 | D04318 | hsa933 | D06349 | hsa917 | D09190 | hsa634 | D10917 |
| hsa3117 | D04318 | hsa1048 | D06350 | hsa920 | D09190 | hsa90273 | D10917 |
| hsa3118 | D04318 | hsa1084 | D06350 | hsa921 | D09190 | hsa1490 | D10969 |
| hsa3119 | D04318 | hsa1087 | D06350 | hsa924 | D09190 | hsa4070 | D10985 |
| hsa3122 | D04318 | hsa1088 | D06350 | hsa925 | D09190 | hsa51744 | D10993 |
| hsa3123 | D04318 | hsa1089 | D06350 | hsa926 | D09190 | hsa925 | D10993 |
| hsa3125 | D04318 | hsa4680 | D06350 | hsa4072 | D09207 | hsa926 | D10993 |
| hsa3126 | D04318 |  |  |  |  |  |  |

Table7. The overall of drug-target interactions for Protein kinases class on new dataset 1

| **drug** | **protein** | **drug** | **protein** | **drug** | **protein** | **drug** | **protein** |
| --- | --- | --- | --- | --- | --- | --- | --- |
| hsa3643 | D00085 | hsa6714 | D06414 | hsa6098 | D09731 | hsa2261 | D10396 |
| hsa5562 | D00595 | hsa7525 | D06414 | hsa1956 | D09733 | hsa2263 | D10396 |
| hsa5563 | D00595 | hsa2064 | D06610 | hsa2064 | D09733 | hsa2321 | D10396 |
| hsa2475 | D00753 | hsa2984 | D06612 | hsa2066 | D09733 | hsa2324 | D10396 |
| hsa5562 | D00944 | hsa2475 | D06669 | hsa3480 | D09746 | hsa3791 | D10396 |
| hsa5563 | D00944 | hsa2321 | D06678 | hsa3716 | D09783 | hsa5156 | D10396 |
| hsa3480 | D00967 | hsa2324 | D06678 | hsa3717 | D09783 | hsa5159 | D10396 |
| hsa25 | D01441 | hsa3791 | D06678 | hsa3718 | D09783 | hsa1111 | D10397 |
| hsa3815 | D01441 | hsa3815 | D06678 | hsa7297 | D09783 | hsa25 | D10399 |
| hsa5156 | D01441 | hsa5156 | D06678 | hsa3815 | D09864 | hsa7010 | D10399 |
| hsa6093 | D01840 | hsa5159 | D06678 | hsa4233 | D09864 | hsa5604 | D10405 |
| hsa9475 | D01840 | hsa5979 | D06678 | hsa558 | D09864 | hsa5605 | D10405 |
| hsa1956 | D01977 | hsa1956 | D07907 | hsa3815 | D09865 | hsa5156 | D10411 |
| hsa5562 | D02206 | hsa6093 | D07941 | hsa4233 | D09865 | hsa2321 | D10423 |
| hsa5563 | D02206 | hsa9475 | D07941 | hsa558 | D09865 | hsa5156 | D10423 |
| hsa1436 | D02664 | hsa25 | D08066 | hsa1017 | D09868 | hsa5159 | D10423 |
| hsa2475 | D02714 | hsa3815 | D08066 | hsa1019 | D09868 | hsa6714 | D10423 |
| hsa1017 | D02880 | hsa5156 | D08066 | hsa1021 | D09868 | hsa6790 | D10423 |
| hsa1019 | D02880 | hsa3643 | D08080 | hsa1022 | D09868 | hsa6795 | D10423 |
| hsa1021 | D02880 | hsa1956 | D08108 | hsa983 | D09868 | hsa9212 | D10423 |
| hsa1022 | D02880 | hsa2064 | D08108 | hsa1956 | D09883 | hsa5604 | D10426 |
| hsa983 | D02880 | hsa2322 | D08279 | hsa2064 | D09883 | hsa5605 | D10426 |
| hsa4881 | D02935 | hsa25 | D08279 | hsa2066 | D09883 | hsa7046 | D10437 |
| hsa4882 | D02935 | hsa6790 | D08279 | hsa3480 | D09908 | hsa1956 | D10439 |
| hsa3815 | D02937 | hsa6795 | D08279 | hsa2260 | D09919 | hsa2064 | D10446 |
| hsa5578 | D02970 | hsa9212 | D08279 | hsa2261 | D09919 | hsa238 | D10450 |
| hsa3717 | D03003 | hsa2322 | D08344 | hsa2263 | D09919 | hsa5580 | D10456 |
| hsa3718 | D03003 | hsa25 | D08344 | hsa2264 | D09919 | hsa5580 | D10457 |
| hsa3717 | D03004 | hsa6790 | D08344 | hsa2321 | D09919 | hsa6093 | D10463 |
| hsa3718 | D03004 | hsa6795 | D08344 | hsa2324 | D09919 | hsa9475 | D10463 |
| hsa5159 | D03065 | hsa9212 | D08344 | hsa3791 | D09919 | hsa3791 | D10465 |
| hsa6093 | D03115 | hsa5562 | D08351 | hsa5979 | D09919 | hsa4233 | D10465 |
| hsa9475 | D03115 | hsa5563 | D08351 | hsa2260 | D09920 | hsa3643 | D10473 |
| hsa2321 | D03218 | hsa5562 | D08352 | hsa2261 | D09920 | hsa2260 | D10481 |
| hsa2324 | D03218 | hsa5563 | D08352 | hsa2263 | D09920 | hsa2261 | D10481 |
| hsa3791 | D03218 | hsa3791 | D08503 | hsa2264 | D09920 | hsa2263 | D10481 |
| hsa3643 | D03230 | hsa3815 | D08503 | hsa2321 | D09920 | hsa2321 | D10481 |
| hsa3643 | D03250 | hsa5156 | D08503 | hsa2324 | D09920 | hsa2324 | D10481 |
| hsa25 | D03252 | hsa5159 | D08503 | hsa3791 | D09920 | hsa3791 | D10481 |
| hsa6714 | D03252 | hsa2322 | D08524 | hsa5979 | D09920 | hsa5156 | D10481 |
| hsa2064 | D03257 | hsa2324 | D08524 | hsa3480 | D09925 | hsa5159 | D10481 |
| hsa3480 | D03297 | hsa3791 | D08524 | hsa2321 | D09926 | hsa5604 | D10486 |
| hsa4881 | D03328 | hsa3815 | D08524 | hsa5156 | D09939 | hsa5605 | D10486 |
| hsa1956 | D03350 | hsa5159 | D08524 | hsa4233 | D09941 | hsa1956 | D10514 |
| hsa2064 | D03350 | hsa5894 | D08524 | hsa3791 | D09945 | hsa2064 | D10514 |
| hsa2065 | D03350 | hsa5979 | D08524 | hsa2984 | D09948 | hsa2066 | D10514 |
| hsa2066 | D03350 | hsa673 | D08524 | hsa25 | D09950 | hsa238 | D10542 |
| hsa1956 | D03455 | hsa3791 | D08544 | hsa25 | D09951 | hsa2475 | D10543 |
| hsa1969 | D03658 | hsa3815 | D08544 | hsa2322 | D09955 | hsa238 | D10551 |
| hsa25 | D03658 | hsa5156 | D08544 | hsa2322 | D09956 | hsa2475 | D10552 |
| hsa2534 | D03658 | hsa5159 | D08544 | hsa3716 | D09959 | hsa3718 | D10585 |
| hsa3815 | D03658 | hsa2321 | D08552 | hsa3717 | D09959 | hsa5604 | D10604 |
| hsa3932 | D03658 | hsa2322 | D08552 | hsa3716 | D09960 | hsa5605 | D10604 |
| hsa5159 | D03658 | hsa2324 | D08552 | hsa3717 | D09960 | hsa5604 | D10615 |
| hsa6714 | D03658 | hsa3791 | D08552 | hsa3716 | D09970 | hsa5605 | D10615 |
| hsa7525 | D03658 | hsa3815 | D08552 | hsa3717 | D09970 | hsa2475 | D10616 |
| hsa1432 | D03736 | hsa5156 | D08552 | hsa3718 | D09970 | hsa2185 | D10618 |
| hsa5600 | D03736 | hsa5159 | D08552 | hsa7297 | D09970 | hsa2185 | D10619 |
| hsa5603 | D03736 | hsa5979 | D08552 | hsa2064 | D09980 | hsa92 | D10620 |
| hsa6300 | D03736 | hsa3480 | D08620 | hsa673 | D09996 | hsa93 | D10620 |
| hsa659 | D03779 | hsa2260 | D08878 | hsa1956 | D10018 | hsa3717 | D10630 |
| hsa5579 | D04014 | hsa3791 | D08878 | hsa5604 | D10024 | hsa2475 | D10635 |
| hsa1956 | D04023 | hsa2321 | D08881 | hsa5605 | D10024 | hsa10000 | D10641 |
| hsa1956 | D04024 | hsa2324 | D08881 | hsa1956 | D10031 | hsa207 | D10641 |
| hsa2064 | D04024 | hsa3791 | D08881 | hsa3480 | D10056 | hsa208 | D10641 |
| hsa2064 | D04025 | hsa2321 | D08883 | hsa2321 | D10062 | hsa1019 | D10652 |
| hsa3551 | D04050 | hsa2324 | D08883 | hsa2324 | D10062 | hsa1021 | D10652 |
| hsa4881 | D04051 | hsa3791 | D08883 | hsa3791 | D10062 | hsa3716 | D10653 |
| hsa4882 | D04051 | hsa2475 | D08900 | hsa3815 | D10062 | hsa3717 | D10653 |
| hsa3643 | D04475 | hsa2261 | D08907 | hsa5156 | D10062 | hsa3718 | D10653 |
| hsa3643 | D04477 | hsa2321 | D08907 | hsa5159 | D10062 | hsa1432 | D10658 |
| hsa3643 | D04539 | hsa2322 | D08907 | hsa5979 | D10062 | hsa5600 | D10658 |
| hsa3643 | D04540 | hsa2324 | D08907 | hsa4881 | D10063 | hsa5603 | D10658 |
| hsa3643 | D04541 | hsa3791 | D08907 | hsa4882 | D10063 | hsa6300 | D10658 |
| hsa3643 | D04542 | hsa3815 | D08907 | hsa673 | D10064 | hsa1432 | D10659 |
| hsa3643 | D04543 | hsa5159 | D08907 | hsa4486 | D10074 | hsa5600 | D10659 |
| hsa3643 | D04544 | hsa2321 | D08947 | hsa2475 | D10076 | hsa5603 | D10659 |
| hsa3643 | D04545 | hsa2324 | D08947 | hsa6790 | D10085 | hsa6300 | D10659 |
| hsa3643 | D04546 | hsa3791 | D08947 | hsa6790 | D10086 | hsa10000 | D10674 |
| hsa3643 | D04547 | hsa3815 | D08947 | hsa2321 | D10095 | hsa207 | D10674 |
| hsa3643 | D04548 | hsa5156 | D08947 | hsa2324 | D10095 | hsa208 | D10674 |
| hsa3643 | D04549 | hsa5159 | D08947 | hsa3791 | D10095 | hsa1019 | D10688 |
| hsa3643 | D04550 | hsa5979 | D08947 | hsa3815 | D10095 | hsa1021 | D10688 |
| hsa3643 | D04551 | hsa1956 | D08950 | hsa5156 | D10095 | hsa4233 | D10696 |
| hsa2322 | D04696 | hsa2064 | D08950 | hsa5159 | D10095 | hsa2322 | D10709 |
| hsa1956 | D04862 | hsa2066 | D08950 | hsa5979 | D10095 | hsa558 | D10709 |
| hsa3480 | D04870 | hsa25 | D08953 | hsa2322 | D10102 | hsa2475 | D10718 |
| hsa5562 | D04966 | hsa3815 | D08953 | hsa5156 | D10102 | hsa3716 | D10721 |
| hsa5563 | D04966 | hsa5156 | D08953 | hsa5159 | D10102 | hsa3717 | D10721 |
| hsa2322 | D05029 | hsa5159 | D08953 | hsa2322 | D10103 | hsa3718 | D10721 |
| hsa3791 | D05029 | hsa1432 | D08963 | hsa5156 | D10103 | hsa3716 | D10728 |
| hsa3815 | D05029 | hsa3480 | D09328 | hsa5159 | D10103 | hsa695 | D10730 |
| hsa5156 | D05029 | hsa3480 | D09345 | hsa673 | D10104 | hsa695 | D10731 |
| hsa5159 | D05029 | hsa6850 | D09347 | hsa94 | D10106 | hsa6093 | D10737 |
| hsa5578 | D05029 | hsa6850 | D09348 | hsa2260 | D10137 | hsa9475 | D10737 |
| hsa5579 | D05029 | hsa2984 | D09355 | hsa2321 | D10137 | hsa6093 | D10738 |
| hsa5582 | D05029 | hsa3791 | D09371 | hsa2324 | D10137 | hsa9475 | D10738 |
| hsa4881 | D05147 | hsa1432 | D09386 | hsa3791 | D10137 | hsa1956 | D10766 |
| hsa4881 | D05148 | hsa2260 | D09589 | hsa3815 | D10137 | hsa2322 | D10800 |
| hsa2263 | D05338 | hsa3791 | D09589 | hsa5159 | D10137 | hsa558 | D10800 |
| hsa1956 | D05350 | hsa1432 | D09602 | hsa5894 | D10137 | hsa1956 | D10858 |
| hsa2321 | D05380 | hsa5600 | D09602 | hsa5979 | D10137 | hsa1956 | D10859 |
| hsa2324 | D05380 | hsa5603 | D09602 | hsa673 | D10137 | hsa3791 | D10862 |
| hsa3791 | D05380 | hsa6300 | D09602 | hsa7010 | D10137 | hsa4233 | D10862 |
| hsa3815 | D05380 | hsa1432 | D09603 | hsa2260 | D10138 | hsa7010 | D10862 |
| hsa5156 | D05380 | hsa5600 | D09603 | hsa2321 | D10138 | hsa238 | D10866 |
| hsa5159 | D05380 | hsa5603 | D09603 | hsa2324 | D10138 | hsa3716 | D10871 |
| hsa1956 | D05399 | hsa6300 | D09603 | hsa3791 | D10138 | hsa3716 | D10872 |
| hsa2064 | D05446 | hsa1017 | D09604 | hsa3815 | D10138 | hsa1019 | D10883 |
| hsa3643 | D05622 | hsa1020 | D09604 | hsa5159 | D10138 | hsa1021 | D10883 |
| hsa5579 | D05784 | hsa1025 | D09604 | hsa5894 | D10138 | hsa695 | D10893 |
| hsa5580 | D05784 | hsa983 | D09604 | hsa5979 | D10138 | hsa1956 | D10898 |
| hsa5581 | D05784 | hsa3791 | D09618 | hsa673 | D10138 | hsa2064 | D10898 |
| hsa5579 | D05785 | hsa4233 | D09618 | hsa7010 | D10138 | hsa2066 | D10898 |
| hsa5580 | D05785 | hsa2321 | D09635 | hsa3716 | D10141 | hsa23476 | D10911 |
| hsa5581 | D05785 | hsa2324 | D09635 | hsa3716 | D10142 | hsa6046 | D10911 |
| hsa2322 | D05819 | hsa3791 | D09635 | hsa5347 | D10154 | hsa8019 | D10911 |
| hsa3791 | D05819 | hsa5156 | D09635 | hsa5347 | D10155 | hsa238 | D10926 |
| hsa3815 | D05819 | hsa5159 | D09635 | hsa5599 | D10168 | hsa4914 | D10926 |
| hsa2322 | D06005 | hsa1432 | D09639 | hsa5601 | D10168 | hsa4915 | D10926 |
| hsa3815 | D06005 | hsa5600 | D09639 | hsa5602 | D10168 | hsa4916 | D10926 |
| hsa5159 | D06005 | hsa5603 | D09639 | hsa4233 | D10173 | hsa2260 | D10927 |
| hsa2475 | D06068 | hsa6300 | D09639 | hsa5604 | D10175 | hsa2261 | D10927 |
| hsa2322 | D06272 | hsa1956 | D09660 | hsa5605 | D10175 | hsa2263 | D10927 |
| hsa2324 | D06272 | hsa25 | D09664 | hsa5604 | D10176 | hsa2264 | D10927 |
| hsa3791 | D06272 | hsa6714 | D09664 | hsa5605 | D10176 | hsa2065 | D10943 |
| hsa3815 | D06272 | hsa25 | D09665 | hsa5347 | D10182 | hsa3480 | D10943 |
| hsa5159 | D06272 | hsa6714 | D09665 | hsa5347 | D10183 | hsa3716 | D10944 |
| hsa5894 | D06272 | hsa5604 | D09666 | hsa2321 | D10190 | hsa3716 | D10945 |
| hsa5979 | D06272 | hsa5605 | D09666 | hsa2324 | D10190 | hsa1956 | D10958 |
| hsa673 | D06272 | hsa92 | D09670 | hsa3791 | D10190 | hsa1432 | D10959 |
| hsa2321 | D06285 | hsa5580 | D09671 | hsa25 | D10202 | hsa5600 | D10959 |
| hsa2324 | D06285 | hsa5581 | D09671 | hsa4067 | D10202 | hsa5603 | D10959 |
| hsa3791 | D06285 | hsa5583 | D09671 | hsa695 | D10223 | hsa6300 | D10959 |
| hsa3815 | D06285 | hsa5588 | D09671 | hsa3791 | D10224 | hsa2984 | D10976 |
| hsa5159 | D06285 | hsa3480 | D09680 | hsa4233 | D10224 | hsa1019 | D10979 |
| hsa2321 | D06402 | hsa2321 | D09683 | hsa3815 | D10229 | hsa1021 | D10979 |
| hsa2322 | D06402 | hsa2324 | D09683 | hsa3716 | D10308 | hsa4217 | D10988 |
| hsa2324 | D06402 | hsa3791 | D09683 | hsa3717 | D10308 | hsa3716 | D10994 |
| hsa3791 | D06402 | hsa1956 | D09689 | hsa3716 | D10315 | hsa3716 | D10995 |
| hsa3815 | D06402 | hsa2064 | D09689 | hsa3717 | D10315 | hsa6093 | D11030 |
| hsa5156 | D06402 | hsa2066 | D09689 | hsa25 | D10334 | hsa9475 | D11030 |
| hsa5159 | D06402 | hsa1956 | D09690 | hsa7010 | D10334 | hsa6093 | D11031 |
| hsa5979 | D06402 | hsa2064 | D09690 | hsa2064 | D10344 | hsa9475 | D11031 |
| hsa1956 | D06407 | hsa2066 | D09690 | hsa3716 | D10358 | hsa5594 | D11038 |
| hsa3791 | D06407 | hsa5580 | D09718 | hsa3717 | D10358 | hsa5595 | D11038 |
| hsa5979 | D06407 | hsa5581 | D09718 | hsa3717 | D10365 | hsa3716 | D11046 |
| hsa25 | D06413 | hsa5583 | D09718 | hsa1019 | D10372 | hsa3717 | D11046 |
| hsa3815 | D06413 | hsa5588 | D09718 | hsa1021 | D10372 | hsa3718 | D11046 |
| hsa5156 | D06413 | hsa1956 | D09724 | hsa10000 | D10381 | hsa3716 | D11048 |
| hsa5159 | D06413 | hsa2064 | D09724 | hsa207 | D10381 | hsa673 | D11053 |
| hsa1969 | D06414 | hsa2066 | D09724 | hsa208 | D10381 | hsa695 | D11070 |
| hsa25 | D06414 | hsa3643 | D09727 | hsa10000 | D10382 | hsa4233 | D11073 |
| hsa2534 | D06414 | hsa25 | D09728 | hsa207 | D10382 | hsa10000 | D11074 |
| hsa3815 | D06414 | hsa6714 | D09728 | hsa208 | D10382 | hsa207 | D11074 |
| hsa3932 | D06414 | hsa238 | D09731 | hsa2260 | D10396 | hsa208 | D11074 |
| hsa5159 | D06414 |  |  |  |  |  |  |

Table8. The overall of drug-target interactions for Transporters class on new dataset 1

| **drug** | **target** | **drug** | **target** | **drug** | **target** | **drug** | **target** |
| --- | --- | --- | --- | --- | --- | --- | --- |
| hsa6530 | D00110 | hsa6833 | D01810 | hsa10060 | D05482 | hsa6524 | D08897 |
| hsa6531 | D00110 | hsa6833 | D01854 | hsa6833 | D05482 | hsa2950 | D08917 |
| hsa6532 | D00110 | hsa6559 | D01877 | hsa6532 | D05491 | hsa931 | D09031 |
| hsa6570 | D00197 | hsa6559 | D01895 | hsa6530 | D05663 | hsa6548 | D09036 |
| hsa6571 | D00197 | hsa9900 | D01914 | hsa6531 | D05663 | hsa931 | D09314 |
| hsa6833 | D00219 | hsa6530 | D02074 | hsa6530 | D05997 | hsa931 | D09321 |
| hsa6530 | D00228 | hsa6531 | D02074 | hsa6530 | D06007 | hsa6530 | D09340 |
| hsa6532 | D00228 | hsa6571 | D02074 | hsa5243 | D06008 | hsa6530 | D09341 |
| hsa6557 | D00247 | hsa6530 | D02078 | hsa6833 | D06177 | hsa2348 | D09343 |
| hsa6558 | D00247 | hsa6531 | D02078 | hsa1071 | D06195 | hsa6529 | D09539 |
| hsa6560 | D00247 | hsa6571 | D02078 | hsa5243 | D06277 | hsa6524 | D09592 |
| hsa6833 | D00271 | hsa6530 | D02080 | hsa5243 | D06387 | hsa6007 | D09663 |
| hsa6557 | D00272 | hsa6529 | D02097 | hsa6559 | D06401 | hsa7276 | D09673 |
| hsa6559 | D00272 | hsa6530 | D02170 | hsa6524 | D06641 | hsa7276 | D09674 |
| hsa6833 | D00294 | hsa6532 | D02170 | hsa6571 | D07097 | hsa6532 | D09698 |
| hsa6557 | D00313 | hsa6530 | D02182 | hsa6833 | D07117 | hsa6532 | D09699 |
| hsa6558 | D00313 | hsa6531 | D02182 | hsa6833 | D07118 | hsa1071 | D09708 |
| hsa6532 | D00326 | hsa6532 | D02182 | hsa10280 | D07316 | hsa6524 | D09763 |
| hsa6557 | D00331 | hsa6530 | D02237 | hsa6530 | D07334 | hsa6557 | D09772 |
| hsa6558 | D00331 | hsa6530 | D02242 | hsa6532 | D07334 | hsa6558 | D09772 |
| hsa6529 | D00332 | hsa6531 | D02242 | hsa6530 | D07444 | hsa6530 | D09890 |
| hsa6833 | D00335 | hsa6532 | D02242 | hsa6531 | D07444 | hsa6530 | D09891 |
| hsa6833 | D00336 | hsa6570 | D02242 | hsa6530 | D07445 | hsa7357 | D09893 |
| hsa6559 | D00340 | hsa6571 | D02242 | hsa6531 | D07445 | hsa7357 | D09894 |
| hsa6559 | D00345 | hsa6532 | D02260 | hsa6571 | D07445 | hsa1080 | D09916 |
| hsa6530 | D00367 | hsa6532 | D02360 | hsa6530 | D07448 | hsa116085 | D09921 |
| hsa6531 | D00367 | hsa6532 | D02362 | hsa6532 | D07448 | hsa55867 | D09921 |
| hsa6532 | D00367 | hsa10060 | D02385 | hsa6530 | D07449 | hsa116085 | D09922 |
| hsa6833 | D00379 | hsa6833 | D02385 | hsa6532 | D07449 | hsa55867 | D09922 |
| hsa6833 | D00380 | hsa6557 | D02386 | hsa6530 | D07473 | hsa598 | D09935 |
| hsa6557 | D00382 | hsa6558 | D02386 | hsa6530 | D07591 | hsa599 | D09935 |
| hsa6558 | D00382 | hsa6530 | D02408 | hsa6531 | D07591 | hsa598 | D09936 |
| hsa6530 | D00394 | hsa6532 | D02408 | hsa6532 | D07704 | hsa599 | D09936 |
| hsa6532 | D00394 | hsa6833 | D02425 | hsa6532 | D07705 | hsa6524 | D09978 |
| hsa6833 | D00418 | hsa6833 | D02427 | hsa6530 | D07727 | hsa6531 | D10014 |
| hsa6559 | D00431 | hsa6833 | D02430 | hsa6532 | D07727 | hsa6524 | D10055 |
| hsa6557 | D00461 | hsa6557 | D02453 | hsa6530 | D07791 | hsa6530 | D10072 |
| hsa6559 | D00461 | hsa6559 | D02453 | hsa6532 | D07791 | hsa6532 | D10072 |
| hsa6530 | D00484 | hsa6530 | D02566 | hsa6530 | D07793 | hsa6530 | D10088 |
| hsa6532 | D00484 | hsa6532 | D02567 | hsa6532 | D07793 | hsa6531 | D10088 |
| hsa6559 | D00519 | hsa6530 | D02570 | hsa6532 | D07805 | hsa6532 | D10088 |
| hsa6833 | D00593 | hsa6532 | D02570 | hsa6530 | D07806 | hsa6530 | D10089 |
| hsa6833 | D00594 | hsa6530 | D02571 | hsa6531 | D07806 | hsa6531 | D10089 |
| hsa6559 | D00650 | hsa6531 | D02571 | hsa6532 | D07806 | hsa6532 | D10089 |
| hsa6559 | D00651 | hsa6532 | D02571 | hsa2030 | D07843 | hsa1071 | D10121 |
| hsa6557 | D00654 | hsa6530 | D02572 | hsa6530 | D07872 | hsa6530 | D10133 |
| hsa6559 | D00654 | hsa6530 | D02573 | hsa6532 | D07872 | hsa6532 | D10133 |
| hsa6557 | D00656 | hsa6530 | D02574 | hsa6530 | D07875 | hsa1080 | D10134 |
| hsa6559 | D00656 | hsa2030 | D02631 | hsa6532 | D07875 | hsa6570 | D10170 |
| hsa6559 | D00657 | hsa10280 | D02684 | hsa6530 | D07880 | hsa6571 | D10170 |
| hsa6557 | D00658 | hsa10280 | D02688 | hsa6532 | D07880 | hsa6570 | D10171 |
| hsa6559 | D00658 | hsa6530 | D02704 | hsa6532 | D07913 | hsa6571 | D10171 |
| hsa9900 | D00709 | hsa6532 | D02787 | hsa6530 | D07938 | hsa6532 | D10184 |
| hsa6530 | D00809 | hsa6531 | D02897 | hsa6531 | D07938 | hsa6532 | D10185 |
| hsa6532 | D00809 | hsa931 | D02994 | hsa6532 | D07945 | hsa6536 | D10186 |
| hsa6530 | D00811 | hsa5243 | D03128 | hsa6532 | D07984 | hsa6524 | D10195 |
| hsa6532 | D00811 | hsa6531 | D03153 | hsa6557 | D07999 | hsa6524 | D10196 |
| hsa6530 | D00812 | hsa6531 | D03215 | hsa6558 | D07999 | hsa6530 | D10199 |
| hsa6532 | D00812 | hsa6548 | D03406 | hsa6557 | D08001 | hsa6524 | D10200 |
| hsa6530 | D00814 | hsa6559 | D03471 | hsa6558 | D08001 | hsa6524 | D10313 |
| hsa6532 | D00814 | hsa6530 | D03491 | hsa6530 | D08030 | hsa2348 | D10434 |
| hsa6530 | D00815 | hsa6532 | D03491 | hsa6530 | D08070 | hsa2350 | D10434 |
| hsa6532 | D00815 | hsa6532 | D03649 | hsa6532 | D08070 | hsa2352 | D10434 |
| hsa6530 | D00816 | hsa6532 | D03713 | hsa6530 | D08071 | hsa390243 | D10434 |
| hsa6532 | D00816 | hsa6530 | D03721 | hsa6532 | D08071 | hsa6530 | D10443 |
| hsa6530 | D00817 | hsa6531 | D03721 | hsa6530 | D08072 | hsa6532 | D10443 |
| hsa6531 | D00817 | hsa6532 | D03721 | hsa6532 | D08072 | hsa6524 | D10459 |
| hsa6530 | D00818 | hsa6530 | D03740 | hsa6530 | D08077 | hsa931 | D10482 |
| hsa6532 | D00820 | hsa6531 | D03740 | hsa6532 | D08077 | hsa6523 | D10669 |
| hsa6530 | D00821 | hsa6571 | D03740 | hsa6530 | D08140 | hsa6524 | D10669 |
| hsa6532 | D00821 | hsa10280 | D03742 | hsa6532 | D08140 | hsa6570 | D10675 |
| hsa6532 | D00822 | hsa10280 | D03744 | hsa6530 | D08187 | hsa6571 | D10675 |
| hsa6532 | D00823 | hsa6530 | D03801 | hsa6531 | D08187 | hsa6530 | D10697 |
| hsa6532 | D00824 | hsa6531 | D03801 | hsa6532 | D08187 | hsa6531 | D10697 |
| hsa6532 | D00825 | hsa2030 | D03906 | hsa6570 | D08187 | hsa6532 | D10697 |
| hsa10280 | D00848 | hsa5243 | D03968 | hsa6571 | D08187 | hsa6530 | D10698 |
| hsa116085 | D01056 | hsa6557 | D04079 | hsa6530 | D08222 | hsa6531 | D10698 |
| hsa6530 | D01107 | hsa6558 | D04079 | hsa6532 | D08222 | hsa6532 | D10698 |
| hsa6532 | D01107 | hsa6557 | D04111 | hsa6530 | D08288 | hsa6530 | D10700 |
| hsa6833 | D01111 | hsa6558 | D04111 | hsa6532 | D08288 | hsa6531 | D10700 |
| hsa6530 | D01179 | hsa6532 | D04148 | hsa10280 | D08297 | hsa6571 | D10701 |
| hsa6532 | D01179 | hsa6530 | D04382 | hsa6530 | D08347 | hsa7276 | D10794 |
| hsa6559 | D01241 | hsa931 | D04489 | hsa6530 | D08348 | hsa6555 | D10795 |
| hsa6559 | D01246 | hsa10280 | D04502 | hsa6530 | D08447 | hsa6555 | D10796 |
| hsa6530 | D01285 | hsa6530 | D04999 | hsa6532 | D08447 | hsa6524 | D10865 |
| hsa6532 | D01285 | hsa6531 | D04999 | hsa6530 | D08472 | hsa6530 | D10870 |
| hsa6530 | D01296 | hsa7357 | D05032 | hsa6530 | D08513 | hsa6531 | D10870 |
| hsa6531 | D01296 | hsa6557 | D05093 | hsa6531 | D08513 | hsa201780 | D10951 |
| hsa6530 | D01304 | hsa6558 | D05093 | hsa6532 | D08513 | hsa345274 | D10951 |
| hsa6557 | D01323 | hsa6530 | D05173 | hsa6571 | D08575 | hsa347051 | D10951 |
| hsa6558 | D01323 | hsa6530 | D05200 | hsa6529 | D08588 | hsa6554 | D10951 |
| hsa6833 | D01356 | hsa931 | D05218 | hsa6557 | D08619 | hsa84068 | D10951 |
| hsa10280 | D01477 | hsa1071 | D05255 | hsa6558 | D08619 | hsa201780 | D10952 |
| hsa6530 | D01546 | hsa6532 | D05374 | hsa931 | D08621 | hsa345274 | D10952 |
| hsa6532 | D01546 | hsa6532 | D05375 | hsa931 | D08622 | hsa347051 | D10952 |
| hsa6833 | D01599 | hsa6530 | D05458 | hsa6532 | D08626 | hsa6554 | D10952 |
| hsa6559 | D01605 | hsa6531 | D05458 | hsa6530 | D08670 | hsa84068 | D10952 |
| hsa6557 | D01634 | hsa6532 | D05458 | hsa6532 | D08670 | hsa2348 | D10953 |
| hsa6558 | D01634 | hsa6530 | D05459 | hsa6530 | D08673 | hsa2348 | D10954 |
| hsa6833 | D01799 | hsa6531 | D05459 | hsa1071 | D08855 | hsa6570 | D10999 |
| hsa10060 | D01810 | hsa6532 | D05459 | hsa9900 | D08879 | hsa6571 | D10999 |
| hsa1080 | D11041 | hsa6524 | D11043 |  |  |  |  |
